# The landscape of host genetic factors involved in immune response to common viral infections

**DOI:** 10.1186/s13073-020-00790-x

**Published:** 2020-10-27

**Authors:** Linda Kachuri, Stephen S. Francis, Maike L. Morrison, George A. Wendt, Yohan Bossé, Taylor B. Cavazos, Sara R. Rashkin, Elad Ziv, John S. Witte

**Affiliations:** 1 Department of Epidemiology and Biostatistics, University of California San Francisco, San Francisco, CA USA; 2 Department of Neurological Surgery, University of California San Francisco, San Francisco, CA USA; 3 Helen Diller Family Comprehensive Cancer Center, University of California, San Francisco, San Francisco, CA USA; 4 Weill Institute for Neurosciences, University of California San Francisco, San Francisco, CA USA; 5 Department of Biology, Stanford University, Stanford, CA USA; 6 Summer Research Training Program, Graduate Division, University of California San Francisco, San Francisco, CA USA; 7 Department of Mathematics, The University of Texas, Austin, TX USA; 8 Department of Molecular Medicine, Université Laval, Institut Universitaire de Cardiologie et de Pneumologie de Québec, Quebec City, QC Canada; 9 Program in Biological and Medical Informatics, University of California San Francisco, San Francisco, CA USA; 10 Center for Applied Bioinformatics, St. Jude Children’s Research Hospital, Memphis, TN USA; 11 Department of Medicine, University of California, San Francisco, San Francisco, CA USA; 12 Institute for Human Genetics, University of California San Francisco, San Francisco, CA USA; 13 Department of Urology, University of California San Francisco, San Francisco, CA USA

**Keywords:** Infection, Virus, Serology, Antigen, Antibody, Immunoglobulin G, Immune response, Human leukocyte antigen (HLA), Polyomavirus, Genome-wide association study (GWAS), Transcriptome-wide association study (TWAS)

## Abstract

**Background:**

Humans and viruses have co-evolved for millennia resulting in a complex host genetic architecture. Understanding the genetic mechanisms of immune response to viral infection provides insight into disease etiology and therapeutic opportunities.

**Methods:**

We conducted a comprehensive study including genome-wide and transcriptome-wide association analyses to identify genetic loci associated with immunoglobulin G antibody response to 28 antigens for 16 viruses using serological data from 7924 European ancestry participants in the UK Biobank cohort.

**Results:**

Signals in human leukocyte antigen (HLA) class II region dominated the landscape of viral antibody response, with 40 independent loci and 14 independent classical alleles, 7 of which exhibited pleiotropic effects across viral families. We identified specific amino acid (AA) residues that are associated with seroreactivity, the strongest associations presented in a range of AA positions within DRβ1 at positions 11, 13, 71, and 74 for Epstein-Barr virus (EBV), Varicella zoster virus (VZV), human herpesvirus 7, (HHV7), and Merkel cell polyomavirus (MCV). Genome-wide association analyses discovered 7 novel genetic loci outside the HLA associated with viral antibody response (*P* < 5.0 × 10^−8^), including *FUT2* (19q13.33) for human polyomavirus BK (BKV), *STING1* (5q31.2) for MCV, and *CXCR5* (11q23.3) and *TBKBP1* (17q21.32) for HHV7. Transcriptome-wide association analyses identified 114 genes associated with response to viral infection, 12 outside of the HLA region, including *ECSCR*: *P* = 5.0 × 10^−15^ (MCV), *NTN5*: *P* = 1.1 × 10^−9^ (BKV), and *P2RY13*: *P* = 1.1 × 10^−8^ EBV nuclear antigen. We also demonstrated pleiotropy between viral response genes and complex diseases, from autoimmune disorders to cancer to neurodegenerative and psychiatric conditions.

**Conclusions:**

Our study confirms the importance of the HLA region in host response to viral infection and elucidates novel genetic determinants beyond the HLA that contribute to host-virus interaction.

## Background

Viruses have been infecting cells for a half a billion years [1]. During our extensive co-evolution, viruses have exerted significant selective pressure on humans and vice versa, overtly during fatal outbreaks, and covertly through cryptic immune interaction when a pathogen remains latent. The recent pandemic of severe acute respiratory syndrome coronavirus 2 (SARS-CoV-2) highlights the paramount public health need to understand human genetic variation in response to viral challenge. Clinical variation in COVID-19 severity and symptomatic presentation may be due to differences host genetic factors relating to immune response [2]. Furthermore, many common infections are cryptically associated with a variety of complex illnesses, especially those with an immunologic component, from cancer to autoimmune and neurologic conditions [3–5]. Despite their broad health relevance, few large-scale genome-wide association studies (GWAS) have been conducted on serological response phenotypes [6–10]. Understanding the genetic architecture of immunologic response to viruses may therefore provide new insight into etiologic mechanisms of diverse complex diseases.

Several common viruses exert a robust cell-mediated and humoral immune response that bi-directionally modulates the balance between latent and lytic infection. Studies have demonstrated a strong heritable component (32–48%) of antibody response [11] and identified associations between host polymorphisms in genes relating to cell entry, cytokine production, and immune response and a variety of viruses [12]. The predominance of previously reported associations has implicated genetic variants in human leucocyte antigen (HLA) class I and II genes in the modulation of immune response to diverse viral antigens [7, 13].

**Fig. 1:**
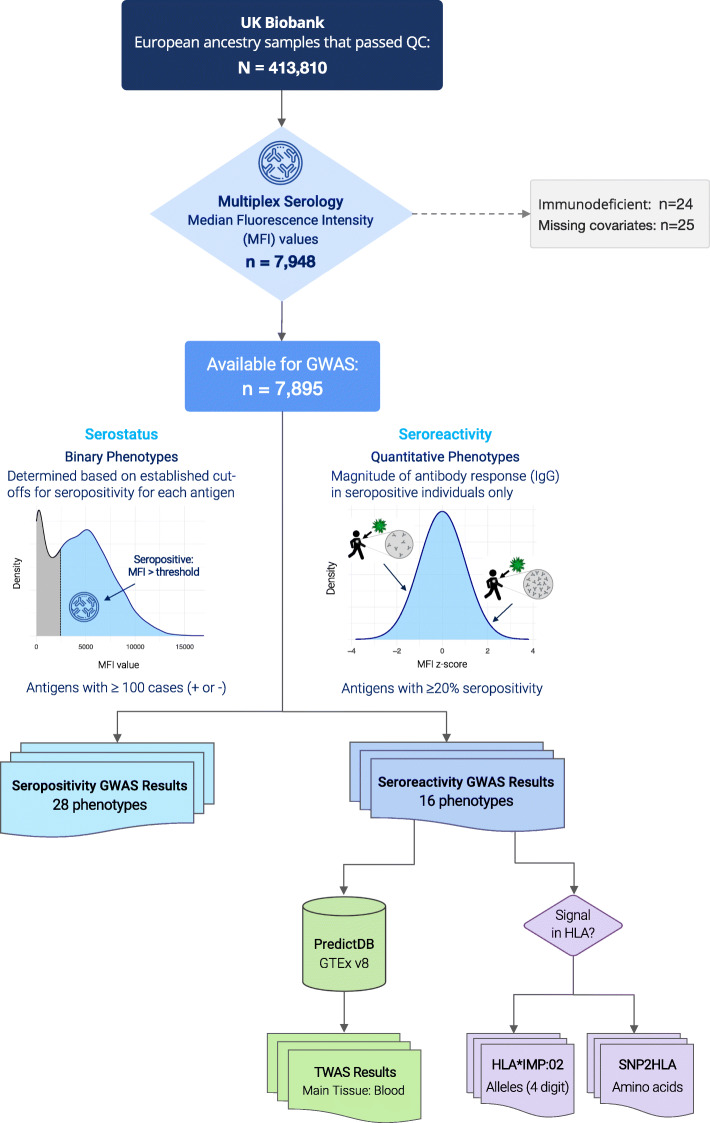
Flow chart describing the main serological phenotypes and association analyses

In this study, we utilize data from the UK Biobank (UKB) cohort [14] to evaluate the relationship between host genetics and immunoglobulin G antibody response to 28 antigens for 16 viruses. Immunoglobulin G (IgG) antibody is the most common antibody in blood, which serves as a stable biomarker of lifetime exposure to common viruses. High levels of specific IgG’s can be the result of chronic infection, while low levels may indicate poor immunity. Viruses assayed in the UKB multiplex serology panel were previously chosen based on putative links to chronic diseases including cancer, autoimmune, and neurodegenerative conditions [15]. We conduct integrative genome-wide and transcriptome-wide analyses of antibody response and positivity to viral antigens (Fig. 1), which elucidate novel genetic underpinnings of viral infection and immune response.

## Methods

### Study population and phenotypes

The UK Biobank (UKB) is a population-based prospective cohort of over 500,000 individuals aged 40–69 years at enrollment in 2006–2010 who completed extensive questionnaires and physical assessments and provided blood samples [14]. Analyses were restricted to individuals of predominantly European ancestry based on self-report and after excluding samples with any of the first two genetic ancestry principal components (PCs) outside of 5 standard deviations (SD) of the population mean (Additional file 1: Figure S1). We removed samples with discordant self-reported and genetic sex, samples with call rates < 97% or heterozygosity > 5 SD from the mean, and one sample from each pair of first-degree relatives identified using KING [16].

Of the 413,810 European ancestry individuals available for analysis, a total of 7948 had serological measures. A multiplex serology panel (IgG) was performed over a 2-week period using previously developed methods [17, 18] that have been successfully applied in epidemiological studies [7, 19]. Details of the serology methods and assay validation performance are described in Mentzer et al. [15] Briefly, multiplex serology was performed using a bead-based glutathione S-transferase (GST) capture assay with glutathione-casein coated fluorescence-labeled polystyrene beads and pathogen-specific GST-X-tag fusion proteins as antigens [15]. Each antigen was loaded onto a distinct bead set and the beads were simultaneously presented to primary serum antibodies at serum dilution 1:1000 [15]. Immunocomplexes were quantified using a Luminex 200 flow cytometer, which produced Median Fluorescence Intensities (MFI) for each antigen. The serology assay showed adequate performance, with a median coefficient of variation (CV) of 17% across all antigens and 3.5% among seropositive samples only [15].

### Genome-wide association analysis

We evaluated the relationship between genetic variants across the genome and serological phenotypes using PLINK 2.0 (October 2017 version) [20]. Participants were genotyped on the Affymetrix Axiom UK Biobank array (89%) or the UK BiLEVE array (11%) [14] with genome-wide imputation performed using the Haplotype Reference Consortium data and the merged UK10K and 1000 Genomes phase 3 reference panels [14]. We excluded variants out of Hardy-Weinberg equilibrium at *p* < 1 × 10^−5^, call rate < 95% (alternate allele dosage within 0.1 of the nearest hard call to be non-missing), imputation quality INFO< 0.30, and MAF < 0.01.

Seropositivity for each antigen was determined using established cut-offs based on prior validation work [15]. The primary GWAS focused on continuous phenotypes (MFI values), which measure the magnitude of antibody response, also referred to as seroreactivity. These analyses were conducted among seropositive individuals only for antigens with seroprevalence of ≥ 20% (*n* = 1500) based on 80% power to detect only common variants with large effect sizes at this sample size (Additional file 1: Figure S2). MFI values were transformed to standardized, normally distributed *z*-scores using ordered quantile normalization [21].

Seroreactivity GWAS was conducted using linear regression with adjustment for age at enrollment, sex, body mass index (BMI), socioeconomic status (Townsend deprivation index), the presence of any autoimmune and/or inflammatory conditions, genotyping array, serology assay date, quality control flag indicating sample spillover or an extra freeze/thaw cycle, and the top 10 genetic ancestry principal components (PC’s). Autoimmune and chronic inflammatory conditions were identified using the following primary and secondary diagnostic ICD-10 codes (E10, M00-03, M05-M14, M32, L20-L30, L40, G35, K50-52, K58, G61) in Hospital Episode Statistics. Individuals diagnosed with any immunodeficiency (ICD-10 D80-89, *n* = 24) were excluded from all analyses.

For all antigens with at least 100 seropositive (or seronegative for pathogens with ubiquitous exposure) individuals, GWAS of discrete seropositivity phenotypes was undertaken using logistic regression, adjusting for the same covariates listed above.

The functional relevance of the lead GWAS loci for antibody response was assessed using in silico functional annotation analyses based on Combined Annotation Dependent Depletion (CADD) [22] scores and RegulomeDB 2.0 [23] and by leveraging external datasets, such as GTEx v8, DICE (Database of Immune Cell Expression) [24], and the Human Plasma Proteome Atlas [25, 26].

### Cross-trait associations with disease

We explored pleiotropic associations between lead variants influencing antibody levels and several chronic diseases with known or hypothesized viral risk factors. Associations with selected cancers were obtained from a cancer pleiotropy meta-analysis of the UK Biobank and Genetic Epidemiology Research on Aging cohorts [27]. Summary statistics for the schizophrenia GWAS of 33,640 cases and 43,456 controls by Lam et al. [28] were downloaded from the Psychiatric Genomics Consortium. Association *p* values were obtained from the National Institute on Aging Genetics of Alzheimer’s Disease Data Storage Site for the GWAS by Jun et al. [29], which included 17,536 cases and 53,711 controls. Associations with *p* < 7.3 × 10^−4^ were considered statistically significant after correction for the number of variants and phenotypes tested.

### HLA regional analysis

For phenotypes displaying a genome-wide significant signal in the HLA region, independent association signals were ascertained using two complementary approaches: clumping and conditional analysis. Clumping is a post-processing step applied to GWAS summary statistics to identify independent association signals by grouping variants based on LD within specific windows. Clumping was performed on all variants with *P* < 5 × 10^−8^ for each phenotype, as well as across phenotypes. Clumps were formed around index variants with the lowest *p* value, and all other variants with LD *r*^2^ > 0.05 within a ± 500-kb window were considered non-independent and assigned to that variant’s clump.

Next, we conducted conditional analyses using a forward stepwise strategy to identify statistically independent signals within each type of variant (SNP/indel or classical HLA allele). Unlike clumping, conditional analyses involve fitting a new model that includes specific variants as covariates, thereby directly accounting for LD and providing association estimates that are adjusted for other relevant SNP effects. A total of 38,655 SNPs/indels on chromosome 6 (29,600,000–33,200,000 bp) were extracted to conduct regional analyses. Classical HLA alleles were imputed for UKB participants at 4-digit resolution using the HLA*IMP:02 algorithm [14], with modified settings to accommodate the addition of diverse samples from population reference panels described by Motyer et al. [30]. Details of the HLA imputation procedure are described in UKB Resource 182. Imputed dosages were available for 362 classical alleles in 11 genes: *HLA-A*, *HLA-B*, and *HLA-C* (class I); *HLA-DRB5*, *HLA-DRB4*, *HLA-DRB3*, *HLA-DRB1*, *HLA-DQA1*, *HLA-DQB1*, *HLA-DPA1*, and *HLA-DPB1* (class II). Allele names with “99:01” for DRB3/4/5, which denote copy number absence, were renamed as “00:00” to avoid confusion with traditional HLA nomenclature. We also used SNP2HLA [31] to impute HLA alleles and corresponding amino acid sequences at a 4-digit resolution in *HLA-A*, *HLA-B*, *HLA-C*, *HLA-DRB1*, *HLA-DQA1*, *HLA-DQB1*, *HLA-DPA1*, and *HLA-DPB1* using the Type 1 Diabetes Genetics Consortium (T1DGC) reference panel comprised of 2767 unrelated individuals of European descent. T1DGC was also among several reference datasets used by HLA*IMP:02. SNP2HLA imputation was conducted using 100-kb windows.

Analyses were restricted to common HLA alleles and amino acid sequences (frequency ≥ 0.01) with imputation quality scores > 0.30, for a total of 1081 markers (101 alleles + 980 amino acid residues). We performed uncertainty-aware analyses using the imputed allele dosages, which is preferred to hard-thresholding approaches [32]. Linear regression models were adjusted for the same set of covariates as the GWAS. Associations for each marker were considered statistically significant if *P* < 4.6 × 10^−5^ based on Bonferroni correction for 1081 tests.

For each antigen response phenotype, we identified SNPs/indels or classical HLA alleles with the lowest *p* value, among variants that achieved Bonferroni-significant associations (*P* < 4.6 × 10^−5^), and performed forward iterative conditional regression to identify other independent signals, until no associations with a conditional *p* value (*P*_cond_) < 5 × 10^−8^ remained. We also assessed the independence of associations across different types of genetic variants by including conditionally independent HLA alleles as covariates in the SNP-based analysis.

For amino acid positions with > 2 possible residues (alleles), we applied the haplotype omnibus test in PLINK 1.07 [33] to obtain an overall *p* value for jointly testing all possible substitutions at that specific position. The omnibus test was applied to all amino acid residues at a given position, even if not all substitutions achieved the Bonferroni-corrected threshold (*P* < 4.6 × 10^−5^) in the single-marker analysis. The frequency of amino acid substitutions at specific HLA alleles was determined using European ancestry reference populations part of the Allele Frequency Net Database (AFND 2020) [34].

### Transcriptome-wide association analysis

Gene transcription levels were imputed and analyzed using the MetaXcan approach [35] applied to GWAS summary statistics. For imputation, we used biologically informed MASHR-M prediction models [36] obtained from the PredictDB repository [37]. These models are based on GTEx v8 with effect sizes computed using MASHR (Multivariate Adaptive Shrinkage in R) [38] for variants fine-mapped with DAP-G (Deterministic Approximation of Posteriors) [39, 40]. An advantage of this approach is that MASHR effect sizes are smoothed by taking advantage of the correlation in cis-eQTL effects across tissues. For each antigen, we performed a transcriptome-wide association study (TWAS) using gene expression levels in whole blood. Statistically significant associations for each gene were determined based on the Bonferroni correction for the number of genes tested.

We also examined gene expression profiles in tissues that represent known infection targets or related pathologies. Human herpesviruses and polyomaviruses are neurotropic and have been implicated in several neurological conditions [41, 42]; therefore, we considered gene expression in the frontal cortex. For Epstein-Barr virus (EBV) antigens additional models included EBV-transformed lymphocytes. Merkel cell polyomavirus (MCV) is a known cause of Merkel cell carcinoma [43], a rare but aggressive type of skin cancer; therefore, we examined transcriptomic profiles in skin tissues for MCV only.

Pathways represented by genes associated with antibody response to viral antigens were summarized by conducting enrichment analysis based on curated gene sets using the R package clusterProfiler (version 3.12.0) [44]. Significantly associated TWAS genes were grouped by virus family (herpesviruses vs. polyomaviruses) and specificity of association (multiple antigens vs. single antigen).

## Results

A random sample of the participants representative of the full UKB cohort was assayed using a multiplex serology panel [15]. We analyzed data from 7924 participants of predominantly European ancestry, described in (Additional file 2: Table S1). Approximately 90% of individuals were seropositive for herpes family viruses with ubiquitous exposure: EBV (EBV EA-D 86.2% to ZEBRA 91.2%), human herpesvirus 7 (HHV7 94.8%), and varicella zoster virus (VZV 92.3%). Seroprevalence was somewhat lower for cytomegalovirus (CMV), ranging between 56.5% (CMV pp28) and 63.3% (CMV pp52), and herpes simplex virus-1 (HSV1 69.3%). Human polyomavirus BKV was more prevalent (95.3%) compared to other polyomaviruses, Merkel cell polyomavirus (MCV 66.1%) and polyomavirus JC (JCV) (56.6%). Less common infections included HSV-2 (15.2%), HPV16 (E6 and E7 oncoproteins: 4.7%), HPV18 (2.4%), human T-cell lymphotropic virus type 1 (HTLV1, 1.6%), hepatitis B (HBV, 1.6%), and hepatitis C (HCV, 0.3%).

### Genetic determinants of response to viral infection

**Fig. 2:**
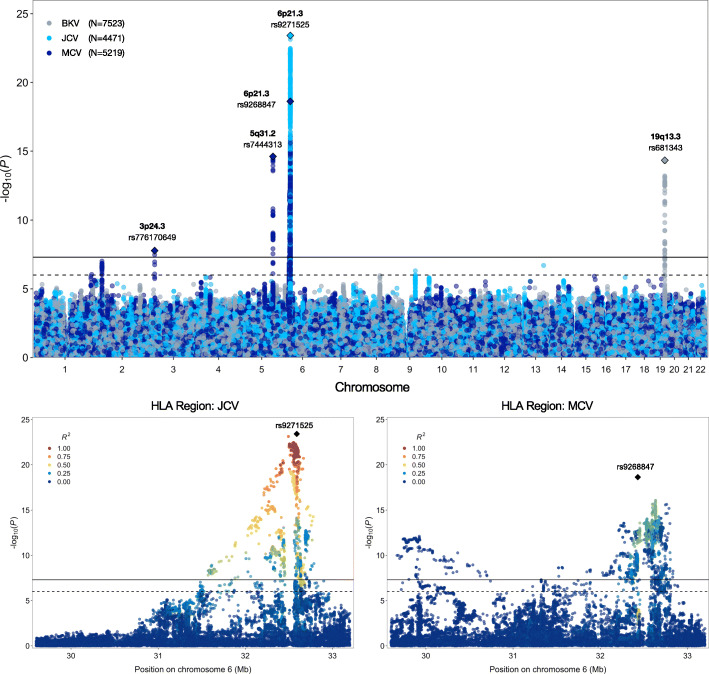
Results from genome-wide and regional association analyses of continuous antibody response phenotypes (MFI *z*-scores) among individuals seropositive for human polyomaviruses BKV, JCV, and Merkel cell (MCV). The lower two panels depict the association signal and linkage disequilibrium (LD) structure in the HLA region for JCV and MCV

Results from our GWAS of antibody response phenotypes were dominated by signals in the HLA region, which were detected for all EBV antigens (EA-D, EBNA, p18, ZEBRA), CMV pp52, HSV1, HHV7, VZV, JCV, and MCV (Table 1; Additional file 1: Figure S3). Most of the top-ranking HLA variants for each antigen were independent of those for other antigens based on *r*^2^ but not *D*′ (Additional file 1: Figure S4). Exceptions were moderate LD between lead variants for EBV ZEBRA and HSV1 (*r*^2^ = 0.45), EBV EBNA and JCV (*r*^2^ = 0.45), and HHV7 and MCV (*r*^2^ = 0.44). However, based on the complex LD structure and effect sizes, we cannot rule out that these linked to rare haplotypes. Outside of the HLA region, genome-wide significant associations with seroreactivity were detected for MCV at 3p24.3 (rs776170649, *LOC339862*: *P* = 1.7 × 10^−8^) and 5q31.2 (rs7444313, *TMEM173* (also known as *STING1*): *P* = 2.4 × 10^−15^); BKV at 19q13.3 (rs681343, *FUT2*: *P* = 4.7 × 10^−15^) (Fig. 2); EBV EBNA at 3q25.1 (rs67886110, *MED12L*: *P* = 1.3 × 10^−9^); HHV-7 at 11q23.3 (rs75438046, *CXCR5*: *P =* 1.3 × 10^−8^) and 17q21.3 (rs1808192, *TBKBP1*: *P* = 9.8 × 10^−9^); and HSV-1 at 10q23.3 (rs11203123: *P* = 3.9 × 10^−8^). However, the loci outside of HLA identified for HHV7 and HSV1 were not statistically significant considering a more stringent significance threshold corrected for the number of seroreactivity phenotypes tested (*P* < 5.0 × 10^−8^/16 = 3.1 × 10^−9^).

**Table 1:** Lead genome-wide significant variants (*P* < 5.0 × 10^−8^) for continuous antibody response phenotypes for antigens with at least 20% seroprevalence

Antigen		*N*	Chr	Position	Variant	Alleles		EAF	Beta^2^	(SE)	P	Function	Nearest gene
						Effect	Other						
CMV	pp52	5000	6	32301427	rs115378818	C	T	0.978	0.633	(0.095)	2.9 × 10^−11^	intronic	*TSBP1*
EBV	EA-D	6806	6	32665840	rs34825357	T	TC	0.409	− 0.114	(0.017)	2.0 × 10^−11^	intergenic	*MTCO3P1*
EBV	EBNA	7003	3	151114852	rs67886110*	G	T	0.596	0.103	(0.017)	1.3 × 10^−9^	intronic	*MED12L*
			6	32451762	rs9269233	A	C	0.249	0.315	(0.019)	3.5 × 10^−61^	intergenic	*HLA-DRB9*
EBV	VCA p18	7492	6	31486158	6:31486158	GT	G	0.245	0.197	(0.018)	7.1 × 10^−27^	intergenic	*PPIAP9*
EBV	ZEBRA	7197	6	32637772	rs9274728	A	G	0.718	− 0.315	(0.018)	4.7 × 10^−67^	intergenic	*HLA-DQB1*
HHV6	IE1A	6077	7	139985625	rs2429218	T	C	0.615	0.106	(0.019)	1.4 × 10^−8^	downstream	*RP5-1136G2.1*
HHV7	U14	7481	6	32602665	rs139299944	C	CT	0.655	0.114	(0.017)	1.5 × 10^−11^	intronic	*HLA-DQA1*
			11	118767564	rs75438046	G	A	0.970	0.280	(0.049)	1.3 × 10^−8^	3′-UTR	*CXCR5* / *BCL9L*
			17	45794706	rs1808192	A	G	0.331	− 0.099	(0.017)	9.8 × 10^−9^	intergenic	*TBKBP1*
HSV1	1gG	5468	6	32627852	rs1130420	G	A	0.583	− 0.122	(0.019)	2.5 × 10^−10^	3′-UTR	*HLA-DQB1*
			10	91189187	rs11203123*	A	C	0.988	0.512	(0.093)	3.9 × 10^−8^	intergenic	*SLC16A12*
VZV	gE/Ig^1^	7289	6	32623193	rs9273325	G	A	0.831	− 0.232	(0.021)	8.2 × 10^−28^	intergenic	*HLA-DQB1*
BKV	VP1	7523	19	49206462	rs681343	C	T	0.491	− 0.125	(0.016)	4.7 × 10^−15^	synonymous	*FUT2*
JCV	VP1	4471	6	32589842	rs9271525	G	A	0.163	− 0.318	(0.031)	3.9 × 10^−24^	intergenic	*HLA-DQA1*
MCV	VP1	5219	3	18238783	rs776170649	CT	C	0.790	− 0.134	(0.024)	1.7 × 10^−8^	intergenic	*LOC339862*
			5	138865423	rs7444313	G	A	0.263	0.169	(0.021)	2.4 × 10^−15^	intergenic	*TMEM173*
			6	32429277	rs9268847	A	G	0.750	− 0.195	(0.022)	2.4 × 10^−19^	intronic	*HLA-DRB9*

^1^VZV antigens gE and gI were co-loaded onto the same Luminex bead set

^2^Regression coefficients were estimated per 1 standard deviation increase in normalized MFI value *z-*scores with adjustment for age at enrollment, sex, body mass index, socioeconomic status (Townsend deprivation index), the presence of any autoimmune conditions, genotyping array, serology assay date, quality control flag, and the top 10 genetic ancestry principal components

*Multi-allelic variants: rs67886110 (G/T and G/C) and rs11203123 (A/C and A/AC)

GWAS of discrete seropositivity phenotypes identified associations in HLA for EBV EA-D (rs2395192: OR = 0.66, *P* = 4.0 × 10^−19^), EBV EBNA (rs9268848: OR = 1.60, *P* = 1.2 × 10^−18^), EBV ZEBRA (rs17211342: 0.63, *P* = 1.6 × 10^−15^), VZV (rs3096688: OR = 0.70, *P* = 3.7 × 10^−8^), JCV (rs9271147: OR = 0.54, *P* = 1.3 × 10^−42^), and MCV (rs17613347: OR = 0.61, *P* = 1.2 × 10^−26^) (Additional file 1: Figure S3; Additional file 2: Table S2). An association with susceptibility to MCV infection was also observed at 5q31.2 (5:138845045_TTATC_T, *ECSCR*: OR = 1.26, *P* = 7.2 × 10^−9^), with high LD (*r*^2^ = 0.95) between seroreactivity and seropositivity lead variants.

Several genome-wide significant associations were observed for antigens with < 20% seroprevalence, which were not included in the GWAS of antibody response due to inadequate sample size (Additional file 2: Table S2). Infection susceptibility variants were identified for HSV2 in 17p13.2 (rs2116443: OR = 1.28, *P* = 4.5 × 10^−8^; *ITGAE*); HPV16 E6 and E7 oncoproteins in 6p21.32 (rs601148: OR = 0.60, *P* = 3.3 × 10^−9^; *HLA-DRB1*) and 19q12 (rs144341759: OR = 0.383, *P* = 4.0 × 10^− 8^; *CTC-448F2.6*); and HPV18 in 14q24.3 (rs4243652: OR = 3.13, *P* = 7.0 × 10^−10^). Associations were also detected for Kaposi’s sarcoma-associated herpesvirus (KSHV), HTLV1, HBV, and HCV, including a variant in the *MERTK* oncogene (HCV Core rs199913364: OR = 0.25, *P* = 1.2 × 10^−8^). After correcting for 28 serostatus phenotypes tested (*P* < 1.8 × 10^−9^), the only statistically significant associations remained for EBV EA-D (rs2395192), EBV EBNA (rs9268848), EBV ZEBRA (rs17211342), JCV (rs9271147), MCV (rs17613347), and HPV18 (rs4243652).

### Functional characterization of GWAS findings

In silico functional analyses of the lead 17 GWAS variants identified enrichment for multiple regulatory elements (summarized in Additional file 2: Table S3). Three variants were predicted to be in the top 10% of deleterious substitutions in GRCh37 based on CADD scores > 10: rs776170649 (MCV, CADD = 15.61), rs139299944 (HHV7, CADD = 12.15), and rs9271525 (JCV, CADD = 10.73). Another HHV7-associated variant, rs1808192 (RegulomeDB rank: 1f), an eQTL and sQTL for *TBKBP1*, mapped to 44 functional elements for multiple transcription factors, including IKZF1, a critical regulator of lymphoid differentiation frequently mutated in B cell malignancies.

Eleven sentinel variants were eQTLs and 8 were splicing QTLs in GTEx, with significant (FDR < 0.05) effects across multiple genes and tissues (Additional file 1: Figure S5). The most common eQTL and sQTL targets included *HLA-DQA1*, *HLA-DQA2*, *HLA-DQB1*, *HLA-DQB2*, *HLA-DRB1*, and *HLA-DRB6*. Outside of HLA, rs681343 (BKV), a synonymous *FUT2* variant was an eQTL for 8 genes, including *FUT2* and *NTN5*. MCV variant in 5q31.2, rs7444313, was an eQTL for 7 genes, with concurrent sQTL effects on *TMEM173*, also known as *STING1* (stimulator of interferon response cGAMP interactor 1) and *CXXC5*. Gene expression profiles in immune cell populations from DICE [24] identified several cell-type-specific effects that were not observed in GTEx. An association with *HLA-DQB1* expression in CD4+ T_H_2 cells was observed for rs9273325, 6:31486158_GT_G was an eQTL for *ATP6V1G2* in naïve CD4+ T cells, and rs1130420 influenced the expression of 8 HLA class II genes in naïve B cells and CD4+ T_H_17 cells.

We identified 7 significant (*p* < 5.0 × 10^−8^) protein quantitative trait loci (pQTL) for 38 proteins (Additional file 2: Table S4). Most of the pQTL targets were components of the adaptive immune response, such as the complement system (C4, CFB), chemokines (CCL15, CCL25), and defensin processing (Beta-defensin 19, Trypsin-3). The greatest number and diversity of pQTL targets (*n* = 16) was observed for rs681343, including BPIFB1, which plays a role in antimicrobial response in oral and nasal mucosa [45]; FUT3, which catalyzes the last step of Lewis antigen biosynthesis; and FGF19, part of the PI3K/Akt/MAPK signaling cascade that is dysregulated in cancer and neurodegenerative diseases [46].

### Cross-trait associations with disease outcomes

To contextualize the relevance of genetic loci involved in infection response, we explored associations with selected cancers, schizophrenia, and that have a known or suspected viral etiology (Additional file 2: Table S5). The strongest secondary signal was observed for rs9273325 (*HLA-DQB1*), which was negatively associated with VZV antibody response and positively associated with schizophrenia susceptibility (OR = 1.13, *P* = 4.3 × 10^−15^). Other significant (Bonferroni *P* < 7.4 × 10^−4^) associations with schizophrenia were detected for HSV1 (rs1130420: OR = 1.06, *P* = 1.8 × 10^−5^), EBV EA-D (rs2647006: OR = 0.96, *P* = 2.7 × 10^−4^), JCV (rs9271525: OR = 1.06, *P =* 6.8 × 10^−5^), and BKV (rs681343: OR = 0.96, *P* = 2.5 × 10^−4^), with the latter being the only pleiotropic signal outside of HLA. Inverse associations with hematologic cancers were observed for HSV1 (rs1130420: OR = 0.89, *P* = 3.5 × 10^−6^), VZV (rs9273325: OR = 0.88, *P* = 4.4 × 10^−5^), and EBV EBNA (rs9269233: OR = 0.88, *P* = 2.7 × 10^−4^) variants. HSV1 antibody response was also linked to Alzheimer’s disease (rs1130420: *P* = 1.2 × 10^−4^).

### Regional HLA associations

Associations within the HLA region were refined by identifying independent (LD *r*^2^ < 0.05 within ± 500 kb) index variants with *P* < 5.0 × 10^−8^ for each antigen response phenotype (Additional file 2: Table S6). Clumping seropositivity associations with respect to lead antibody response variants did not retain any loci, suggesting non-independence in signals for infection and reactivity for the same antigen. This was also confirmed based on genetic correlations (*r*_g_) estimated using LD score regression [47], which ranged from *r*_g_ = 0.407 (*p* = 5.1 × 10^−3^) for VZV to *r*_g_ = 0.896 (*p* = 3.1 × 10^−7^) for MCV. For this reason, all subsequent analyses focus on seroreactivity phenotypes. Clumping across phenotypes to assess the independence of HLA associations for different antigens identified 40 independent index variants: EBV EBNA (12), VZV (11), EBV ZEBRA (8), EBV p18 (5), MCV (3), and EBV EA-D (1) (Additional file 2: Table S7). No LD clumps were anchored by variants detected for CMV pp52, HHV7, HSV1, or JCV, suggesting that the HLA signals for these antigens are captured by lead loci for other phenotypes. The largest region with the lowest *p* value was anchored by rs9274728 (*P* = 4.7 × 10^−67^) near *HLA-DQB1*, originally detected for EBV ZEBRA. Of the 11 VZV-associated variants, the largest clump was formed around rs4990036 (*P* = 4.5 × 10^−26^) in *HLA-B*.

**Fig. 3:**
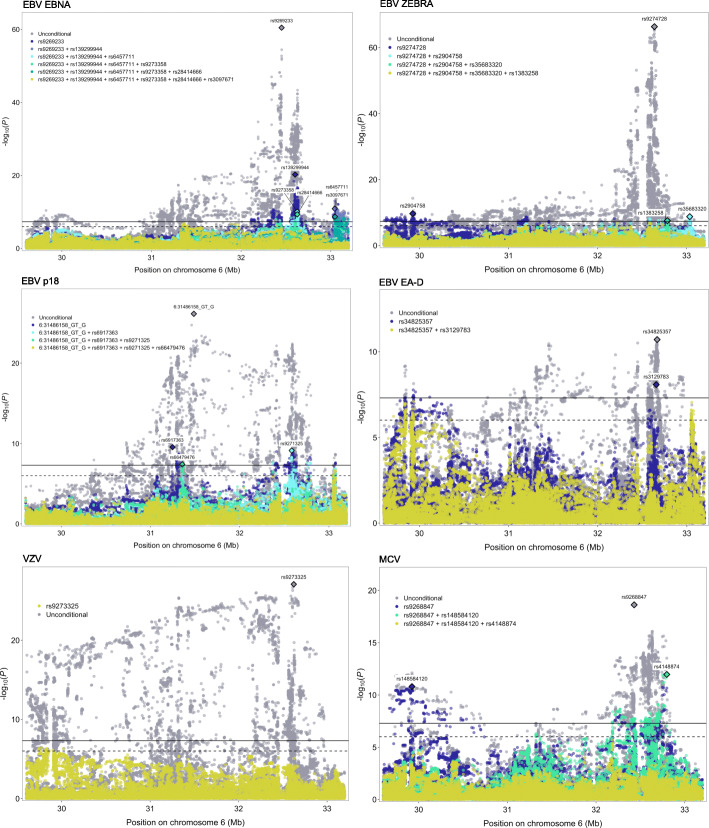
Regional association plots for conditionally independent HLA genetic variants that were significantly (*P* < 5.0 × 10^−8^, solid black line) associated with each continuous antibody response phenotype. The suggestive significance threshold corresponds to *P* < 1.0 × 10^−6^ (dotted black line)

Iterative conditional analyses adjusting for the HLA SNP/indel with the lowest *p* value were performed until no variants remained with *P*_cond_ < 5.0 × 10^−8^. Additional independent variants were identified for EBV EBNA (rs139299944, rs6457711, rs9273358, rs28414666, rs3097671), EBV ZEBRA (rs2904758, rs35683320, rs1383258), EBV p18 (rs6917363, rs9271325, rs66479476), and MCV (rs148584120, rs4148874) (Fig. 3; Additional file 2: Table S8). For CMV pp52, HHV7, HSV1, JCV, and VZV, the regional HLA signal was captured by the top GWAS variant (Fig. 2; Additional file 2: Table S8).

Next, we tested 101 classical HLA alleles and performed analogous iterative conditional analyses for significantly associated variants (*P* < 4.6 × 10^−5^). To help with the interpretation of our results, we depict the LD structure for HLA alleles in class II genes in Additional file 1: Figure S6. Significant associations across viruses were predominantly observed for class II HLA alleles. Five statistically independent signals were identified for antibody response to EBV ZEBRA (DRB4*00:00: *β* = − 0.246, *P* = 1.4 × 10^−46^; DQB1*04:02: *β*_cond_ = 0.504, *P*_cond_ = 1.0 × 10^−19^; DRB1*04:04: *β*_cond_ = 0.376, *P*_cond_ = 1.1 × 10^−18^; DQA1*02:01: *β*_cond_ = 0.187, *P*_cond_ = 1.1 × 10^−10^; A*03:01: *β*_cond_ = 0.129, *P*_cond_ = 1.9 × 10^−8^) (Fig. 3; Additional file 2: Table S9). DRB4*00:00 represents copy number absence, which co-occurs with DRB1*04 and DRB1*07 alleles [48]. This is consistent with the magnitude and direction of unconditional associations observed for DRB1*07:01 (*β* = 0.251, *P* = 1.3 × 10^−26^) and DRB4*04:01 (*β* = 0.293, *P* = 7.9 × 10^−22^). Five conditionally independent alleles were also identified for EBV EBNA: DRB5*00:00: *β* = − 0.246, *P* = 8.7 × 10^−30^; DRB3*02:02: *β*_cond_ = 0.276, *P*_cond_ = 6.8 × 10^−30^; DQB1*02:01: *β*_cond_ = − 0.164, *P*_cond_ = 3.6 × 10^−12^; DRB4*00:00: *β* = 0.176, *P*_cond_ = 8.3 × 10^−17^; and DPB1*03:01: *β*_cond_ = − 0.220, *P*_cond_ = 4.7 × 10^−14^ (Fig. 3; Additional file 2: Table S10). DRB5*00:00 denotes a copy number absence that sits on a common haplotype comprised of DRB1*15:01, DQB1*06:02, DQA1*01:02 [48], which may also include DRB5*01:01 [49] (Additional file 1: Figure S6). The presence of the DRB1*15:01-DQB1*06:02-DQA1*01:02 haplotype was associated with increased EBV EBNA seroreactivity (*β* = 0.330, *P* = 2.5 × 10^−28^). Fewer independent alleles were observed for EBV p18 (DRB5*00:00: *β* = − 0.210, *P* = 1.7 × 10^−22^; DRB1*04:04: *β*_cond_ = 0.357, *P*_cond_ = 1.3 × 10^−18^) (Fig. 3; Additional file 2: Tables S11).

DQB1*02:01 was the only independently associated allele for EBV EA-D (*β* = − 0.154, *P* = 8.4 × 10^−11^) and HSV1 (*β* = 0.145, *P* = 2.8 × 10^−8^), although its effects were in opposite directions for each antigen (Additional file 2: Table S12). For VZV, associations with 16 classical alleles were accounted for by DRB1*03:01 (β = 0.236, *P* = 7.3 × 10^−26^). JCV shared the same lead allele as EBV EBNA and EBV p18 (DRB5*00:00: *β* = 0.350, *P* = 1.2 × 10^−21^) (Additional file 2: Table S12). Four conditionally independent signals were identified for MCV (DQA1*01:01: *β* = 0.215, *P* = 1.1 × 10^−15^; DRB1*04:04: *β*_cond_ = − 0.362, *P*_cond_ = 3.0 × 10^−11^; A*29:02: *β*_cond_ = − 0.350, *P* = 1.0 × 10^−11^; DRB1*15:01: *β*_cond_ = − 0.203, *P* = 3.7 × 10^−12^) (Fig. 3; Additional file 2: Table S13). Lastly, we integrated associations across variant types by including conditionally independent HLA alleles as covariates in the SNP-based analysis. With the exception of EBV antigens and HHV7, classical HLA alleles captured all genome-wide significant SNP signals (Additional file 1: Figure S7).

Finally, we tested 980 HLA amino acid substitutions (Additional file 2: Tables S14-S23), followed by omnibus haplotype tests at each position that had a significant amino acid and more than two possible alleles. The strongest allele-specific and haplotype associations were found at different positions in the same protein for EBV p18 (DRβ1 Ala -17: *β* = − 0.194, *P* = 1.0 × 10^−21^; DRβ1 (13): *P*_omni_ = 4.6 × 10^−22^; Additional file 2: Table S14), MCV (DQβ1 Leu-26: *β* = − 0.173, *P* = 7.0 × 10^−18^; DQβ1 (125): *P*_omni_ = 2.0 × 10^−17^; Additional file 2: Table S15), HHV7 (DQβ1 His-30: *β* = − 0.111, *P* = 1.2 × 10^−8^; DQβ1 (57): *P*_omni_ = 5.6 × 10^−9^; Additional file 2: Table S16), and HHV6 IE1B at (DRβ1 Ile-67: *β* = 0.131, *P* = 1.6 × 10^−8^; DRβ1 (13): *P*_omni_ = 1.1 × 10^−5^; Additional file 2: Table S17).

The strongest residue-specific and haplotype associations mapped to the same amino acid position for four phenotypes: EBV ZEBRA (Additional file 2: Table S18), HHV6 IE1A (Additional file 2: Table S17), HSV1 (Additional file 2: Table S19), and JCV (Additional file 2: Table S20). Amino acid residues at DQα1 (175) were associated with antibody response to EBV ZEBRA (Glu: *β* = 0.279, *P* = 1.1 × 10^−61^; *P*_omni_ = 8.3 × 10^−62^). Glu-175 is present in DQA1*02:01 (*P* = 4.9 × 10^−27^), DQA1*03:01 (*P* = 1.3 × 10^−16^), and DQA1*04:01 (*P* = 1.9 × 10^−12^) and seems to better summarize the EBV ZEBRA signal at this locus. Substitutions in DRβ1 (96) contained the strongest predictors of JCV seroreactivity (His or Tyr: *β* = 0.325, *P* = 1.6 × 10^−25^; *P*_omni_ = 7.7 × 10^−23^). His-96/Tyr-96 are in high LD (*r*^2^ = 0.92) with DRB5*00:00, the top JCV-associated allele. However, this might mask the signal for Gln-96 (*β* = − 0.310, *P* = 9.0 × 10^−23^), which is part of the DRB1*15:01 sequence (*β* = − 0.309, *P* = 9.0 × 10^−21^; LD *r*^2^ = 0.94). The lead signal for HSV1 mapped to DQβ1 (57) (Ala: *β* = 0.123, *P* = 2.2 × 10^−10^; *P*_omni_ = 6.5 × 10^−9^), which aligns with the association for the lead HSV1-allele DQB1*02:01.

For EBV EBNA, the strongest haplotype association was in DRβ1 (37) (*P*_omni_ = 1.1 × 10^−55^), while the residue with the lowest *p* value was DQβ1 Ala-57 (*β* = − 0.237, *P* = 1.4 × 10^−42^) (Additional file 2: Table S21). Ala-57 maps to multiple DQB1 alleles and achieved a stronger signal for EBV EBNA than any classical HLA allele. Asp-9 in HLA-B showed the strongest association with antibody response to EBV EA-D (*β* = − 0.146, *P* = 1.8 × 10^−9^; Additional file 2: Table S22) and VZV (*β* = 0.237, *P* = 9.7 × 10^−25^; Additional file 2: Table S23). This amino acid sequence is part of B*08:01, which had analogous effects on both phenotypes (EBV EA-D: *β* = − 0.144, *P* = 2.7 × 10^−9^; VZV: *β* = 0.238, *P* = 4.7 × 10^−25^). Haplotypes with the lowest overall *p* values were found in DQβ1 (71) for VZV (*P*_omni_ = 9.8 × 10^−19^) and DRβ1 (11) for EBV EA-D (*P*_omni_ = 1.7 × 10^−10^).

### TWAS of genes involved in antibody response

**Fig. 4:**
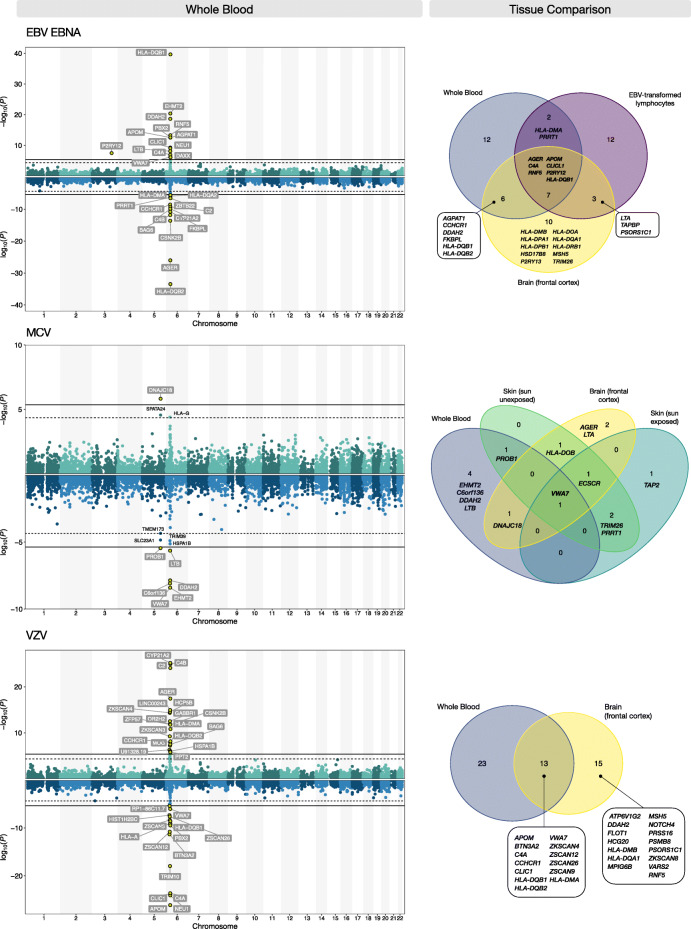
Transcriptome-wide association study (TWAS) results for continuous antigen response phenotypes. Two Manhattan plots depicting the associations for genes with a positive direction of effect (increased expression leads to higher antibody response) and genes with a negative direction of effect (increased expression is associated with a reduced antibody response). The threshold for statistical significance was determined based on the Bonferroni correction for the number of genes tested (*P* < 4.2 × 10^−6^, solid black line), while the suggestive significance threshold was set at *P* < 4.2 × 10^−5^ (dotted black line)

Based on known targets of infection or related pathologies, we considered expression in the frontal cortex (Additional file 2: Table S24), EBV-transformed lymphocytes for EBV antigens (Additional file 2: Table S25), and skin for MCV (Additional file 2: Table S26). Concordance across tissues was summarized using Venn diagrams (Fig. 4; Additional file 1: Figure S8). TWAS identified 114 genes significantly associated (*P*_TWAS_ < 4.2 × 10^−6^) with antibody response in at least one tissue, 54 of which were associated with a single phenotype, while 60 influenced seroreactivity to multiple antigens. We also include results for 87 additional suggestively (*P*_TWAS_ < 4.2 × 10^−5^) associated genes.

The TWAS results included a predominance of associations in HLA class II genes. Some of the strongest overall associations were observed for *HLA-DRB5* (EBV ZEBRA: *P*_cortex_ = 4.2 × 10^−45^) and *HLA-DRB1* (EBV EBNA: *P*_cortex_ = 6.7 × 10^−39^; EBV ZEBRA: *P*_cortex_ = 3.3 × 10^−33^; JCV: *P*_cortex_ = 6.5 × 10^−14^; EBV p18: *P*_cortex_ = 2.2 × 10^−12^). Increased expression of *HLA-DQB2* was positively associated with antibody response to EBV ZEBRA (*P*_blood_ = 7.6 × 10^−19^), JCV (*P*_blood_ = 9.9 × 10^−10^), VZV (*P*_blood_ = 7.0 × 10^−9^), HHV7 (*P*_blood_ = 7.3 × 10^−8^), and HSV1 (*P*_blood_ = 3.3 × 10^−7^), but negatively associated with EBV EBNA (*P*_blood_ = 3.6 × 10^−34^) and EBV p18 (*P*_blood_ = 2.1 × 10^−8^), in a consistent manner across tissues. The opposite was observed for *HLA-DQB1*, with positive effects on EBV EBNA and EBV p18 and inverse associations with EBV ZEBRA, JCV, VZV, HHV7, and HSV1.

The TWAS analyses also identified a number of significant associations in the HLA class III region that were not detected in other analyses. The top-ranking VZV associated gene was *APOM* (*P*_blood_ = 7.5 × 10^−27^, *P*_cortex_ = 1.1 × 10^−25^). Interestingly, opposite directions of effect were observed for *C4A* and *C4B* gene expression. Increased *C4A* expression was positively associated with all EBV antigens (Additional file 2: Table S25), but negatively associated with VZV (*P*_blood_ = 2.3 × 10^−24^) and HSV1 (*P*_cortex_ = 1.8 × 10^−5^) antibody levels (Additional file 2: Table S24). On the other hand, increased *C4B* expression was inversely associated with EBV phenotypes, but positively associated with VZV (*P*_blood_ = 8.1 × 10^−25^) and HSV1 (*P*_blood_ = 1.1 × 10^−5^). A similar pattern was also observed for *CYP21A2* and *C2*, with positive effects on antibody response to VZV and HSV1, and negative effects for all EBV antigens. Other novel TWAS findings were detected for HHV7 in 22q13.2 (*CTA-223H9.9*: *P*_TWAS_ = 2.5 × 10^−6^; *CSDC2*: *P*_TWAS_ = 3.0 × 10^−6^; *TEF*: *P*_TWAS_ = 3.1 × 10^−6^) and 1q31.2 (*RGS1*: *P*_TWAS_ = 3.3 × 10^−6^).

**Fig. 5:**
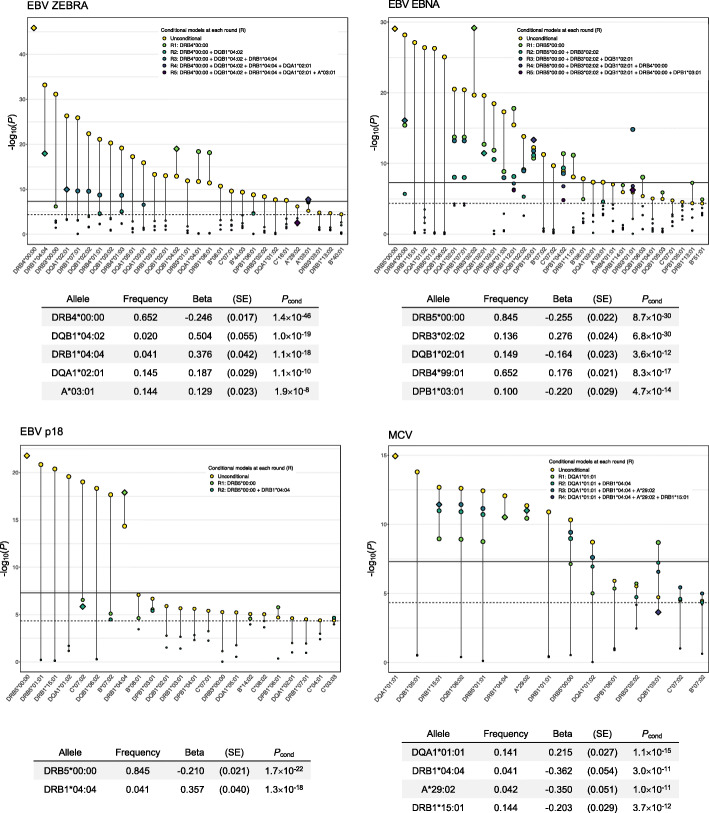
Conditionally independent classical HLA alleles significantly (*P*_cond_ < 5.0 × 10^−8^, solid line) associated with each continuous antibody response phenotype. Only classical alleles that surpassed the Bonferroni-corrected significance threshold (*P* < 4.6 × 10^−5^, dotted line) were included in conditional analyses

The TWAS recapitulated several GWAS-identified loci: 3q25.1 for EBV EBNA (*P2RY13*: *P*_cortex_ = 1.1 × 10^−8^; *P2RY12*: *P*_blood_ = 3.3 × 10^−8^) and 19q13.33 for BKV (*FUT2*: *P*_TWAS_ = 8.1 × 10^−13^; *NTN5*: *P*_TWAS_ = 1.1 × 10^−9^). Transcriptomic profiles in skin tissues provided supporting evidence for the role of multiple genes in 5q31.2 in modulating MCV antibody response (Fig. 5; Additional file 2: Table S26). The strongest signal was observed in for *ECSCR* (skin sun unexposed: *P*_TWAS_ = 5.0 × 10^−15^; skin sun exposed: *P*_TWAS_ = 4.2 × 10^−13^), followed by *PROB1* (sun unexposed: *P*_TWAS_ = 1.5 × 10^−11^). *ECSCR* expression was also associated based on expression in the frontal cortex, while *PROB1* exhibited a significant, but attenuated effect in whole blood. *VWA7* was the only gene associated across all four tissues for MCV and was also associated with antibody response to several EBV antigens.

Comparison of results for seroreactivity and seropositivity revealed a number of genes implicated in both steps of the infection process (Additional file 2: Table S27). Associations with HLA DQA and DQB genes in whole blood and HLA-DRB genes in the frontal cortex were observed for EBV antigens, JCV, and MCV. For MCV, the strongest seropositivity signals were observed for HLA class III genes *AGER* (*P*_cortex_ = 9.0 × 10^−21^) and *EHMT2* (*P*_blood_ = 5.8 × 10^−18^), which were also among the top-ranking genes for seroreactivity. Increased *ECSCR* expression conferred an increased susceptibility to MCV infection (*P*_cortex_ = 1.8 × 10^−8^), mirroring its effect on seroreactivity. In contrast to antibody response, no significant associations with any HLA genes were observed for VZV seropositivity.

Analyses using the Reactome database identified significant (*q*_FDR_ < 0.05) enrichment for TWAS-identified genes in pathways involved in initiating antiviral responses, such as MHC class II antigen presentation, TCR signaling, and interferon (IFN) signaling (Additional file 1: Figure S9). Pathways unique to herpesviruses included folding, assembly, and peptide loading of class I MHC (*q* = 3.2 × 10^−7^) and initial triggering of complement (*q* = 9.8 × 10^−3^). Polyomaviruses were associated with the non-canonical nuclear factor (NF)-κΒ pathway activated by tumor necrosis factor (TNF) superfamily (*q* = 1.9 × 10^−3^).

## Discussion

We performed genome-wide and transcriptome-wide association studies for serological phenotypes for 16 common viruses in a well-characterized, population-based cohort. We discovered novel genetic determinants of viral antibody response beyond the HLA region for BKV, MCV, HHV7, and EBV EBNA. Consistent with previous studies [7, 8], we detected strong signals for immune response to diverse viral antigens in the HLA region, with a predominance of associations observed for alleles and amino acids in *HLA-DRB1* and *HLA-DQB1*, as well as transcriptome-level associations for multiple class II and III HLA genes. Taken together, the findings of this work provide a resource for further understanding the complex interplay between viruses and the human genome, as well as a first step towards understanding genetic determinants of reactivity to common infections.

One of our main findings is the discovery of 5q31.2 as a susceptibility locus for MCV infection and MCV antibody response, implicating two main genes: *TMEM173* (or *STING1*) and *ECSCR*. The former encodes STING (stimulator of interferon genes), an endoplasmic reticulum (ER) protein that controls the transcription of host defense genes and plays a critical role in response to DNA and RNA viruses [50]. STING is activated by cyclic GMP-AMP synthase (cGAS), a cytosolic DNA sensor that mounts a response to invading pathogens by inducing IFN1 and NF-κΒ signaling [51, 52]. Polyomaviruses penetrate the ER membrane during cell entry, a process that may be unique to this viral family [53], which may trigger STING signaling in a distinct manner from other viruses [53]. Multiple cancer-causing viruses, such as KSHV, HBV, and HPV18, encode oncoproteins that disrupt cGAS-STING activity, which illustrates the evolutionary pressure on DNA tumor viruses to develop functions against this pathway and its importance in carcinogenesis [51]. Furthermore, cGAS-STING activation has been shown to trigger antitumor T cell responses, a mechanism that can be leveraged by targeted immunotherapies [54–56]. Several studies suggest STING agonists may be effective against tumors resistant to PD-1 blockade, as well as promising adjuvants in cancer vaccines [57–59].

*ECSCR* expression in skin and brain tissues was associated with MCV antibody response and infection. This gene encodes an endothelial cell-specific chemotaxis regulator, which plays a role in angiogenesis and apoptosis [60]. *ECSCR* is a negative regulator of PI3K/Akt signaling by enhancing membrane localization of *PTEN* and operates in tandem with VEGFR-2 and other receptor tyrosine kinases [61]. In addition to 5q31.2, another novel MCV seroreactivity associated region was identified in 3p24.3, anchored by rs776170649, which has been linked to platelet phenotypes [62]. These findings align with a role of platelet activation in defense against infections via degranulation-mediated release of chemokines and β-defensin [63].

Genetic variation within Fucosyltransferase 2 (*FUT2*) has been studied extensively in the context of human infections; however, its effect on BKV seroreactivity is novel. Homozygotes for the nonsense mutation (rs601338 G>A) that inactivates the FUT2 enzyme are unable to secrete ABO(H) histo-blood group antigens or express them on mucosal surfaces [64, 65]. The allele which confers increased BKV antibody response (rs681343-T) is in LD (*r*^2^ = 1.00) with rs601338-A, the non-secretor allele, which confers resistance to norovirus [66, 67], rotavirus [68], *Helicobacter pylori* [69], childhood ear infection, mumps, and common colds [13]. However, increased susceptibility to other pathogens, such as meningococcus and pneumococcus [70], has also been observed in non-secretors. Isolating the underlying mechanisms for BKV response is challenging because *FUT2* is a pleiotropic locus associated with diverse phenotypes, including autoimmune and inflammatory conditions [71, 72], serum lipids [73], B vitamins [65, 74], alcohol consumption [75], and even certain cancers [76]. In addition to *FUT2* in 19q13.33, *NTN5* (netrin 5) suggests a possible link between BKV and neurological conditions. NTN5 is primarily expressed in neuroproliferative areas, suggesting a role in adult neurogenesis, which is dysregulated in glioblastoma and Alzheimer’s disease [77, 78].

We also report the first GWAS of serological phenotypes for HHV7. Genetic determinants of HHV7 antibody response in 6p21.32 were predominantly localized in *HLA-DQA1* and *HLA-DQB1*, with associations similar to other herpesviruses. In 11q23.3, rs75438046 maps to the 3′ UTR of *CXCR5*, which controls viral infection in B cell follicles [79], and *BCL9L*, a translocation target in acute lymphoblastic leukemia [80] and transcriptional activator of the Wnt/β-catenin cancer signaling pathway [81]. In 17q21.32, *TBKBP1* encodes an adaptor protein that binds to TBK1 and is part of the TNF/NF-κΒ interaction network, where it regulates immune responses to infectious triggers, such as IFN1 signaling [82]. Interestingly, a protein interactome map recently revealed that SARS-CoV-2 nonstructural protein 13 (Nsp13) includes TBK1-TBKBP1 among its targets [83]. Other functions of the TBK1-TBKBP1 axis relate to tumor growth and immunosuppression through induction of PD-L1 [84].

Several additional genes involved in HHV7 immune response were identified in TWAS. *TEF* in 22q13.2 is an apoptotic regulator of hematopoietic progenitors with tumor promoting effects mediated by inhibition of G1/S cell cycle transition and Akt/FOXO signaling [85]. *RGS1* in 1q31.2 has been linked to multiple autoimmune diseases, including multiple sclerosis [86], as well as poor prognosis in melanoma and diffuse large B cell lymphoma mediated by inactivation of Akt/ERK [87, 88].

Other genes outside of the HLA region associated with viral infection response were detected for EBV EBNA in 3q25.1. The lead variant (rs67886110) is an eQTL for *MED12L* and *P2RY12* genes, which have been linked to neurodegenerative conditions [89, 90]. *P2RY12* and *P2RY13*, identified in TWAS, are purinergic receptor genes that regulate microglia homeostasis and have been implicated in Alzheimer’s susceptibility via inflammatory and neurotrophic mechanisms [90].

Considering genetic variation within the HLA region, our results confirm its pivotal role at the interface of host pathogen interactions and highlight the extensive sharing of HLA variants that mediate these interactions across virus families and antigens. Genes in this region code for cell-surface proteins that facilitate antigenic peptide presentation to immune cells that regulate responses to invading pathogens. This region is critical for adaptive immune response but also has significant overlap with susceptibility alleles for autoimmune diseases. We identified 40 independent SNPs/indels associated with EBV (EBNA, EA-D, VCA p18, and ZEBRA), VZV, and MCV antibody response that accounted for all significant HLA associations for other phenotypes. However, compared to conditional analyses, clumping may overestimate the number of independent variants due to the complex long-range LD structure in HLA and violation of the assumption that 500-kb windows are sufficient to exclude correlated variants in this region. Of the 14 conditionally independent, genome-wide significant classical alleles identified for 10 antigens, 7 were associated with multiple phenotypes. The most commonly shared HLA alleles were DRB5*00:00, DRB1*04:04, an known rheumatoid arthritis risk allele [91], and DQB1*02:01, associated with celiac disease risk [92]. Although allele absence represented by DRB5*00:00 may have a functional role in altering antigen response, a more likely explanation for the observed signal is that DRB5*00:00 acts as a biomarker for a specific class II haplotype. Rather than having a direct causal effect on antigen presentation, this “null allele” summarizes signals from multiple HLA loci, including the extended DRB5*01:01-DRB1*15:01-DQB1*06:02-DQA1*01:02 haplotype that has been implicated in the etiology of multiple autoimmune diseases and EBV EBNA IgG levels. DRB1*15:01-DQB1*06:02-DQA1*01:02 is protective for type 1 diabetes [93], while DRB5*01:01-DRB15:01 confers the strongest risk for developing multiple sclerosis [86]. Amino acid residues in DRβ1 at positions 11, 13, 71, and 74 and in DQβ1 codon 57 represent established susceptibility loci for rheumatoid arthritis [94], type 1 diabetes [95], and multiple sclerosis [96] that exhibited strong associations with IgG levels for EBV, HHV7, VZV, JCV, and MCV antigens, and in some cases harbored the top signal of all HLA variants. Further research is needed to delineate shared genetic pathways that invoke autoimmunity and influence viral response.

Despite the predominance of association in HLA class II, several notable associations in HLA class I were detected. A*29:02 conferred reduced MCV seroreactivity and its sequence overlaps with amino acid residues in the A α1 domain (Thr-9, Leu-62, Gln-63, Asn-77, and Met-97) that were also significantly associated with decreased MCV antibody response. This is consistent with downregulation of MHC I as a potential mechanism through which Merkel cell tumors evade immune surveillance [97]. The strongest residue-specific signal for EBV EA-D and VZV mapped to B-Asp-9, which is located in the peptide binding groove and tags the B*08:01 allele, part of the HLA 8.1 ancestral haplotype. There is extensive evidence linking HLA 8.1, and B*08:01 specifically, with autoimmune diseases [98] and certain cancers [99, 100], which may be attributed to its high cell-surface stability and increased probability of CD8+ T cell activation.

Comparison with other studies of host genetics and viral infection susceptibility shows that our results align with previously reported findings [7–9, 101] (Additional file 2: Table S28). We replicated most associations from two of the largest GWAS of humoral immune response in European ancestry subjects by Hammer et al. [7] (*n* = 2363) and Scepanovic et al. [8] (*n* = 1000), including HLA SNPs, alleles, amino acids, and haplotypes linked to EBV EBNA IgG, MCV IgG and serostatus, and JCV serostatus. We also replicated two *HLA-DRB1* variants (rs477515, rs2854275) associated with EBV EBNA antibody levels in a Mexican American population [9]. GWAS of HPV16 L1 replicated a variant previously linked to HPV8 seropositivity (rs9357152, *P* = 0.008, 6). Some of our findings contrast with Tian et al. [13], although we confirmed selected associations, such as A*02:01 (shingles) with VZV (*P* = 4.1 × 10^−8^) and rs2596465 (mononucleosis) with EBV EBNA (*P* = 3.3 × 10^−9^) and EBV p18 (*P* = 1.0 × 10^−12^). These differences may be partly accounted for by self-reported disease status in Tian et al. which is likely to reflect symptom severity and may be an imprecise indicator of infection with certain viruses or the magnitude of antibody response to infection.

One of the most striking findings in SNP-based HLA analyses was the genome-wide significant association between rs9273325, index VZV antibody response variant, and risk of schizophrenia. Previous epidemiologic and serologic studies have linked infections to schizophrenia, although the underlying mechanisms remain to be elucidated [102]. Viruses are plausible etiologic candidates for schizophrenia due to their ability to invade the central nervous system and disrupt neurodevelopmental processes by targeting specific neurons, as well as the potential for latent infection to negatively impact plasticity and neurogenesis via pro-inflammatory and aberrant immune signaling [102, 103]. These observations are consistent with the established role the HLA region, including *HLA-DQB1*, in schizophrenia etiology [104, 105], and is further supported by previously reported associations for rs9273325 with blood cell traits [62] and immunoglobulin A deficiency [106], as well as its role as an eQTL for *HLA-DQB1* in CD4+ T_2_h cells. Schizophrenia susceptibility alleles DRB1*03:01 [104], DQB1*02:01, and B*08:01 were also the top three alleles associated with VZV antibody response in the unconditional analysis. Enhanced complement activity has been proposed as the mechanism mediating the synaptic loss and excessive pruning which is a hallmark of schizophrenia pathophysiology [107]. Complement component 4 (C4) alleles were found to increase risk of schizophrenia proportionally to their effect on increasing *C4A* expression in brain tissue [107]. Using gene expression models in whole blood and the frontal cortex we demonstrated that increased *C4A* expression is negatively associated with VZV antibody response. We also observed associations with *C4A* and *C4B* in EBV and HSV-1, but not other viruses. Taken together, these findings delineate a potential mechanism through which aberrant immune response to VZV infection, and potentially HSV-1 and EBV, may increase susceptibility to schizophrenia. However, cautious interpretation is warranted due to significant pleiotropy between HLA loci associated with viral infection and broad immune function.

Several limitations of this work should be noted. First, the UK Biobank is unrepresentative of the general UK population due to low participation resulting in healthy volunteer bias [108]. However, since the observed pattern of seroprevalence is consistent with previously published estimates [15], we believe the impact of this bias is likely to be minimal on genetic associations with serological phenotypes. Second, our analyses were restricted to participants of European ancestry due to limited serology data for other ancestries, which limits the generalizability of our findings to diverse populations. Third, we were unable to conduct formal statistical replication of novel GWAS and TWAS signals in an independent sample due to the lack of such a population. Nevertheless, our successful replication of multiple previously reported variants and, combined with the observation that newly discovered genes and variants are part of essential adaptive and innate immunity pathways, support the credibility of our findings. Lastly, we also stress caution in the interpretation of GWAS results for non-ubiquitous pathogens, such as HBV, HCV, and HPV, due to a lack of information on exposure, as well as low numbers of seropositive individuals.

Our study also has distinct advantages. The large sample size of the UK Biobank facilitated more powerful genetic association analyses than previous studies, particularly in a population-based cohort unselected for disease status. Our detailed HLA analysis shows independent effects of specific HLA alleles and pleiotropic effects across multiple viruses. Analyses of genetic associations in external datasets further demonstrate a connection between host genetic factors influencing immune response to infection and susceptibility to cancers and neurological conditions.

The results of this work highlight widespread genetic pleiotropy between pathways involved in regulating humoral immune response to novel and common viruses, as well as complex diseases. The complex evolutionary relationship between viruses and humans is not dictated simply by infection and acute sickness, it is a complex nuanced architecture of initial challenge tempered with tolerance of viral latency over time. Yet it is that architecture that is evolutionarily optimized to maximize fitness early in life, the result of which may be increased risk for complex diseases later in life. Understanding this complex interplay through both targeted association studies and functional investigations between host genetic factors and immune response has implications for complex disease etiology and may facilitate the discovery of novel therapeutics in a wide range of diseases.

## Conclusions

We present a genome-wide investigation host genetic factors influencing antibody response to common viral antigens. Our study confirms the importance of HLA class II genes in modulating IgG levels for human herpes and polyomaviruses and illustrates the complexity of genetic effects in this region represented by single variants, classical alleles, and amino acid substitutions. We also uncovered novel genetic loci beyond HLA that contribute to host-virus interaction, including signals in 3q25.1, 5q31.2, and 19q13.33, which may operate via direct effects on gene expression. Taken together, the findings presented here provide a resource of genetic determinants of immune response to common viruses, which may be leveraged in future studies of complex disease etiology and personalized therapeutics or vaccines.

## Supplementary information

**Additional file 1.** This folder contains the pdf file with all Supplementary Figures (Figures S1-S9) that are referenced throughout the manuscript, with corresponding figure legends.

**Additional file 2.** This folder contains the pdf file with all Supplementary Tables (Tables S1-S28) referenced throughout the manuscript, with corresponding descriptions.

## Data Availability

The data used in this study are available via a direct application to the UK Biobank (http://www.ukbiobank.ac.uk/register-apply/). Individual-level data are open access and can be obtained after completing a registration process and obtaining approval from the UK Biobank Data Access committee. Type 1 Diabetes Genetics Consortium (T1DGC) reference panel used for imputation with SNP2HLA was obtained via approved access from the National Institute of Diabetes and Digestive and Kidney Disease (NIDDK) Central Repository: https://repository.niddk.nih.gov/studies/t1dgc-special/. Gene expression prediction models used in transcriptome-wide association analyses are available from the PredictDB repository, published on Zenodo [37]: https://zenodo.org/record/3518299.

## Abbreviations

BKV: BK polyomavirus
EBNA: Epstein-Barr nuclear antigen
EBV: Epstein-Barr virus
eQTL: Expression quantitative trait locus
GWAS: Genome-wide association study
HBV: Hepatitis B virus
HCV: Hepatitis C virus
HHV6: Human herpesvirus 6
HHV7: Human herpesvirus 7
HLA: Human leukocyte antigen
HPV16: Human papillomavirus 16
HPV18: Human papillomavirus 18
HSV1: Herpes simplex virus 1
HSV2: Herpes simplex virus
IgG: Immunoglobulin G
JCV: JC polyomavirus
MCV: Merkel cell polyomavirus
MFI: Median fluorescence intensity
OR: Odds ratio
SNP: Single nucleotide polymorphism
sQTL: Splicing quantitative trait locus
TWAS: Transcriptome-wide association study
UKB: UK Biobank
VZV: Varicella zoster virus
