# Supplementary material for: The landscape of host genetic factors involved in immune response to common viral infections
Source: Genome Med. 2020 Oct 27;12:93. doi: 10.1186/s13073-020-00790-x (PMC7590248; doi:10.1186/s13073-020-00790-x)
Supplement: Supplementary file 1 — Additional file 1. This folder contains the pdf file with all Supplementary Figures (Figures S1-S9) that are referenced throughout the manuscript, with corresponding figure legends. [file 13073_2020_790_MOESM1_ESM.pdf]

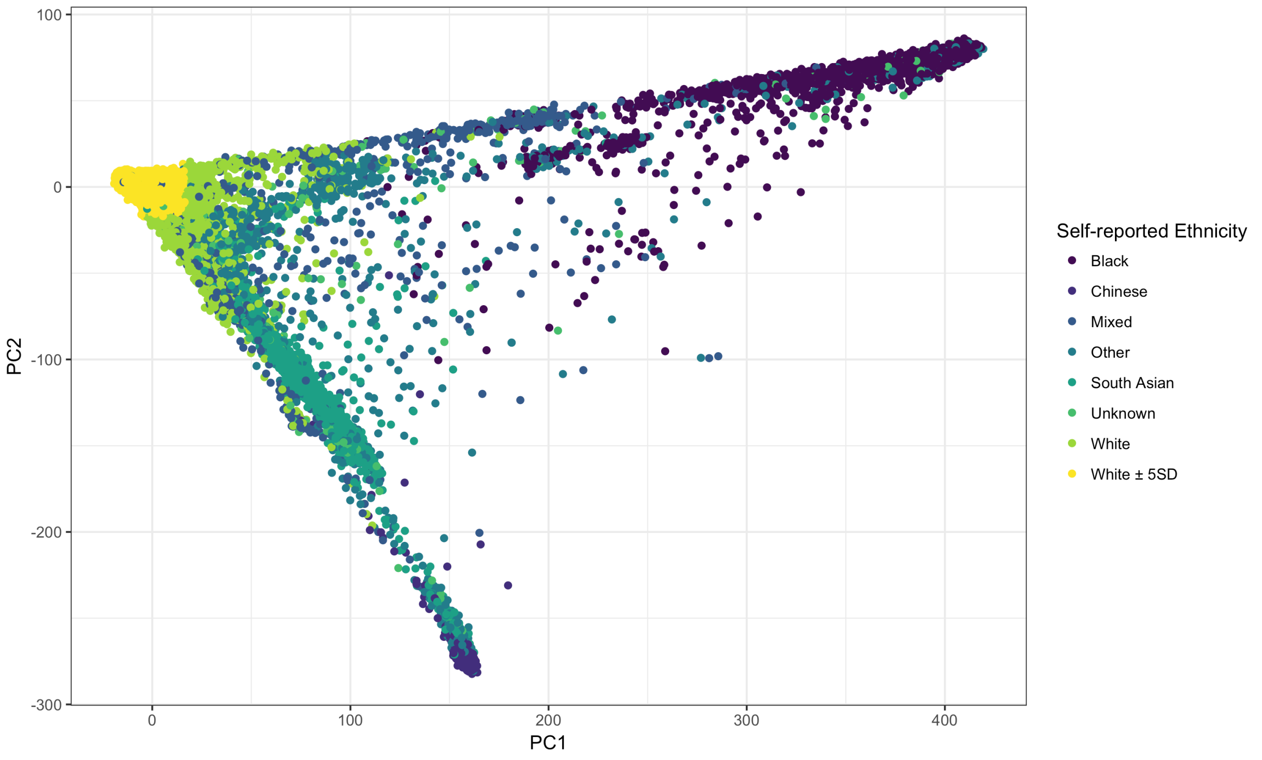

Figure S1: Genetic ancestry principal components plot visualizing the clustering of UK Biobank participants based on self-reported ancestry along principal components (PC) 1 and 2. For all analyses, European ancestry individuals were classified based on self-reported White ethnicity and PC1 and PC2 within 5 standard deviations (SD) of the population mean.

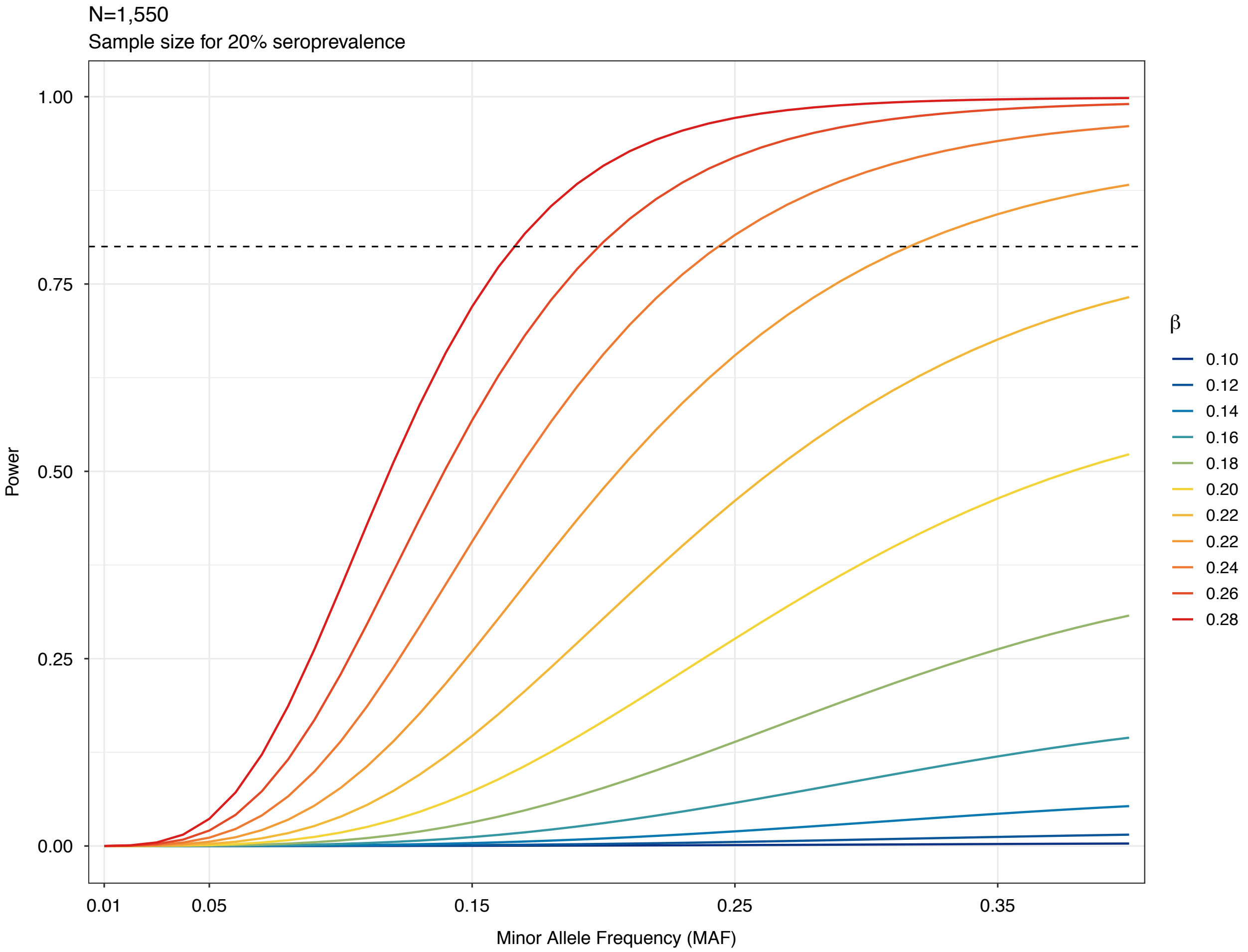

Figure S2: Power calculations for GWAS of continuous seroreactivity phenotypes assuming additive (per-allele) genetic effects and an alpha threshold of 5.0×10^{-8}

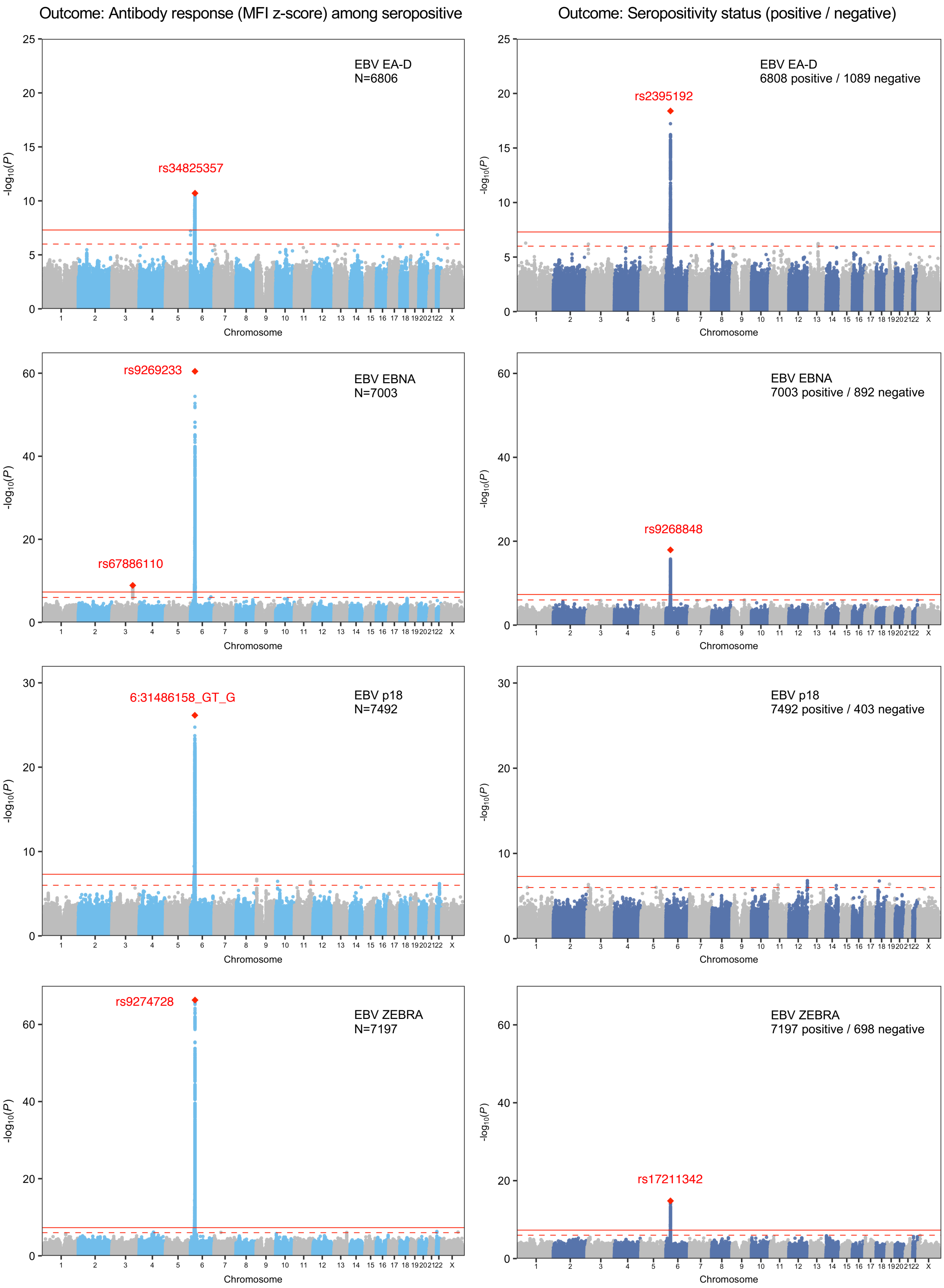

Additional File 2: Figures S1 – S9

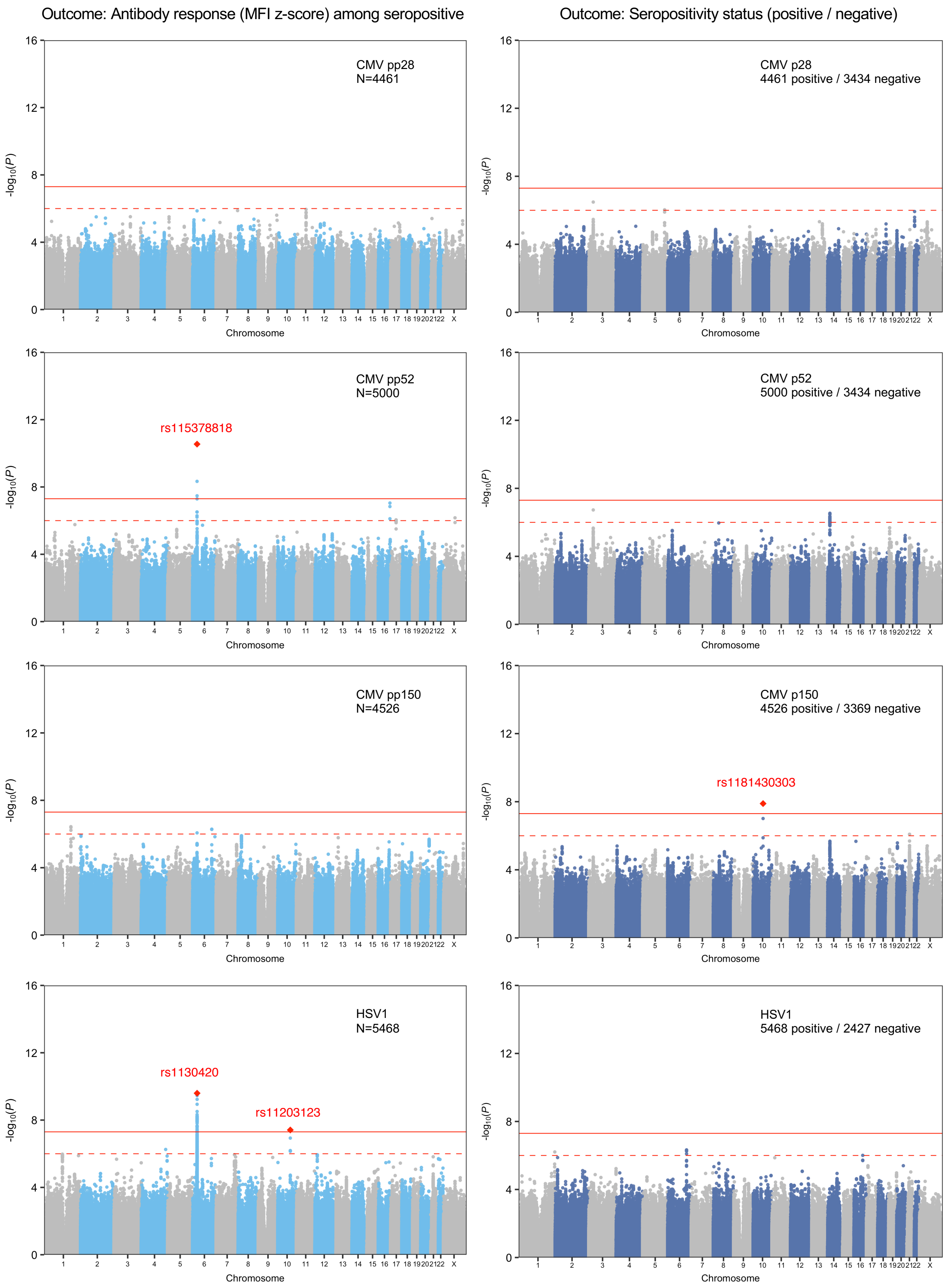

Additional File 2: Figures S1 – S9

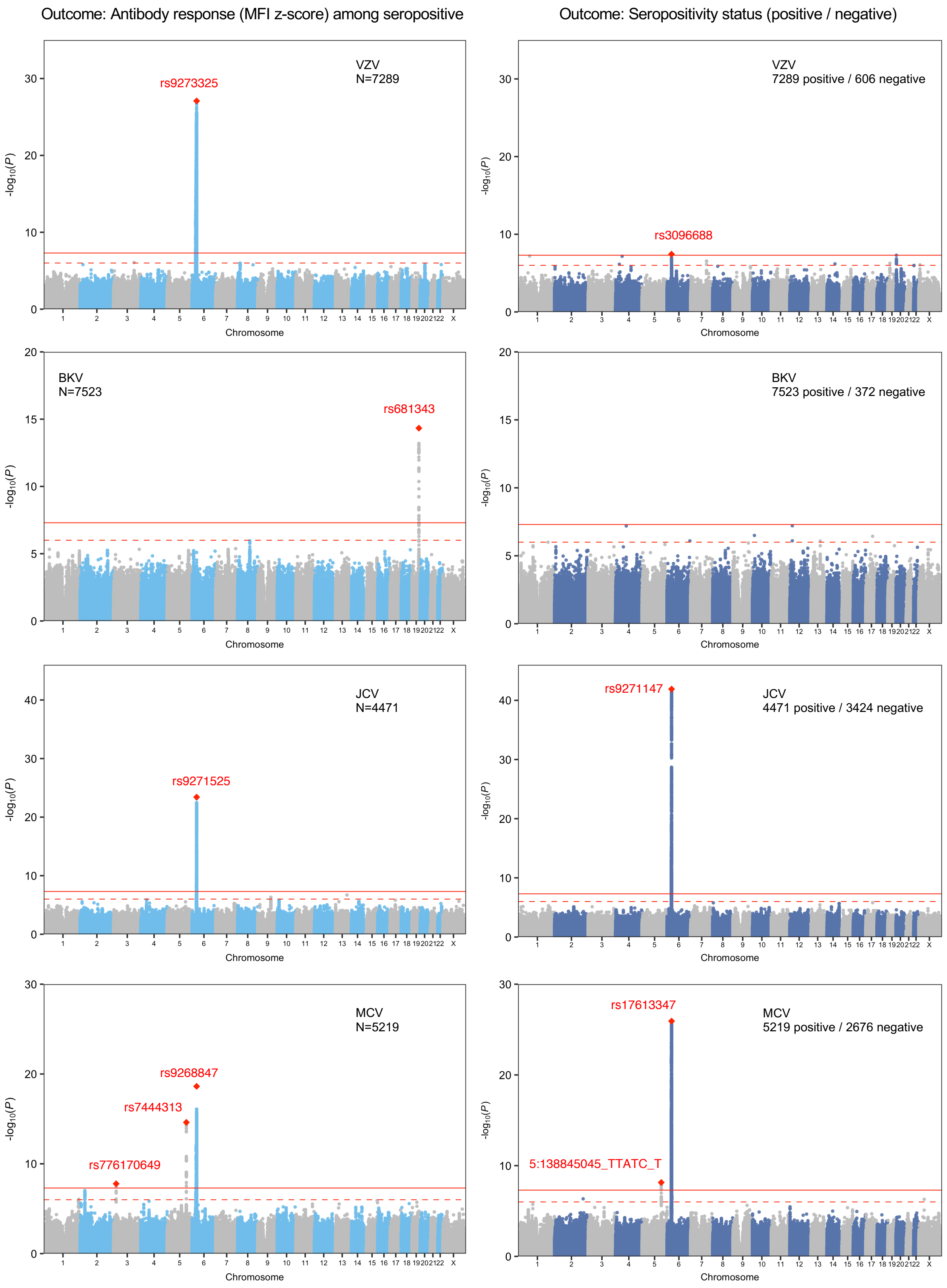

Additional File 2: Figures S1 – S9

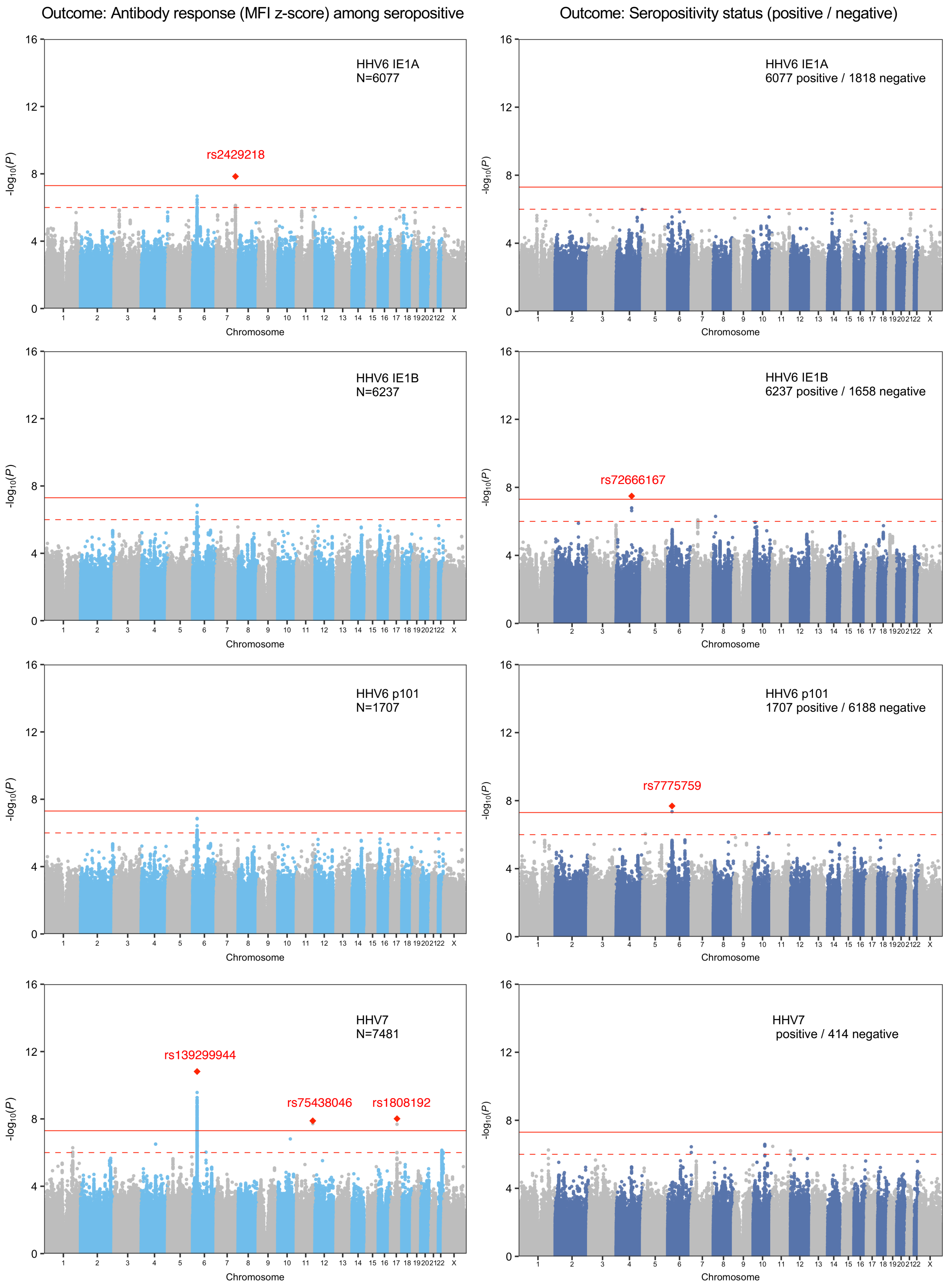

Additional File 2: Figures S1 – S9

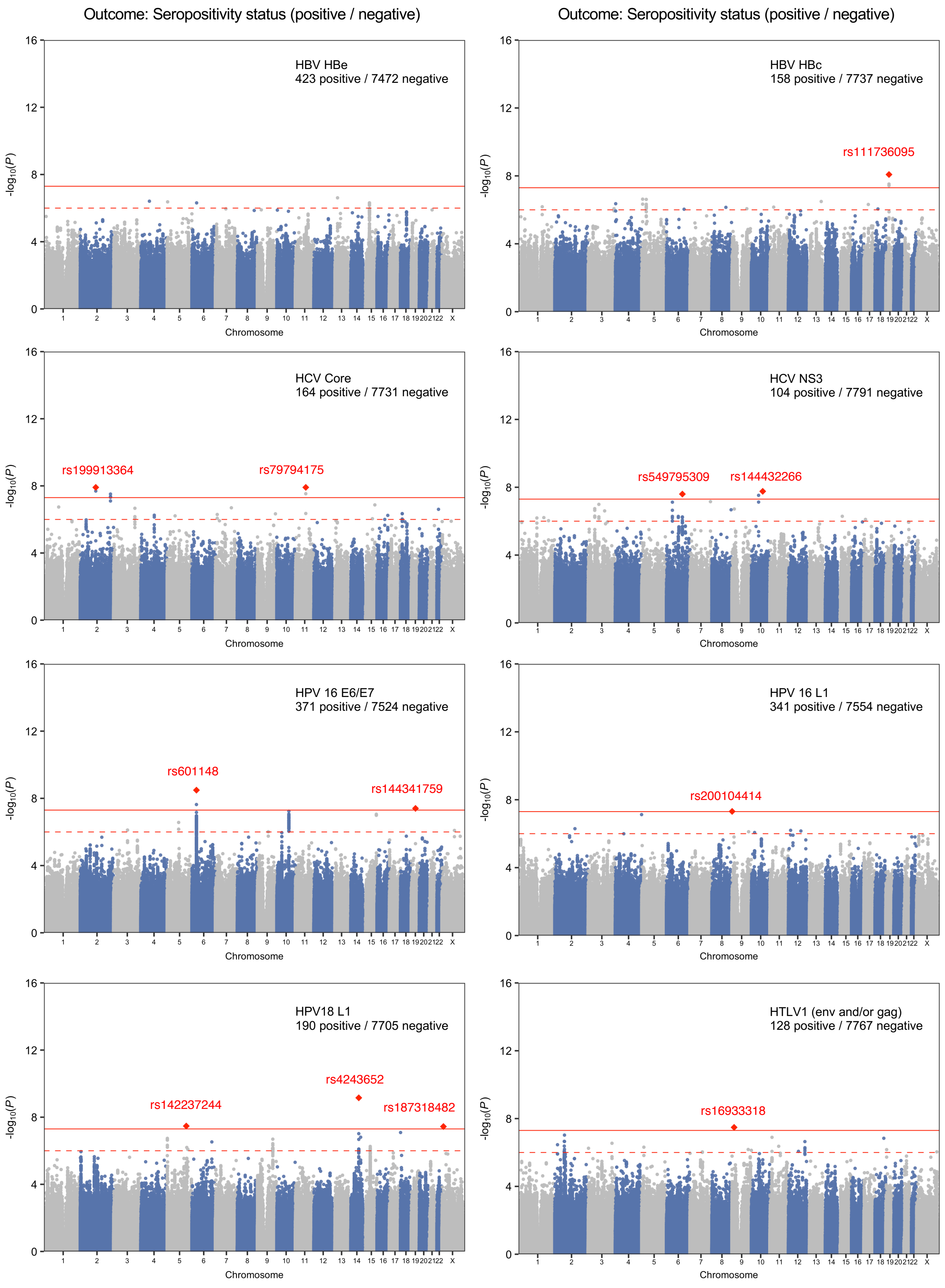

Additional File 2: Figures S1 – S9

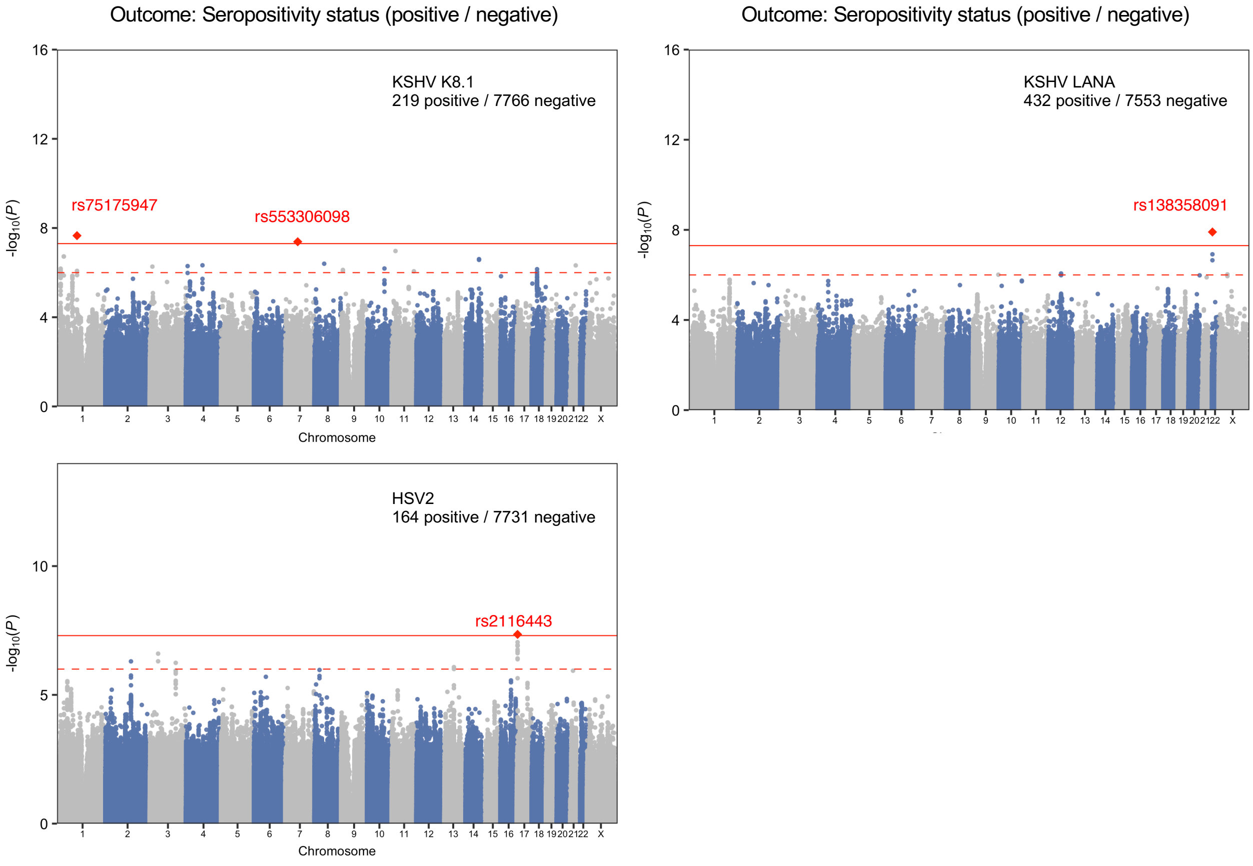

Figure S3: Manhattan plots visualizing genome-wide association results for continuous antibody response phenotypes (MFI z-scores) and dichotomous seropositivity phenotypes for each antigen.

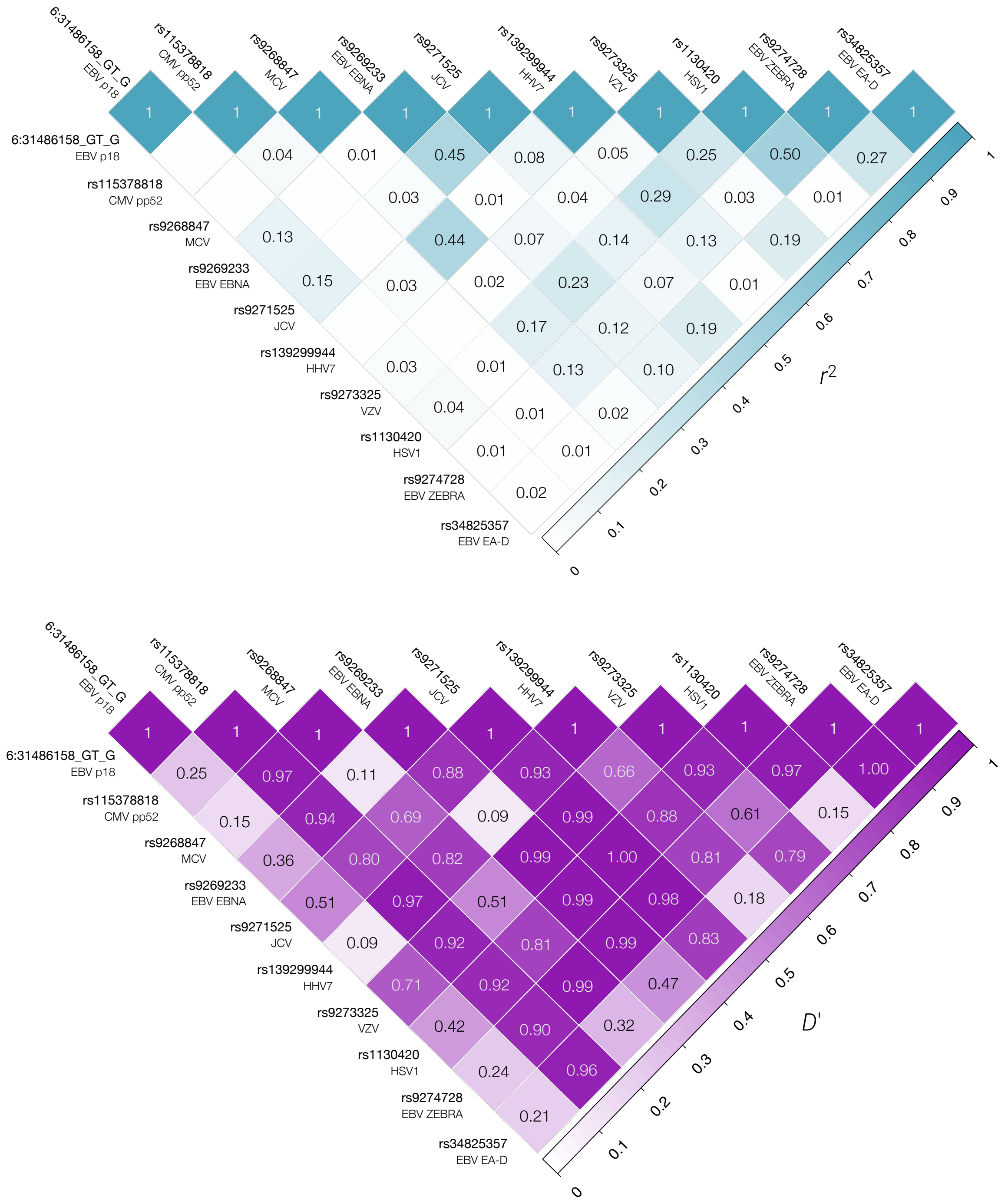

Figure S4: Linkage disequilibrium (LD) structure based on r^2 and D' between the top-ranking variants in HLA associated with continuous antibody response phenotypes (MFI z-scores) for each antigen/

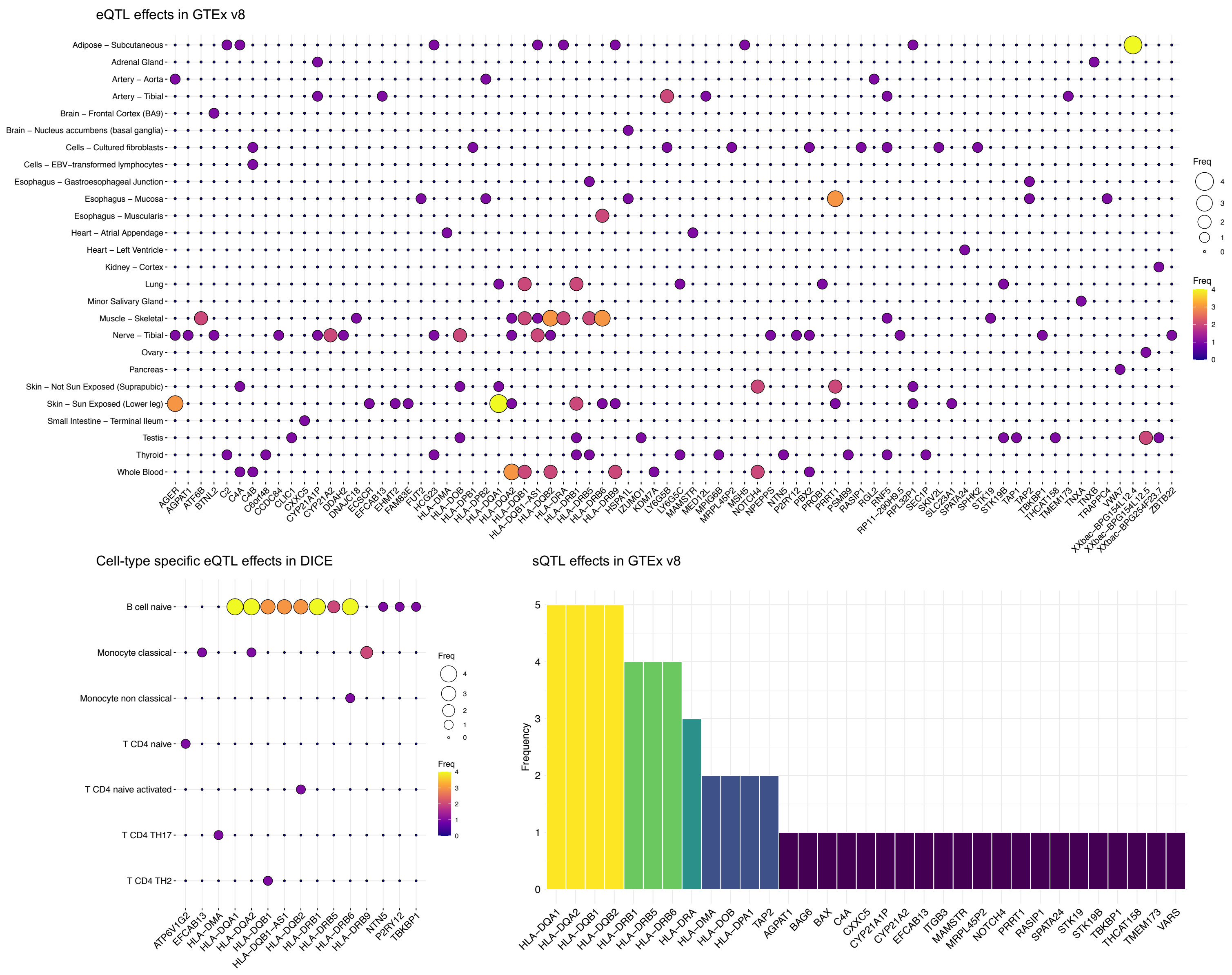

Figure S5: Summary of expression (eQTL) and splicing quantitative trait loci (sQTL) associations obtained in GTEx v8 and DICE (Database of Immune Cell Expression) for the genome-wide significant variants ($P<5.0\times10^{-8}$) for continuous antibody response phenotypes. In each panel, frequency corresponds to the number of variants with a specific gene-tissue or gene-cell combination.

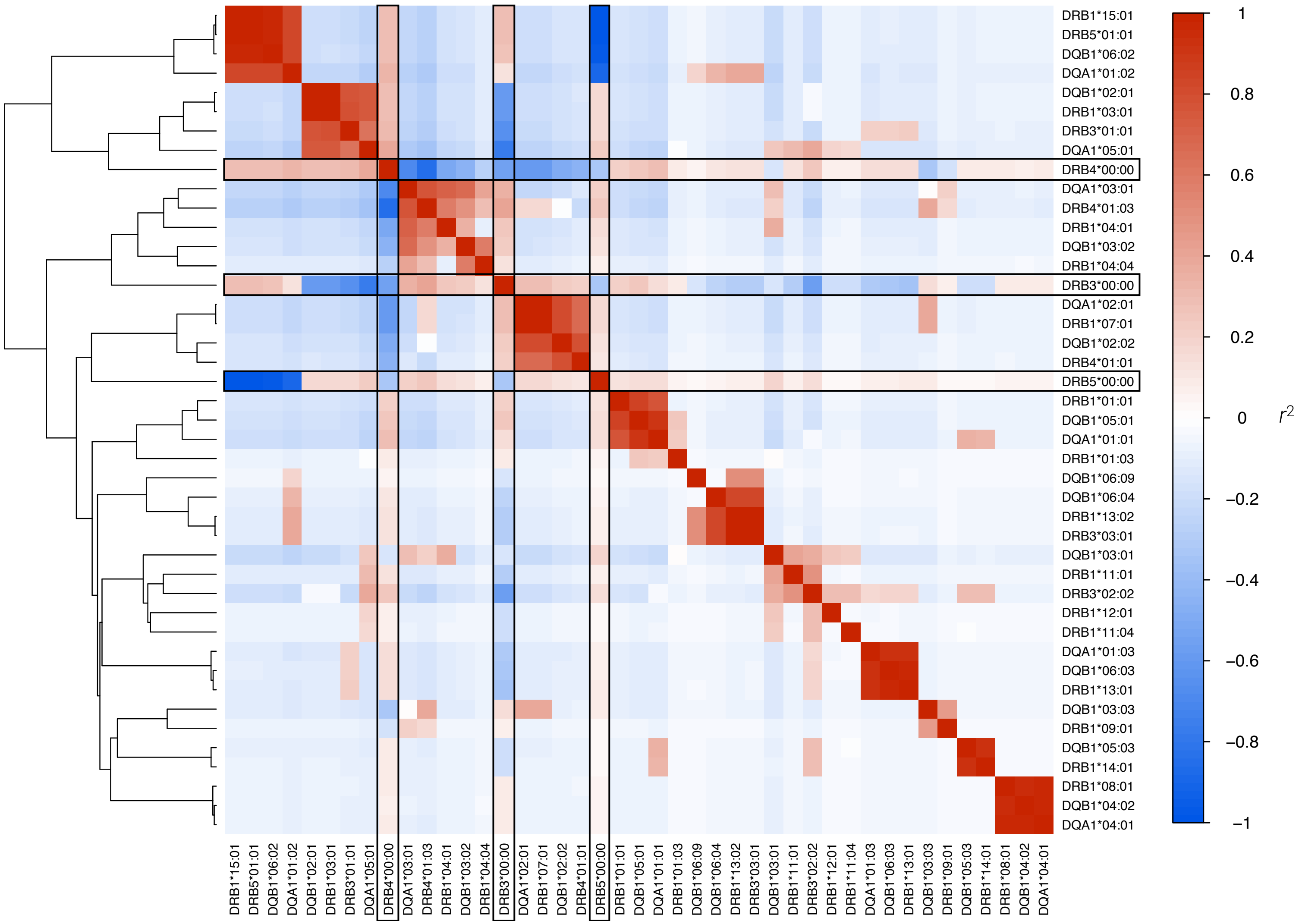

Figure S6: Linkage disequilibrium (LD) structure between classical HLA alleles in *HLA-DRB1*, *HLA-DRB3*, *HLA-DRB4*, *HLA-DRB5*, *HLA-DQA1*, and *HLA-DQB1* genes. Negative r^2 values indicate correlation with the absence of certain alleles.

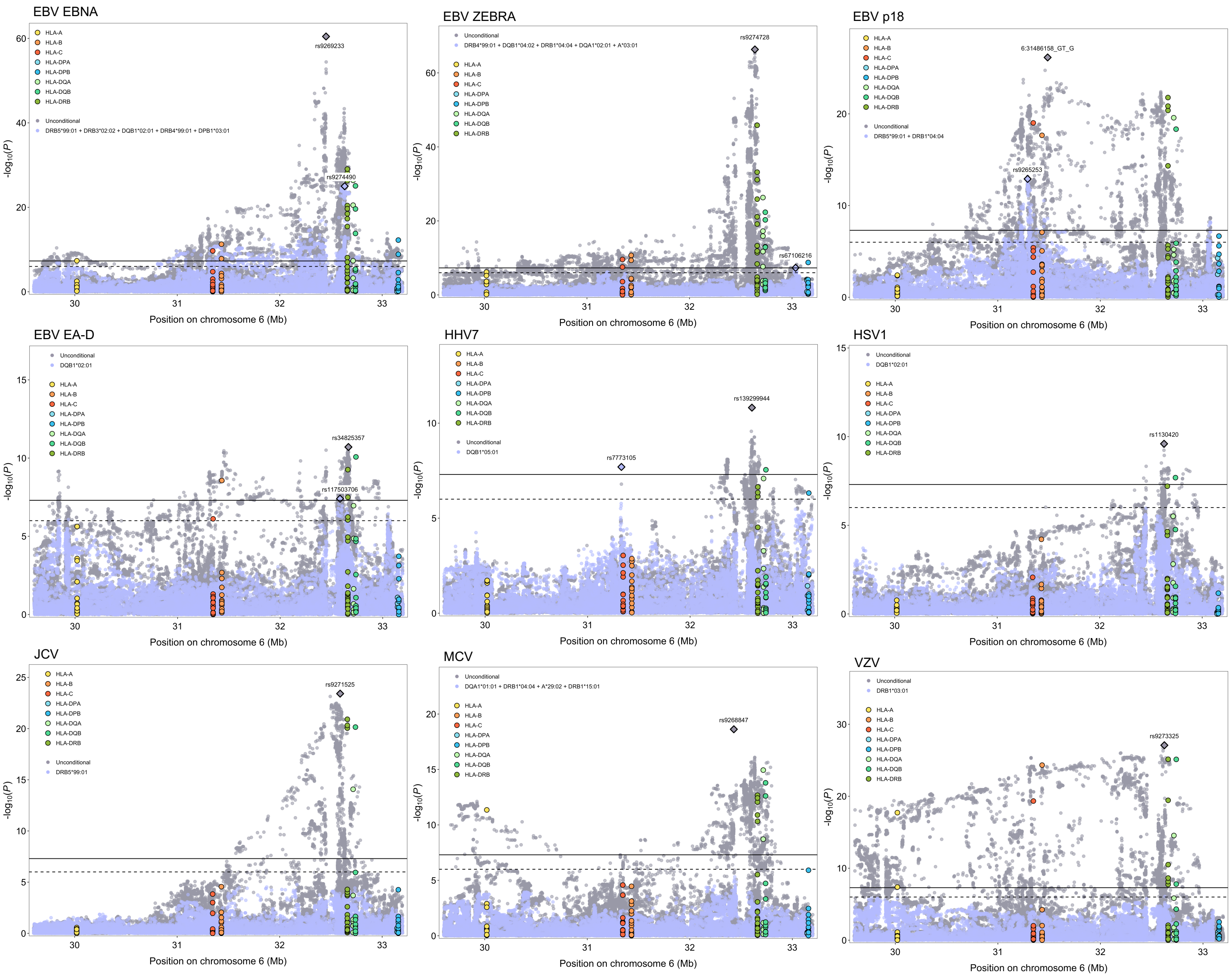

Figure S7: Regional association plots depicting results from analyses conditioning on statistically independent classical HLA allele.

Additional File 2: Figures S1 – S9

HHV7

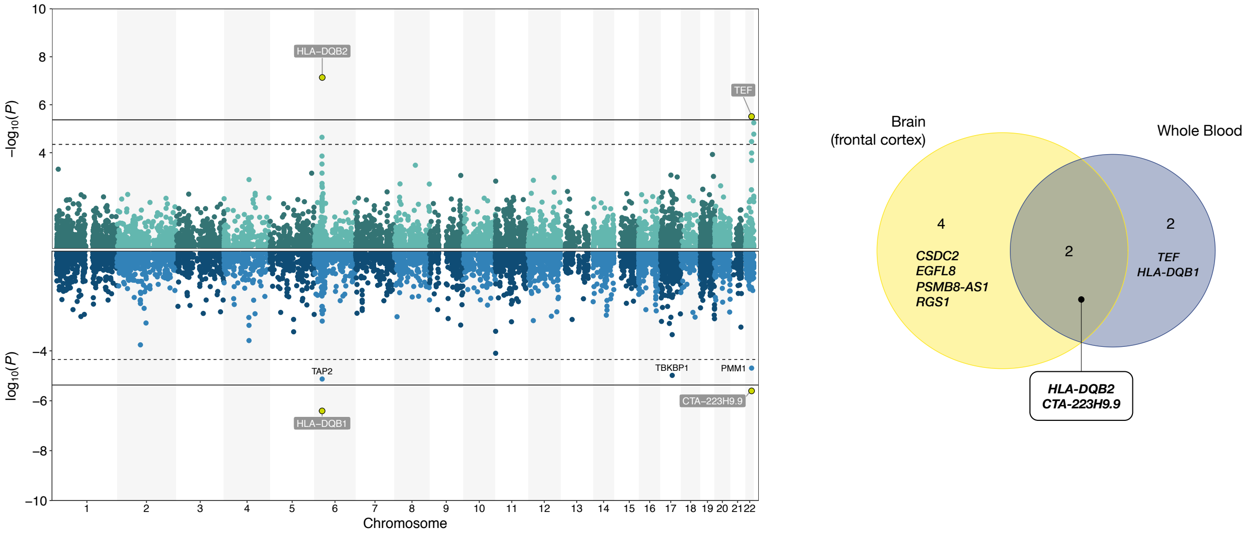

HSV1

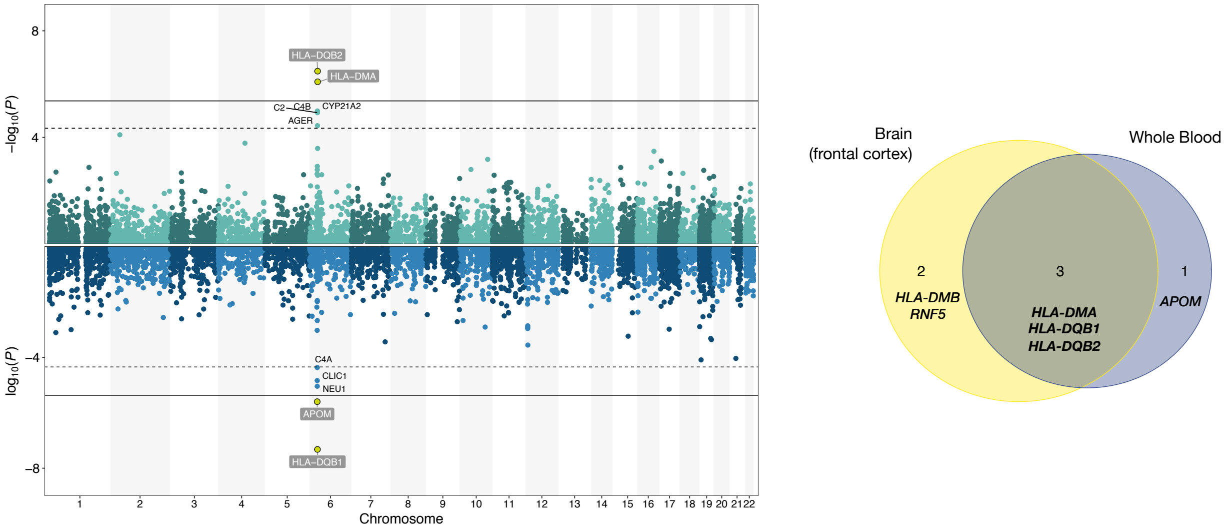

JCV

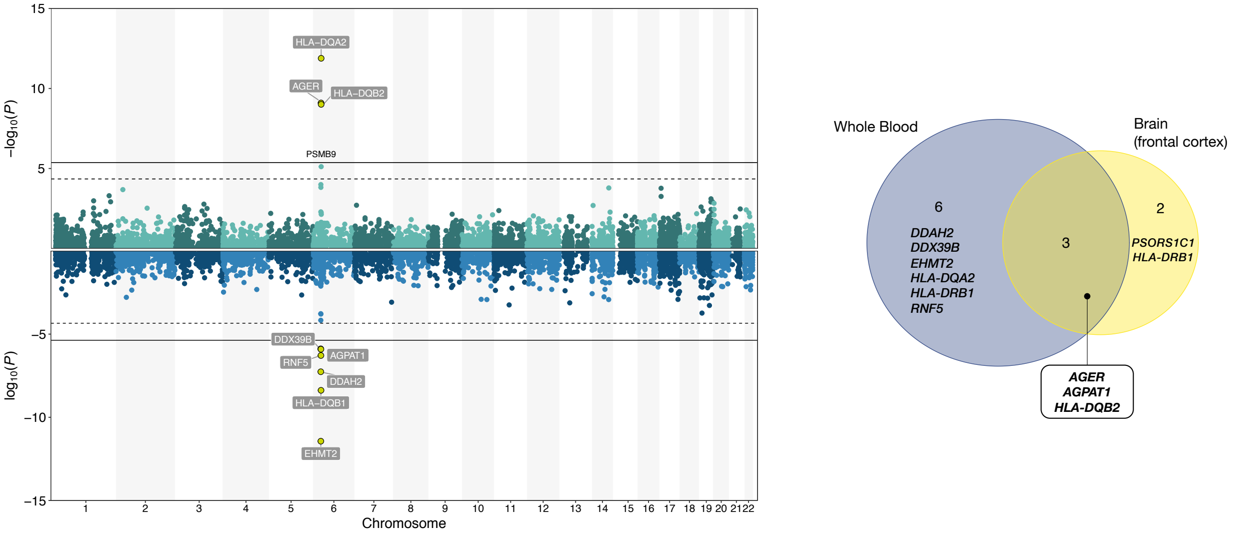

Additional File 2: Figures S1 – S9

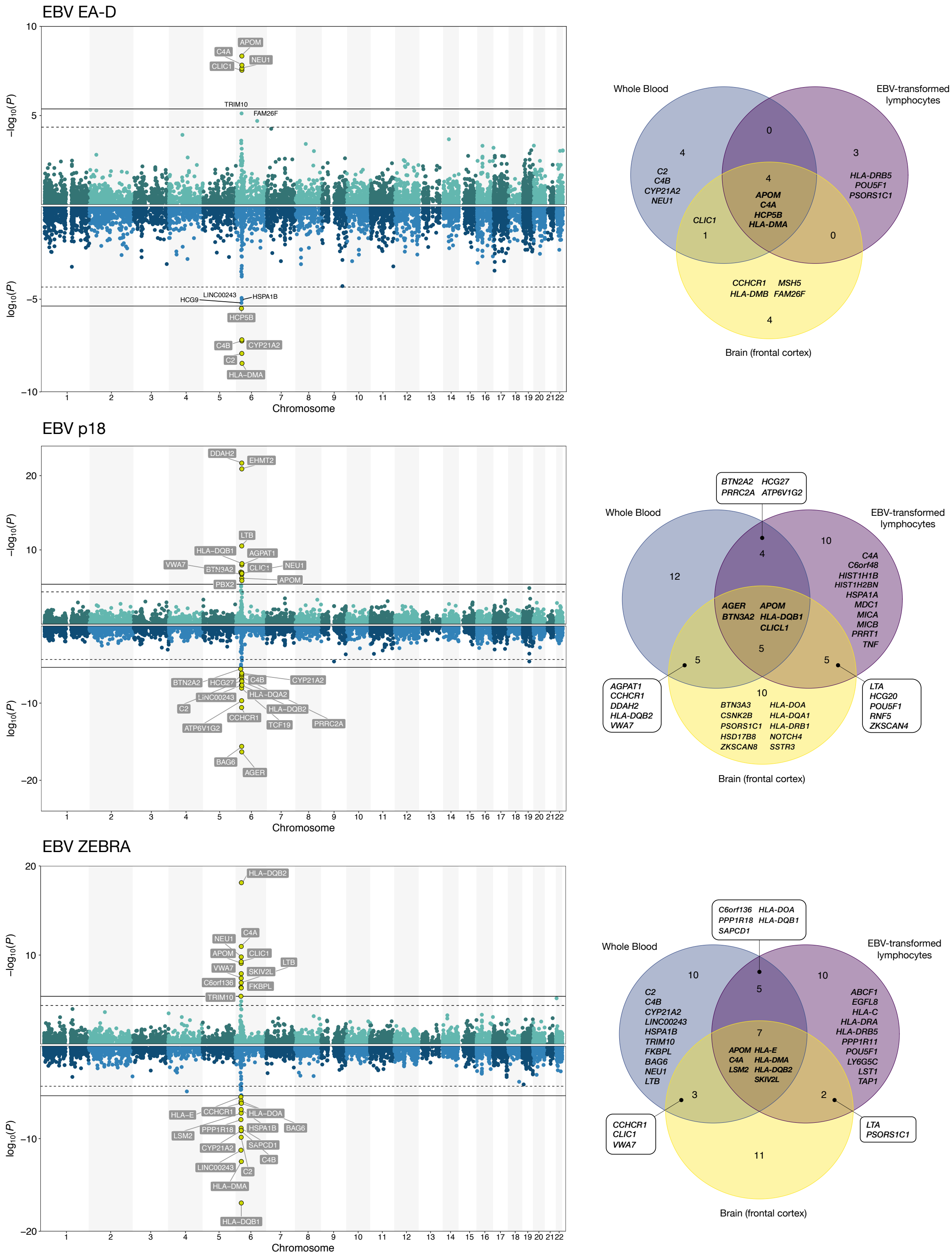

Supplementary Figure S8: TWAS associations with continuous antigen response phenotypes. Two Manhattan plots depicting the transcriptome-wide associations for genes with a positive direction of effect (increased expression leads to higher antibody response) and genes with a negative direction of effect (increased expression associated with a decreasing.

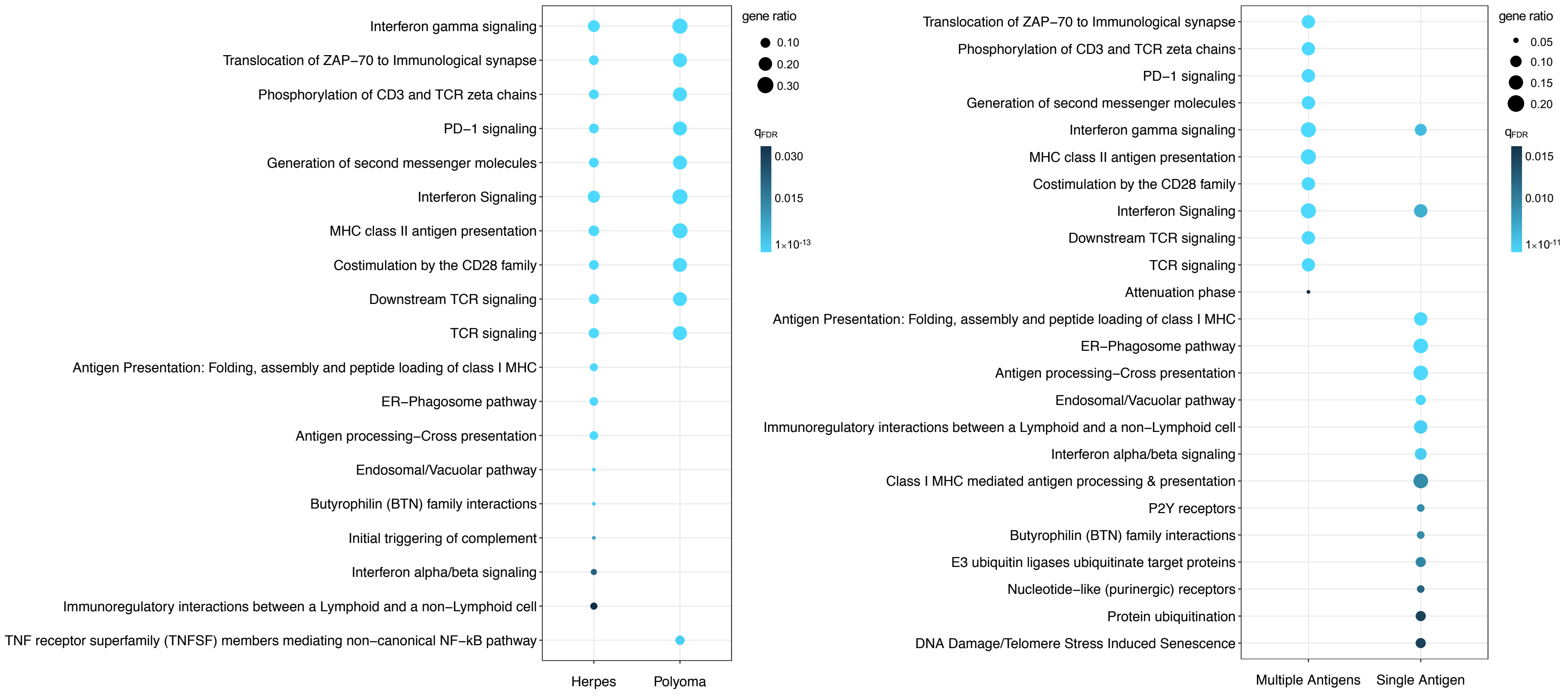

Figure S9: Visualization of significantly (q_{FDR}<0.05) enriched Reactome pathways for TWAS-identified genes grouped by virus family (human herpes viruses vs. human polyoma viruses) and specificity of association (multiple antigens vs. single antigen). Gene ratio corresponds to the size of the overlap between the input gene list with a specific gene set to the size of the overlap between the input gene list with all the members of the collection of gene sets.
