# Supplementary material for: The landscape of host genetic factors involved in immune response to common viral infections
Source: Genome Med. 2020 Oct 27;12:93. doi: 10.1186/s13073-020-00790-x (PMC7590248; doi:10.1186/s13073-020-00790-x)
Supplement: Supplementary file 2 — Additional file 2. This folder contains the pdf file with all Supplementary Tables (Tables S1-S28) referenced throughout the manuscript, with corresponding descriptions. [file 13073_2020_790_MOESM2_ESM.pdf]

Table S1: Characteristics of the UK Biobank serology sub-cohort consisting of predominantly European ancestry participants that remained after quality control filters.

Characteristic	Serology Data (N=7948)		No Serology Data (N=405,857)	
	N	(%)	N	(%)
Age at assessment				
Mean (SD)	56.74	(8.07)	56.74	(8.00)
Sex				
Male	3519	(44.3)	187685	(46.2)
Smoking status				
Never smoker	4348	(55.0)	218228	(54.0)
Former smoker	2774	(35.1)	143908	(35.6)
Current smoker	789	(14.48)	42285	(10.5)
Body-mass index (BMI) in kg/m^2				
Mean (SD)	27.36	(4.82)	27.41	(4.77)
Townsend deprivation index				
Mean (SD)	-1.52	(2.96)	-1.47	(2.99)
Autoimmune or chronic inflammatory condition	1033	(13.0)	50467	(12.4)
Any type of immune deficiency	1223	(0.3)	24	(0.3)
Cancer (prevalent or incident)	1069	(13.4)	52971	(13.1)

Included in genetic association analyses (N=7985)

Human Herpes viruses	Seropositive	(%)
Cytomegalovirus (CMV)	4437	(56.2)
CMV pp28	4461	(56.5)
CMV pp52	5000	(63.3)
CMV pp150	4526	(57.3)
Epstein-Barr virus (EBV)	7470	(94.6)
EBV EA-D	6806	(86.2)
EBV EBNA	7003	(88.7)
EBV VCA p18	7492	(94.9)
EBV ZEBRA	7197	(91.2)
Human Herpesvirus-6 (HHV6)	7156	(90.6)
HHV6 IE1A	6077	(77.0)
HHV6 IE1B	6237	(79.0)
HHV6 p101 k	6188	(78.4)
Human Herpesvirus-7 (HHV7) U14	7481	(94.8)
Herpes Simplex virus-1 (HSV1) 1gG	5468	(69.3)
Herpes Simplex virus-2 (HSV2) 2mgG	1214	(15.4)
Kaposi's Sarcoma-Associated Herpesvirus (KSHV)	607	(7.7)
KSHV K.81	219	(2.8)
KSHV LANA	432	(5.5)
VZV gE/Ig	7316	(92.3)

Human Polyomaviruses		
BKV	7523	(95.3)
JCV	4471	(56.6)
Merkel Cell Polyomavirus (MCV)	5219	(66.1)
Human Papillomaviruses		
HPV16 E6 or E7 (oncoproteins)	371	(4.7)
HPV16 L1	341	(4.3)
HPV18 L1	190	(2.4)
Hepatitis viruses		
Hepatitis B (HBV)	128	(1.6)
HBV HBc	158	(2.0)
HBV HBe	423	(5.4)
Hepatitis C (HCV)	23	(0.3)
HCV Core	164	(2.1)
HCV NS3	104	(1.3)
Human T-lymphotropic viruses		
Human T-lymphotropic Virus-1 (HTLV1)	128	(1.6)
HTLV1 env	70	(0.9)
HTLV1 gag	59	(0.3)

Table S2: Odds ratios (OR) for lead variants associated with seropositivity for each antigen. Linkage disequilibrium (LD) r^2 was calculated with respect to the lead variant for the continuous seroreactivity phenotype for the same antigen, if applicable.

Antigen		Chr	Position	Variant	Alleles		EAF	OR	P	Function	Nearest Gene	INFO	LD (r^2/ D′) with Seroreactivity Variant	
					Effect	Other								
CMV	pp150	10	71789604	rs1181430303	CAAT	C	0.988	0.362	1.3×10^{-8}	intergenic	*RP11-262I2.2*	0.974	-	
EBV	EA-D	6	32447644	rs2395192	C	T	0.447	0.656	4.0×10^{-19}	intergenic	*HLA-DRB9*	0.983	rs34825357:	0.669 / 0.880
EBV	EBNA	6	32429303	rs9268848	G	A	0.449	1.598	1.2×10^{-18}	intronic	*HLA-DRB9*	0.992	rs9269233:	0.293 / 0.854
EBV	ZEBRA	6	32578323	rs17211342	A	G	0.542	0.623	1.6×10^{-15}	intergenic	*HLA-DRB1*	0.999	rs9274728:	0.387 / 0.913
HHV6	IE1B	4	111337175	rs72666167	G	A	0.952	1.588	3.2×10^{-8}	intronic	*ENPEP / ZNF969P*	0.950	-	
HHV6	p101k	6	31352446	rs7775759	G	A	0.730	0.785	2.1×10^{-8}	intergenic	*HLA-S*	1.000	-	
HSV2	2mgG	17	3707723	rs2116443	C	T	0.485	1.279	4.5×10^{-8}	upstream	*ITGAE*	0.993	-	
KSHV	K8.1	1	94134854	rs75175947	G	C	0.988	0.221	2.2×10^{-8}	intronic	*BCAR3*	0.924	-	
		7	67785001	rs553306098	ATATAT	A	0.983	0.242	4.1×10^{-8}	upstream	*LOC105375341*	0.790	-	
KSHV	LANA	22	19399297	rs138358091	A	C	0.983	0.344	1.2×10^{-8}	intronic	*HIRA*	0.938	-	
VZV	gE/Ig	6	32161034	rs3096688	C	T	0.301	0.701	3.7×10^{-8}	intronic	*GPSM3*	0.995	rs9273325:	0.074 / 0.910
HPV16	E6/E7	6	32573265	rs601148	G	T	0.805	0.603	3.3×10^{-9}	intergenic	*HLA-DRB1*	0.973	-	
		19	30611215	rs144341759	G	A	0.975	0.383	4.0×10^{-8}	intergenic	*CTC-448F2.6*	0.920	-	
HPV16	L1	9	1061974	rs200104414	A	ATG	0.981	0.295	4.9×10^{-8}	intergenic	*RPS27AP14*	0.822	-	
HPV18	L1	5	139338366	rs142237244	G	A	0.987	0.201	3.4×10^{-8}	intronic	*NRG2*	0.890	-	
		14	77060652	rs4243652	G	A	0.035	3.132	7.0×10^{-10}	intronic	*LOC105370576*	0.987	-	
		X	12493856	rs187318482	C	G	0.989	0.271	3.6×10^{-8}	intronic	*FRMPD4*	0.750	-	
HBV	HBc	19	22899930	rs111736095	C	T	0.984	0.217	8.3×10^{-9}	upstream	*CTC-457E21.9*	0.898	-	
HCV	Core	2	112655155	rs199913364	CAG	C	0.980	0.248	1.2×10^{-8}	upstream	*MERTK*	0.942	-	
		11	75259052	rs79794175	C	T	0.933	0.406	1.2×10^{-8}	intergenic	*SERPINH1*	1	-	
HCV	NS3	6	115406379	rs549795309	C	T	0.979	0.204	2.5×10^{-8}	intergenic	*RP11-282C5.1*	0.886	-	
		10	82580895	rs144432266	A	C	0.989	0.158	1.7×10^{-8}	intergenic	*FARSBP1*	0.874	-	
JCV	VP1	6	32577385	rs9271147	T	C	0.152	0.536	1.3×10^{-42}	intergenic	*HLA-DQA1*	0.998	rs9271525:	0.940 / 1.000
MCV	VP1	6	32621980	rs17613347	T	C	0.150	0.613	1.2×10^{-26}	intergenic	*HLA-DQB1*	0.995	rs9268847:	0.055 / 0.975
		5	138845045	5: 138845045	TTATC	T	0.259	1.259	7.2×10^{-9}	intergenic	*ECSCR*	0.985	rs7444313:	0.946 / 0.981
HTLV1[1]		9	15483425	rs16933318	G	C	0.990	0.153	3.3×10^{-8}	intronic	*PSIP1*	0.948	-	

[1] Case counts for HTLV1 env and HTLV1 gag antigens were <100, therefore results are presented for HTLV1 based on seropositivity for at least one antigen

Table S3: Functional annotations for lead genome-wide significant variants ($P<5.0\times10^{-8}$) associated with antibody response. Expression (eQTL) and splicing quantitative trait loci (sQTL) associations with FDR<0.05 were obtained in GTEx v8 and DICE (Database of Immune Cell Expression). Plasma protein (pQTL) associations with $P<5.0\times10^{-8}$ were obtained from the Human Plasma Proteome atlas.

Phenotype		Chr	Position	Variant	Alleles		CADD[1]	RegulomeDB		eQTL (GTEx)		eQTL (DICE)		sQTL (GTEx)	pQTL
					Effect	Other		Score[2]	Rank[3]	Genes	Tissues	Genes	Cells	Genes	Proteins
CMV	pp52	6	32301427	rs115378818	C	T	4.55	0.29	5	-	-	-	-	-	-
EBV	EA-D	6	32665840	rs34825357	T	TC	10.63	0.63	5	25	49	-	-	-	12
EBV	EBNA	3	151114852	rs67886110	G	T	1.55	0.20	6	2	29	1	1	-	-
		6	32451762	rs9269233	A	C	0.60	0.13	5	27	48	4	10	13	4
EBV	p18	6	31486158	6:31486158	GT	G	0.02	-	-	-	-	1	5		-
EBV	ZEBRA	6	32637772	rs9274728	A	G	7.92	0.95	5	22	49	8	15	9	5
HHV6	IE1A	7	139985625	rs2429218	T	C	0.64	0.61	4	1	1	-	-	-	-
HHV7	U14	6	32602665	rs139299944	C	CT	12.15	0.72	5	15	48	-	-	9	-
		11	118767564	rs75438046	G	A	7.00	0.61	4	2	6	-	-		-
		17	45794706	rs1808192	A	G	4.18	0.55	1f	6	35	2	11	5	-
HSV1	1gG	6	32627852	rs1130420	G	A	9.73	0.74	2b	-	-	8	15	-	2
		10	91189187	rs11203123	A	C	1.41	0.53	5	-	-	-	-	-	-
VZV	gE/lg[1]	6	32623193	rs9273325	G	A	4.91	0.13	5	-	-	1	1	-	15
BKV	VP1	19	49206462	rs681343	C	T	0.06	0.13	5	8	40	1	7	3	16
JCV	VP1	6	32589842	rs9271525	G	A	10.73	0.15	3a	35	49	8	15	14	4
MCV	VP1	3	18238783	rs776170649	CT	C	15.61	0.35	6	-	-	-	-	-	-
		5	138865423	rs7444313	G	A	1.89	0.36	6	7	31	-	-	3	-
		6	32429277	rs9268847	A	G	5.57	0.18	7	16	28	-	-	8	-

[1] Combined Annotation Dependent Depletion (CADD) score: >10 corresponds to the top 10% most deleterious substitutions in the genome

[2] Score: ranges from 0 to 1, with 1 being most likely to be a regulatory variant. This score is based on a model that integrates functional genomics features with continuous values such as ChIP-seq signal, DNase-seq signal, information content change, and DeepSEA scores

[3] Rank: 1f = eQTL + TF binding / DNase peak; 2b = TF binding + any motif + DNase Footprint + DNase peak; 3a = TF binding + any motif + DNase peak; 4= TF binding + DNase peak; 5= TF binding or DNase peak; 6 or greater = minimal evidence

Table S4: Significant effects ($P<5.0\times10^{-8}$) on plasma protein levels (pQTL) observed for genome-wide significant variants for viral antigen IgG levels using data from the Human Plasma Proteome atlas.

Phenotype	Variant	Target Protein	P_{pQTL}	Source Dataset [PMID]
EBV EA-D	rs34825357	Glutamate receptor 4	1.5×10^{-28}	Sun et al. [29875488]
		T-cell surface protein tactile	6.5×10^{-21}	
		Complement C4	1.2×10^{-20}	
		cAMP-specific 3,5-cyclic phosphodiesterase 4D	2.8×10^{-20}	
		HLA class II histocompatibility antigen, DQ alpha 2 chain	1.1×10^{-19}	
		Vesicle-fusing ATPase	5.9×10^{-12}	
		Beta-defensin 119	1.0×10^{-10}	
		Killer cell immunoglobulin-like receptor 2DS2	4.0×10^{-10}	
		Interleukin-21	4.6×10^{-10}	
		GDH/6PGL endoplasmic bifunctional protein	5.9×10^{-10}	
		MHC class I polypeptide-related sequence B	8.3×10^{-10}	
		Trypsin-3	2.9×10^{-8}	
EBV EBNA	rs9269233	HLA class II histocompatibility antigen, DQ alpha 2 chain	2.0×10^{-21}	Sun et al. [29875488]
		GDH/6PGL endoplasmic bifunctional protein	3.2×10^{-13}	
		Killer cell immunoglobulin-like receptor 2DS2	1.3×10^{-10}	
		Ameloblastin	3.7×10^{-8}	
EBV ZEBRA	rs9274728	Complement C4	6.9×10^{-16}	Sun et al. [29875488]
		MHC class I polypeptide-related sequence B	6.6×10^{-11}	
		Interleukin-21	2.2×10^{-10}	
		Ubiquitin carboxyl-terminal hydrolase 25	8.9×10^{-10}	
		T-cell surface protein tactile	3.8×10^{-8}	
HSV1	rs1130420	Trypsin-3	7.2×10^{-15}	Sun et al. [29875488]
		Rac GTPase-activating protein 1	1.7×10^{-13}	
VZV	rs9273325	HLA class II histocompatibility antigen, DQ alpha 2 chain	4.9×10^{-38}	Sun et al. [29875488]
		Glutamate receptor 4	6.8×10^{-26}	
		cAMP-specific 3,5-cyclic phosphodiesterase 4D	1.7×10^{-24}	
		MHC class I polypeptide-related sequence B	8.3×10^{-22}	
		Complement C4	2.0×10^{-18}	
		T-cell surface protein tactile	7.6×10^{-16}	
		Trypsin-3	2.0×10^{-13}	
		Rac GTPase-activating protein 1	4.5×10^{-11}	
		Beta-defensin 119	3.6×10^{-10}	
		Interleukin-21	1.9×10^{-9}	
		Tenascin-X	2.0×10^{-9}	
		Ubiquitin-like protein ISG15	3.9×10^{-9}	
		Palmitoyl-protein thioesterase 1	4.5×10^{-9}	
		Polypeptide N-acetylgalactosaminyltransferase 1	4.6×10^{-9}	
		Coiled-coil domain-containing protein 134	4.6×10^{-8}	

		Protein FAM3D	1.8×10^{-110}	
		Golgi membrane protein 1	1.5×10^{-28}	
		C-C motif chemokine 15	2.2×10^{-27}	Sun et al. [29875488]
		Transcobalamin-1	1.8×10^{-21}	
		Secreted and transmembrane protein 1	7.2×10^{-20}	
		Lithostathine-1-alpha	2.3×10^{-18}	Yao et al. [30111768]
		Protein FAM177A1	5.5×10^{-18}	
		Galactoside 3(4)-L-fucosyltransferase	4.0×10^{-16}	
BKV	rs681343	Intestinal-type alkaline phosphatase	1.3×10^{-15}	
		C-C motif chemokine 25	1.4×10^{-13}	Sun et al. [29875488]
		Fibroblast growth factor 19 (FGF19)	2.6×10^{-13}	
		BPI fold-containing family B member 1 (BPIFB1)	5.4×10^{-10}	
		N-acetyllactosaminide beta-1,3-N-acetylglucosaminyltransferase 2 (FUT3)	6.8×10^{-9}	Sun et al. [29875488]
		Lactase-phlorizin hydrolase	1.2×10^{-8}	
		Tissue factor	8.7×10^{-9}	Folkersen et al. [28369058]
		Stromelysin-2	3.5×10^{-8}	
		GDH/6PGL endoplasmic bifunctional protein	4.2×10^{-26}	
JCV	rs9271525	Killer cell immunoglobulin-like receptor 2DS2	2.2×10^{-11}	Sun et al. [29875488]
		Osteocalcin	6.5×10^{-9}	
		Complement factor B	3.1×10^{-8}	

Table S5: Odds ratios (OR) for the effect of top seroreactivity variants on selected cancers, schizophrenia, and Alzheimer's disease (AD). Associations with $P<7.3\times10^{-4}$ were considered statistically significant after Bonferroni correction for the number of genetic variants and phenotypes tested.

Antigen		Chr	Position	Variant	Alleles		Effect on antibody response (MFI)		Hematologic cancers[1] 3262 cases / 410,350 controls		Melanoma 6777 cases / 410,350 controls		Schizophrenia[2] 33,640 cases / 43,456 controls		AD[3] 17,536 cases / 53,711 controls
					Effect	Other	Beta	(SE)	OR	P	OR	P	OR	P	P
CMV	pp52	6	32301427	rs115378818	C	T	0.633	(0.095)	0.809	0.058	0.886	0.12	0.989	0.84	0.44
EBV	EA-D	6	32665840	rs34825357†	T	TC	-0.114	(0.017)	0.978	0.45	1.054	0.017	0.960	2.7×10^{-4}	0.75
EBV	EBNA	3	151114852	rs67886110*	G	T	0.103	(0.017)	0.960	0.11	0.993	0.68	1.023	0.037	0.068
		6	32451762	rs9269233†	A	C	0.315	(0.019)	0.878	2.7×10^{-4}	1.078	2.8×10^{-3}	1.034	6.2×10^{-3}	0.28
EBV	VCA p18	6	31486158	6:31486158†	GT	G	0.197	(0.018)	0.921	6.1×10^{-3}	0.993	0.75	1.030	0.020	0.016
EBV	ZEBRA	6	32637772	rs9274728	A	G	-0.315	(0.018)	0.975	0.35	1.060	3.3×10^{-3}	0.993	0.59	1.7×10^{-3}
HHV6	IE1A	7	139985625	rs2429218	T	C	0.106	(0.019)	1.035	0.19	0.990	0.58	1.024	0.041	0.24
HHV7	U14	6	32602665	rs139299944	C	CT	0.114	(0.017)	1.079	4.1×10^{-3}	0.986	0.43	0.990	0.38	-
		11	118767564	rs75438046	G	A	0.280	(0.049)	0.913	0.22	0.919	0.11	0.955	0.18	4.1×10^{-3}
		17	45794706	rs1808192†	A	G	-0.099	(0.017)	0.989	0.69	0.981	0.32	1.000	0.99	0.029
HSV1	1gG	6	32627852	rs1130420†	G	A	-0.122	(0.019)	0.888	3.5×10^{-6}	1.023	0.22	1.055	1.8×10^{-5}	1.2×10^{-4}
		10	91189187	rs11203123*†	A	C	0.512	(0.093)	0.948	0.71	0.966	0.75	0.985	0.70	0.85
VZV	gE/lg[1]	6	32623193	rs9273325	G	A	-0.232	(0.021)	0.876	4.4×10^{-5}	0.965	0.12	1.131	4.3×10^{-15}	0.16
BKV	VP1	19	49206462	rs681343	C	T	-0.125	(0.016)	1.004	0.87	1.037	0.040	0.960	2.5×10^{-4}	0.16
JCV	VP1	6	32589842	rs9271525	G	A	-0.318	(0.031)	0.925	0.026	1.015	0.53	1.060	6.8×10^{-5}	4.7×10^{-3}
MCV	VP1	3	18238783	rs776170649†	CT	C	-0.134	(0.024)	1.006	0.86	0.975	0.23	1.045	9.0×10^{-4}	0.83
		5	138865423	rs7444313	G	A	0.169	(0.021)	0.957	0.13	1.026	0.21	1.019	0.12	0.033
		6	32429277	rs9268847	A	G	-0.195	(0.022)	1.018	0.54	0.949	8.3×10^{-3}	0.968	8.4×10^{-3}	0.14

[1] Cancer types included: non-Hodgkin lymphoma and lymphocytic leukemia

[2] Estimates obtained from a meta-analysis of the UK Biobank and Genetic Epidemiology Research on Aging cohorts [Rashkin et al. (2019) *bioRxiv* 635367]

[3] Summary statistics for European ancestry subjects were obtained from Lam, Chen et al. *Nat Genet.* (2019) 51(12):1670-1678 [PMID: 31740837]

[4] Summary statistics obtained from Jun et al. *Mol Psychiatry.* (2016) 21(1):108-17 [PMID: 25778476]

† Proxies for unavailable variants: rs2647006 for rs34825357 (schizophrenia and AD; LD r^2=0.99); rs7755774 for rs9269233 (AD; LD r^2=0.71); rs3093986 for 6:31486158_GT_G (AD; LD r^2=0.74); rs7219420 for rs1808192 (AD; LD r^2=0.87); rs4713572 for rs1130420 (AD; LD r^2=0.91); rs11203124 for rs11203123 (AD; LD r^2=1.00); rs7618405 for rs776170649 (AD; LD r^2=0.99)

* Multi-allelic variants: rs67886110 (G/T and G/C) and rs11203123 (A/C and A/AC)

Table S6: Independent sentinel variants in the HLA region identified by clumping (LD $r^2<0.05$ ±500kb) associations with $P<5\times10^{-8}$ for each antigen response phenotype.

Antigen	Chr	Position	Variant	Alleles Effect	Alleles Other	EAF	Beta	(SE)	P	INFO	Clump Size[1]
CMV pp52	6	32301427	rs115378818	C	T	0.986	0.633	(0.095)	2.8×10^{-11}	0.731	2
EBV EA-D	6	32665840	rs34825357	T	TC	0.409	-0.114	(0.017)	2.0×10^{-11}	0.998	779
	6	31451370	rs3132470	A	G	0.855	0.159	(0.024)	3.4×10^{-11}	1.000	207
	6	29842444	rs1611657	G	A	0.458	0.105	(0.017)	6.9×10^{-10}	0.996	19
	6	32583876	rs117503706	G	A	0.986	-0.423	(0.072)	4.8×10^{-9}	0.912	15
	6	30764907	rs1264377	G	A	0.818	0.119	(0.022)	4.7×10^{-8}	1	0
EBV EBNA	6	32451762	rs9269233	A	C	0.249	0.315	(0.019)	3.5×10^{-61}	0.961	3885
	6	32628432	rs9273507	A	G	0.570	0.167	(0.017)	7.5×10^{-23}	0.994	228
	6	31884823	rs3130682	T	C	0.148	0.220	(0.023)	3.0×10^{-21}	0.993	269
	6	32390436	rs143810596	T	G	0.977	-0.538	(0.058)	1.3×10^{-20}	0.989	51
	6	31297772	rs9265517	C	T	0.233	0.172	(0.020)	4.5×10^{-18}	0.975	967
	6	33048628	rs1042136	A	C	0.838	0.176	(0.023)	1.3×10^{-14}	0.991	158
	6	31835164	rs693906	G	C	0.842	0.177	(0.023)	2.0×10^{-14}	0.984	130
	6	31892641	rs9267677	T	C	0.905	0.206	(0.028)	3.6×10^{-13}	1	58
	6	32201469	rs41315395	C	A	0.855	-0.167	(0.023)	1.2×10^{-12}	0.997	3
	6	32387809	rs2395163	T	C	0.779	0.144	(0.020)	2.0×10^{-12}	1	25
	6	29822779	6:29822779	AAAAC	A	0.365	0.114	(0.017)	4.1×10^{-11}	0.999	113
	6	33083096	rs6457714	T	A	0.789	-0.132	(0.020)	6.8×10^{-11}	0.997	35
	6	32609147	rs12722051	A	T	0.795	0.127	(0.021)	1.3×10^{-9}	0.989	7
	6	31198786	rs3130935	A	G	0.621	0.098	(0.017)	1.5×10^{-8}	1.000	7
	6	32623017	rs28468461	T	C	0.969	-0.256	(0.046)	2.9×10^{-8}	0.993	0
	6	32542378	rs116206645	T	A	0.979	0.403	(0.074)	5.0×10^{-8}	0.699	0
EBV p18	6	31486158	6:31486158	GT	G	0.245	0.197	(0.018)	7.1×10^{-27}	0.994	1651
	6	32589978	rs9271536	A	T	0.179	0.200	(0.020)	3.9×10^{-23}	0.999	2455
	6	32069806	rs3096695	G	C	0.145	0.208	(0.022)	1.6×10^{-20}	0.997	16
	6	30921417	6:30921417	GA	G	0.119	0.165	(0.025)	2.7×10^{-11}	0.975	42
	6	30727983	rs3095339	A	G	0.722	0.112	(0.017)	7.3×10^{-11}	1.000	24
	6	32797876	rs241436	A	G	0.549	0.099	(0.016)	2.4×10^{-10}	1	2
	6	31379391	rs3828879	C	T	0.876	0.147	(0.024)	5.1×10^{-10}	1.000	3
	6	32206539	rs3134937	C	T	0.751	0.112	(0.018)	6.3×10^{-10}	0.981	20
	6	33073440	rs2064475	G	A	0.768	0.111	(0.019)	2.2×10^{-9}	1.000	21
	6	32045864	rs781341398	GAA	G	0.966	-0.277	(0.046)	2.7×10^{-9}	0.922	2
	6	32754876	rs116309362	T	C	0.976	-0.335	(0.057)	3.5×10^{-9}	0.895	1
	6	26370707	rs9379862	T	C	0.743	0.103	(0.018)	5.6×10^{-9}	1.000	1
	6	31448564	rs3099843	G	T	0.855	0.126	(0.022)	1.1×10^{-8}	1.000	12
	6	31362207	rs1052409	T	C	0.845	0.125	(0.022)	1.2×10^{-8}	0.978	7
	6	28039586	rs4713135	G	A	0.761	0.102	(0.019)	3.4×10^{-8}	1	0
	6	32637772	rs9274728	A	G	0.718	-0.315	(0.018)	4.7×10^{-67}	0.995	3836

EBV ZEBRA	6	32045864	rs781341398	GAA	G	0.966	-0.428	(0.048)	5.1×10^{-19}	0.922	20
	6	32211085	rs412657	T	G	0.622	0.145	(0.017)	1.0×10^{-17}	1	321
	6	32774954	rs6917611	G	C	0.608	0.142	(0.017)	1.9×10^{-17}	1.000	198
	6	32634588	rs113140854	C	T	0.983	-0.534	(0.067)	1.5×10^{-15}	0.827	253
	6	31672242	rs28366157	A	G	0.937	-0.266	(0.034)	2.3×10^{-15}	0.997	374
	6	31418810	rs3131623	T	A	0.817	0.167	(0.021)	2.6×10^{-15}	0.992	610
	6	29923351	rs2904758	G	A	0.280	0.144	(0.018)	5.0×10^{-15}	0.990	97
	6	32635948	rs541213498	C	T	0.987	-0.586	(0.078)	4.8×10^{-14}	0.780	5
	6	32626702	rs35779483	A	G	0.693	-0.135	(0.018)	6.1×10^{-14}	0.999	33
	6	30820373	rs2263298	C	T	0.849	0.154	(0.023)	2.4×10^{-11}	1.000	200
	6	33017502	rs3130177	G	A	0.717	-0.121	(0.018)	3.1×10^{-11}	0.996	5
	6	31150844	rs138206303	A	AGT	0.883	-0.167	(0.025)	4.8×10^{-11}	0.975	22
	6	31928799	rs419788	T	C	0.324	-0.113	(0.017)	1.0×10^{-10}	1	8
	6	32581008	rs3129747	T	C	0.698	-0.108	(0.018)	1.2×10^{-9}	0.989	8
	6	30434999	rs2516670	G	A	0.160	0.132	(0.022)	3.3×10^{-9}	1	2
	6	33045823	rs2073520	A	G	0.868	0.145	(0.025)	5.7×10^{-9}	0.972	1
	6	32707395	rs28371351	G	A	0.988	-0.519	(0.095)	4.2×10^{-8}	0.647	0
HHV7	6	32602665	rs139299944	C	CT	0.655	0.114	(0.017)	1.5×10^{-11}	0.998	437
HSV1	6	32627852	rs1130420	G	A	0.583	-0.122	(0.019)	2.5×10^{-10}	0.976	61
VZV	6	32623193	rs9273325	G	A	0.831	-0.232	(0.021)	8.2×10^{-28}	0.998	2708
	6	31323506	rs4990036	C	T	0.851	-0.240	(0.023)	4.5×10^{-26}	0.997	972
	6	32014828	rs433061	G	A	0.869	-0.249	(0.024)	6.5×10^{-26}	0.999	69
	6	30743729	rs3130668	G	A	0.855	-0.228	(0.023)	1.9×10^{-22}	0.978	271
	6	29986324	rs3115631	T	A	0.873	-0.229	(0.025)	4.1×10^{-20}	0.955	1442
	6	29356331	rs9257809	A	G	0.875	-0.216	(0.024)	1.5×10^{-18}	1	147
	6	28734676	rs1233604	G	A	0.882	-0.215	(0.025)	2.5×10^{-17}	1	291
	6	28214698	rs17720293	C	T	0.866	-0.191	(0.024)	2.1×10^{-15}	1	192
	6	27556141	rs13201294	A	T	0.870	-0.182	(0.024)	7.1×10^{-14}	0.996	94
	6	26743531	rs13211434	G	C	0.878	-0.188	(0.025)	1.1×10^{-13}	0.938	241
	6	26189356	rs13204572	G	C	0.896	-0.166	(0.027)	5.8×10^{-10}	0.996	37
	6	32589842	rs9271525	G	A	0.163	-0.137	(0.022)	1.0×10^{-9}	0.977	229
	6	30020252	rs546626159	A	ATT	0.749	0.108	(0.020)	3.0×10^{-8}	0.968	0
JCV	6	32589842	rs9271525	G	A	0.163	-0.318	(0.031)	3.9×10^{-24}	0.977	2335
	6	32080146	rs3130342	A	C	0.149	-0.245	(0.032)	1.3×10^{-14}	1	43
	6	31574306	rs372079459	T	TAA	0.198	-0.163	(0.028)	5.0×10^{-9}	0.972	2
MCV	6	32429277	rs9268847	A	G	0.750	-0.195	(0.022)	2.4×10^{-19}	0.995	2450
	6	32760665	rs113322198	C	CACTT	0.224	-0.196	(0.024)	2.3×10^{-16}	0.996	328
	6	32232358	rs9268070	T	C	0.149	-0.216	(0.029)	5.0×10^{-14}	0.999	38
	6	29924127	rs148584120	T	C	0.958	0.375	(0.052)	7.7×10^{-13}	0.972	160
	6	30475514	6:30475514	TGTGC	T	0.959	0.320	(0.052)	9.6×10^{-10}	0.966	10
	6	31561211	rs2736190	T	C	0.269	-0.134	(0.022)	2.1×10^{-9}	0.993	5
	6	27763714	rs76148407	C	G	0.966	0.299	(0.055)	4.8×10^{-8}	0.971	0

Table S7: Forty independent sentinel variants in the HLA region identified by clumping (LD r^2<0.05 ±500kb) associations with P<5×10^{-8} across 10 antigen response phenotypes.

Primary Phenotype	Chr	Position	Variant	Alleles		EAF	Beta	(SE)	P	INFO	Clump Size[1]
				Effect	Other						
EBV ZEBRA	6	32637772	rs9274728	G	A	0.718	-0.315	(0.018)	4.7×10^{-67}	0.995	14065
EBV EBNA	6	32633879	rs9274490	G	A	0.742	0.259	(0.019)	1.6×10^{-40}	0.997	4788
EBV EBNA	6	32608521	rs6907155	A	C	0.907	-0.317	(0.029)	2.9×10^{-27}	0.945	152
EBV p18	6	31486158	6:31486158	G	GT	0.245	0.197	(0.018)	7.1×10^{-27}	0.994	4087
VZV	6	31323506	rs4990036	T	C	0.851	-0.240	(0.023)	4.5×10^{-26}	0.997	1022
VZV	6	32014828	rs433061	A	G	0.869	-0.249	(0.024)	6.5×10^{-26}	0.999	323
VZV	6	30743729	rs3130668	A	G	0.855	-0.228	(0.023)	1.9×10^{-22}	0.978	509
EBV EBNA	6	32006317	rs6468	T	C	0.197	0.195	(0.021)	1.4×10^{-20}	0.963	353
VZV	6	29986324	rs3115631	A	T	0.873	-0.229	(0.025)	4.1×10^{-20}	0.955	1499
EBV ZEBRA	6	32045864	rs781341398	G	GAA	0.966	-0.428	(0.048)	5.1×10^{-19}	0.922	9
VZV	6	29356331	rs9257809	G	A	0.875	-0.216	(0.024)	1.5×10^{-18}	1	147
EBV p18	6	32786917	rs3763354	A	G	0.225	0.160	(0.018)	4.6×10^{-18}	1	687
VZV	6	28734676	rs1233604	A	G	0.882	-0.215	(0.025)	2.5×10^{-17}	1	291
EBV ZEBRA	6	32634588	rs113140854	T	C	0.983	-0.534	(0.067)	1.5×10^{-15}	0.827	257
VZV	6	28214698	rs17720293	T	C	0.866	-0.191	(0.024)	2.1×10^{-15}	1	193
EBV ZEBRA	6	31672242	rs28366157	G	A	0.937	-0.266	(0.034)	2.3×10^{-15}	0.997	197
EBV EBNA	6	33048628	rs1042136	C	A	0.838	0.176	(0.023)	1.3×10^{-14}	0.991	185
MCV	6	32630837	6:32630837	T	G	0.891	-0.233	(0.030)	1.5×10^{-14}	0.959	162
EBV EBNA	6	32551368	rs138008524	G	A	0.985	-0.625	(0.081)	1.9×10^{-14}	0.666	0
EBV ZEBRA	6	32635948	rs541213498	T	C	0.987	-0.586	(0.078)	4.8×10^{-14}	0.780	2
VZV	6	27556141	rs13201294	T	A	0.870	-0.182	(0.024)	7.1×10^{-14}	0.996	94
VZV	6	26743531	rs13211434	C	G	0.878	-0.188	(0.025)	1.1×10^{-13}	0.938	243
EBV ZEBRA	6	29796768	rs1632940	C	T	0.227	0.145	(0.020)	2.3×10^{-13}	0.991	268
EBV EBNA	6	31892641	rs9267677	C	T	0.905	0.206	(0.028)	3.6×10^{-13}	1	53
EBV p18	6	30921417	6:30921417	G	GA	0.119	0.165	(0.025)	2.7×10^{-11}	0.975	40
EBV EBNA	6	32598090	rs1281943	C	T	0.913	0.211	(0.032)	4.0×10^{-11}	0.919	16
EBV ZEBRA	6	31150844	rs138206303	AGT	A	0.883	-0.167	(0.025)	4.8×10^{-11}	0.975	31
MCV	6	30364587	rs2516687	T	A	0.960	0.341	(0.052)	5.3×10^{-11}	1	26
EBV EBNA	6	33083096	rs6457714	A	T	0.789	-0.132	(0.020)	6.8×10^{-11}	0.997	39
VZV	6	32853288	rs2244447	G	C	0.592	-0.106	(0.017)	2.9×10^{-10}	0.995	3
EBV EBNA	6	32965942	rs176248	A	G	0.707	0.115	(0.018)	3.2×10^{-10}	1	1
EBV p18	6	31379391	rs3828879	T	C	0.876	0.147	(0.024)	5.1×10^{-10}	1.000	0
VZV	6	26189356	rs13204572	C	G	0.896	-0.166	(0.027)	5.8×10^{-10}	0.996	37
EBV EA-D	6	32583876	rs117503706	A	G	0.986	-0.423	(0.072)	4.8×10^{-9}	0.912	15
EBV EBNA	6	32587835	rs71542419	A	C	0.916	-0.168	(0.029)	9.5×10^{-9}	0.973	6
EBV p18	6	31362207	rs1052409	C	T	0.845	0.125	(0.022)	1.2×10^{-8}	0.978	7
EBV EBNA	6	32722961	rs71565347	A	G	0.983	0.374	(0.067)	2.7×10^{-8}	0.991	3

EBV ZEBRA	6	32707395	rs28371351	A	G	0.988	-0.519	(0.095)	4.2×10^{-8}	0.647	0
MCV	6	27763714	rs76148407	G	C	0.966	0.299	(0.055)	4.8×10^{-8}	0.971	0
EBV EBNA	6	32542378	rs116206645	A	T	0.979	0.403	(0.074)	5.0×10^{-8}	0.699	0

[1] Clump size refers to the number of non-independent (LD r^2>0.05) variants clumped together with the sentinel variant

Table S8: Conditionally independent genome-wide significant (P_{cond}<5×10^{-8}) variants in the HLA identified through forward iterative conditional analyses for each antigen response phenotype.

Antigen	Round[1]	Chr	Position	Variant	Alleles		EAF	Beta	(SE)	P_{cond}	INFO
					Effect	Other					
EBV EBNA	0	6	32451762	rs9269233	A	C	0.249	0.315	(0.019)	3.5×10^{-61}	0.961
	1	6	32602665	rs139299944	C	CT	0.655	-0.160	(0.017)	5.9×10^{-21}	0.998
	2	6	33045272	rs6457711	C	A	0.752	-0.129	(0.019)	1.3×10^{-11}	0.994
	3	6	32626019	rs9273358	C	T	0.548	0.110	(0.017)	9.0×10^{-11}	0.996
	4	6	32626451	rs28414666	G	A	0.789	0.221	(0.035)	4.8×10^{-10}	0.997
	5	6	33047612	rs3097671	G	C	0.835	0.133	(0.022)	2.1×10^{-9}	1
EBV ZEBRA	0	6	32637772	rs9274728	A	G	0.718	-0.315	(0.018)	4.7×10^{-67}	0.995
	1	6	29923351	rs2904758	G	A	0.280	0.115	(0.018)	2.2×10^{-10}	0.990
	2	6	33034507	rs35683320	G	A	0.825	0.129	(0.022)	1.9×10^{-9}	0.982
	3	6	32783405	rs1383258	C	T	0.962	0.235	(0.042)	3.3×10^{-8}	1.000
EBV p18	0	6	31486158	6:31486158	GT	G	0.245	0.197	(0.018)	7.1×10^{-27}	0.994
	1	6	31247169	rs6917363	G	A	0.431	0.102	(0.016)	2.7×10^{-10}	0.999
	2	6	32582513	rs9271325	C	G	0.527	0.099	(0.016)	7.3×10^{-10}	1.000
	3	6	31356536	rs66479476	A	G	0.784	0.110	(0.020)	3.9×10^{-8}	0.963
MCV	0	6	32429277	rs9268847	A	G	0.750	-0.195	(0.022)	2.4×10^{-19}	0.995
	1	6	29924127	rs148584120	T	C	0.958	0.350	(0.052)	1.6×10^{-11}	0.972
	2	6	32797488	rs4148874	C	T	0.264	-0.158	(0.022)	1.1×10^{-12}	1
EBV EA-D	0	6	32665840	rs34825357	T	TC	0.409	-0.114	(0.017)	2.0×10^{-11}	0.998
	1	6	32655730	rs3129783	A	G	0.471	0.100	(0.017)	8.4×10^{-9}	0.980
CMV pp52	0	6	32301427	rs115378818	C	T	0.978	0.633	(0.095)	2.8×10^{-11}	0.731
HHV7	0	6	32602665	rs139299944	C	CT	0.655	0.114	(0.017)	1.5×10^{-11}	0.998
HSV1	0	6	32627852	rs1130420	G	A	0.583	-0.122	(0.019)	2.5×10^{-10}	0.976
VZV	0	6	32589842	rs9271525	G	A	0.831	-0.318	(0.031)	3.9×10^{-24}	0.977
JCV	0	6	32623193	rs9273325	G	A	0.163	-0.232	(0.021)	8.2×10^{-28}	0.998

[1] Conditional analysis round. Round 0 corresponds to results from unconditional analyses (not adjusted for any SNPs/indels).

Table S9: Associations from conditional analyses of classical HLA alleles associated with EBV ZEBRA antigen response at the Bonferroni-corrected threshold of $P<4.6\times10^{-5}$. Independent alleles identified at each round are in bold.

Allele	Unconditional (Round 0)			Conditional Round 1			Conditional Round 2			Conditional Round 3			Conditional Round 4			Conditional Round 5		
	Beta	(SE)	P	Beta	(SE)	P_{cond}	Beta	(S)E	P_{cond}	Beta	(SE)	P_{cond}	Beta	(SE)	P_{cond}	Beta	(SE)	P_{cond}
**DRB4*00:00**	**-0.246**	**(0.017)**	**1.4×10^{-46}**	-	-	-	-	-	-	-	-	-	-	-	-	-	-	-
**DQB1*04:02**	0.415	(0.056)	1.3×10^{-13}	**0.504**	**(0.055)**	**1.0×10^{-19}**	-	-	-	-	-	-	-	-	-	-	-	-
**DRB1*04:04**	0.508	(0.042)	6.8×10^{-34}	0.378	(0.043)	1.1×10^{-18}	**0.376**	**(0.042)**	**1.1×10^{-18}**	-	-	-	-	-	-	-	-	-
**DQA1*02:01**	0.254	(0.023)	4.9×10^{-27}	0.096	(0.028)	6.3×10^{-4}	0.098	(0.028)	4.4×10^{-4}	**0.187**	**(0.029)**	**1.1×10^{-10}**	-	-	-	-	-	-
**A*03:01**	0.107	(0.024)	6.2×10^{-6}	0.125	(0.023)	8.4×10^{-8}	0.128	(0.023)	3.2×10^{-8}	0.125	(0.023)	5.8×10^{-8}	**0.129**	**(0.023)**	**1.9×10^{-8}**	-	-	-
DRB3*00:00	0.202	(0.017)	7.4×10^{-32}	0.100	(0.020)	6.7×10^{-7}	0.067	(0.020)	9.5×10^{-4}	0.063	(0.020)	2.0×10^{-3}	0.060	(0.020)	2.7×10^{-3}	0.043	(0.020)	0.036
DRB1*07:01	0.251	(0.023)	1.3×10^{-26}	0.093	(0.028)	9.2×10^{-4}	0.094	(0.028)	7.1×10^{-4}	0.184	(0.029)	2.4×10^{-10}	-0.038	(0.185)	0.84	-0.050	(0.184)	0.79
DQB1*02:02	0.300	(0.030)	4.5×10^{-23}	0.128	(0.034)	1.5×10^{-4}	0.135	(0.034)	6.1×10^{-5}	0.217	(0.034)	2.8×10^{-10}	0.115	(0.053)	0.029	0.120	(0.053)	0.022
DRB4*01:01	0.293	(0.030)	7.9×10^{-22}	0.137	(0.033)	3.2×10^{-5}	0.139	(0.033)	2.3×10^{-5}	0.200	(0.033)	1.9×10^{-9}	0.114	(0.040)	4.7×10^{-3}	0.112	(0.040)	5.7×10^{-3}
DQB1*03:02	0.251	(0.027)	5.2×10^{-21}	0.103	(0.029)	4.4×10^{-4}	0.107	(0.029)	2.6×10^{-4}	-0.057	(0.035)	0.10	0.060	(0.040)	0.13	0.057	(0.040)	0.15
DRB4*01:03	0.173	(0.019)	7.0×10^{-20}	-0.142	(0.032)	1.1×10^{-5}	-0.144	(0.032)	8.1×10^{-6}	-0.194	(0.032)	2.2×10^{-9}	-0.112	(0.039)	3.9×10^{-3}	-0.110	(0.039)	4.7×10^{-3}
DQA1*05:01	-0.169	(0.019)	5.7×10^{-18}	-0.067	(0.021)	1.4×10^{-3}	-0.043	(0.021)	0.043	-0.039	(0.021)	0.066	-0.038	(0.021)	0.074	-0.019	(0.021)	0.36
DQA1*03:01	0.168	(0.020)	1.3×10^{-16}	-0.044	(0.027)	0.10	-0.047	(0.027)	0.080	-0.145	(0.028)	2.8×10^{-7}	0.153	(0.073)	0.036	0.148	(0.073)	0.043
DRB1*03:01	-0.172	(0.023)	5.1×10^{-14}	-0.077	(0.024)	1.2×10^{-3}	-0.058	(0.024)	0.015	-0.055	(0.024)	0.020	-0.055	(0.024)	0.019	-0.037	(0.024)	0.12
DQB1*02:01	-0.170	(0.023)	9.8×10^{-14}	-0.078	(0.024)	9.3×10^{-4}	-0.059	(0.024)	0.012	-0.057	(0.023)	0.016	-0.057	(0.023)	0.014	-0.039	(0.024)	0.097
DRB3*01:01	-0.154	(0.022)	1.3×10^{-12}	-0.054	(0.023)	0.018	-0.033	(0.023)	0.15	-0.031	(0.023)	0.17	-0.031	(0.023)	0.17	-0.014	(0.023)	0.52
DQA1*04:01	0.402	(0.057)	1.9×10^{-12}	0.506	(0.057)	4.2×10^{-19}	0.114	(0.232)	0.62	0.107	(0.231)	0.64	0.064	(0.230)	0.78	0.073	(0.230)	0.75
DRB1*08:01	0.409	(0.059)	3.9×10^{-12}	0.519	(0.058)	7.2×10^{-19}	0.118	(0.204)	0.56	0.125	(0.203)	0.54	0.093	(0.203)	0.65	0.088	(0.202)	0.66
B*08:01	-0.157	(0.023)	2.1×10^{-11}	-0.080	(0.024)	7.3×10^{-4}	-0.062	(0.024)	9.2×10^{-3}	-0.062	(0.024)	9.0×10^{-3}	-0.062	(0.024)	8.8×10^{-3}	-0.040	(0.024)	0.093
C*07:01	-0.135	(0.021)	2.6×10^{-10}	-0.072	(0.022)	8.0×10^{-4}	-0.057	(0.022)	7.9×10^{-3}	-0.055	(0.021)	9.9×10^{-3}	-0.053	(0.021)	0.013	-0.034	(0.022)	0.12
B*44:03	0.215	(0.034)	4.4×10^{-10}	0.101	(0.035)	3.8×10^{-3}	0.102	(0.035)	3.5×10^{-3}	0.140	(0.035)	6.1×10^{-5}	0.076	(0.037)	0.039	0.089	(0.037)	0.016
DPB1*06:01	0.550	(0.091)	1.7×10^{-9}	0.386	(0.091)	2.1×10^{-5}	0.379	(0.090)	2.7×10^{-5}	0.019	(0.101)	0.85	0.027	(0.101)	0.79	0.028	(0.101)	0.78
DRB3*02:02	-0.142	(0.024)	4.3×10^{-9}	-0.055	(0.025)	0.026	-0.034	(0.025)	0.17	-0.031	(0.025)	0.21	-0.029	(0.025)	0.24	-0.020	(0.025)	0.41
DQA1*01:02	-0.116	(0.021)	2.2×10^{-8}	-0.015	(0.022)	0.48	0.008	(0.022)	0.72	0.011	(0.022)	0.60	0.014	(0.022)	0.52	-0.002	(0.022)	0.92
C*16:01	0.219	(0.040)	3.0×10^{-8}	0.111	(0.040)	5.4×10^{-3}	0.109	(0.040)	6.1×10^{-3}	0.142	(0.040)	3.5×10^{-4}	0.082	(0.041)	0.045	0.092	(0.041)	0.024
A*29:02	0.209	(0.042)	6.6×10^{-7}	0.130	(0.042)	1.9×10^{-3}	0.127	(0.042)	2.4×10^{-3}	0.151	(0.042)	2.9×10^{-4}	0.109	(0.042)	9.5×10^{-3}	0.126	(0.042)	2.8×10^{-3}

DRB3*03:01	-0.181	(0.042)	1.5×10^{-5}	-0.092	(0.042)	0.028	-0.073	(0.042)	0.079	-0.069	(0.041)	0.097	-0.066	(0.041)	0.11	-0.069	(0.041)	0.096
DRB1*13:02	-0.178	(0.042)	2.0×10^{-5}	-0.090	(0.042)	0.032	-0.070	(0.042)	0.091	-0.066	(0.041)	0.11	-0.063	(0.041)	0.13	-0.065	(0.041)	0.11
B*40:01	0.150	(0.036)	3.7×10^{-5}	0.109	(0.036)	2.5×10^{-3}	0.092	(0.036)	0.010	0.011	(0.037)	0.77	0.024	(0.037)	0.52	0.031	(0.037)	0.39

Table S10: Associations from conditional analyses of classical HLA alleles associated with EBV EBNA antigen response at the Bonferroni-corrected threshold of $P<4.6\times10^{-5}$. Independent alleles identified at each round are in bold.

Allele	Unconditional (Round 0)			Conditional Round 1			Conditional Round 2			Conditional Round 3			Conditional Round 4			Conditional Round 5		
	Beta	SE	P	Beta	SE	P_{cond}	Beta	SE	P_{cond}	Beta	SE	P_{cond}	Beta	SE	P_{cond}	Beta	SE	P_{cond}
**DRB5*00:00**	**-0.255**	**(0.022)**	**8.7×10^{-30}**	-	-	-	-	-	-	-	-	-	-	-	-	-	-	-
**DRB3*02:02**	0.224	(0.024)	2.2×10^{-20}	**0.276**	**(0.024)**	**6.8×10^{-30}**	-	-	-	-	-	-	-	-	-	-	-	-
**DQB1*02:01**	-0.217	(0.023)	2.4×10^{-20}	-0.174	(0.024)	2.0×10^{-13}	**-0.164**	**(0.023)**	**3.6×10^{-12}**	-	-	-	-	-	-	-	-	-
**DRB4*00:00**	0.198	(0.018)	6.6×10^{-29}	0.150	(0.018)	3.8×10^{-16}	0.092	(0.019)	2.1×10^{-6}	**0.176**	**(0.021)**	**8.3×10^{-17}**	-	-	-	-	-	-
**DPB1*03:01**	-0.215	(0.030)	5.7×10^{-13}	-0.199	(0.030)	1.8×10^{-11}	-0.201	(0.029)	6.9×10^{-12}	-0.206	(0.029)	1.7×10^{-12}	**-0.220**	**(0.029)**	**4.7×10^{-14}**	-	-	-
DRB1*15:01	0.253	(0.023)	7.8×10^{-28}	0.025	(0.079)	0.75	0.052	(0.079)	0.51	0.048	(0.078)	0.54	0.027	(0.078)	0.73	0.025	(0.078)	0.75
DQA1*01:02	0.221	(0.020)	4.1×10^{-27}	0.085	(0.039)	0.027	0.138	(0.038)	3.3×10^{-4}	0.110	(0.039)	4.5×10^{-3}	0.018	(0.040)	0.65	0.046	(0.040)	0.26
DRB5*01:01	0.248	(0.023)	5.2×10^{-27}	-0.049	(0.085)	0.57	-0.016	(0.085)	0.85	-0.016	(0.084)	0.85	-0.036	(0.084)	0.66	-0.036	(0.084)	0.67
DQB1*06:02	0.244	(0.023)	8.5×10^{-26}	-0.024	(0.067)	0.72	0.006	(0.066)	0.92	0.001	(0.066)	0.99	-0.028	(0.066)	0.67	-0.037	(0.065)	0.57
DQA1*02:01	-0.231	(0.024)	3.1×10^{-21}	-0.188	(0.025)	1.9×10^{-14}	-0.142	(0.025)	9.1×10^{-9}	-0.190	(0.025)	6.3×10^{-14}	-0.113	(0.029)	9.2×10^{-5}	-0.117	(0.029)	4.9×10^{-5}
DRB1*07:01	-0.230	(0.024)	3.8×10^{-21}	-0.188	(0.024)	1.9×10^{-14}	-0.142	(0.025)	9.8×10^{-9}	-0.189	(0.025)	6.4×10^{-14}	-0.113	(0.029)	9.1×10^{-5}	-0.117	(0.029)	4.7×10^{-5}
DRB1*03:01	-0.211	(0.023)	3.4×10^{-19}	-0.168	(0.024)	1.4×10^{-12}	-0.157	(0.024)	2.8×10^{-11}	0.119	(0.127)	0.35	0.003	(0.127)	0.98	-0.005	(0.126)	0.97
DRB4*01:03	-0.171	(0.020)	5.0×10^{-18}	-0.123	(0.020)	1.4×10^{-9}	-0.067	(0.021)	1.3×10^{-3}	-0.125	(0.022)	1.0×10^{-8}	0.029	(0.033)	0.38	0.035	(0.033)	0.29
DRB1*12:01	0.578	(0.071)	3.5×10^{-16}	0.617	(0.070)	1.6×10^{-18}	0.422	(0.073)	7.1×10^{-9}	0.393	(0.073)	6.9×10^{-8}	0.365	(0.072)	4.8×10^{-7}	0.360	(0.072)	6.4×10^{-7}
DQB1*02:02	-0.242	(0.031)	1.5×10^{-14}	-0.194	(0.032)	7.5×10^{-10}	-0.144	(0.032)	5.1×10^{-6}	-0.196	(0.032)	1.1×10^{-9}	-0.105	(0.035)	2.6×10^{-3}	-0.106	(0.035)	2.4×10^{-3}
B*07:02	0.162	(0.023)	5.4×10^{-12}	0.038	(0.027)	0.16	0.038	(0.027)	0.16	0.027	(0.027)	0.31	0.020	(0.027)	0.46	0.023	(0.026)	0.38
C*07:02	0.144	(0.023)	2.1×10^{-10}	0.030	(0.026)	0.24	0.031	(0.025)	0.23	0.020	(0.025)	0.43	0.007	(0.025)	0.77	0.012	(0.025)	0.62
DPB1*04:02	0.165	(0.027)	1.3×10^{-9}	0.187	(0.027)	4.2×10^{-12}	0.167	(0.027)	4.4×10^{-10}	0.159	(0.027)	2.7×10^{-9}	0.140	(0.027)	1.7×10^{-7}	0.116	(0.027)	1.6×10^{-5}
DRB1*11:01	0.264	(0.046)	8.1×10^{-9}	0.312	(0.045)	6.9×10^{-12}	0.082	(0.052)	0.11	0.051	(0.052)	0.32	0.034	(0.051)	0.51	0.024	(0.051)	0.64
B*08:01	-0.136	(0.024)	1.5×10^{-8}	-0.105	(0.024)	1.1×10^{-5}	-0.066	(0.024)	6.3×10^{-3}	0.112	(0.034)	1.1×10^{-3}	0.082	(0.034)	0.017	0.076	(0.034)	0.027
DQA1*03:01	-0.117	(0.021)	4.5×10^{-8}	-0.067	(0.022)	1.9×10^{-3}	-0.010	(0.022)	0.65	-0.054	(0.023)	0.018	0.084	(0.028)	2.6×10^{-3}	0.085	(0.028)	2.3×10^{-3}
A*03:01	0.130	(0.024)	4.6×10^{-8}	0.092	(0.024)	1.1×10^{-4}	0.100	(0.024)	2.5×10^{-5}	0.085	(0.024)	3.1×10^{-4}	0.073	(0.024)	2.1×10^{-3}	0.075	(0.023)	1.4×10^{-3}
DRB4*01:01	-0.168	(0.032)	9.4×10^{-8}	-0.126	(0.032)	6.9×10^{-5}	-0.078	(0.032)	0.014	-0.117	(0.032)	2.4×10^{-4}	-0.025	(0.034)	0.46	-0.032	(0.034)	0.35
DRB1*14:01	0.291	(0.060)	1.2×10^{-6}	0.315	(0.059)	1.2×10^{-7}	0.120	(0.062)	0.053	0.097	(0.062)	0.118	0.075	(0.062)	0.22	0.090	(0.061)	0.14
DRB3*01:01	-0.107	(0.022)	1.3×10^{-6}	-0.060	(0.022)	7.1×10^{-3}	-0.003	(0.023)	0.90	0.279	(0.035)	1.5×10^{-15}	0.197	(0.038)	1.6×10^{-7}	0.187	(0.037)	5.8×10^{-7}
DQB1*06:03	0.167	(0.036)	4.3×10^{-6}	0.207	(0.036)	9.0×10^{-9}	0.140	(0.036)	1.1×10^{-4}	0.112	(0.036)	2.1×10^{-3}	0.047	(0.037)	0.21	0.048	(0.037)	0.19

DRB1*04:01	-0.122	(0.027)	9.3×10^{-6}	-0.074	(0.028)	7.4×10^{-3}	-0.022	(0.028)	0.44	-0.061	(0.028)	0.031	0.045	(0.031)	0.15	0.035	(0.031)	0.26
DQB1*05:03	0.243	(0.055)	1.1×10^{-5}	0.266	(0.055)	1.3×10^{-6}	0.078	(0.057)	0.17	0.053	(0.057)	0.35	0.034	(0.057)	0.55	0.047	(0.057)	0.41
C*07:01	-0.094	(0.022)	1.7×10^{-5}	-0.065	(0.022)	3.0×10^{-3}	-0.039	(0.022)	0.07	0.086	(0.027)	1.7×10^{-3}	0.066	(0.027)	0.015	0.064	(0.027)	0.020
DPB1*05:01	0.243	(0.058)	2.9×10^{-5}	0.226	(0.057)	8.6×10^{-5}	0.219	(0.057)	1.2×10^{-4}	0.201	(0.057)	4.2×10^{-4}	0.169	(0.057)	2.8×10^{-3}	0.148	(0.057)	8.8×10^{-3}
DRB1*13:01	0.150	(0.037)	4.3×10^{-5}	0.198	(0.036)	5.6×10^{-8}	0.133	(0.037)	3.0×10^{-4}	0.104	(0.037)	4.7×10^{-3}	0.037	(0.038)	0.33	0.038	(0.038)	0.31
B*51:01	0.183	(0.045)	4.6×10^{-5}	0.194	(0.044)	1.2×10^{-5}	0.164	(0.044)	2.0×10^{-4}	0.144	(0.044)	1.1×10^{-3}	0.135	(0.044)	2.1×10^{-3}	0.144	(0.044)	1.0×10^{-3}

Table S11: Associations from conditional analyses of classical HLA alleles associated with EBV p18 antigen response at the Bonferroni-corrected threshold of $P<4.6\times10^{-5}$. Independent alleles identified at each round are in bold.

Allele	Unconditional (Round 0)			Conditional Round 1			Conditional Round 2		
	Beta	(SE)	P	Beta	(SE)	P_{cond}	Beta	(SE)	P_{cond}
**DRB5*00:00**	**-0.210**	**(0.021)**	**1.7×10^{-22}**	-	-	-	-	-	-
**DRB1*04:04**	0.319	(0.041)	4.7×10^{-15}	**0.357**	**(0.040)**	**1.3×10^{-18}**	-	-	-
DRB5*01:01	0.211	(0.022)	1.4×10^{-21}	0.041	(0.083)	0.62	0.042	(0.082)	0.61
DRB1*15:01	0.209	(0.022)	4.2×10^{-21}	0.022	(0.077)	0.78	0.026	(0.077)	0.73
DQA1*01:02	0.181	(0.020)	2.7×10^{-20}	0.067	(0.037)	0.069	0.085	(0.037)	0.020
C*07:02	0.196	(0.022)	9.7×10^{-20}	0.125	(0.024)	2.9×10^{-7}	0.117	(0.024)	1.4×10^{-6}
DQB1*06:02	0.199	(0.022)	4.7×10^{-19}	-0.043	(0.064)	0.50	-0.039	(0.064)	0.54
B*07:02	0.195	(0.022)	2.2×10^{-18}	0.115	(0.026)	8.1×10^{-6}	0.106	(0.026)	3.3×10^{-5}
B*08:01	-0.119	(0.022)	8.3×10^{-8}	-0.094	(0.022)	2.4×10^{-5}	-0.079	(0.022)	3.6×10^{-4}
DPB1*03:01	-0.143	(0.028)	2.1×10^{-7}	-0.129	(0.027)	2.9×10^{-6}	-0.126	(0.027)	4.0×10^{-6}
DQB1*02:01	-0.105	(0.022)	1.3×10^{-6}	-0.069	(0.022)	1.7×10^{-3}	-0.047	(0.022)	0.031
DRB1*03:01	-0.102	(0.022)	2.2×10^{-6}	-0.067	(0.022)	2.2×10^{-3}	-0.045	(0.022)	0.040
DPB1*04:01	0.076	(0.016)	2.5×10^{-6}	0.046	(0.016)	5.0×10^{-3}	0.052	(0.016)	1.5×10^{-3}
C*07:01	-0.093	(0.020)	4.0×10^{-6}	-0.070	(0.020)	5.8×10^{-4}	-0.056	(0.020)	5.7×10^{-3}
DRB3*00:00	0.075	(0.016)	5.5×10^{-6}	0.031	(0.017)	0.074	0.001	(0.017)	0.95
DQA1*05:01	-0.084	(0.019)	6.0×10^{-6}	-0.045	(0.019)	0.017	-0.020	(0.019)	0.29
B*14:02	-0.212	(0.048)	8.8×10^{-6}	-0.199	(0.047)	2.7×10^{-5}	-0.183	(0.047)	1.1×10^{-4}
C*08:02	-0.181	(0.041)	9.2×10^{-6}	-0.165	(0.041)	4.8×10^{-5}	-0.149	(0.040)	2.2×10^{-4}
DPB1*06:01	0.379	(0.089)	2.0×10^{-5}	0.424	(0.088)	1.7×10^{-6}	0.077	(0.100)	0.44
DQA1*02:01	-0.096	(0.023)	2.5×10^{-5}	-0.059	(0.023)	0.010	-0.038	(0.023)	0.099
DRB1*07:01	-0.095	(0.023)	3.1×10^{-5}	-0.058	(0.023)	0.011	-0.037	(0.023)	0.11
C*04:01	-0.117	(0.028)	4.1×10^{-5}	-0.093	(0.028)	1.1×10^{-3}	-0.081	(0.028)	4.0×10^{-3}
C*03:03	-0.139	(0.034)	4.2×10^{-5}	-0.130	(0.034)	1.1×10^{-4}	-0.142	(0.034)	2.2×10^{-5}

Table S12: Associations from conditional analyses of classical HLA alleles associated with antigen response at the Bonferroni-corrected threshold of $P<4.6\times10^{-5}$. Independent alleles identified at each round are in bold. Results are presented for antigens with a single conditionally independent classical allele.

Antigen	Allele	Unconditional (Round 0)			Conditional Round 1		
		Beta	(SE)	P	Beta	SE	P_{cond}
CMV pp52	**DRB1*01:03**	**-0.538**	**(0.075)**	**9.4×10^{-13}**	-	-	-
	C*07:01	-0.091	(0.025)	2.7×10^{-4}	-0.099	(0.025)	6.4×10^{-5}
	DPB1*03:01	0.121	(0.034)	3.7×10^{-4}	0.112	(0.034)	8.9×10^{-4}
EBV EA-D	**DQB1*02:01**	**-0.154**	**(0.024)**	**8.4×10^{-11}**	-	-	-
	DRB1*03:01	-0.147	(0.024)	5.5×10^{-10}	0.109	(0.124)	0.38
	B*08:01	-0.144	(0.024)	2.7×10^{-9}	-0.064	(0.035)	0.068
	DRB1*09:01	0.407	(0.073)	3.2×10^{-8}	0.383	(0.073)	1.9×10^{-7}
	DQA1*03:01	0.110	(0.021)	1.1×10^{-7}	0.085	(0.021)	6.6×10^{-5}
	DRB4*00:00	-0.088	(0.018)	5.8×10^{-7}	-0.058	(0.018)	1.5×10^{-3}
	C*07:01	-0.109	(0.022)	7.7×10^{-7}	-0.032	(0.028)	0.25
	DRB3*00:00	0.087	(0.018)	9.0×10^{-7}	0.033	(0.021)	0.12
	A*03:01	0.114	(0.024)	2.4×10^{-6}	0.098	(0.024)	5.6×10^{-5}
	DRB4*01:03	0.085	(0.019)	1.1×10^{-5}	0.057	(0.020)	4.4×10^{-3}
	DQA1*05:01	-0.088	(0.020)	1.4×10^{-5}	0.026	(0.031)	0.40
	DQB1*03:03	0.164	(0.038)	1.4×10^{-5}	0.142	(0.038)	1.9×10^{-4}
	DRB3*01:01	-0.096	(0.022)	2.0×10^{-5}	0.035	(0.034)	0.32
	DQB1*03:01	0.094	(0.022)	2.1×10^{-5}	0.069	(0.023)	2.2×10^{-3}
HHV7	**DQB1*05:01**	**-0.139**	**(0.025)**	**2.8×10^{-8}**	-	-	-
	DQA1*01:01	-0.125	(0.023)	8.2×10^{-8}	-0.045	(0.053)	0.40
	DRB1*01:01	-0.146	(0.028)	2.3×10^{-7}	-0.049	(0.052)	0.35
	DRB4*00:00	-0.085	(0.017)	4.5×10^{-7}	-0.066	(0.017)	1.6×10^{-4}
	DPB1*04:02	-0.133	(0.026)	4.8×10^{-7}	-0.115	(0.027)	1.5×10^{-5}
	DRB1*04:01	0.128	(0.026)	7.3×10^{-7}	0.110	(0.026)	2.3×10^{-5}
	DRB4*01:03	0.077	(0.019)	3.0×10^{-5}	0.058	(0.019)	2.0×10^{-3}
HSV1	**DQB1*02:01**	**0.145**	**(0.026)**	**2.1×10^{-8}**	-	-	-
	DRB1*03:01	0.140	(0.026)	6.3×10^{-8}	-0.090	(0.147)	0.54
	DQA1*01:02	-0.110	(0.024)	3.1×10^{-6}	-0.086	(0.024)	3.7×10^{-4}
	DQB1*06:02	-0.115	(0.027)	1.7×10^{-5}	-0.091	(0.027)	7.9×10^{-4}
	DRB5*00:00	0.110	(0.026)	2.3×10^{-5}	0.086	(0.026)	1.2×10^{-3}
	DRB1*15:01	-0.110	(0.027)	3.6×10^{-5}	-0.086	(0.027)	1.5×10^{-3}
	DRB3*01:01	0.102	(0.025)	3.6×10^{-5}	-0.011	(0.038)	0.78
	DRB5*01:01	-0.109	(0.026)	3.7×10^{-5}	-0.085	(0.027)	1.5×10^{-3}
VZV	**DRB1*03:01**	**0.236**	**(0.022)**	**7.3×10^{-26}**	-	-	-
	DQB1*02:01	0.236	(0.022)	7.5×10^{-26}	0.117	(0.126)	0.35
	B*08:01	0.238	(0.023)	4.7×10^{-25}	0.131	(0.034)	1.2×10^{-4}

	DRB3*01:01	0.197	(0.021)	3.7×10^{-20}	0.053	(0.034)	0.12
	C*07:01	0.194	(0.021)	4.6×10^{-20}	0.088	(0.027)	1.4×10^{-3}
	A*01:01	0.181	(0.021)	1.9×10^{-18}	0.104	(0.023)	6.9×10^{-6}
	DQA1*05:01	0.153	(0.019)	2.8×10^{-15}	-0.013	(0.031)	0.67
	DRB3*00:00	-0.114	(0.017)	3.2×10^{-11}	-0.013	(0.021)	0.54
	DRB5*00:00	0.136	(0.023)	2.3×10^{-9}	0.096	(0.023)	3.1×10^{-5}
	DRB5*01:01	-0.135	(0.023)	7.1×10^{-9}	-0.095	(0.023)	5.1×10^{-5}
	DQB1*06:02	-0.133	(0.023)	1.6×10^{-8}	-0.095	(0.024)	5.4×10^{-5}
	DRB1*15:01	-0.132	(0.023)	1.8×10^{-8}	-0.092	(0.024)	1.0×10^{-4}
	A*02:01	-0.103	(0.019)	4.1×10^{-8}	-0.079	(0.019)	3.0×10^{-5}
	DQA1*01:02	-0.099	(0.021)	1.4×10^{-6}	-0.057	(0.021)	6.7×10^{-3}
	**DRB5*00:00**	**0.305**	**(0.032)**	**1.2×10^{-21}**	-	-	-
	DRB5*01:01	-0.309	(0.033)	4.8×10^{-21}	-0.097	(0.120)	0.42
	DQB1*06:02	-0.310	(0.033)	7.2×10^{-21}	-0.116	(0.093)	0.21
JCV	DRB1*15:01	-0.309	(0.033)	9.0×10^{-21}	-0.082	(0.112)	0.46
	DQA1*01:02	-0.218	(0.028)	8.5×10^{-15}	0.006	(0.049)	0.89
	DQB1*03:01	0.133	(0.027)	1.1×10^{-6}	0.086	(0.028)	1.7×10^{-3}
	B*07:02	-0.132	(0.031)	2.8×10^{-5}	0.012	(0.035)	0.73

Table S13: Associations from conditional analyses of classical HLA alleles associated with MCV antigen response at the Bonferroni-corrected threshold of $P<4.6\times10^{-5}$. Independent alleles identified at each round are in bold.

Allele	Unconditional (Round 0)			Conditional Round 1			Conditional Round 2			Conditional Round 3			Conditional Round 4		
	Beta	(SE)	P	Beta	(SE)	P_{cond}	Beta	(SE)	P_{cond}	Beta	(SE)	P_{cond}	Beta	(SE)	P_{cond}
**DQA1*01:01**	**0.215**	**(0.027)**	**1.1×10^{-15}**	-	-	-	-	-	-	-	-	-	-	-	-
**DRB1*04:04**	-0.390	(0.054)	8.6×10^{-13}	**-0.362**	**(0.054)**	**3.0×10^{-11}**	-	-	-	-	-	-	-	-	-
**A*29:02**	-0.359	(0.052)	4.5×10^{-12}	-0.342	(0.052)	3.7×10^{-11}	**-0.350**	**(0.051)**	**1.0×10^{-11}**	-	-	-	-	-	-
**DRB1*15:01**	-0.213	(0.029)	2.1×10^{-13}	-0.178	(0.029)	1.1×10^{-9}	-0.199	(0.029)	1.0×10^{-11}	**-0.203**	**(0.029)**	**3.7×10^{-12}**	-	-	-
DQB1*05:01	0.220	(0.029)	1.6×10^{-14}	0.070	(0.066)	0.29	0.070	(0.066)	0.29	0.070	(0.065)	0.29	0.066	(0.065)	0.31
DQB1*06:02	-0.213	(0.029)	2.5×10^{-13}	-0.179	(0.029)	1.2×10^{-9}	-0.199	(0.029)	1.2×10^{-11}	-0.204	(0.029)	3.7×10^{-12}	-0.103	(0.124)	0.41
DRB5*01:01	-0.210	(0.029)	3.7×10^{-13}	-0.176	(0.029)	1.8×10^{-9}	-0.196	(0.029)	1.9×10^{-11}	-0.200	(0.029)	7.2×10^{-12}	0.067	(0.227)	0.77
DRB1*01:01	0.216	(0.032)	1.3×10^{-11}	0.046	(0.050)	0.35	0.044	(0.050)	0.38	0.044	(0.050)	0.37	0.041	(0.049)	0.41
DRB5*00:00	0.185	(0.028)	4.8×10^{-11}	0.152	(0.028)	7.4×10^{-8}	0.173	(0.028)	1.1×10^{-9}	0.177	(0.028)	3.8×10^{-10}	-0.094	(0.089)	0.29
DQA1*01:02	-0.151	(0.025)	1.9×10^{-9}	-0.113	(0.026)	9.8×10^{-6}	-0.136	(0.026)	1.1×10^{-7}	-0.143	(0.026)	2.5×10^{-8}	0.004	(0.044)	0.93
DPB1*06:01	-0.591	(0.122)	1.2×10^{-6}	-0.556	(0.121)	4.4×10^{-6}	-0.228	(0.137)	0.095	-0.208	(0.136)	0.13	-0.217	(0.136)	0.11
DRB3*02:02	0.129	(0.028)	3.0×10^{-6}	0.131	(0.028)	1.9×10^{-6}	0.118	(0.027)	1.9×10^{-5}	0.109	(0.027)	6.8×10^{-5}	0.081	(0.028)	3.4×10^{-3}
DQB1*03:01	0.108	(0.025)	1.9×10^{-5}	0.153	(0.026)	2.1×10^{-9}	0.139	(0.026)	6.0×10^{-8}	0.131	(0.025)	2.7×10^{-7}	0.096	(0.026)	2.3×10^{-4}
C*07:02	-0.118	(0.028)	2.7×10^{-5}	-0.116	(0.028)	3.0×10^{-5}	-0.117	(0.028)	2.5×10^{-5}	-0.128	(0.028)	3.7×10^{-6}	-0.051	(0.031)	0.096
B*07:02	-0.121	(0.029)	3.4×10^{-5}	-0.116	(0.029)	6.3×10^{-5}	-0.118	(0.029)	4.4×10^{-5}	-0.127	(0.029)	1.0×10^{-5}	-0.039	(0.033)	0.23
C*16:01	-0.178	(0.048)	2.1×10^{-4}	-0.154	(0.048)	1.3×10^{-3}	-0.164	(0.048)	5.7×10^{-4}	0.096	(0.064)	0.13	0.073	(0.064)	0.25
DQB1*03:02	-0.114	(0.033)	4.8×10^{-4}	-0.080	(0.033)	0.015	0.064	(0.040)	0.11	0.051	(0.040)	0.20	0.024	(0.040)	0.55

Table S14: Associations for HLA amino acid (AA) sequence substitutions that were significantly associated (P<4.6×10^{-5}) with seroreactivity to EBV p18. At each position that contained a significantly associated allele and >2 possible substitutions, we tested all common (frequency ≥0.01) haplotypes at that position using the omnibus test (P_{omni}) in the conditional haplotype module of PLINK 1.07. Variants and haplotypes with the lowest p-value are indicated in bold and shaded.

Gene	AA position	AA	A1	A2	FRQ	BETA	SE	P	P_{omni}	INFO
B	9	D	P	A	0.146	-0.117	0.022	1.23E-07		0.999
B	11		S	A	0.306	0.110	0.017	2.88E-10		0.931
B	12		V	M	0.321	0.097	0.017	7.39E-09		0.997
B	24	A	P	A	0.251	-0.087	0.019	2.90E-06		0.964
B	45	MG	P	A	0.128	-0.104	0.024	1.61E-05		0.949
B	45	M	P	A	0.126	-0.103	0.024	2.15E-05		0.962
B	46		A	E	0.126	-0.103	0.024	2.15E-05		0.962
B	67	Y	P	A	0.170	0.154	0.022	9.30E-13		0.939
B	67	YS	P	A	0.573	0.109	0.016	1.66E-11		0.932
B	67	FC	P	A	0.371	-0.099	0.017	6.77E-09		0.875
B	67	FM	P	A	0.301	-0.100	0.017	9.02E-09	5.80E-14	0.950
B	67	YM	P	A	0.225	0.104	0.020	2.55E-07		0.859
B	67	F	P	A	0.246	-0.094	0.019	9.50E-07		0.881
B	67	YC	P	A	0.295	0.078	0.018	1.01E-05		0.933
B	69	A	P	A	0.279	0.091	0.018	8.00E-07		0.883
B	69	T	P	A	0.718	-0.091	0.019	1.09E-06		0.871
B	70	Q	P	A	0.169	0.154	0.021	8.43E-13		0.944
B	70	QK	P	A	0.226	0.126	0.019	7.04E-11	9.52E-12	0.943
B	70	QS	P	A	0.225	0.104	0.020	2.38E-07		0.863
B	70	N	P	A	0.718	-0.091	0.019	1.09E-06		0.871
B	71		A	T	0.282	0.091	0.019	1.08E-06		0.871
B	103	VM	P	A	0.834	0.134	0.030	9.74E-06		0.407
B	103	V	P	A	0.833	0.133	0.030	1.13E-05		0.408
B	116	S	P	A	0.175	-0.175	0.027	1.05E-10		0.539
B	116	Sx	P	A	0.194	-0.154	0.024	1.25E-10		0.640
B	116	YFD	P	A	0.732	0.152	0.024	3.68E-10		0.471
B	116	LS	P	A	0.249	-0.167	0.027	6.15E-10	2.37E-08	0.388
B	116	YD	P	A	0.632	0.116	0.019	1.85E-09		0.673
B	116	YDL	P	A	0.706	0.108	0.018	4.02E-09		0.863
B	116	YDx	P	A	0.650	0.117	0.020	1.04E-08		0.597
B	116	FS	P	A	0.275	-0.110	0.019	1.76E-08		0.780
B	152	E	P	A	0.292	0.177	0.024	1.22E-13		0.484

B	152	V	P	A	0.689	-0.134	0.022	8.79E-10		0.559
B	156	R	P	A	0.141	0.200	0.027	4.84E-14		0.710
B	156	Rx	P	A	0.160	0.180	0.027	1.51E-11		0.604
B	156	RW	P	A	0.184	0.139	0.025	3.16E-08	3.22E-12	0.623
B	156	DL	P	A	0.797	-0.100	0.023	1.32E-05		0.715
B	156	RL	P	A	0.670	0.086	0.020	2.10E-05		0.579
B	163	E	P	A	0.287	0.130	0.019	4.83E-12		0.824
B	163	Ex	P	A	0.305	0.123	0.019	1.08E-10	9.76E-12	0.747
B	163	EL	P	A	0.701	0.113	0.019	1.81E-09		0.804
B	163	T	P	A	0.280	-0.099	0.018	8.06E-08		0.881
B	178	K	P	A	0.210	0.165	0.021	5.06E-15		0.800
B	178	T	P	A	0.771	-0.155	0.021	3.48E-13		0.712
C	-15	L	P	A	0.419	0.072	0.016	8.83E-06		1.000
C	-15	I	P	A	0.580	-0.071	0.016	9.26E-06		0.997
C	1	C	P	A	0.774	0.094	0.019	5.42E-07		1.000
C	1	G	P	A	0.226	-0.094	0.019	5.57E-07		0.997
C	14		W	R	0.085	-0.116	0.028	4.37E-05		1.000
C	49		E	A	0.085	-0.116	0.028	4.37E-05		0.999
C	99	S	P	A	0.157	0.192	0.021	4.70E-19		0.998
C	99	YF	P	A	0.808	-0.171	0.020	7.91E-18	6.01E-19	0.998
C	99	Y	P	A	0.714	-0.088	0.017	3.12E-07		0.998
C	99	YC	P	A	0.749	-0.090	0.018	5.32E-07		0.998
C	116	SL	P	A	0.585	0.069	0.017	3.03E-05		0.929
C	219	W	P	A	0.264	-0.080	0.018	8.01E-06		0.998
C	219	R	P	A	0.735	0.079	0.018	8.53E-06		0.995
C	303	M	P	A	0.084	-0.118	0.029	3.70E-05		0.987
DPB1	8		V	L	0.280	-0.094	0.017	7.50E-08		0.998
DPB1	9	F	P	A	0.720	0.094	0.017	7.50E-08		0.998
DPB1	9	Y	P	A	0.237	-0.076	0.018	3.39E-05		0.995
DPB1	11		L	G	0.216	-0.078	0.019	4.37E-05		0.994
DPB1	55	D	P	A	0.402	-0.068	0.017	3.82E-05		0.921
DPB1	56		E	A	0.402	-0.068	0.017	3.82E-05		0.921
DPB1	76	V	P	A	0.201	-0.131	0.021	2.61E-10		0.881
DPB1	76	M	P	A	0.779	0.119	0.020	2.52E-09		0.888
DPB1	84	D	P	A	0.307	-0.084	0.017	7.58E-07		0.997
DPB1	84	G	P	A	0.686	0.077	0.017	4.74E-06		0.997
DPB1	85		E	G	0.307	-0.084	0.017	7.64E-07		0.997
DPB1	86		P	A	0.693	0.084	0.017	7.65E-07		0.997
DPB1	87		V	M	0.307	-0.084	0.017	7.62E-07		0.997
DPB1	96	K	P	A	0.302	-0.071	0.017	3.34E-05		0.991

DPB1	96	R	P	A	0.696	0.070	0.017	3.60E-05		0.996
DPB1	170	I	P	A	0.302	-0.071	0.017	3.35E-05		0.991
DPB1	170	T	P	A	0.696	0.071	0.017	3.57E-05		0.996
DPB1	194	R	P	A	0.857	0.106	0.025	1.60E-05		0.782
DQA1	-16	M	P	A	0.580	-0.098	0.016	8.92E-10		0.998
DQA1	-16	L	P	A	0.420	0.097	0.016	1.04E-09		0.999
DQA1	11	C	P	A	0.396	0.087	0.016	6.25E-08		0.999
DQA1	11	Y	P	A	0.604	-0.087	0.016	6.25E-08		0.999
DQA1	18		F	S	0.396	0.087	0.016	6.25E-08		0.999
DQA1	41		K	R	0.171	0.148	0.023	5.90E-11		0.816
DQA1	45		A	V	0.396	0.087	0.016	6.30E-08		0.999
DQA1	47	RQ	P	A	0.600	0.100	0.016	6.26E-10		0.986
DQA1	47	R	P	A	0.395	0.087	0.016	6.48E-08	2.05E-09	0.997
DQA1	47	K	P	A	0.136	-0.096	0.023	3.14E-05		0.979
DQA1	48		W	L	0.396	0.087	0.016	6.20E-08		0.999
DQA1	50	E	P	A	0.396	0.087	0.016	6.37E-08		0.998
DQA1	52	S	P	A	0.396	0.087	0.016	6.31E-08		0.999
DQA1	52	H	P	A	0.136	-0.096	0.023	3.14E-05		0.979
DQA1	53	K	P	A	0.396	0.087	0.016	6.20E-08		0.999
DQA1	54		L	F	0.136	-0.096	0.023	3.14E-05		0.979
DQA1	55		G	R	0.396	0.087	0.016	6.36E-08		0.998
DQA1	56	x	P	A	0.400	-0.100	0.016	6.42E-10		0.985
DQA1	56	G	P	A	0.396	0.087	0.016	6.36E-08		0.998
DQA1	61		G	F	0.396	0.087	0.016	6.20E-08		0.999
DQA1	64		R	T	0.396	0.087	0.016	6.37E-08		0.998
DQA1	66		M	I	0.396	0.087	0.016	6.20E-08		0.999
DQA1	69	L	P	A	0.580	-0.097	0.016	1.01E-09		0.998
DQA1	69	A	P	A	0.396	0.087	0.016	6.20E-08		0.999
DQA1	75		S	I	0.240	-0.084	0.018	4.44E-06		0.995
DQA1	76	L	P	A	0.400	-0.100	0.016	6.37E-10		0.985
DQA1	76	M	P	A	0.396	0.087	0.016	6.31E-08		0.998
DQA1	80		Y	S	0.396	0.087	0.016	6.20E-08		0.999
DQA1	107	T	P	A	0.758	0.085	0.018	3.78E-06		0.986
DQA1	107	I	P	A	0.240	-0.084	0.018	4.48E-06		0.995
DQA1	130	S	P	A	0.829	-0.148	0.023	5.21E-11		0.817
DQA1	130	A	P	A	0.172	0.148	0.023	5.87E-11		0.818
DQA1	156	F	P	A	0.760	0.084	0.018	4.49E-06		0.994
DQA1	156	L	P	A	0.240	-0.084	0.018	4.49E-06		0.994
DQA1	161	D	P	A	0.760	0.084	0.018	4.48E-06		0.995
DQA1	161	E	P	A	0.240	-0.084	0.018	4.50E-06		0.994

DQA1	163	I	P	A	0.760	0.084	0.018	4.48E-06		0.994
DQA1	163	S	P	A	0.240	-0.084	0.018	4.49E-06		0.995
DQA1	175	Qx	P	A	0.396	0.088	0.016	5.49E-08	1.83E-08	0.999
DQA1	175	Q	P	A	0.396	0.088	0.016	5.54E-08		0.999
DQA1	175	K	P	A	0.240	-0.084	0.018	4.49E-06		0.995
DQA1	175	QE	P	A	0.760	0.084	0.018	4.65E-06		0.995
DQA1	207	M	P	A	0.085	0.168	0.034	9.79E-07		0.593
DQA1	207	V	P	A	0.915	-0.167	0.034	9.99E-07		0.593
DQA1	218	R	P	A	0.603	-0.089	0.016	3.54E-08		0.999
DQA1	218	Q	P	A	0.397	0.089	0.016	3.68E-08		0.999
DQB1	-4	V	P	A	0.607	-0.090	0.016	2.23E-08		0.997
DQB1	-4	L	P	A	0.361	0.085	0.017	3.07E-07		0.976
DQB1	-5	PS	P	A	0.757	-0.134	0.018	9.65E-14	1.84E-12	0.997
DQB1	-5	L	P	A	0.211	0.139	0.019	5.97E-13		0.970
DQB1	-5	P	P	A	0.607	-0.090	0.016	2.24E-08		0.996
DQB1	-5	Px	P	A	0.639	-0.085	0.017	3.07E-07		0.976
DQB1	-6	T	P	A	0.605	-0.092	0.016	1.36E-08	1.66E-07	0.988
DQB1	-6	TA	P	A	0.607	-0.090	0.016	2.26E-08		0.996
DQB1	-6	Tx	P	A	0.637	-0.087	0.017	1.93E-07		0.967
DQB1	-6	S	P	A	0.361	0.085	0.017	3.07E-07		0.976
DQB1	-10	S	P	A	0.254	-0.117	0.018	7.14E-11		0.999
DQB1	-10	A	P	A	0.714	0.099	0.017	1.40E-08		0.974
DQB1	-18	A	P	A	0.434	-0.121	0.016	3.25E-14		0.990
DQB1	-18	V	P	A	0.534	0.111	0.016	2.97E-12		0.971
DQB1	-21	G	P	A	0.607	-0.090	0.016	2.25E-08		0.996
DQB1	-21	D	P	A	0.361	0.085	0.017	3.07E-07		0.976
DQB1	9	YL	P	A	0.836	-0.201	0.022	3.01E-20	4.35E-19	0.912
DQB1	9	F	P	A	0.164	0.201	0.022	3.02E-20		0.912
DQB1	9	Y	P	A	0.833	-0.193	0.022	3.91E-19		0.913
DQB1	9	Yx	P	A	0.833	-0.193	0.022	3.91E-19		0.913
DQB1	28		S	T	0.254	-0.117	0.018	7.01E-11		1.000
DQB1	30	S	P	A	0.254	-0.117	0.018	7.01E-11		1.000
DQB1	30	Y	P	A	0.500	0.101	0.016	2.69E-10		0.939
DQB1	37	Y	P	A	0.743	0.119	0.018	2.85E-11		0.999
DQB1	37	I	P	A	0.254	-0.117	0.018	7.01E-11		1.000
DQB1	38		V	A	0.409	-0.109	0.016	7.37E-12		0.996
DQB1	46		E	V	0.254	-0.117	0.018	7.05E-11		0.999
DQB1	47		F	Y	0.254	-0.117	0.018	7.02E-11		0.999
DQB1	52		L	P	0.254	-0.117	0.018	7.02E-11		0.999
DQB1	53		Q	L	0.395	0.088	0.016	5.31E-08		0.997

DQB1	55	L	P	A	0.254	-0.117	0.018	7.02E-11		0.999
DQB1	55	R	P	A	0.417	0.099	0.016	6.64E-10		0.997
DQB1	57	AV	P	A	0.552	-0.078	0.016	1.58E-06		0.952
DQB1	57	A	P	A	0.401	-0.073	0.016	6.35E-06	3.21E-05	0.982
DQB1	57	D	P	A	0.438	0.069	0.016	2.13E-05		0.950
DQB1	66		D	E	0.281	-0.097	0.017	3.28E-08		0.998
DQB1	67		I	V	0.281	-0.096	0.017	3.30E-08		0.998
DQB1	70	R	P	A	0.618	-0.099	0.016	2.15E-09		0.966
DQB1	70	G	P	A	0.358	0.086	0.017	2.45E-07		0.965
DQB1	71	KA	P	A	0.404	-0.108	0.016	9.86E-12		0.996
DQB1	71	K	P	A	0.254	-0.117	0.018	7.02E-11	2.00E-12	0.999
DQB1	71	T	P	A	0.572	0.093	0.016	3.90E-09		0.995
DQB1	71	KD	P	A	0.278	-0.094	0.018	7.17E-08		0.998
DQB1	74	A	P	A	0.254	-0.117	0.018	7.02E-11		0.999
DQB1	74	E	P	A	0.572	0.093	0.016	3.84E-09		0.995
DQB1	75		V	L	0.428	-0.093	0.016	3.88E-09		0.995
DQB1	77		R	T	0.406	-0.107	0.016	1.88E-11		0.995
DQB1	84	Q	P	A	0.605	-0.088	0.016	5.41E-08		0.996
DQB1	84	E	P	A	0.393	0.087	0.016	8.65E-08		0.996
DQB1	85	L	P	A	0.605	-0.088	0.016	5.38E-08		0.997
DQB1	85	V	P	A	0.393	0.087	0.016	8.66E-08		0.996
DQB1	86	E	P	A	0.605	-0.088	0.016	5.44E-08		0.996
DQB1	86	Ex	P	A	0.607	-0.087	0.016	8.62E-08	4.05E-07	0.996
DQB1	86	EG	P	A	0.637	-0.085	0.017	3.71E-07		0.965
DQB1	86	A	P	A	0.361	0.083	0.017	5.87E-07		0.965
DQB1	87	LY	P	A	0.786	-0.137	0.019	8.99E-13		0.956
DQB1	87	F	P	A	0.212	0.137	0.019	1.57E-12	5.29E-12	0.955
DQB1	87	L	P	A	0.605	-0.088	0.016	5.34E-08		0.997
DQB1	87	Lx	P	A	0.606	-0.087	0.016	8.54E-08		0.997
DQB1	89	T	P	A	0.605	-0.088	0.016	5.41E-08		0.997
DQB1	89	G	P	A	0.393	0.087	0.016	8.62E-08		0.996
DQB1	90	T	P	A	0.605	-0.088	0.016	5.31E-08		0.997
DQB1	90	I	P	A	0.394	0.087	0.016	8.51E-08		0.997
DQB1	125	AS	P	A	0.752	-0.130	0.018	4.75E-13		0.989
DQB1	125	G	P	A	0.244	0.130	0.018	7.12E-13	1.99E-12	0.998
DQB1	125	A	P	A	0.605	-0.088	0.016	5.33E-08		0.998
DQB1	125	Ax	P	A	0.606	-0.087	0.016	8.54E-08		0.997
DQB1	203	I	P	A	0.606	-0.091	0.016	2.31E-08		0.991
DQB1	203	V	P	A	0.391	0.089	0.016	3.54E-08		0.997
DQB1	220	H	P	A	0.603	-0.088	0.016	6.00E-08		0.985

DQB1	220	R	P	A	0.394	0.087	0.016	8.02E-08		0.996
DQB1	221	H	P	A	0.604	-0.088	0.016	6.93E-08		0.986
DQB1	221	Q	P	A	0.394	0.087	0.016	7.62E-08		0.995
DRB1	-1	A	P	A	0.814	-0.196	0.021	3.01E-21		0.921
DRB1	-1	S	P	A	0.149	0.198	0.022	1.27E-19		0.994
**DRB1**	**-17**	**A**	**P**	**A**	**0.801**	**-0.194**	**0.020**	**1.03E-21**		**0.911**
DRB1	-17	T	P	A	0.162	0.195	0.021	6.16E-20		0.977
DRB1	-25	R	P	A	0.462	-0.085	0.016	1.24E-07		0.944
DRB1	-25	K	P	A	0.501	0.071	0.016	6.80E-06		0.986
DRB1	4	R	P	A	0.846	0.098	0.022	8.38E-06		0.974
DRB1	4	Q	P	A	0.151	-0.097	0.022	9.89E-06		1.000
DRB1	9	W	P	A	0.300	0.071	0.017	3.05E-05		0.985
DRB1	10	Q	P	A	0.501	0.070	0.016	6.88E-06		0.987
DRB1	10	Y	P	A	0.493	-0.066	0.016	2.43E-05		0.987
DRB1	11	P	P	A	0.156	0.201	0.021	8.06E-21		1.000
DRB1	11	PL	P	A	0.162	0.195	0.021	6.16E-20		0.977
DRB1	11	PD	P	A	0.168	0.181	0.021	3.17E-18		1.000
DRB1	11	SVG	P	A	0.826	-0.176	0.021	1.81E-17		0.978
DRB1	11	PV	P	A	0.350	0.129	0.016	3.94E-15		0.999
DRB1	11	SGD	P	A	0.644	-0.127	0.016	1.02E-14		0.986
DRB1	11	SGL	P	A	0.638	-0.122	0.016	5.98E-14		0.999
DRB1	11	SG	P	A	0.632	-0.120	0.016	1.45E-13		0.986
DRB1	11	SLD	P	A	0.511	-0.072	0.016	4.17E-06	7.87E-21	0.999
DRB1	11	SPV	P	A	0.844	0.099	0.022	5.48E-06		0.975
DRB1	11	SD	P	A	0.506	-0.071	0.016	6.66E-06		0.987
DRB1	11	GD	P	A	0.151	-0.097	0.022	9.89E-06		1.000
DRB1	11	SL	P	A	0.499	-0.067	0.016	1.57E-05		0.999
DRB1	11	GL	P	A	0.144	-0.097	0.022	1.70E-05		0.974
DRB1	11	PG	P	A	0.295	0.074	0.017	1.75E-05		1.000
DRB1	11	S	P	A	0.493	-0.066	0.016	2.43E-05		0.987
DRB1	11	G	P	A	0.138	-0.095	0.023	3.00E-05		1.000
DRB1	11	SVD	P	A	0.700	-0.071	0.017	3.05E-05		0.985
DRB1	12		T	K	0.493	-0.066	0.016	2.43E-05		0.987
DRB1	13	R	P	A	0.156	0.201	0.021	8.06E-21		1.000
DRB1	13	RG	P	A	0.204	0.178	0.020	1.73E-19		0.932
DRB1	13	SYF	P	A	0.607	-0.137	0.016	3.46E-17		0.953
DRB1	13	RH	P	A	0.345	0.134	0.016	2.90E-16	**4.46E-22**	1.000
DRB1	13	RF	P	A	0.180	0.165	0.020	8.99E-16		0.978
DRB1	13	SHY	P	A	0.772	-0.151	0.019	2.90E-15		0.919
DRB1	13	SY	P	A	0.584	-0.124	0.016	1.57E-14		0.941

DRB1	13	SYG	P	A	0.632	-0.120	0.016	1.45E-13		0.986
DRB1	13	SF	P	A	0.469	-0.084	0.016	1.15E-07		0.955
DRB1	13	SFG	P	A	0.517	-0.076	0.016	1.06E-06		1.000
DRB1	13	YF	P	A	0.162	-0.104	0.021	1.19E-06		0.976
DRB1	13	S	P	A	0.445	-0.075	0.016	3.33E-06		0.942
DRB1	13	SHF	P	A	0.657	-0.076	0.017	5.54E-06		0.950
DRB1	13	RY	P	A	0.295	0.074	0.017	1.75E-05		1.000
DRB1	13	SG	P	A	0.493	-0.066	0.016	2.43E-05		0.987
DRB1	13	Y	P	A	0.138	-0.095	0.023	3.00E-05		1.000
DRB1	13	SRH	P	A	0.790	0.082	0.020	3.84E-05		0.912
DRB1	14		K	E	0.138	-0.095	0.023	3.00E-05		1.000
DRB1	16	V	P	A	0.462	-0.085	0.016	1.24E-07		0.944
DRB1	16	A	P	A	0.501	0.071	0.016	6.80E-06		0.986
DRB1	25		Q	R	0.138	-0.095	0.023	3.00E-05		1.000
DRB1	26	F	P	A	0.837	0.160	0.030	1.31E-07		0.343
DRB1	28	D	P	A	0.812	0.115	0.021	5.52E-08		0.882
DRB1	28	E	P	A	0.175	-0.114	0.022	1.97E-07		0.876
DRB1	30	YH	P	A	0.838	0.104	0.021	1.19E-06		0.976
DRB1	30	Y	P	A	0.815	0.102	0.021	1.47E-06		0.898
DRB1	30	YCH	P	A	0.844	0.102	0.022	2.20E-06		1.000
DRB1	30	YC	P	A	0.821	0.100	0.021	2.76E-06		0.918
DRB1	30	YHG	P	A	0.850	0.102	0.022	3.79E-06		0.974
DRB1	30	YG	P	A	0.827	0.100	0.022	4.88E-06		0.893
DRB1	30	YHR	P	A	0.844	0.099	0.022	5.48E-06		0.975
DRB1	30	YR	P	A	0.821	0.096	0.021	6.66E-06	3.04E-06	0.896
DRB1	30	LR	P	A	0.144	-0.100	0.022	6.87E-06		0.999
DRB1	30	YCG	P	A	0.833	0.097	0.022	8.96E-06		0.913
DRB1	30	LG	P	A	0.151	-0.097	0.022	9.89E-06		1.000
DRB1	30	YCR	P	A	0.826	0.094	0.022	1.22E-05		0.916
DRB1	30	LC	P	A	0.144	-0.097	0.022	1.70E-05		0.974
DRB1	30	YGR	P	A	0.833	0.094	0.022	2.14E-05		0.890
DRB1	30	L	P	A	0.138	-0.095	0.023	3.00E-05		1.000
DRB1	30	LH	P	A	0.161	-0.092	0.022	3.86E-05		0.910
DRB1	32	NA	H	Y	0.392	-0.099	0.019	8.47E-08		0.734
DRB1	37	S	P	A	0.169	0.197	0.021	4.65E-20		0.934
DRB1	37	SY	P	A	0.469	0.156	0.018	9.52E-18		0.714
DRB1	37	SL	P	A	0.184	0.177	0.021	2.59E-17	2.98E-20	0.911
DRB1	37	NF	P	A	0.516	-0.149	0.018	2.79E-16		0.703
DRB1	37	NL	P	A	0.376	-0.100	0.019	1.57E-07		0.675
DRB1	37	N	P	A	0.361	-0.094	0.019	1.14E-06		0.664

DRB1	37	FL	P	A	0.170	-0.105	0.022	1.53E-06		0.885
DRB1	37	F	P	A	0.155	-0.101	0.023	8.59E-06		0.898
DRB1	57	DS	P	A	0.819	0.107	0.021	4.63E-07		0.895
DRB1	57	V	P	A	0.166	-0.103	0.021	1.56E-06		0.969
DRB1	60	Y	P	A	0.819	0.107	0.021	4.63E-07		0.895
DRB1	60	S	P	A	0.166	-0.103	0.021	1.56E-06		0.969
DRB1	70	Q	P	A	0.444	0.125	0.021	1.55E-09		0.471
DRB1	70	D	P	A	0.523	-0.102	0.020	6.40E-07		0.488
DRB1	71	A	P	A	0.127	0.248	0.026	6.63E-21		0.785
DRB1	71	KA	P	A	0.367	0.180	0.025	7.46E-13	7.18E-21	0.328
DRB1	71	KR	P	A	0.632	-0.103	0.021	1.27E-06		0.478
DRB1	73	NA	G	A	0.259	-0.142	0.022	1.08E-10		0.542
DRB1	74	AL	P	A	0.700	0.146	0.021	9.98E-12		0.540
DRB1	74	RQ	P	A	0.259	-0.142	0.022	1.08E-10		0.542
DRB1	74	A	P	A	0.674	0.118	0.021	1.32E-08		0.554
DRB1	74	AE	P	A	0.715	0.112	0.021	1.32E-07	1.11E-07	0.558
DRB1	74	QE	P	A	0.180	-0.103	0.022	3.32E-06		0.825
DRB1	74	RE	P	A	0.162	-0.140	0.032	1.51E-05		0.321
DRB1	74	Q	P	A	0.138	-0.095	0.023	3.00E-05		1.000
DRB1	78	NA	V	Y	0.151	-0.097	0.022	9.89E-06		1.000
DRB1	96	Qx	P	A	0.175	0.191	0.021	4.25E-19		0.910
DRB1	96	Q	P	A	0.162	0.188	0.021	7.37E-19		0.999
DRB1	96	QE	P	A	0.186	0.183	0.021	9.58E-18		0.864
DRB1	96	HY	P	A	0.801	-0.183	0.021	1.34E-17	1.05E-19	0.796
DRB1	96	HE	P	A	0.637	-0.134	0.017	9.51E-16		0.946
DRB1	96	QY	P	A	0.350	0.130	0.016	2.20E-15		0.997
DRB1	96	H	P	A	0.612	-0.132	0.017	5.05E-15		0.867
DRB1	96	Hx	P	A	0.626	-0.129	0.017	7.53E-15		0.912
DRB1	133	R	P	A	0.818	-0.205	0.022	4.34E-21		0.834
DRB1	133	L	P	A	0.156	0.201	0.021	7.00E-21		0.999
DRB1	142	V	P	A	0.818	-0.205	0.022	4.34E-21		0.834
DRB1	142	M	P	A	0.156	0.201	0.021	7.00E-21		0.999
DRB1	149	H	P	A	0.449	-0.072	0.017	1.61E-05		0.837
DRB1	149	Q	P	A	0.525	0.068	0.016	2.18E-05		0.919
DRB1	181	M	P	A	0.163	-0.104	0.022	1.69E-06		0.949
DRB1	233	R	P	A	0.335	-0.085	0.017	6.11E-07		0.954
DRB1	233	T	P	A	0.626	0.069	0.016	2.51E-05		0.985

Table S15: Associations for HLA amino acid (AA) sequence substitutions that were significantly associated ($P<4.6\times10^{-5}$) with seroreactivity to MCV. At each position that contained a significantly associated allele and >2 possible substitutions, we tested all common (frequency ≥0.01) haplotypes at that position using the omnibus test (P_{omni}) in the conditional haplotype module of PLINK 1.07. Variants and haplotypes with the lowest p-value are indicated in bold and shaded.

Gene	AA position	AA	A1	A2	FRQ	BETA	SE	P	P_{omni}	INFO
A	9	T	P	A	0.077	-0.204	0.037	2.69E-08		0.996
A	9	FY	P	A	0.805	0.124	0.025	5.37E-07		0.991
A	62	L	P	A	0.040	-0.360	0.050	9.71E-13		0.998
A	62	EL	P	A	0.135	-0.178	0.029	5.32E-10	1.10E-12	0.999
A	62	QG	P	A	0.768	0.105	0.024	8.24E-06		0.940
A	63	Q	P	A	0.040	-0.360	0.050	1.06E-12		0.997
A	77	N	P	A	0.366	-0.086	0.021	3.43E-05		0.872
A	97	M	P	A	0.235	-0.101	0.023	1.55E-05		0.945
A	97	IR	P	A	0.765	0.101	0.023	1.59E-05		0.945
B	143	S	P	A	0.073	-0.221	0.054	4.60E-05		0.416
B	147	L	P	A	0.073	-0.221	0.054	4.62E-05		0.415
B	178	K	P	A	0.210	-0.128	0.027	2.14E-06		0.800
B	178	T	P	A	0.771	0.121	0.027	1.05E-05		0.712
C	99	S	P	A	0.157	-0.118	0.028	2.18E-05		0.998
C	116	S	P	A	0.571	-0.089	0.020	9.84E-06	1.11E-04	0.939
C	116	SL	P	A	0.585	-0.086	0.020	2.02E-05		0.929
C	152	A	P	A	0.403	-0.082	0.020	3.82E-05		0.999
DQA1	25		F	Y	0.310	-0.111	0.022	6.43E-07		0.858
DQA1	41		K	R	0.171	-0.155	0.029	6.74E-08		0.816
DQA1	129	Q	P	A	0.224	0.152	0.024	3.32E-10		0.831
DQA1	129	H	P	A	0.776	-0.152	0.024	3.33E-10		0.831
DQA1	130	S	P	A	0.829	0.155	0.029	6.53E-08		0.817
DQA1	130	A	P	A	0.172	-0.155	0.029	6.75E-08		0.818
DQB1	-5	S	P	A	0.150	0.210	0.026	1.07E-15		0.992
DQB1	-5	PL	P	A	0.819	-0.183	0.025	1.44E-13	2.64E-17	0.959
DQB1	-5	L	P	A	0.211	-0.138	0.024	1.73E-08		0.970
DQB1	-5	PS	P	A	0.757	0.124	0.023	5.77E-08		0.997
DQB1	-9	I	P	A	0.150	0.210	0.026	1.07E-15		0.992
DQB1	-9	M	P	A	0.819	-0.183	0.025	1.46E-13		0.959
DQB1	-27	S	P	A	0.150	0.210	0.026	1.06E-15		0.992
DQB1	-27	A	P	A	0.819	-0.183	0.025	1.45E-13		0.959
DQB1	9	YL	P	A	0.836	0.201	0.028	1.07E-12	2.11E-12	0.912
DQB1	9	F	P	A	0.164	-0.201	0.028	1.07E-12		0.912

DQB1	9	Y	P	A	0.833	0.196	0.028	2.42E-12		0.913
DQB1	9	Yx	P	A	0.833	0.196	0.028	2.42E-12		0.913
DQB1	14	L	P	A	0.152	0.209	0.026	1.31E-15		0.992
DQB1	14	M	P	A	0.848	-0.209	0.026	1.31E-15		0.992
**DQB1**	**26**	**L**	**P**	**A**	**0.646**	**-0.173**	**0.020**	**7.03E-18**		**0.990**
DQB1	26	G	P	A	0.174	0.176	0.025	1.74E-12		0.991
DQB1	30	H	P	A	0.246	0.154	0.023	1.44E-11		0.918
DQB1	30	Y	P	A	0.500	-0.085	0.020	1.57E-05		0.939
DQB1	38		V	A	0.409	0.087	0.019	7.43E-06		0.996
DQB1	57	AD	P	A	0.839	-0.183	0.026	2.61E-12		0.937
DQB1	57	V	P	A	0.151	0.185	0.027	4.41E-12		0.939
DQB1	71	A	P	A	0.150	0.210	0.026	1.07E-15		0.992
DQB1	71	KT	P	A	0.827	-0.176	0.025	1.70E-12	3.29E-14	0.992
DQB1	71	KA	P	A	0.404	0.086	0.019	8.82E-06		0.996
DQB1	74	S	P	A	0.173	0.176	0.025	1.72E-12		0.992
DQB1	77		R	T	0.406	0.086	0.019	8.67E-06		0.995
DQB1	87	Y	P	A	0.182	0.185	0.025	9.74E-14		0.945
DQB1	87	LF	P	A	0.816	-0.184	0.025	1.16E-13	8.92E-16	0.945
DQB1	87	F	P	A	0.212	-0.139	0.025	1.71E-08		0.955
DQB1	87	LY	P	A	0.786	0.137	0.024	2.18E-08		0.956
DQB1	116	I	P	A	0.150	0.210	0.026	1.06E-15		0.992
DQB1	116	V	P	A	0.849	-0.209	0.026	1.28E-15		0.992
DQB1	125	S	P	A	0.150	0.210	0.026	1.06E-15		0.992
DQB1	125	AG	P	A	0.849	-0.209	0.026	1.28E-15	**1.98E-17**	0.992
DQB1	125	G	P	A	0.244	-0.123	0.023	7.22E-08		0.998
DQB1	125	AS	P	A	0.752	0.122	0.023	7.50E-08		0.989
DQB1	224	Q	P	A	0.848	-0.211	0.026	9.21E-16		0.975
DQB1	224	R	P	A	0.150	0.210	0.026	1.06E-15		0.992
DRB1	-1	S	P	A	0.149	-0.208	0.029	4.04E-13		0.994
DRB1	-1	A	P	A	0.814	0.186	0.027	3.97E-12		0.921
DRB1	-16	V	P	A	0.462	0.136	0.020	4.39E-12		0.944
DRB1	-16	A	P	A	0.501	-0.128	0.019	2.59E-11		0.986
DRB1	-17	T	P	A	0.162	-0.177	0.028	2.12E-10		0.977
DRB1	-17	A	P	A	0.801	0.161	0.026	7.95E-10		0.911
DRB1	-25	R	P	A	0.462	0.136	0.020	4.39E-12		0.944
DRB1	-25	K	P	A	0.501	-0.128	0.019	2.59E-11		0.986
DRB1	9	W	P	A	0.300	-0.125	0.021	6.54E-09		0.985
DRB1	9	E	P	A	0.687	0.119	0.021	2.22E-08		0.985
DRB1	10	Q	P	A	0.501	-0.128	0.019	2.61E-11		0.987
DRB1	10	Y	P	A	0.493	0.123	0.019	1.64E-10		0.987

DRB1	11	P	P	A	0.156	-0.188	0.028	2.06E-11	5.56E-13	1.000
DRB1	11	SLD	P	A	0.511	0.128	0.019	2.13E-11		0.999
DRB1	11	SL	P	A	0.499	0.126	0.019	5.02E-11		0.999
DRB1	11	SD	P	A	0.506	0.125	0.019	7.20E-11		0.987
DRB1	11	S	P	A	0.493	0.123	0.019	1.64E-10		0.987
DRB1	11	PL	P	A	0.162	-0.177	0.028	2.12E-10		0.977
DRB1	11	PD	P	A	0.168	-0.171	0.027	2.87E-10		1.000
DRB1	11	PV	P	A	0.350	-0.129	0.020	3.07E-10		0.999
DRB1	11	SGL	P	A	0.638	0.124	0.020	9.28E-10		0.999
DRB1	11	SGD	P	A	0.644	0.124	0.020	1.22E-09		0.986
DRB1	11	PG	P	A	0.295	-0.130	0.021	1.63E-09		1.000
DRB1	11	SVG	P	A	0.826	0.161	0.027	2.28E-09		0.978
DRB1	11	SG	P	A	0.632	0.120	0.020	3.47E-09		0.986
DRB1	11	SVL	P	A	0.693	0.124	0.021	6.00E-09		1.000
DRB1	11	SVD	P	A	0.700	0.125	0.021	6.54E-09		0.985
DRB1	11	SV	P	A	0.687	0.119	0.021	2.22E-08		0.985
DRB1	12		T	K	0.493	0.123	0.019	1.64E-10		0.987
DRB1	13	SFG	P	A	0.517	0.134	0.019	3.08E-12	8.51E-14	1.000
DRB1	13	SF	P	A	0.469	0.131	0.019	1.52E-11		0.955
DRB1	13	R	P	A	0.156	-0.188	0.028	2.06E-11		1.000
DRB1	13	RH	P	A	0.345	-0.136	0.020	3.75E-11		1.000
DRB1	13	SG	P	A	0.493	0.123	0.019	1.64E-10		0.987
DRB1	13	S	P	A	0.445	0.121	0.020	6.14E-10		0.942
DRB1	13	SYF	P	A	0.607	0.124	0.020	7.54E-10		0.953
DRB1	13	RY	P	A	0.295	-0.130	0.021	1.63E-09		1.000
DRB1	13	SYG	P	A	0.632	0.120	0.020	3.47E-09		0.986
DRB1	13	RG	P	A	0.204	-0.144	0.025	1.71E-08		0.932
DRB1	13	SHF	P	A	0.657	0.116	0.021	3.35E-08		0.950
DRB1	13	RF	P	A	0.180	-0.147	0.027	3.44E-08		0.978
DRB1	13	SY	P	A	0.584	0.110	0.020	3.87E-08		0.941
DRB1	13	SHG	P	A	0.682	0.111	0.021	1.40E-07		0.985
DRB1	13	SH	P	A	0.634	0.100	0.021	1.48E-06		0.938
DRB1	13	SHY	P	A	0.772	0.115	0.025	3.01E-06		0.919
DRB1	26	F	P	A	0.837	-0.229	0.037	4.19E-10		0.343
DRB1	32		H	Y	0.392	0.144	0.023	2.22E-10		0.734
DRB1	37	S	P	A	0.169	-0.169	0.028	1.60E-09	1.37E-11	0.934
DRB1	37	SL	P	A	0.184	-0.152	0.027	2.42E-08		0.911
DRB1	37	NL	P	A	0.376	0.124	0.023	1.20E-07		0.675
DRB1	37	N	P	A	0.361	0.121	0.024	3.22E-07		0.664
DRB1	37	SF	P	A	0.323	-0.109	0.022	7.27E-07		0.898

DRB1	37	SY	P	A	0.469	-0.110	0.023	1.05E-06		0.714
DRB1	37	NY	P	A	0.662	0.102	0.022	2.83E-06		0.889
DRB1	37	NF	P	A	0.516	0.105	0.023	3.25E-06		0.703
DRB1	71	A	P	A	0.127	-0.232	0.035	2.30E-11		0.785
DRB1	71	KE	P	A	0.481	0.111	0.026	2.27E-05		0.480
DRB1	74	RE	P	A	0.162	0.226	0.039	9.06E-09		0.321
DRB1	74	AQ	P	A	0.812	-0.171	0.035	1.21E-06	0.0119	0.378
DRB1	74	RL	P	A	0.146	0.174	0.040	1.35E-05		0.314
DRB1	96	QY	P	A	0.350	-0.129	0.020	2.54E-10		0.997
DRB1	96	Q	P	A	0.162	-0.171	0.028	6.19E-10		0.999
DRB1	96	HE	P	A	0.637	0.124	0.021	2.48E-09		0.946
DRB1	96	Qx	P	A	0.175	-0.157	0.028	1.98E-08		0.910
DRB1	96	Hx	P	A	0.626	0.110	0.021	1.33E-07		0.912
DRB1	96	H	P	A	0.612	0.102	0.021	1.25E-06		0.867
DRB1	96	QE	P	A	0.186	-0.130	0.028	3.00E-06		0.864
DRB1	133	L	P	A	0.156	-0.188	0.028	2.35E-11		0.999
DRB1	133	R	P	A	0.818	0.156	0.028	4.76E-08		0.834
DRB1	140	A	P	A	0.331	-0.103	0.022	2.26E-06		0.907
DRB1	142	M	P	A	0.156	-0.188	0.028	2.35E-11		0.999
DRB1	142	V	P	A	0.818	0.156	0.028	4.76E-08		0.834
DRB1	149	Q	P	A	0.525	-0.113	0.020	1.12E-08		0.919
DRB1	149	H	P	A	0.449	0.102	0.021	6.48E-07		0.837

Table S16: Associations for HLA amino acid (AA) sequence substitutions that were significantly associated ($P<4.6\times10^{-5}$) with seroreactivity to HHV7. At each position that contained a significantly associated allele and >2 possible substitutions, we tested all common (frequency ≥0.01) haplotypes at that position using the omnibus test (P_{omni}) in the conditional haplotype module of PLINK 1.07. Variants and haplotypes with the lowest p-value are indicated in bold and shaded.

Gene	AA position	AA	A1	A2	FRQ	BETA	SE	P	P_{omni}	INFO
B	67	SC	P	A	0.528	-0.075	0.017	6.25E-06		0.924
B	116	YDS	P	A	0.807	0.142	0.032	7.34E-06		0.324
B	116	Fx	P	A	0.119	-0.139	0.034	3.83E-05		0.427
DPB1	178	M	P	A	0.106	-0.131	0.026	4.01E-07		0.991
DPB1	178	L	P	A	0.892	0.131	0.026	4.38E-07		0.975
DQA1	-16	L	P	A	0.420	-0.088	0.016	5.70E-08		0.999
DQA1	-16	M	P	A	0.580	0.088	0.016	6.46E-08		0.998
DQA1	11	C	P	A	0.396	-0.088	0.016	9.68E-08		0.999
DQA1	11	Y	P	A	0.604	0.088	0.016	9.69E-08		0.999
DQA1	18		F	S	0.396	-0.088	0.016	9.69E-08		0.999
DQA1	45		A	V	0.396	-0.088	0.016	9.56E-08		0.999
DQA1	47	R	P	A	0.395	-0.088	0.016	9.46E-08		0.997
DQA1	47	RC	P	A	0.660	-0.085	0.017	3.59E-07		0.996
DQA1	48		W	L	0.396	-0.088	0.016	9.65E-08		0.999
DQA1	50	E	P	A	0.396	-0.088	0.016	9.51E-08		0.998
DQA1	50	L	P	A	0.340	0.085	0.017	3.60E-07		0.996
DQA1	52	S	P	A	0.396	-0.088	0.016	9.56E-08		0.999
DQA1	53	K	P	A	0.396	-0.088	0.016	9.65E-08		0.999
DQA1	53	R	P	A	0.340	0.085	0.017	3.60E-07		0.996
DQA1	55		G	R	0.396	-0.088	0.016	9.53E-08		0.998
DQA1	56	G	P	A	0.396	-0.088	0.016	9.53E-08		0.998
DQA1	61		G	F	0.396	-0.088	0.016	9.65E-08		0.999
DQA1	64		R	T	0.396	-0.088	0.016	9.53E-08		0.998
DQA1	66		M	I	0.396	-0.088	0.016	9.65E-08		0.999
DQA1	69	L	P	A	0.580	0.088	0.016	5.55E-08		0.998
DQA1	69	A	P	A	0.396	-0.088	0.016	9.65E-08		0.999
DQA1	76	M	P	A	0.396	-0.088	0.016	9.60E-08		0.998
DQA1	80		Y	S	0.396	-0.088	0.016	9.65E-08		0.999
DQA1	129	H	P	A	0.776	0.109	0.021	1.21E-07		0.831
DQA1	129	Q	P	A	0.224	-0.109	0.021	1.21E-07		0.831
DQA1	175	Qx	P	A	0.396	-0.088	0.016	9.83E-08		0.999
DQA1	175	Q	P	A	0.396	-0.088	0.016	9.87E-08	1.04E-07	0.999
DQA1	175	E	P	A	0.364	0.081	0.017	1.12E-06		0.996

DQA1	175	QK	P	A	0.636	-0.081	0.017	1.12E-06		0.996
DQA1	215	L	P	A	0.339	0.084	0.017	5.26E-07		0.996
DQA1	215	F	P	A	0.661	-0.084	0.017	5.27E-07		0.996
DQA1	218	R	P	A	0.603	0.086	0.016	1.63E-07		0.999
DQA1	218	Q	P	A	0.397	-0.086	0.016	1.66E-07		0.999
DQB1	-4	L	P	A	0.361	-0.095	0.017	2.40E-08		0.976
DQB1	-4	V	P	A	0.607	0.088	0.017	8.73E-08		0.997
DQB1	-5	Px	P	A	0.639	0.095	0.017	2.40E-08	2.44E-08	0.976
DQB1	-5	P	P	A	0.607	0.088	0.017	8.72E-08		0.996
DQB1	-5	S	P	A	0.150	-0.120	0.023	1.45E-07		0.992
DQB1	-5	PL	P	A	0.819	0.103	0.021	1.48E-06		0.959
DQB1	-6	Tx	P	A	0.637	0.095	0.017	2.23E-08	3.27E-07	0.967
DQB1	-6	S	P	A	0.361	-0.095	0.017	2.40E-08		0.976
DQB1	-6	T	P	A	0.605	0.089	0.017	8.11E-08		0.988
DQB1	-6	TA	P	A	0.607	0.088	0.017	8.72E-08		0.996
DQB1	-9	I	P	A	0.150	-0.120	0.023	1.45E-07		0.992
DQB1	-9	M	P	A	0.819	0.103	0.021	1.48E-06		0.959
DQB1	-18	V	P	A	0.534	-0.066	0.016	4.27E-05		0.971
DQB1	-21	D	P	A	0.361	-0.095	0.017	2.40E-08		0.976
DQB1	-21	G	P	A	0.607	0.089	0.017	8.59E-08		0.996
DQB1	-27	S	P	A	0.150	-0.120	0.023	1.45E-07		0.992
DQB1	-27	A	P	A	0.819	0.103	0.021	1.47E-06		0.959
DQB1	14	L	P	A	0.152	-0.114	0.023	4.75E-07		0.992
DQB1	14	M	P	A	0.848	0.114	0.023	4.78E-07		0.992
DQB1	26	G	P	A	0.174	-0.101	0.021	2.28E-06		0.991
**DQB1**	**30**	**H**	**P**	**A**	**0.246**	**-0.111**	**0.019**	**1.16E-08**		**0.918**
DQB1	53		Q	L	0.395	-0.088	0.016	9.66E-08		0.997
DQB1	55	R	P	A	0.417	-0.086	0.016	1.43E-07		0.997
DQB1	57	V	P	A	0.151	-0.123	0.023	8.14E-08	2.48E-08	0.939
DQB1	57	AD	P	A	0.839	0.112	0.022	6.03E-07		0.937
DQB1	57	AS	P	A	0.411	0.069	0.017	2.67E-05		0.981
DQB1	70	G	P	A	0.358	-0.095	0.017	2.63E-08		0.965
DQB1	70	R	P	A	0.618	0.089	0.017	1.11E-07		0.966
DQB1	71	A	P	A	0.150	-0.120	0.023	1.45E-07		0.992
DQB1	71	KT	P	A	0.827	0.101	0.021	2.27E-06		0.992
DQB1	74	S	P	A	0.173	-0.101	0.021	2.28E-06		0.992
DQB1	84	E	P	A	0.393	-0.091	0.017	3.98E-08		0.996
DQB1	84	Q	P	A	0.605	0.088	0.016	9.58E-08		0.996
DQB1	85	V	P	A	0.393	-0.091	0.017	3.98E-08		0.996
DQB1	85	L	P	A	0.605	0.088	0.016	9.60E-08		0.997

DQB1	86	A	P	A	0.361	-0.095	0.017	2.89E-08		0.965
DQB1	86	Ex	P	A	0.607	0.091	0.017	3.98E-08	5.71E-07	0.996
DQB1	86	EG	P	A	0.637	0.092	0.017	7.25E-08		0.965
DQB1	86	E	P	A	0.605	0.088	0.016	9.66E-08		0.996
DQB1	87	Lx	P	A	0.606	0.091	0.017	4.04E-08		0.997
DQB1	87	L	P	A	0.605	0.088	0.016	9.73E-08	2.41E-08	0.997
DQB1	87	Y	P	A	0.182	-0.109	0.021	3.59E-07		0.945
DQB1	87	LF	P	A	0.816	0.104	0.021	1.04E-06		0.945
DQB1	89	G	P	A	0.393	-0.091	0.017	3.97E-08		0.996
DQB1	89	T	P	A	0.605	0.088	0.016	9.59E-08		0.997
DQB1	90	I	P	A	0.394	-0.091	0.017	4.01E-08		0.997
DQB1	90	T	P	A	0.605	0.088	0.016	9.66E-08		0.997
DQB1	116	I	P	A	0.150	-0.120	0.023	1.46E-07		0.992
DQB1	116	V	P	A	0.849	0.114	0.023	4.75E-07		0.992
DQB1	125	Ax	P	A	0.606	0.091	0.017	4.01E-08		0.997
DQB1	125	A	P	A	0.605	0.088	0.016	9.64E-08	**5.63E-09**	0.998
DQB1	125	S	P	A	0.150	-0.120	0.023	1.46E-07		0.992
DQB1	125	AG	P	A	0.849	0.114	0.023	4.75E-07		0.992
DQB1	203	V	P	A	0.391	-0.091	0.017	3.70E-08		0.997
DQB1	203	I	P	A	0.606	0.088	0.017	1.03E-07		0.991
DQB1	220	R	P	A	0.394	-0.090	0.017	4.47E-08		0.996
DQB1	220	H	P	A	0.603	0.089	0.017	9.20E-08		0.985
DQB1	221	Q	P	A	0.394	-0.090	0.017	4.81E-08		0.995
DQB1	221	H	P	A	0.604	0.089	0.017	7.19E-08		0.986
DQB1	224	R	P	A	0.150	-0.120	0.023	1.46E-07		0.992
DQB1	224	Q	P	A	0.848	0.117	0.023	2.78E-07		0.975
DRB1	-16	A	P	A	0.501	0.067	0.016	2.69E-05		0.986
DRB1	-25	K	P	A	0.501	0.067	0.016	2.69E-05		0.986
DRB1	10	Q	P	A	0.501	0.067	0.016	2.65E-05		0.987
DRB1	11	VG	P	A	0.332	0.085	0.017	4.64E-07		0.999
DRB1	11	SPL	P	A	0.655	-0.080	0.017	1.74E-06		0.999
DRB1	11	SLD	P	A	0.511	-0.075	0.016	3.04E-06		0.999
DRB1	11	SPD	P	A	0.662	-0.077	0.017	4.48E-06	3.68E-07	0.986
DRB1	11	SL	P	A	0.499	-0.071	0.016	8.07E-06		0.999
DRB1	11	SP	P	A	0.650	-0.072	0.017	1.46E-05		0.986
DRB1	11	SD	P	A	0.506	-0.068	0.016	2.07E-05		0.987
DRB1	13	HY	P	A	0.327	0.088	0.017	1.80E-07		0.999
DRB1	13	SFG	P	A	0.517	-0.077	0.016	1.46E-06	7.25E-07	1
DRB1	13	SRF	P	A	0.625	-0.075	0.017	7.74E-06		0.952
DRB1	13	SRG	P	A	0.650	-0.072	0.017	1.46E-05		0.986

DRB1	13	SF	P	A	0.469	-0.069	0.016	2.43E-05	0.955
DRB1	98	E	P	A	0.339	0.083	0.017	6.70E-07	0.998
DRB1	98	K	P	A	0.648	-0.081	0.017	1.78E-06	0.946
DRB1	104	A	P	A	0.339	0.083	0.017	6.70E-07	0.998
DRB1	104	S	P	A	0.638	-0.079	0.017	4.15E-06	0.906

Table S17: Associations for HLA amino acid (AA) sequence substitutions that were significantly associated (P<4.6×10^{-5}) with seroreactivity to HHV6 IE1A and HHV6 IE1B. At each position that contained a significantly associated allele and >2 possible substitutions, we tested all common (frequency ≥0.01) haplotypes at that position using the omnibus test (P_{omni}) in the conditional haplotype module of PLINK 1.07. Variants and haplotypes with the lowest p-value are indicated in bold and shaded.

Phenotype	Gene	AA position	AA	A1	A2	FRQ	BETA	SE	P	P_{omni}	INFO
HHV6 IE1A	B	97	SWV	P	A	0.355	-0.088	0.020	8.91E-06		0.919
HHV6 IE1A	B	97	RN	P	A	0.534	0.088	0.020	9.71E-06		0.780
HHV6 IE1A	B	97	SV	P	A	0.350	-0.088	0.020	9.87E-06		0.924
HHV6 IE1A	B	97	STV	P	A	0.461	-0.088	0.020	1.03E-05		0.782
HHV6 IE1A	B	97	R	P	A	0.478	0.085	0.020	1.85E-05	4.43E-04	0.776
HHV6 IE1A	B	97	RW	P	A	0.483	0.085	0.020	2.00E-05		0.779
HHV6 IE1A	B	97	RT	P	A	0.589	0.081	0.019	2.86E-05		0.916
HHV6 IE1A	B	97	SW	P	A	0.318	-0.086	0.021	3.10E-05		0.908
HHV6 IE1A	B	97	SNV	P	A	0.407	-0.081	0.019	3.20E-05		0.921
HHV6 IE1A	B	97	S	P	A	0.313	-0.084	0.021	3.92E-05		0.926
**HHV6 IE1A**	**C**	**116**	**S**	**P**	**A**	**0.571**	**-0.099**	**0.019**	**1.80E-07**		0.939
HHV6 IE1A	C	116	SL	P	A	0.585	-0.094	0.019	8.69E-07	**4.56E-06**	0.929
HHV6 IE1A	C	116	SY	P	A	0.727	-0.085	0.020	2.96E-05		0.993
HHV6 IE1A	C	156	RD	P	A	0.282	0.094	0.021	5.99E-06	2.94E-04	0.944
HHV6 IE1A	C	156	R	P	A	0.265	0.091	0.021	1.47E-05		0.943
HHV6 IE1B	DQA1	25		F	Y	0.310	0.083	0.020	4.40E-05		0.858
HHV6 IE1B	DQA1	47	K	P	A	0.136	0.125	0.026	1.42E-06		0.979
HHV6 IE1B	DQA1	52	H	P	A	0.136	0.125	0.026	1.42E-06		0.979
HHV6 IE1B	DQA1	54		L	F	0.136	0.125	0.026	1.42E-06		0.979
HHV6 IE1B	DQB1	135	G	P	A	0.100	0.126	0.029	1.70E-05		0.993
HHV6 IE1B	DQB1	135	D	P	A	0.898	-0.118	0.029	4.15E-05		0.991
HHV6 IE1B	DRB1	4	Q	P	A	0.151	0.106	0.025	1.60E-05		1.000
HHV6 IE1B	DRB1	4	R	P	A	0.846	-0.106	0.025	1.65E-05		0.974
HHV6 IE1B	DRB1	11	G	P	A	0.138	0.120	0.025	2.62E-06		1.000
HHV6 IE1B	DRB1	11	GL	P	A	0.144	0.112	0.025	9.45E-06	1.46E-05	0.974
HHV6 IE1B	DRB1	11	GD	P	A	0.151	0.106	0.025	1.60E-05		1.000
HHV6 IE1B	DRB1	13	Y	P	A	0.138	0.120	0.025	2.62E-06		1.000
HHV6 IE1B	DRB1	13	YG	P	A	0.186	0.097	0.023	3.37E-05	**1.14E-05**	0.924
HHV6 IE1B	DRB1	13	YF	P	A	0.162	0.099	0.024	4.19E-05		0.976
HHV6 IE1B	DRB1	14	NA	K	E	0.138	0.120	0.025	2.62E-06		1.000
HHV6 IE1B	DRB1	25	NA	Q	R	0.138	0.120	0.025	2.62E-06		1.000
HHV6 IE1B	DRB1	28	E	P	A	0.175	0.106	0.025	1.64E-05		0.876
HHV6 IE1B	DRB1	30	LR	P	A	0.144	0.118	0.025	2.35E-06	1.53E-04	0.999

HHV6 IE1B	DRB1	30	L	P	A	0.138	0.120	0.025	2.62E-06		1.000
HHV6 IE1B	DRB1	30	YCG	P	A	0.833	-0.111	0.025	7.40E-06		0.913
HHV6 IE1B	DRB1	30	YHG	P	A	0.850	-0.111	0.025	8.29E-06		0.974
HHV6 IE1B	DRB1	30	LH	P	A	0.161	0.112	0.025	8.43E-06		0.910
HHV6 IE1B	DRB1	30	LC	P	A	0.144	0.112	0.025	9.45E-06		0.974
HHV6 IE1B	DRB1	30	YCH	P	A	0.844	-0.105	0.024	1.39E-05		1.000
HHV6 IE1B	DRB1	30	LG	P	A	0.151	0.106	0.025	1.60E-05		1.000
HHV6 IE1B	DRB1	30	YG	P	A	0.827	-0.104	0.025	2.35E-05		0.893
HHV6 IE1B	DRB1	30	YGR	P	A	0.833	-0.105	0.025	2.71E-05		0.890
HHV6 IE1B	DRB1	30	YC	P	A	0.821	-0.099	0.024	3.60E-05		0.918
HHV6 IE1B	DRB1	30	YH	P	A	0.838	-0.099	0.024	4.19E-05		0.976
HHV6 IE1B	DRB1	30	YCR	P	A	0.826	-0.099	0.024	4.20E-05		0.916
HHV6 IE1B	DRB1	37	F	P	A	0.155	0.118	0.025	3.63E-06		0.898
HHV6 IE1B	DRB1	37	FL	P	A	0.170	0.107	0.025	1.44E-05		0.885
HHV6 IE1B	DRB1	57	V	P	A	0.166	0.098	0.024	4.62E-05		0.969
HHV6 IE1B	DRB1	60	S	P	A	0.166	0.098	0.024	4.62E-05		0.969
**HHV6 IE1B**	**DRB1**	**67**	**I**	**P**	**A**	**0.526**	**0.131**	**0.023**	**1.57E-08**		**0.496**
HHV6 IE1B	DRB1	67	L	P	A	0.344	-0.116	0.023	6.80E-07		0.524
HHV6 IE1B	DRB1	70	D	P	A	0.523	0.125	0.023	8.81E-08		0.488
HHV6 IE1B	DRB1	70	Q	P	A	0.444	-0.123	0.024	1.73E-07		0.471
HHV6 IE1B	DRB1	71	KA	P	A	0.367	-0.117	0.028	4.00E-05		0.328
HHV6 IE1B	DRB1	74	Q	P	A	0.138	0.120	0.025	2.62E-06	8.68E-05	1.000
HHV6 IE1B	DRB1	74	QL	P	A	0.164	0.106	0.024	1.04E-05		0.988
HHV6 IE1B	DRB1	78		V	Y	0.151	0.106	0.025	1.60E-05		1.000
HHV6 IE1B	DRB1	180	V	P	A	0.752	0.095	0.023	2.86E-05		0.780
HHV6 IE1B	DRB1	181	M	P	A	0.163	0.106	0.024	1.44E-05		0.949

Table S18: Associations for HLA amino acid (AA) sequence substitutions that were significantly associated ($P<4.6\times10^{-5}$) with seroreactivity to EBV ZEBRA. At each position that contained a significantly associated allele and >2 possible substitutions, we tested all common (frequency ≥0.01) haplotypes at that position using the omnibus test (P_{omni}) in the conditional haplotype module of PLINK 1.07. Variants and haplotypes with the lowest p-value are indicated in bold and shaded.

Gene	AA position	AA	A1	A2	FRQ	BETA	SE	P	P_{omni}	INFO
A	-11	L	P	A	0.111	0.142	0.026	5.87E-08		0.996
A	-11	S	P	A	0.888	-0.141	0.026	7.34E-08		0.983
A	9	T	P	A	0.077	0.161	0.030	1.20E-07		0.996
A	9	FY	P	A	0.805	-0.089	0.021	1.64E-05		0.991
A	62	L	P	A	0.040	0.193	0.041	2.75E-06		0.998
A	63	Q	P	A	0.040	0.193	0.041	2.86E-06		0.997
A	70		Q	H	0.301	0.087	0.019	3.19E-06		0.905
A	144	Q	P	A	0.189	0.117	0.022	1.16E-07		0.913
A	144	K	P	A	0.811	-0.117	0.022	1.18E-07		0.913
A	151		R	H	0.145	0.147	0.025	2.02E-09		0.885
A	152	AV	P	A	0.791	-0.094	0.021	6.40E-06		0.946
A	152	ER	P	A	0.200	0.092	0.021	1.75E-05	3.84E-06	0.892
A	152	EW	P	A	0.197	0.091	0.022	2.39E-05		0.892
A	156	L	P	A	0.556	0.080	0.018	7.73E-06		0.851
A	161		D	E	0.146	0.106	0.023	6.47E-06		0.999
A	246	A	P	A	0.845	-0.105	0.023	4.26E-06		0.998
A	246	S	P	A	0.155	0.105	0.023	4.28E-06		0.999
A	298	F	P	A	0.110	0.136	0.026	2.23E-07		1.000
A	298	I	P	A	0.877	-0.125	0.026	1.47E-06		0.880
A	299	A	P	A	0.155	0.105	0.023	4.50E-06		0.999
A	299	T	P	A	0.832	-0.098	0.023	1.76E-05		0.906
A	307	R	P	A	0.110	0.136	0.026	2.23E-07		1.000
A	307	M	P	A	0.877	-0.125	0.026	1.46E-06		0.880
A	334	M	P	A	0.155	0.105	0.023	4.17E-06		0.999
A	334	V	P	A	0.833	-0.098	0.023	1.68E-05		0.906
B	9	D	P	A	0.146	-0.155	0.023	3.84E-11		0.999
B	24	T	P	A	0.333	0.116	0.017	1.66E-11		0.988
B	24	S	P	A	0.416	-0.077	0.017	4.02E-06		0.982
B	32		L	Q	0.313	0.115	0.018	6.95E-11		0.988
B	41		T	A	0.276	0.111	0.018	1.29E-09		0.977
B	45	K	P	A	0.257	0.111	0.019	3.52E-09		0.975
B	45	KG	P	A	0.259	0.111	0.019	3.81E-09	3.84E-08	0.968
B	45	KM	P	A	0.383	0.094	0.017	3.25E-08		0.963

B	45	ET	P	A	0.615	-0.094	0.017	3.39E-08		0.957
B	63		E	N	0.480	0.105	0.017	1.78E-09		0.869
B	67	F	P	A	0.246	-0.138	0.020	7.87E-12	3.79E-10	0.881
B	67	FM	P	A	0.301	-0.121	0.018	3.81E-11		0.950
B	67	SC	P	A	0.528	0.096	0.017	1.94E-08		0.924
B	67	YF	P	A	0.416	-0.096	0.018	5.27E-08		0.873
B	67	YS	P	A	0.573	0.092	0.017	7.57E-08		0.932
B	67	FC	P	A	0.371	-0.095	0.018	1.45E-07		0.875
B	67	S	P	A	0.403	0.086	0.017	8.36E-07		0.903
B	67	SM	P	A	0.458	0.081	0.018	4.74E-06		0.855
B	80	T	P	A	0.236	0.100	0.023	1.06E-05		0.675
B	97	SW	P	A	0.318	-0.084	0.019	5.69E-06	2.54E-04	0.908
B	97	S	P	A	0.313	-0.083	0.019	7.85E-06		0.926
B	97	SWV	P	A	0.355	-0.077	0.018	1.99E-05		0.919
B	97	SV	P	A	0.350	-0.076	0.018	2.43E-05		0.924
B	113	H	P	A	0.712	-0.085	0.020	1.61E-05		0.798
B	113	Y	P	A	0.269	0.083	0.019	1.97E-05		0.877
B	116	YFS	P	A	0.707	-0.096	0.020	1.16E-06	5.88E-05	0.769
B	116	DL	P	A	0.275	0.094	0.020	1.57E-06		0.843
B	116	Dx	P	A	0.219	0.111	0.024	4.38E-06		0.596
B	116	D	P	A	0.201	0.109	0.024	5.23E-06		0.675
B	116	YS	P	A	0.606	-0.083	0.020	3.52E-05		0.648
B	143	S	P	A	0.073	0.207	0.045	4.00E-06		0.416
B	143	T	P	A	0.909	-0.199	0.044	5.80E-06		0.320
B	147	L	P	A	0.073	0.208	0.045	3.94E-06		0.415
B	147	W	P	A	0.909	-0.199	0.044	5.79E-06		0.319
B	156	DW	P	A	0.311	-0.096	0.022	1.72E-05	0.41	0.529
B	156	Dx	P	A	0.287	-0.097	0.023	1.94E-05		0.529
B	156	D	P	A	0.268	-0.094	0.022	2.59E-05		0.587
B	163	EL	P	A	0.701	0.119	0.020	1.75E-09	5.6E-08	0.804
B	163	T	P	A	0.280	-0.116	0.019	2.78E-09		0.881
B	163	Ex	P	A	0.305	0.082	0.020	4.58E-05		0.747
C	66		N	K	0.201	-0.114	0.021	3.04E-08		0.998
C	156	Q	P	A	0.043	0.221	0.041	9.05E-08	1.26E-06	0.918
C	156	DQ	P	A	0.060	0.154	0.035	1.10E-05		0.938
C	156	WQ	P	A	0.207	0.085	0.020	2.79E-05		0.997
C	193	P	P	A	0.954	-0.205	0.038	8.07E-08		0.995
C	193	L	P	A	0.046	0.204	0.038	9.05E-08		0.998
DPA1	31		Q	M	0.174	-0.091	0.022	2.64E-05		0.987
DPA1	50		R	Q	0.174	-0.092	0.022	2.32E-05		0.987

DPA1	83		A	T	0.175	-0.090	0.022	3.12E-05		0.986
DPA1	111	R	P	A	0.174	-0.091	0.022	2.64E-05		0.987
DPA1	111	K	P	A	0.825	0.090	0.022	3.53E-05		0.986
DPA1	127	P	P	A	0.174	-0.092	0.022	2.31E-05		0.986
DPA1	127	L	P	A	0.825	0.090	0.022	3.11E-05		0.986
DPA1	160	V	P	A	0.174	-0.092	0.022	2.32E-05		0.987
DPA1	160	F	P	A	0.825	0.090	0.022	3.14E-05		0.986
DPA1	228	P	P	A	0.174	-0.092	0.022	2.34E-05		0.986
DPA1	228	T	P	A	0.825	0.090	0.022	3.15E-05		0.987
DQA1	-16	M	P	A	0.580	0.112	0.017	1.53E-11		0.998
DQA1	-16	L	P	A	0.420	-0.112	0.017	1.66E-11		0.999
DQA1	11	C	P	A	0.396	-0.148	0.017	1.88E-18		0.999
DQA1	11	Y	P	A	0.604	0.148	0.017	1.88E-18		0.999
DQA1	18		F	S	0.396	-0.148	0.017	1.89E-18		0.999
DQA1	25		F	Y	0.310	0.087	0.019	3.98E-06		0.858
DQA1	26		S	T	0.204	0.164	0.020	6.12E-16		0.980
DQA1	34		E	Q	0.484	0.184	0.016	1.87E-29		0.989
DQA1	40		G	E	0.264	-0.117	0.019	4.09E-10		0.995
DQA1	41		K	R	0.171	-0.114	0.024	1.59E-06		0.816
DQA1	45		A	V	0.396	-0.148	0.017	1.85E-18		0.999
DQA1	47	RC	P	A	0.660	-0.252	0.017	2.71E-49		0.996
DQA1	47	K	P	A	0.136	0.262	0.024	6.18E-28		0.979
DQA1	47	R	P	A	0.395	-0.148	0.017	1.83E-18	6.10E-50	0.997
DQA1	47	Q	P	A	0.204	0.164	0.020	6.12E-16		0.980
DQA1	47	C	P	A	0.264	-0.117	0.019	4.12E-10		0.996
DQA1	48		W	L	0.396	-0.148	0.017	1.85E-18		0.999
DQA1	50	L	P	A	0.340	0.252	0.017	2.70E-49		0.996
DQA1	50	E	P	A	0.396	-0.148	0.017	1.84E-18	1.54E-48	0.998
DQA1	50	V	P	A	0.264	-0.117	0.019	4.13E-10		0.995
DQA1	51		L	F	0.264	-0.116	0.019	4.13E-10		0.996
DQA1	52	H	P	A	0.136	0.262	0.024	6.18E-28		0.979
DQA1	52	S	P	A	0.396	-0.148	0.017	1.85E-18		0.999
DQA1	53	R	P	A	0.340	0.252	0.017	2.70E-49		0.996
DQA1	53	K	P	A	0.396	-0.148	0.017	1.85E-18	1.54E-48	0.999
DQA1	53	Q	P	A	0.264	-0.117	0.019	4.13E-10		0.995
DQA1	54		L	F	0.136	0.262	0.024	6.18E-28		0.979
DQA1	55		G	R	0.396	-0.148	0.017	1.84E-18		0.998
DQA1	56	G	P	A	0.396	-0.148	0.017	1.84E-18		0.998
DQA1	56	R	P	A	0.204	0.164	0.020	6.12E-16		0.980
DQA1	61		G	F	0.396	-0.148	0.017	1.85E-18		0.999

DQA1	64		R	T	0.396	-0.148	0.017	1.84E-18		0.998
DQA1	66		M	I	0.396	-0.148	0.017	1.85E-18		0.999
DQA1	69	A	P	A	0.396	-0.148	0.017	1.85E-18		0.999
DQA1	69	L	P	A	0.580	0.112	0.017	1.54E-11	2.92E-23	0.998
DQA1	69	T	P	A	0.024	0.327	0.053	1.04E-09		0.996
DQA1	75		S	I	0.240	-0.167	0.019	4.32E-18		0.995
DQA1	76	M	P	A	0.396	-0.148	0.017	1.85E-18		0.998
DQA1	76	V	P	A	0.204	0.164	0.020	6.12E-16		0.980
DQA1	80		Y	S	0.396	-0.148	0.017	1.85E-18		0.999
DQA1	107	I	P	A	0.240	-0.167	0.019	4.42E-18		0.995
DQA1	107	T	P	A	0.758	0.166	0.019	6.60E-18		0.986
DQA1	129	H	P	A	0.776	0.141	0.021	2.36E-11		0.831
DQA1	129	Q	P	A	0.224	-0.141	0.021	2.36E-11		0.831
DQA1	130	A	P	A	0.172	-0.114	0.024	1.57E-06		0.818
DQA1	130	S	P	A	0.829	0.114	0.024	1.66E-06		0.817
DQA1	156	F	P	A	0.760	0.167	0.019	4.55E-18		0.994
DQA1	156	L	P	A	0.240	-0.167	0.019	4.81E-18		0.994
DQA1	161	D	P	A	0.760	0.167	0.019	4.75E-18		0.995
DQA1	161	E	P	A	0.240	-0.167	0.019	4.86E-18		0.994
DQA1	163	I	P	A	0.760	0.167	0.019	4.84E-18		0.994
DQA1	163	S	P	A	0.240	-0.167	0.019	4.85E-18		0.995
**DQA1**	**175**	**E**	**P**	**A**	**0.364**	**0.279**	**0.017**	**1.14E-61**		**0.996**
DQA1	175	QK	P	A	0.636	-0.279	0.017	1.15E-61		0.996
DQA1	175	Qx	P	A	0.396	-0.148	0.017	1.99E-18	**8.35E-62**	0.999
DQA1	175	Q	P	A	0.396	-0.148	0.017	2.03E-18		0.999
DQA1	175	K	P	A	0.240	-0.167	0.019	4.93E-18		0.995
DQA1	175	QE	P	A	0.760	0.166	0.019	6.20E-18		0.995
DQA1	187	T	P	A	0.206	0.166	0.020	2.23E-16		0.971
DQA1	187	A	P	A	0.795	-0.165	0.020	4.95E-16		0.971
DQA1	207	V	P	A	0.915	0.186	0.036	2.24E-07		0.593
DQA1	207	M	P	A	0.085	-0.186	0.036	2.31E-07		0.593
DQA1	215	L	P	A	0.339	0.251	0.017	1.28E-48		0.996
DQA1	215	F	P	A	0.661	-0.251	0.017	1.30E-48		0.996
DQA1	218	R	P	A	0.603	0.146	0.017	4.43E-18		0.999
DQA1	218	Q	P	A	0.397	-0.146	0.017	5.20E-18		0.999
DQB1	-4	V	P	A	0.607	0.144	0.017	1.27E-17		0.997
DQB1	-4	L	P	A	0.361	-0.135	0.017	6.83E-15		0.976
DQB1	-5	P	P	A	0.607	0.144	0.017	1.27E-17		0.996
DQB1	-5	Px	P	A	0.639	0.135	0.017	6.81E-15	5.15E-16	0.976
DQB1	-5	PS	P	A	0.757	0.121	0.019	1.66E-10		0.997

DQB1	-5	L	P	A	0.211	-0.114	0.020	1.70E-08		0.970
DQB1	-5	PL	P	A	0.819	0.107	0.022	8.85E-07		0.959
DQB1	-6	TA	P	A	0.607	0.145	0.017	1.25E-17	8.03E-17	0.996
DQB1	-6	T	P	A	0.605	0.142	0.017	5.73E-17		0.988
DQB1	-6	S	P	A	0.361	-0.135	0.017	6.83E-15		0.976
DQB1	-6	Tx	P	A	0.637	0.133	0.017	2.88E-14		0.967
DQB1	-9	M	P	A	0.819	0.107	0.022	9.15E-07		0.959
DQB1	-21	G	P	A	0.607	0.145	0.017	1.21E-17		0.996
DQB1	-21	D	P	A	0.361	-0.135	0.017	6.83E-15		0.976
DQB1	-27	A	P	A	0.819	0.107	0.022	8.90E-07		0.959
DQB1	30	H	P	A	0.246	-0.128	0.020	1.04E-10		0.918
DQB1	30	Y	P	A	0.500	0.078	0.017	3.60E-06		0.939
DQB1	53		Q	L	0.395	-0.148	0.017	1.73E-18		0.997
DQB1	55	R	P	A	0.417	-0.108	0.017	9.17E-11		0.997
DQB1	55	P	P	A	0.329	0.103	0.017	4.07E-09		0.997
DQB1	56		L	P	0.022	0.412	0.056	1.87E-13		0.995
DQB1	57	A	P	A	0.401	0.134	0.017	3.00E-15	4.87E-14	0.982
DQB1	57	AS	P	A	0.411	0.130	0.017	1.16E-14		0.981
DQB1	57	AV	P	A	0.552	0.079	0.017	2.73E-06		0.952
DQB1	57	D	P	A	0.438	-0.078	0.017	4.74E-06		0.950
DQB1	57	AD	P	A	0.839	0.103	0.023	7.29E-06		0.937
DQB1	57	V	P	A	0.151	-0.104	0.024	1.01E-05		0.939
DQB1	70	G	P	A	0.358	-0.135	0.017	1.40E-14	3.16E-22	0.965
DQB1	70	E	P	A	0.024	0.367	0.054	8.95E-12		0.989
DQB1	70	R	P	A	0.618	0.093	0.017	7.38E-08		0.966
DQB1	71	D	P	A	0.024	0.367	0.054	8.95E-12		0.989
DQB1	84	Q	P	A	0.605	0.148	0.017	1.72E-18		0.996
DQB1	84	E	P	A	0.393	-0.147	0.017	2.96E-18		0.996
DQB1	85	L	P	A	0.605	0.148	0.017	1.72E-18		0.997
DQB1	85	V	P	A	0.393	-0.147	0.017	2.96E-18		0.996
DQB1	86	E	P	A	0.605	0.148	0.017	1.70E-18	1.19E-17	0.996
DQB1	86	Ex	P	A	0.607	0.147	0.017	2.96E-18		0.996
DQB1	86	EG	P	A	0.637	0.139	0.017	1.38E-15		0.965
DQB1	86	A	P	A	0.361	-0.138	0.017	2.33E-15		0.965
DQB1	87	L	P	A	0.605	0.148	0.017	1.70E-18		0.997
DQB1	87	Lx	P	A	0.606	0.147	0.017	2.93E-18		0.997
DQB1	87	LY	P	A	0.786	0.119	0.020	4.78E-09		0.956
DQB1	87	F	P	A	0.212	-0.118	0.020	7.05E-09		0.955
DQB1	87	LF	P	A	0.816	0.111	0.022	3.33E-07		0.945
DQB1	87	Y	P	A	0.182	-0.110	0.022	5.06E-07		0.945

DQB1	89	T	P	A	0.605	0.148	0.017	1.73E-18		0.997
DQB1	89	G	P	A	0.393	-0.147	0.017	2.97E-18		0.996
DQB1	90	T	P	A	0.605	0.148	0.017	1.73E-18		0.997
DQB1	90	I	P	A	0.394	-0.147	0.017	2.96E-18		0.997
DQB1	125	A	P	A	0.605	0.148	0.017	1.73E-18		0.998
DQB1	125	Ax	P	A	0.606	0.147	0.017	2.96E-18	1.08E-17	0.997
DQB1	125	G	P	A	0.244	-0.125	0.019	4.94E-11		0.998
DQB1	125	AS	P	A	0.752	0.124	0.019	5.13E-11		0.989
DQB1	135	G	P	A	0.100	0.272	0.027	1.27E-23		0.993
DQB1	135	D	P	A	0.898	-0.263	0.027	1.09E-22		0.991
DQB1	140	T	P	A	0.350	0.138	0.017	7.93E-16		0.997
DQB1	140	A	P	A	0.648	-0.137	0.017	1.52E-15		0.996
DQB1	182	N	P	A	0.350	0.138	0.017	7.86E-16		0.997
DQB1	182	S	P	A	0.648	-0.137	0.017	1.49E-15		0.997
DQB1	185	I	P	A	0.172	0.261	0.021	1.39E-33		0.985
DQB1	185	T	P	A	0.826	-0.257	0.021	8.05E-33		0.984
DQB1	203	I	P	A	0.606	0.144	0.017	1.77E-17		0.991
DQB1	203	V	P	A	0.391	-0.144	0.017	2.05E-17		0.997
DQB1	220	R	P	A	0.394	-0.147	0.017	2.89E-18		0.996
DQB1	220	H	P	A	0.603	0.147	0.017	4.64E-18		0.985
DQB1	221	Q	P	A	0.394	-0.147	0.017	2.90E-18		0.995
DQB1	221	H	P	A	0.604	0.147	0.017	4.26E-18		0.986
DRB1	-1	x	P	A	0.037	0.228	0.052	1.07E-05		0.684
DRB1	-16	V	P	A	0.462	-0.213	0.017	1.31E-36		0.944
DRB1	-16	A	P	A	0.501	0.178	0.016	1.40E-27	2.50E-39	0.986
DRB1	-16	x	P	A	0.037	0.228	0.052	1.07E-05		0.684
DRB1	-17	T	P	A	0.162	-0.100	0.022	9.28E-06		0.977
DRB1	-17	x	P	A	0.037	0.228	0.052	1.07E-05		0.684
DRB1	-24	L	P	A	0.775	-0.197	0.020	8.79E-23		0.937
DRB1	-24	F	P	A	0.188	0.174	0.021	5.29E-17	4.09E-25	0.997
DRB1	-24	x	P	A	0.037	0.228	0.052	1.07E-05		0.684
DRB1	-25	R	P	A	0.462	-0.213	0.017	1.31E-36		0.944
DRB1	-25	K	P	A	0.501	0.178	0.016	1.40E-27	2.50E-39	0.986
DRB1	-25	x	P	A	0.037	0.228	0.052	1.07E-05		0.684
DRB1	4	Q	P	A	0.151	0.233	0.023	6.30E-25		1.000
DRB1	4	R	P	A	0.846	-0.234	0.023	7.60E-25		0.974
DRB1	9	W	P	A	0.300	0.084	0.018	2.52E-06		0.985
DRB1	9	E	P	A	0.687	-0.080	0.018	5.74E-06		0.985
DRB1	10	Q	P	A	0.501	0.178	0.016	1.34E-27		0.987
DRB1	10	Y	P	A	0.493	-0.173	0.016	5.65E-26		0.987

DRB1	11	VG	P	A	0.332	0.251	0.017	1.59E-48		0.999
DRB1	11	SPL	P	A	0.655	-0.245	0.017	5.55E-47		0.999
DRB1	11	SPD	P	A	0.662	-0.245	0.017	3.51E-46		0.986
DRB1	11	SP	P	A	0.650	-0.239	0.017	9.87E-45		0.986
DRB1	11	SLD	P	A	0.511	-0.179	0.016	6.85E-28		0.999
DRB1	11	G	P	A	0.138	0.255	0.023	2.01E-27		1.000
DRB1	11	SL	P	A	0.499	-0.177	0.016	2.18E-27		0.999
DRB1	11	SD	P	A	0.506	-0.174	0.016	1.90E-26		0.987
DRB1	11	S	P	A	0.493	-0.173	0.016	5.65E-26		0.987
DRB1	11	GD	P	A	0.151	0.233	0.023	6.30E-25		1.000
DRB1	11	GL	P	A	0.144	0.239	0.023	8.99E-25	8.33E-48	0.974
DRB1	11	SPV	P	A	0.844	-0.220	0.022	1.49E-22		0.975
DRB1	11	V	P	A	0.194	0.161	0.020	3.68E-15		0.999
DRB1	11	VD	P	A	0.206	0.151	0.020	3.92E-14		0.999
DRB1	11	VL	P	A	0.200	0.150	0.020	1.45E-13		0.980
DRB1	11	SPG	P	A	0.788	-0.142	0.020	1.19E-12		0.981
DRB1	11	PG	P	A	0.295	0.091	0.018	3.80E-07		1.000
DRB1	11	SVL	P	A	0.693	-0.087	0.018	9.52E-07		1.000
DRB1	11	SVD	P	A	0.700	-0.084	0.018	2.52E-06		0.985
DRB1	11	SV	P	A	0.687	-0.080	0.018	5.74E-06		0.985
DRB1	11	SVG	P	A	0.826	0.097	0.022	8.35E-06		0.978
DRB1	11	PL	P	A	0.162	-0.100	0.022	9.28E-06		0.977
DRB1	12		T	K	0.493	-0.173	0.016	5.65E-26		0.987
DRB1	13	SRF	P	A	0.625	-0.276	0.017	1.25E-58		0.952
DRB1	13	SR	P	A	0.602	-0.257	0.017	6.27E-52		0.940
DRB1	13	HY	P	A	0.327	0.259	0.017	2.74E-51		0.999
DRB1	13	SRG	P	A	0.650	-0.239	0.017	9.87E-45		0.986
DRB1	13	SF	P	A	0.469	-0.211	0.017	1.00E-36		0.955
DRB1	13	S	P	A	0.445	-0.200	0.017	7.64E-33		0.942
DRB1	13	YG	P	A	0.186	0.247	0.021	3.01E-30		0.924
DRB1	13	SFG	P	A	0.517	-0.184	0.016	1.37E-29		1.000
DRB1	13	Y	P	A	0.138	0.255	0.023	2.01E-27	4.71E-58	1.000
DRB1	13	SG	P	A	0.493	-0.173	0.016	5.65E-26		0.987
DRB1	13	SRH	P	A	0.790	-0.206	0.021	2.55E-23		0.912
DRB1	13	HG	P	A	0.237	0.182	0.020	1.97E-20		0.938
DRB1	13	YF	P	A	0.162	0.204	0.022	4.38E-20		0.976
DRB1	13	H	P	A	0.189	0.173	0.021	5.36E-17		0.999
DRB1	13	SRY	P	A	0.740	-0.154	0.019	6.74E-16		0.926
DRB1	13	HF	P	A	0.212	0.142	0.020	1.19E-12		0.981
DRB1	13	SHF	P	A	0.657	-0.108	0.018	8.53E-10		0.950

DRB1	13	SH	P	A	0.634	-0.091	0.017	1.68E-07		0.938
DRB1	13	RY	P	A	0.295	0.091	0.018	3.80E-07		1.000
DRB1	13	SYF	P	A	0.607	-0.086	0.017	5.36E-07		0.953
DRB1	13	RF	P	A	0.180	-0.105	0.022	1.26E-06		0.978
DRB1	13	SY	P	A	0.584	-0.071	0.017	3.08E-05		0.941
DRB1	13	SHG	P	A	0.682	-0.073	0.018	3.33E-05		0.985
DRB1	14		K	E	0.138	0.255	0.023	2.01E-27		1.000
DRB1	25		Q	R	0.138	0.255	0.023	2.01E-27		1.000
DRB1	26	F	P	A	0.837	0.255	0.032	1.13E-15		0.343
DRB1	26	L	P	A	0.026	-0.249	0.059	2.53E-05		0.719
DRB1	28	E	P	A	0.175	0.192	0.023	3.20E-17		0.876
DRB1	28	D	P	A	0.812	-0.177	0.022	9.15E-16		0.882
DRB1	30	L	P	A	0.138	0.255	0.023	2.01E-27		1.000
DRB1	30	LG	P	A	0.151	0.233	0.023	6.30E-25		1.000
DRB1	30	LC	P	A	0.144	0.239	0.023	8.99E-25		0.974
DRB1	30	LR	P	A	0.144	0.236	0.023	1.97E-24		0.999
DRB1	30	YHR	P	A	0.844	-0.220	0.022	1.49E-22		0.975
DRB1	30	LH	P	A	0.161	0.225	0.023	1.67E-22		0.910
DRB1	30	YCH	P	A	0.844	-0.217	0.022	2.93E-22		1.000
DRB1	30	YHG	P	A	0.850	-0.221	0.023	5.14E-22		0.974
DRB1	30	YCR	P	A	0.826	-0.208	0.022	1.48E-20		0.916
DRB1	30	YGR	P	A	0.833	-0.212	0.023	3.04E-20	8.34E-23	0.890
DRB1	30	YH	P	A	0.838	-0.204	0.022	4.38E-20		0.976
DRB1	30	YCG	P	A	0.833	-0.208	0.023	5.98E-20		0.913
DRB1	30	YR	P	A	0.821	-0.195	0.022	1.64E-18		0.896
DRB1	30	YC	P	A	0.821	-0.192	0.022	2.94E-18		0.918
DRB1	30	YG	P	A	0.827	-0.195	0.023	6.89E-18		0.893
DRB1	30	Y	P	A	0.815	-0.180	0.022	2.18E-16		0.898
DRB1	30	YLG	P	A	0.966	0.280	0.059	2.16E-06		0.548
DRB1	30	CH	P	A	0.029	-0.286	0.069	3.45E-05		0.462
DRB1	30	YL	P	A	0.954	0.192	0.047	4.46E-05		0.661
DRB1	32		H	Y	0.392	-0.247	0.019	3.18E-37		0.734
DRB1	33		H	N	0.189	0.173	0.021	5.33E-17		0.999
DRB1	37	YF	P	A	0.455	0.291	0.019	1.64E-53		0.703
DRB1	37	NS	P	A	0.530	-0.280	0.019	1.39E-49		0.695
DRB1	37	NL	P	A	0.376	-0.242	0.020	6.88E-34		0.675
DRB1	37	N	P	A	0.361	-0.235	0.020	1.93E-31	4.19E-57	0.664
DRB1	37	F	P	A	0.155	0.238	0.023	4.56E-24		0.898
DRB1	37	FL	P	A	0.170	0.208	0.023	7.82E-20		0.885
DRB1	37	Y	P	A	0.301	0.165	0.021	3.69E-15		0.681

DRB1	37	YL	P	A	0.316	0.149	0.021	8.33E-13		0.674
DRB1	37	SL	P	A	0.184	-0.112	0.022	4.65E-07		0.911
DRB1	37	S	P	A	0.169	-0.102	0.023	7.38E-06		0.934
DRB1	37	SF	P	A	0.323	0.077	0.018	2.36E-05		0.898
DRB1	47		F	Y	0.545	-0.290	0.018	1.10E-54		0.750
DRB1	57	DA	P	A	0.781	-0.257	0.021	1.34E-32		0.821
DRB1	57	D	P	A	0.766	-0.242	0.021	3.15E-29		0.774
DRB1	57	V	P	A	0.166	0.209	0.022	4.73E-21	9.97E-25	0.969
DRB1	57	DS	P	A	0.819	-0.192	0.022	3.41E-18		0.895
DRB1	57	S	P	A	0.053	0.318	0.051	3.51E-10		0.481
DRB1	57	DV	P	A	0.931	-0.216	0.048	7.28E-06		0.407
DRB1	60	S	P	A	0.166	0.209	0.022	4.73E-21		0.969
DRB1	60	Y	P	A	0.819	-0.192	0.022	3.41E-18		0.895
DRB1	71	R	P	A	0.392	0.297	0.025	1.11E-31		0.351
DRB1	71	KR	P	A	0.632	0.256	0.022	1.92E-30	2.38E-32	0.478
DRB1	71	E	P	A	0.241	-0.238	0.025	7.50E-21		0.445
DRB1	71	KE	P	A	0.481	-0.152	0.022	4.03E-12		0.480
DRB1	73		G	A	0.259	0.151	0.023	5.00E-11		0.542
DRB1	74	QL	P	A	0.164	0.279	0.022	1.24E-36		0.988
DRB1	74	RA	P	A	0.794	-0.268	0.022	9.57E-35		0.835
DRB1	74	Q	P	A	0.138	0.255	0.023	2.01E-27		1.000
DRB1	74	QE	P	A	0.180	0.240	0.023	1.65E-25		0.825
DRB1	74	AE	P	A	0.715	-0.194	0.022	2.00E-18		0.558
DRB1	74	A	P	A	0.674	-0.182	0.022	4.98E-17	1.36E-35	0.554
DRB1	74	RQ	P	A	0.259	0.151	0.023	5.00E-11		0.542
DRB1	74	RE	P	A	0.162	-0.211	0.034	5.16E-10		0.321
DRB1	74	AL	P	A	0.700	-0.139	0.022	5.55E-10		0.540
DRB1	74	L	P	A	0.026	0.304	0.053	9.28E-09		0.938
DRB1	74	LE	P	A	0.068	0.175	0.042	2.88E-05		0.573
DRB1	78		V	Y	0.151	0.233	0.023	6.30E-25		1.000
DRB1	86		V	G	0.477	-0.329	0.025	2.49E-40		0.364
DRB1	96	Y	P	A	0.188	0.173	0.021	6.80E-17		0.997
DRB1	96	Yx	P	A	0.201	0.173	0.021	1.67E-16		0.923
DRB1	96	YE	P	A	0.213	0.160	0.021	7.24E-15		0.880
DRB1	96	HQ	P	A	0.774	-0.157	0.021	3.13E-14	1.91E-17	0.822
DRB1	96	HY	P	A	0.801	0.118	0.023	1.82E-07		0.796
DRB1	96	QE	P	A	0.186	-0.112	0.023	6.15E-07		0.864
DRB1	96	Qx	P	A	0.175	-0.105	0.023	3.13E-06		0.910
DRB1	96	Q	P	A	0.162	-0.098	0.022	1.17E-05		0.999
DRB1	98	K	P	A	0.648	-0.258	0.017	5.79E-50		0.946

DRB1	98	E	P	A	0.339	0.252	0.017	1.75E-49		0.998
DRB1	104	S	P	A	0.638	-0.261	0.017	4.45E-50		0.906
DRB1	104	A	P	A	0.339	0.252	0.017	1.75E-49		0.998
DRB1	120	N	P	A	0.194	0.161	0.020	4.61E-15		0.997
DRB1	120	S	P	A	0.781	-0.158	0.021	3.67E-14		0.858
DRB1	133	R	P	A	0.818	0.104	0.023	6.34E-06		0.834
DRB1	140	A	P	A	0.331	0.083	0.018	4.87E-06		0.907
DRB1	140	T	P	A	0.643	-0.078	0.018	2.08E-05		0.815
DRB1	142	V	P	A	0.818	0.104	0.023	6.34E-06		0.834
DRB1	149	H	P	A	0.449	-0.188	0.017	5.18E-27		0.837
DRB1	149	Q	P	A	0.525	0.181	0.017	6.38E-27		0.919
DRB1	180	V	P	A	0.752	-0.202	0.021	1.55E-22		0.780
DRB1	180	L	P	A	0.196	0.173	0.021	9.86E-17	3.22E-24	0.951
DRB1	180	x	P	A	0.044	0.299	0.061	1.08E-06		0.320
DRB1	181	T	P	A	0.789	-0.243	0.022	1.22E-28		0.766
DRB1	181	M	P	A	0.163	0.217	0.022	5.26E-22	1.85E-33	0.949
DRB1	181	x	P	A	0.044	0.298	0.061	1.14E-06		0.318
DRB1	189	x	P	A	0.043	0.219	0.052	2.33E-05		0.570
DRB1	231	x	P	A	0.039	0.222	0.052	1.98E-05		0.634
DRB1	233	R	P	A	0.335	-0.208	0.018	1.15E-31		0.954
DRB1	233	T	P	A	0.626	0.168	0.017	9.40E-23	5.76E-34	0.985
DRB1	233	x	P	A	0.039	0.222	0.052	1.98E-05		0.634

Table S19: Associations for HLA amino acid (AA) sequence substitutions that were significantly associated (P<4.6×10^{-5}) with seroreactivity to HSV1. At each position that contained a significantly associated allele and >2 possible substitutions, we tested all common (frequency ≥0.01) haplotypes at that position using the omnibus test (P_{omni}) in the conditional haplotype module of PLINK 1.07. Variants and haplotypes with the lowest p-value are indicated in bold and shaded.

Gene	AA position	AA	A1	A2	FRQ	BETA	SE	P	P_{omni}	INFO
DQA1	-16	L	P	A	0.420	-0.097	0.019	3.77E-07		0.999
DQA1	-16	M	P	A	0.580	0.097	0.019	4.02E-07		0.998
DQA1	11	Y	P	A	0.604	0.101	0.019	1.57E-07		0.999
DQA1	11	C	P	A	0.396	-0.101	0.019	1.57E-07		0.999
DQA1	18		F	S	0.396	-0.101	0.019	1.57E-07		0.999
DQA1	41		K	R	0.171	-0.120	0.027	7.52E-06		0.816
DQA1	45		A	V	0.396	-0.101	0.019	1.58E-07		0.999
DQA1	47	R	P	A	0.395	-0.101	0.019	1.60E-07		0.997
DQA1	47	RK	P	A	0.531	-0.090	0.019	1.91E-06		0.988
DQA1	48		W	L	0.396	-0.101	0.019	1.57E-07		0.999
DQA1	50	E	P	A	0.396	-0.101	0.019	1.59E-07		0.998
DQA1	52	S	P	A	0.396	-0.101	0.019	1.58E-07		0.999
DQA1	52	R	P	A	0.468	0.090	0.019	1.88E-06		0.989
DQA1	53	K	P	A	0.396	-0.101	0.019	1.57E-07		0.999
DQA1	55		G	R	0.396	-0.101	0.019	1.59E-07		0.998
DQA1	56	G	P	A	0.396	-0.101	0.019	1.59E-07		0.998
DQA1	61		G	F	0.396	-0.101	0.019	1.57E-07		0.999
DQA1	64		R	T	0.396	-0.101	0.019	1.59E-07		0.998
DQA1	66		M	I	0.396	-0.101	0.019	1.57E-07		0.999
DQA1	69	A	P	A	0.396	-0.101	0.019	1.57E-07		0.999
DQA1	69	L	P	A	0.580	0.097	0.019	3.82E-07		0.998
DQA1	76	M	P	A	0.396	-0.101	0.019	1.58E-07		0.998
DQA1	80		Y	S	0.396	-0.101	0.019	1.57E-07		0.999
DQA1	130	A	P	A	0.172	-0.120	0.027	7.46E-06		0.818
DQA1	130	S	P	A	0.829	0.120	0.027	8.26E-06		0.817
DQA1	175	Qx	P	A	0.396	-0.101	0.019	1.77E-07		0.999
DQA1	175	Q	P	A	0.396	-0.101	0.019	1.77E-07		0.999
DQA1	218	R	P	A	0.603	0.100	0.019	2.09E-07		0.999
DQA1	218	Q	P	A	0.397	-0.100	0.019	2.34E-07		0.999
DQB1	-4	V	P	A	0.607	0.098	0.019	4.43E-07		0.997
DQB1	-4	L	P	A	0.361	-0.086	0.020	1.45E-05		0.976
DQB1	-5	P	P	A	0.607	0.098	0.019	4.42E-07	1.51E-06	0.996
DQB1	-5	PS	P	A	0.757	0.109	0.022	4.46E-07		0.997

DQB1	-5	L	P	A	0.211	-0.100	0.023	1.17E-05		0.970
DQB1	-5	Px	P	A	0.639	0.086	0.020	1.45E-05		0.976
DQB1	-6	T	P	A	0.605	0.098	0.019	4.19E-07		0.988
DQB1	-6	TA	P	A	0.607	0.098	0.019	4.44E-07	1.80E-06	0.996
DQB1	-6	Tx	P	A	0.637	0.087	0.020	1.41E-05		0.967
DQB1	-6	S	P	A	0.361	-0.086	0.020	1.45E-05		0.976
DQB1	-10	S	P	A	0.254	0.112	0.022	2.31E-07		0.999
DQB1	-10	A	P	A	0.714	-0.087	0.021	3.60E-05		0.974
DQB1	-21	G	P	A	0.607	0.098	0.019	4.43E-07		0.996
DQB1	-21	D	P	A	0.361	-0.086	0.020	1.45E-05		0.976
DQB1	9	Y	P	A	0.833	0.107	0.026	3.57E-05	4.70E-05	0.913
DQB1	9	Yx	P	A	0.833	0.107	0.026	3.57E-05		0.913
DQB1	28		S	T	0.254	0.112	0.022	2.30E-07		1.000
DQB1	30	S	P	A	0.254	0.112	0.022	2.30E-07		1.000
DQB1	37	I	P	A	0.254	0.112	0.022	2.30E-07		1.000
DQB1	37	Y	P	A	0.743	-0.106	0.021	8.49E-07		0.999
DQB1	46		E	V	0.254	0.112	0.022	2.30E-07		0.999
DQB1	47		F	Y	0.254	0.112	0.022	2.30E-07		0.999
DQB1	52		L	P	0.254	0.112	0.022	2.30E-07		0.999
DQB1	53		Q	L	0.395	-0.101	0.019	1.72E-07		0.997
DQB1	55	L	P	A	0.254	0.112	0.022	2.30E-07		0.999
DQB1	55	R	P	A	0.417	-0.098	0.019	2.93E-07		0.997
**DQB1**	**57**	**A**	**P**	**A**	**0.401**	**0.123**	**0.019**	**2.20E-10**		**0.982**
DQB1	57	AS	P	A	0.411	0.116	0.019	1.59E-09	**6.50E-09**	0.981
DQB1	57	AV	P	A	0.552	0.111	0.019	8.88E-09		0.952
DQB1	57	D	P	A	0.438	-0.106	0.019	4.17E-08		0.950
DQB1	66		D	E	0.281	0.096	0.021	4.46E-06		0.998
DQB1	67		I	V	0.281	0.096	0.021	4.47E-06		0.998
DQB1	70	R	P	A	0.618	0.089	0.020	7.21E-06		0.966
DQB1	70	G	P	A	0.358	-0.087	0.020	1.35E-05		0.965
DQB1	71	K	P	A	0.254	0.112	0.022	2.30E-07	7.06E-06	0.999
DQB1	71	KD	P	A	0.278	0.101	0.021	1.38E-06		0.998
DQB1	74	A	P	A	0.254	0.112	0.022	2.30E-07		0.999
DQB1	84	Q	P	A	0.605	0.101	0.019	1.72E-07		0.996
DQB1	84	E	P	A	0.393	-0.097	0.019	6.22E-07		0.996
DQB1	85	L	P	A	0.605	0.101	0.019	1.73E-07		0.997
DQB1	85	V	P	A	0.393	-0.097	0.019	6.22E-07		0.996
DQB1	86	E	P	A	0.605	0.101	0.019	1.73E-07		0.996
DQB1	86	Ex	P	A	0.607	0.097	0.019	6.21E-07	1.30E-06	0.996
DQB1	86	EG	P	A	0.637	0.096	0.020	1.63E-06		0.965

DQB1	86	A	P	A	0.361	-0.091	0.020	5.53E-06		0.965
DQB1	87	L	P	A	0.605	0.101	0.019	1.72E-07	2.90E-07	0.997
DQB1	87	Lx	P	A	0.606	0.097	0.019	6.21E-07		0.997
DQB1	87	LY	P	A	0.786	0.112	0.023	1.01E-06		0.956
DQB1	87	F	P	A	0.212	-0.106	0.023	3.83E-06		0.955
DQB1	89	T	P	A	0.605	0.101	0.019	1.72E-07		0.997
DQB1	89	G	P	A	0.393	-0.097	0.019	6.21E-07		0.996
DQB1	90	T	P	A	0.605	0.101	0.019	1.72E-07		0.997
DQB1	90	I	P	A	0.394	-0.097	0.019	6.19E-07		0.997
DQB1	125	AS	P	A	0.752	0.113	0.021	1.59E-07	1.54E-07	0.989
DQB1	125	A	P	A	0.605	0.101	0.019	1.71E-07		0.998
DQB1	125	Ax	P	A	0.606	0.097	0.019	6.16E-07		0.997
DQB1	125	G	P	A	0.244	-0.107	0.022	6.50E-07		0.998
DQB1	203	I	P	A	0.606	0.098	0.019	4.21E-07		0.991
DQB1	203	V	P	A	0.391	-0.093	0.019	1.60E-06		0.997
DQB1	220	H	P	A	0.603	0.099	0.019	3.14E-07		0.985
DQB1	220	R	P	A	0.394	-0.097	0.019	5.62E-07		0.996
DQB1	221	H	P	A	0.604	0.099	0.019	3.83E-07		0.986
DQB1	221	Q	P	A	0.394	-0.097	0.019	5.24E-07		0.995
DRB1	-1	S	P	A	0.149	-0.115	0.026	1.15E-05		0.994
DRB1	-17	T	P	A	0.162	-0.118	0.026	4.53E-06		0.977
DRB1	-17	A	P	A	0.801	0.101	0.024	3.91E-05		0.911
DRB1	11	PL	P	A	0.162	-0.118	0.026	4.53E-06	6.31E-04	0.977
DRB1	11	P	P	A	0.156	-0.114	0.026	1.00E-05		1.000
DRB1	11	SVG	P	A	0.826	0.105	0.025	2.59E-05		0.978
DRB1	13	RG	P	A	0.204	-0.113	0.024	2.05E-06	5.68E-04	0.932
DRB1	13	SHY	P	A	0.772	0.103	0.023	7.69E-06		0.919
DRB1	13	R	P	A	0.156	-0.114	0.026	1.00E-05		1.000
DRB1	13	RF	P	A	0.180	-0.101	0.025	4.14E-05		0.978
DRB1	37	SL	P	A	0.184	-0.130	0.025	2.79E-07	1.13E-04	0.911
DRB1	37	S	P	A	0.169	-0.118	0.026	4.82E-06		0.934
DRB1	71	A	P	A	0.127	-0.140	0.032	9.94E-06		0.785
DRB1	96	QE	P	A	0.186	-0.115	0.026	7.48E-06	1.51E-04	0.864
DRB1	96	HY	P	A	0.801	0.115	0.026	7.85E-06		0.796
DRB1	96	Qx	P	A	0.175	-0.112	0.026	1.44E-05		0.910
DRB1	96	Q	P	A	0.162	-0.109	0.025	1.70E-05		0.999
DRB1	133	R	P	A	0.818	0.117	0.026	7.98E-06		0.834
DRB1	133	L	P	A	0.156	-0.114	0.026	9.84E-06		0.999
DRB1	142	V	P	A	0.818	0.117	0.026	7.98E-06		0.834
DRB1	142	M	P	A	0.156	-0.114	0.026	9.84E-06		0.999

Table S20: Associations for HLA amino acid (AA) sequence substitutions that were significantly associated ($P<4.6\times10^{-5}$) with seroreactivity to JCV. At each position that contained a significantly associated allele and >2 possible substitutions, we tested all common (frequency ≥0.01) haplotypes at that position using the omnibus test (P_{omni}) in the conditional haplotype module of PLINK 1.07. Variants and haplotypes with the lowest p-value are indicated in bold and shaded.

Gene	AA position	AA	A1	A2	FRQ	BETA	SE	P	P_{omni}	INFO
DQA1	-16	M	P	A	0.580	0.130	0.021	1.09E-09		0.998
DQA1	-16	L	P	A	0.420	-0.129	0.021	1.42E-09		0.999
DQA1	11	C	P	A	0.396	-0.134	0.022	6.73E-10		0.999
DQA1	11	Y	P	A	0.604	0.134	0.022	6.73E-10		0.999
DQA1	18		F	S	0.396	-0.134	0.022	6.72E-10		0.999
DQA1	41		K	R	0.171	-0.181	0.031	6.86E-09		0.816
DQA1	45		A	V	0.396	-0.134	0.022	6.78E-10		0.999
DQA1	47	R	P	A	0.395	-0.134	0.022	6.96E-10		0.997
DQA1	47	RK	P	A	0.531	-0.111	0.021	2.28E-07	5.78E-09	0.988
DQA1	47	RC	P	A	0.660	-0.089	0.021	3.73E-05		0.996
DQA1	48		W	L	0.396	-0.134	0.022	6.71E-10		0.999
DQA1	50	E	P	A	0.396	-0.134	0.022	6.86E-10		0.998
DQA1	50	L	P	A	0.340	0.089	0.021	3.73E-05		0.996
DQA1	52	S	P	A	0.396	-0.134	0.022	6.80E-10		0.999
DQA1	52	R	P	A	0.468	0.111	0.021	2.22E-07		0.989
DQA1	53	K	P	A	0.396	-0.134	0.022	6.70E-10		0.999
DQA1	53	R	P	A	0.340	0.089	0.021	3.73E-05		0.996
DQA1	55		G	R	0.396	-0.134	0.022	6.85E-10		0.998
DQA1	56	G	P	A	0.396	-0.134	0.022	6.85E-10		0.998
DQA1	61		G	F	0.396	-0.134	0.022	6.70E-10		0.999
DQA1	64		R	T	0.396	-0.134	0.022	6.86E-10		0.998
DQA1	66		M	I	0.396	-0.134	0.022	6.70E-10		0.999
DQA1	69	A	P	A	0.396	-0.134	0.022	6.70E-10		0.999
DQA1	69	L	P	A	0.580	0.129	0.021	1.35E-09		0.998
DQA1	76	M	P	A	0.396	-0.134	0.022	6.77E-10		0.998
DQA1	80		Y	S	0.396	-0.134	0.022	6.70E-10		0.999
DQA1	130	A	P	A	0.172	-0.181	0.031	6.86E-09		0.818
DQA1	130	S	P	A	0.829	0.181	0.031	6.88E-09		0.817
DQA1	175	Qx	P	A	0.396	-0.134	0.022	6.53E-10		0.999
DQA1	175	Q	P	A	0.396	-0.134	0.022	6.54E-10	3.70E-09	0.999
DQA1	175	QK	P	A	0.636	-0.088	0.021	3.89E-05		0.996
DQA1	175	E	P	A	0.364	0.088	0.021	3.89E-05		0.996
DQA1	218	R	P	A	0.603	0.133	0.022	1.01E-09		0.999

DQA1	218	Q	P	A	0.397	-0.133	0.022	1.03E-09		0.999
DQB1	-4	L	P	A	0.361	-0.142	0.022	2.95E-10		0.976
DQB1	-4	V	P	A	0.607	0.129	0.022	2.82E-09		0.997
DQB1	-5	Px	P	A	0.639	0.142	0.022	2.95E-10	4.09E-10	0.976
DQB1	-5	L	P	A	0.211	-0.162	0.027	1.84E-09		0.97
DQB1	-5	P	P	A	0.607	0.129	0.022	2.83E-09		0.996
DQB1	-5	PS	P	A	0.757	0.135	0.025	6.41E-08		0.997
DQB1	-6	Tx	P	A	0.637	0.143	0.023	2.64E-10	1.43E-09	0.967
DQB1	-6	S	P	A	0.361	-0.142	0.022	2.95E-10		0.976
DQB1	-6	T	P	A	0.605	0.130	0.022	2.57E-09		0.988
DQB1	-6	TA	P	A	0.607	0.129	0.022	2.83E-09		0.996
DQB1	-18	V	P	A	0.534	-0.127	0.021	2.28E-09		0.971
DQB1	-18	A	P	A	0.434	0.123	0.021	6.40E-09		0.99
DQB1	-21	D	P	A	0.361	-0.142	0.022	2.95E-10		0.976
DQB1	-21	G	P	A	0.607	0.129	0.022	2.79E-09		0.996
DQB1	9	Y	P	A	0.833	0.274	0.031	1.68E-18	1.31E-18	0.913
DQB1	9	Yx	P	A	0.833	0.274	0.031	1.68E-18		0.913
DQB1	9	F	P	A	0.164	-0.269	0.031	1.43E-17		0.912
DQB1	9	YL	P	A	0.836	0.269	0.031	1.44E-17		0.912
DQB1	13	A	P	A	0.180	0.125	0.027	3.69E-06		0.984
DQB1	13	G	P	A	0.820	-0.125	0.027	3.71E-06		0.985
DQB1	26	Y	P	A	0.180	0.125	0.027	3.70E-06		0.985
DQB1	45		E	G	0.177	0.135	0.027	7.33E-07		0.985
DQB1	53		Q	L	0.395	-0.134	0.022	7.00E-10		0.997
DQB1	55	R	P	A	0.417	-0.131	0.021	1.09E-09		0.997
DQB1	70	G	P	A	0.358	-0.144	0.023	2.39E-10		0.965
DQB1	70	R	P	A	0.618	0.137	0.022	7.54E-10		0.966
DQB1	84	E	P	A	0.393	-0.136	0.022	4.21E-10		0.996
DQB1	84	Q	P	A	0.605	0.134	0.022	7.06E-10		0.996
DQB1	85	V	P	A	0.393	-0.136	0.022	4.21E-10		0.996
DQB1	85	L	P	A	0.605	0.134	0.022	7.07E-10		0.997
DQB1	86	A	P	A	0.361	-0.148	0.023	5.23E-11	5.52E-10	0.965
DQB1	86	EG	P	A	0.637	0.146	0.023	9.21E-11		0.965
DQB1	86	Ex	P	A	0.607	0.136	0.022	4.20E-10		0.996
DQB1	86	E	P	A	0.605	0.134	0.022	7.07E-10		0.996
DQB1	87	F	P	A	0.212	-0.171	0.027	2.55E-10	7.08E-11	0.955
DQB1	87	Lx	P	A	0.606	0.136	0.022	4.16E-10		0.997
DQB1	87	LY	P	A	0.786	0.167	0.027	5.35E-10		0.956
DQB1	87	L	P	A	0.605	0.134	0.022	6.99E-10		0.997
DQB1	89	G	P	A	0.393	-0.136	0.022	4.20E-10		0.996

DQB1	89	T	P	A	0.605	0.134	0.022	7.06E-10		0.997
DQB1	90	I	P	A	0.394	-0.136	0.022	4.16E-10		0.997
DQB1	90	T	P	A	0.605	0.134	0.022	6.99E-10		0.997
DQB1	125	Ax	P	A	0.606	0.136	0.022	4.17E-10		0.997
DQB1	125	A	P	A	0.605	0.134	0.022	6.99E-10	1.16E-09	0.998
DQB1	125	G	P	A	0.244	-0.143	0.025	8.92E-09		0.998
DQB1	125	AS	P	A	0.752	0.141	0.025	1.62E-08		0.989
DQB1	167	R	P	A	0.817	-0.127	0.027	2.18E-06		0.987
DQB1	167	H	P	A	0.181	0.125	0.027	3.24E-06		0.988
DQB1	203	V	P	A	0.391	-0.131	0.022	1.71E-09		0.997
DQB1	203	I	P	A	0.606	0.130	0.022	2.79E-09		0.991
DQB1	220	R	P	A	0.394	-0.135	0.022	4.57E-10		0.996
DQB1	220	H	P	A	0.603	0.134	0.022	7.30E-10		0.985
DQB1	221	Q	P	A	0.394	-0.135	0.022	4.86E-10		0.995
DQB1	221	H	P	A	0.604	0.135	0.022	5.85E-10		0.986
DRB1	-1	S	P	A	0.149	-0.310	0.032	9.03E-22		0.994
DRB1	-1	A	P	A	0.814	0.252	0.030	3.26E-17		0.921
DRB1	-17	T	P	A	0.162	-0.315	0.031	1.32E-23		0.977
DRB1	-17	A	P	A	0.801	0.260	0.029	5.41E-19		0.911
DRB1	-24	L	P	A	0.775	-0.110	0.026	1.74E-05		0.937
DRB1	9	E	P	A	0.687	0.154	0.023	4.64E-11		0.985
DRB1	9	W	P	A	0.300	-0.155	0.024	6.88E-11		0.985
DRB1	11	PL	P	A	0.162	-0.315	0.031	1.32E-23		0.977
DRB1	11	SVG	P	A	0.826	0.297	0.030	7.61E-23		0.978
DRB1	11	P	P	A	0.156	-0.312	0.032	8.40E-23		1
DRB1	11	PD	P	A	0.168	-0.294	0.030	4.84E-22		1
DRB1	11	SV	P	A	0.687	0.154	0.023	4.64E-11	8.68E-21	0.985
DRB1	11	SVD	P	A	0.700	0.155	0.024	6.88E-11		0.985
DRB1	11	SVL	P	A	0.693	0.150	0.023	1.77E-10		1
DRB1	11	PG	P	A	0.295	-0.151	0.024	2.61E-10		1
DRB1	11	VG	P	A	0.332	0.088	0.022	4.56E-05		0.999
DRB1	13	RF	P	A	0.180	-0.297	0.030	2.15E-23		0.978
DRB1	13	R	P	A	0.156	-0.312	0.032	8.40E-23		1
DRB1	13	SHY	P	A	0.772	0.242	0.027	7.94E-19		0.919
DRB1	13	RG	P	A	0.204	-0.244	0.028	1.14E-17		0.932
DRB1	13	SHG	P	A	0.682	0.158	0.023	1.28E-11	2.01E-21	0.985
DRB1	13	SH	P	A	0.634	0.147	0.023	1.47E-10		0.938
DRB1	13	RY	P	A	0.295	-0.151	0.024	2.61E-10		1
DRB1	13	SHF	P	A	0.657	0.137	0.023	3.06E-09		0.95
DRB1	13	SRF	P	A	0.625	-0.097	0.022	7.80E-06		0.952

DRB1	13	HY	P	A	0.327	0.093	0.022	1.86E-05		0.999
DRB1	13	HG	P	A	0.237	0.103	0.025	4.04E-05		0.938
DRB1	37	S	P	A	0.169	-0.321	0.031	2.90E-24		0.934
DRB1	37	SL	P	A	0.184	-0.303	0.031	8.26E-23		0.911
DRB1	37	SF	P	A	0.323	-0.160	0.024	4.56E-11		0.898
DRB1	37	NY	P	A	0.662	0.156	0.024	9.94E-11	2.69E-21	0.889
DRB1	37	NS	P	A	0.530	-0.131	0.024	6.58E-08		0.695
DRB1	37	YF	P	A	0.455	0.131	0.024	7.87E-08		0.703
DRB1	37	YL	P	A	0.316	0.126	0.027	2.98E-06		0.674
DRB1	37	Y	P	A	0.301	0.127	0.027	3.10E-06		0.681
DRB1	67	L	P	A	0.344	0.121	0.027	9.71E-06		0.524
DRB1	71	A	P	A	0.127	-0.384	0.039	1.04E-22		0.785
DRB1	71	KE	P	A	0.481	0.174	0.029	1.43E-09	4.59E-22	0.48
DRB1	71	KR	P	A	0.632	0.147	0.029	3.23E-07		0.478
DRB1	96	HY	P	A	0.801	0.325	0.031	1.61E-25		0.796
DRB1	96	QE	P	A	0.186	-0.323	0.031	4.48E-25		0.864
DRB1	96	Qx	P	A	0.175	-0.320	0.031	2.80E-24	7.73E-23	0.91
DRB1	96	Q	P	A	0.162	-0.310	0.031	3.03E-23		0.999
DRB1	96	H	P	A	0.612	0.096	0.023	3.12E-05		0.867
DRB1	133	R	P	A	0.818	0.326	0.032	2.50E-24		0.834
DRB1	133	L	P	A	0.156	-0.311	0.032	1.08E-22		0.999
DRB1	140	T	P	A	0.643	0.171	0.024	1.65E-12		0.815
DRB1	140	A	P	A	0.331	-0.163	0.024	1.14E-11		0.907
DRB1	142	V	P	A	0.818	0.326	0.032	2.50E-24		0.834
DRB1	142	M	P	A	0.156	-0.311	0.032	1.08E-22		0.999

Table S21: Associations for HLA amino acid (AA) sequence substitutions that were significantly associated ($P<4.6\times10^{-5}$) with seroreactivity to EBV EBNA. At each position that contained a significantly associated allele and >2 possible substitutions, we tested all common (frequency ≥0.01) haplotypes at that position using the omnibus test (P_{omni}) in the conditional haplotype module of PLINK 1.07. Variants and haplotypes with the lowest p-value are indicated in bold and shaded.

Gene	AA position	AA	A1	A2	FRQ	BETA	SE	P	P_{omni}	INFO
A	152	E	P	A	0.188	0.126	0.022	6.87E-09	6.51E-09	0.949
A	152	EW	P	A	0.197	0.115	0.022	1.18E-07		0.892
A	152	ER	P	A	0.200	0.112	0.022	2.09E-07		0.892
A	152	AV	P	A	0.791	-0.095	0.021	5.50E-06		0.946
A	161		D	E	0.146	0.126	0.024	1.11E-07		0.999
B	9	D	P	A	0.146	-0.137	0.024	1.27E-08		0.999
B	11		S	A	0.306	0.096	0.018	1.80E-07		0.931
B	12		V	M	0.321	0.089	0.018	5.20E-07		0.997
B	67	Y	P	A	0.170	0.137	0.023	1.61E-09	2.12E-10	0.939
B	67	YS	P	A	0.573	0.077	0.017	8.48E-06		0.932
B	67	YC	P	A	0.295	0.081	0.019	1.69E-05		0.933
B	67	FM	P	A	0.301	-0.076	0.019	4.07E-05		0.950
B	70	Q	P	A	0.169	0.137	0.023	1.61E-09		0.944
B	70	QK	P	A	0.226	0.101	0.020	7.13E-07		0.943
B	152	E	P	A	0.292	0.205	0.025	4.90E-16		0.484
B	152	V	P	A	0.689	-0.178	0.023	1.27E-14		0.559
B	156	RW	P	A	0.184	0.174	0.026	4.23E-11	5.79E-10	0.623
B	156	Rx	P	A	0.160	0.183	0.028	7.80E-11		0.604
B	156	DL	P	A	0.797	-0.152	0.024	2.80E-10		0.715
B	156	R	P	A	0.141	0.172	0.028	8.00E-10		0.710
B	156	D	P	A	0.268	-0.121	0.023	1.34E-07		0.587
B	156	Dx	P	A	0.287	-0.119	0.023	3.67E-07		0.529
B	156	DW	P	A	0.311	-0.104	0.023	5.06E-06		0.529
B	163	Ex	P	A	0.305	0.090	0.020	7.41E-06	1.39E-04	0.747
B	163	T	P	A	0.280	-0.083	0.020	2.79E-05		0.881
B	163	E	P	A	0.287	0.082	0.020	3.17E-05		0.824
B	178	T	P	A	0.771	-0.137	0.023	1.12E-09		0.712
B	178	K	P	A	0.210	0.128	0.022	9.08E-09		0.800
C	99	S	P	A	0.157	0.143	0.023	3.22E-10	1.24E-09	0.998
C	99	YC	P	A	0.749	-0.112	0.019	3.19E-09		0.998
C	99	YF	P	A	0.808	-0.122	0.021	7.00E-09		0.998
C	99	Y	P	A	0.714	-0.103	0.018	1.50E-08		0.998
DPB1	8		V	L	0.280	-0.115	0.018	4.51E-10		0.998

DPB1	9	Y	P	A	0.237	-0.139	0.020	1.36E-12		0.995
DPB1	9	F	P	A	0.720	0.115	0.018	4.47E-10		0.998
DPB1	11		L	G	0.216	-0.145	0.020	8.23E-13		0.994
DPB1	57		D	E	0.154	-0.162	0.024	8.94E-12		0.965
DPB1	65		L	I	0.172	-0.178	0.023	6.56E-15		0.935
DPB1	76	M	P	A	0.779	0.102	0.021	1.32E-06		0.888
DPB1	76	V	P	A	0.201	-0.097	0.022	1.10E-05		0.881
DPB1	84	D	P	A	0.307	-0.086	0.018	1.72E-06		0.997
DPB1	84	G	P	A	0.686	0.086	0.018	1.91E-06		0.997
DPB1	85		E	G	0.307	-0.086	0.018	1.75E-06		0.997
DPB1	86		P	A	0.693	0.086	0.018	1.74E-06		0.997
DPB1	87		V	M	0.307	-0.086	0.018	1.73E-06		0.997
DPB1	96	K	P	A	0.302	-0.088	0.018	1.62E-06		0.991
DPB1	96	R	P	A	0.696	0.086	0.018	2.48E-06		0.996
DPB1	170	I	P	A	0.302	-0.088	0.018	1.63E-06		0.991
DPB1	170	T	P	A	0.696	0.086	0.018	2.49E-06		0.996
DPB1	178	L	P	A	0.892	-0.157	0.027	4.33E-09		0.975
DPB1	178	M	P	A	0.106	0.154	0.027	7.33E-09		0.991
DQA1	11	C	P	A	0.396	0.203	0.017	6.33E-33		0.999
DQA1	11	Y	P	A	0.604	-0.203	0.017	6.33E-33		0.999
DQA1	-16	M	P	A	0.580	-0.193	0.017	1.46E-30		0.998
DQA1	-16	L	P	A	0.420	0.193	0.017	2.67E-30		0.999
DQA1	18		F	S	0.396	0.203	0.017	6.34E-33		0.999
DQA1	26		S	T	0.204	-0.123	0.021	7.46E-09		0.980
DQA1	34		E	Q	0.484	-0.158	0.017	1.52E-21		0.989
DQA1	41		K	R	0.171	0.267	0.024	1.38E-29		0.816
DQA1	45		A	V	0.396	0.203	0.017	6.47E-33		0.999
DQA1	47	R	P	A	0.395	0.204	0.017	7.00E-33		0.997
DQA1	47	RC	P	A	0.660	0.203	0.018	1.16E-30		0.996
DQA1	47	K	P	A	0.136	-0.231	0.025	8.82E-21	6.29E-41	0.979
DQA1	47	RQ	P	A	0.600	0.127	0.017	1.94E-13		0.986
DQA1	47	Q	P	A	0.204	-0.123	0.021	7.46E-09		0.980
DQA1	47	RK	P	A	0.531	0.091	0.017	6.50E-08		0.988
DQA1	48		W	L	0.396	0.203	0.017	6.22E-33		0.999
DQA1	50	E	P	A	0.396	0.203	0.017	6.72E-33		0.998
DQA1	50	L	P	A	0.340	-0.203	0.018	1.15E-30		0.996
DQA1	52	S	P	A	0.396	0.203	0.017	6.49E-33		0.999
DQA1	52	H	P	A	0.136	-0.231	0.025	8.82E-21	1.17E-39	0.979
DQA1	52	R	P	A	0.468	-0.092	0.017	6.05E-08		0.989
DQA1	53	K	P	A	0.396	0.203	0.017	6.22E-33		0.999

DQA1	53	R	P	A	0.340	-0.203	0.018	1.15E-30		0.996
DQA1	54		L	F	0.136	-0.231	0.025	8.82E-21		0.979
DQA1	55		G	R	0.396	0.203	0.017	6.71E-33		0.998
DQA1	56	G	P	A	0.396	0.203	0.017	6.71E-33		0.998
DQA1	56	x	P	A	0.400	-0.127	0.017	2.07E-13	5.14E-32	0.985
DQA1	56	R	P	A	0.204	-0.123	0.021	7.46E-09		0.980
DQA1	61		G	F	0.396	0.203	0.017	6.21E-33		0.999
DQA1	64		R	T	0.396	0.203	0.017	6.73E-33		0.998
DQA1	66		M	I	0.396	0.203	0.017	6.21E-33		0.999
DQA1	69	A	P	A	0.396	0.203	0.017	6.21E-33		0.999
DQA1	69	L	P	A	0.580	-0.193	0.017	2.15E-30		0.998
DQA1	76	M	P	A	0.396	0.203	0.017	6.50E-33		0.998
DQA1	76	L	P	A	0.400	-0.127	0.017	2.07E-13	5.14E-32	0.985
DQA1	76	V	P	A	0.204	-0.123	0.021	7.46E-09		0.980
DQA1	80		Y	S	0.396	0.203	0.017	6.21E-33		0.999
DQA1	129	H	P	A	0.776	-0.101	0.021	2.33E-06		0.831
DQA1	129	Q	P	A	0.224	0.101	0.021	2.35E-06		0.831
DQA1	130	S	P	A	0.829	-0.267	0.024	1.29E-29		0.817
DQA1	130	A	P	A	0.172	0.267	0.023	1.32E-29		0.818
DQA1	175	Qx	P	A	0.396	0.204	0.017	5.72E-33		0.999
DQA1	175	Q	P	A	0.396	0.204	0.017	5.85E-33	3.21E-39	0.999
DQA1	175	E	P	A	0.364	-0.205	0.017	6.68E-32		0.996
DQA1	175	QK	P	A	0.636	0.205	0.017	6.72E-32		0.996
DQA1	187	T	P	A	0.206	-0.127	0.021	2.27E-09		0.971
DQA1	187	A	P	A	0.795	0.125	0.021	3.72E-09		0.971
DQA1	207	M	P	A	0.085	0.181	0.036	4.94E-07		0.593
DQA1	207	V	P	A	0.915	-0.181	0.036	5.02E-07		0.593
DQA1	215	L	P	A	0.339	-0.203	0.018	2.02E-30		0.996
DQA1	215	F	P	A	0.661	0.203	0.018	2.05E-30		0.996
DQA1	218	Q	P	A	0.397	0.203	0.017	1.29E-32		0.999
DQA1	218	R	P	A	0.603	-0.203	0.017	1.35E-32		0.999
DQB1	-4	L	P	A	0.361	0.217	0.017	3.73E-35		0.976
DQB1	-4	V	P	A	0.607	-0.207	0.017	8.70E-34		0.997
DQB1	-5	Px	P	A	0.639	-0.217	0.017	3.73E-35		0.976
DQB1	-5	P	P	A	0.607	-0.207	0.017	8.48E-34	2.73E-38	0.996
DQB1	-5	L	P	A	0.211	0.243	0.020	1.89E-33		0.970
DQB1	-5	PS	P	A	0.757	-0.216	0.019	3.65E-30		0.997
DQB1	-6	Tx	P	A	0.637	-0.218	0.017	2.99E-35		0.967
DQB1	-6	S	P	A	0.361	0.217	0.017	3.73E-35	5.24E-34	0.976
DQB1	-6	T	P	A	0.605	-0.208	0.017	6.77E-34		0.988

DQB1	-6	TA	P	A	0.607	-0.207	0.017	8.79E-34		0.996
DQB1	-10	S	P	A	0.254	-0.256	0.019	2.20E-39		0.999
DQB1	-10	A	P	A	0.714	0.240	0.019	9.83E-37		0.974
DQB1	-18	A	P	A	0.434	-0.165	0.017	2.09E-22		0.990
DQB1	-18	V	P	A	0.534	0.163	0.017	4.97E-22		0.971
DQB1	-21	D	P	A	0.361	0.217	0.017	3.73E-35		0.976
DQB1	-21	G	P	A	0.607	-0.207	0.017	9.02E-34		0.996
DQB1	9	YL	P	A	0.836	-0.218	0.023	1.09E-21	4.05E-21	0.912
DQB1	9	F	P	A	0.164	0.218	0.023	1.10E-21		0.912
DQB1	9	Y	P	A	0.833	-0.210	0.022	1.09E-20		0.913
DQB1	9	Yx	P	A	0.833	-0.210	0.022	1.09E-20		0.913
DQB1	28		S	T	0.254	-0.256	0.019	2.17E-39		1.000
DQB1	30	S	P	A	0.254	-0.256	0.019	2.17E-39	1.37E-38	1.000
DQB1	30	Y	P	A	0.500	0.119	0.017	4.56E-12		0.939
DQB1	30	H	P	A	0.246	0.105	0.020	1.42E-07		0.918
DQB1	37	Y	P	A	0.743	0.256	0.019	8.87E-40		0.999
DQB1	37	I	P	A	0.254	-0.256	0.019	2.17E-39		1.000
DQB1	38		V	A	0.409	-0.163	0.017	8.38E-22		0.996
DQB1	46		E	V	0.254	-0.256	0.019	2.24E-39		0.999
DQB1	47		F	Y	0.254	-0.256	0.019	2.17E-39		0.999
DQB1	52		L	P	0.254	-0.256	0.019	2.16E-39		0.999
DQB1	53		Q	L	0.395	0.204	0.017	5.33E-33		0.997
DQB1	55	L	P	A	0.254	-0.256	0.019	2.16E-39		0.999
DQB1	55	R	P	A	0.417	0.198	0.017	1.33E-31		0.997
**DQB1**	**57**	**A**	**P**	**A**	**0.401**	**-0.237**	**0.017**	**1.37E-42**	6.13E-44	**0.982**
DQB1	57	AV	P	A	0.552	-0.231	0.017	1.06E-41		0.952
DQB1	57	AS	P	A	0.411	-0.223	0.017	1.43E-38		0.981
DQB1	57	D	P	A	0.438	0.222	0.017	2.16E-38		0.950
DQB1	66		D	E	0.281	-0.244	0.019	4.39E-38		0.998
DQB1	67		I	V	0.281	-0.244	0.019	4.45E-38		0.998
DQB1	70	G	P	A	0.358	0.218	0.018	4.86E-35		0.965
DQB1	70	R	P	A	0.618	-0.210	0.017	1.02E-33		0.966
DQB1	71	K	P	A	0.254	-0.256	0.019	2.16E-39	4.64E-38	0.999
DQB1	71	KD	P	A	0.278	-0.243	0.019	1.18E-37		0.998
DQB1	71	KA	P	A	0.404	-0.164	0.017	8.36E-22		0.996
DQB1	71	T	P	A	0.572	0.162	0.017	1.04E-21		0.995
DQB1	74	A	P	A	0.254	-0.256	0.019	2.16E-39		0.999
DQB1	74	E	P	A	0.572	0.162	0.017	1.01E-21		0.995
DQB1	75		V	L	0.428	-0.162	0.017	1.04E-21		0.995
DQB1	77		R	T	0.406	-0.162	0.017	2.23E-21		0.995

DQB1	84	Q	P	A	0.605	-0.204	0.017	5.58E-33		0.996
DQB1	84	E	P	A	0.393	0.203	0.017	1.46E-32		0.996
DQB1	85	L	P	A	0.605	-0.204	0.017	5.54E-33		0.997
DQB1	85	V	P	A	0.393	0.203	0.017	1.46E-32		0.996
DQB1	86	EG	P	A	0.637	-0.216	0.017	1.16E-34	1.71E-33	0.965
DQB1	86	A	P	A	0.361	0.215	0.018	3.26E-34		0.965
DQB1	86	E	P	A	0.605	-0.204	0.017	5.58E-33		0.996
DQB1	86	Ex	P	A	0.607	-0.203	0.017	1.45E-32		0.996
DQB1	87	L	P	A	0.605	-0.204	0.017	5.34E-33	4.61E-38	0.997
DQB1	87	LY	P	A	0.786	-0.240	0.020	9.27E-33		0.956
DQB1	87	Lx	P	A	0.606	-0.203	0.017	1.40E-32		0.997
DQB1	87	F	P	A	0.212	0.240	0.020	2.05E-32		0.955
DQB1	89	T	P	A	0.605	-0.204	0.017	5.58E-33		0.997
DQB1	89	G	P	A	0.393	0.203	0.017	1.46E-32		0.996
DQB1	90	T	P	A	0.605	-0.204	0.017	5.33E-33		0.997
DQB1	90	I	P	A	0.394	0.203	0.017	1.40E-32		0.997
DQB1	125	A	P	A	0.605	-0.204	0.017	5.33E-33	4.20E-35	0.998
DQB1	125	Ax	P	A	0.606	-0.203	0.017	1.39E-32		0.997
DQB1	125	G	P	A	0.244	0.211	0.019	6.99E-29		0.998
DQB1	125	AS	P	A	0.752	-0.211	0.019	7.35E-29		0.989
DQB1	135	G	P	A	0.100	-0.217	0.028	1.03E-14		0.993
DQB1	135	D	P	A	0.898	0.209	0.028	6.04E-14		0.991
DQB1	203	I	P	A	0.606	-0.206	0.017	2.01E-33		0.991
DQB1	203	V	P	A	0.391	0.206	0.017	2.61E-33		0.997
DQB1	220	R	P	A	0.394	0.203	0.017	1.24E-32		0.996
DQB1	220	H	P	A	0.603	-0.203	0.017	2.38E-32		0.985
DQB1	221	Q	P	A	0.394	0.203	0.017	1.20E-32		0.995
DQB1	221	H	P	A	0.604	-0.203	0.017	2.34E-32		0.986
DRB1	-1	A	P	A	0.814	-0.230	0.022	2.71E-26		0.921
DRB1	-1	S	P	A	0.149	0.239	0.023	1.06E-25		0.994
DRB1	-17	A	P	A	0.801	-0.235	0.021	2.62E-28		0.911
DRB1	-17	T	P	A	0.162	0.242	0.022	1.59E-27		0.977
DRB1	-24	F	P	A	0.188	-0.147	0.022	1.67E-11		0.997
DRB1	-24	L	P	A	0.775	0.124	0.021	4.11E-09		0.937
DRB1	4	R	P	A	0.846	0.187	0.023	1.44E-15		0.974
DRB1	4	Q	P	A	0.151	-0.186	0.023	1.50E-15		1.000
DRB1	11	VG	P	A	0.332	-0.221	0.018	6.95E-36	2.60E-49	0.999
DRB1	11	SPD	P	A	0.662	0.220	0.018	1.70E-35		0.986
DRB1	11	SVG	P	A	0.826	-0.250	0.021	5.55E-31		0.978
DRB1	11	PD	P	A	0.168	0.251	0.022	6.11E-31		1.000

DRB1	11	SPL	P	A	0.655	0.202	0.017	7.17E-31		0.999
DRB1	11	SP	P	A	0.650	0.201	0.017	1.50E-30		0.986
DRB1	11	PL	P	A	0.162	0.242	0.022	1.59E-27		0.977
DRB1	11	P	P	A	0.156	0.243	0.022	1.64E-27		1.000
DRB1	11	G	P	A	0.138	-0.229	0.024	4.29E-21		1.000
DRB1	11	GL	P	A	0.144	-0.223	0.024	1.96E-20		0.974
DRB1	11	GD	P	A	0.151	-0.186	0.023	1.50E-15		1.000
DRB1	11	SPV	P	A	0.844	0.182	0.023	4.37E-15		0.975
DRB1	11	V	P	A	0.194	-0.145	0.021	1.47E-11		0.999
DRB1	11	VL	P	A	0.200	-0.142	0.021	3.24E-11		0.980
DRB1	11	VD	P	A	0.206	-0.118	0.021	1.91E-08		0.999
DRB1	11	SPG	P	A	0.788	0.115	0.021	3.40E-08		0.981
DRB1	13	SHY	P	A	0.772	-0.269	0.020	2.58E-41		0.919
DRB1	13	RG	P	A	0.204	0.268	0.021	1.76E-38		0.932
DRB1	13	HY	P	A	0.327	-0.222	0.018	8.36E-36		0.999
DRB1	13	SRG	P	A	0.650	0.201	0.017	1.50E-30		0.986
DRB1	13	RF	P	A	0.180	0.243	0.021	6.99E-30		0.978
DRB1	13	R	P	A	0.156	0.243	0.022	1.64E-27		1.000
DRB1	13	SRF	P	A	0.625	0.175	0.018	4.92E-23		0.952
DRB1	13	Y	P	A	0.138	-0.229	0.024	4.29E-21		1.000
DRB1	13	SR	P	A	0.602	0.157	0.017	2.66E-19		0.940
DRB1	13	YF	P	A	0.162	-0.179	0.023	4.95E-15	2.95E-51	0.976
DRB1	13	H	P	A	0.189	-0.146	0.022	1.66E-11		0.999
DRB1	13	FG	P	A	0.071	0.226	0.035	1.20E-10		0.784
DRB1	13	SY	P	A	0.584	-0.107	0.017	6.07E-10		0.941
DRB1	13	G	P	A	0.048	0.260	0.043	1.86E-09		0.753
DRB1	13	HF	P	A	0.212	-0.115	0.021	3.40E-08		0.981
DRB1	13	YG	P	A	0.186	-0.122	0.022	3.84E-08		0.924
DRB1	13	SYF	P	A	0.607	-0.095	0.017	5.14E-08		0.953
DRB1	13	SH	P	A	0.634	-0.086	0.018	9.34E-07		0.938
DRB1	13	SRH	P	A	0.790	0.091	0.021	1.66E-05		0.912
DRB1	13	SHF	P	A	0.657	-0.074	0.018	3.04E-05		0.950
DRB1	14	NA	K	E	0.138	-0.229	0.024	4.29E-21		1.000
DRB1	16	Y	P	A	0.048	0.260	0.043	1.86E-09		0.753
DRB1	16	H	P	A	0.952	-0.260	0.043	1.89E-09		0.751
DRB1	25	NA	Q	R	0.138	-0.229	0.024	4.29E-21		1.000
DRB1	26	F	P	A	0.837	-0.219	0.032	6.59E-12		0.343
DRB1	26	L	P	A	0.026	0.389	0.058	2.49E-11		0.719
DRB1	28	E	P	A	0.175	-0.151	0.023	1.10E-10		0.876
DRB1	28	D	P	A	0.812	0.118	0.023	1.91E-07		0.882

DRB1	30	H	P	A	0.023	0.821	0.076	5.58E-27		0.470
DRB1	30	HG	P	A	0.035	0.540	0.054	1.02E-23		0.647
DRB1	30	CH	P	A	0.029	0.653	0.067	2.97E-22		0.462
DRB1	30	YLR	P	A	0.959	-0.483	0.050	1.12E-21		0.618
DRB1	30	L	P	A	0.138	-0.229	0.024	4.29E-21		1.000
DRB1	30	LR	P	A	0.144	-0.224	0.024	7.32E-21		0.999
DRB1	30	LC	P	A	0.144	-0.223	0.024	1.96E-20		0.974
DRB1	30	YHG	P	A	0.850	0.218	0.024	3.10E-20		0.974
DRB1	30	YLC	P	A	0.959	-0.436	0.049	5.44E-19		0.693
DRB1	30	HR	P	A	0.028	0.555	0.064	3.51E-18		0.568
DRB1	30	YL	P	A	0.954	-0.399	0.046	8.51E-18		0.661
DRB1	30	YLG	P	A	0.966	-0.473	0.058	5.19E-16	3.74E-34	0.548
DRB1	30	LG	P	A	0.151	-0.186	0.023	1.50E-15		1.000
DRB1	30	YCH	P	A	0.844	0.183	0.023	1.78E-15		1.000
DRB1	30	YHR	P	A	0.844	0.182	0.023	4.37E-15		0.975
DRB1	30	YH	P	A	0.838	0.179	0.023	4.95E-15		0.976
DRB1	30	YCG	P	A	0.833	0.138	0.023	4.02E-09		0.913
DRB1	30	LH	P	A	0.161	-0.140	0.024	4.32E-09		0.910
DRB1	30	YG	P	A	0.827	0.134	0.023	7.92E-09		0.893
DRB1	30	YGR	P	A	0.833	0.136	0.024	8.54E-09		0.890
DRB1	30	YC	P	A	0.821	0.105	0.023	3.37E-06		0.918
DRB1	30	YCR	P	A	0.826	0.106	0.023	3.94E-06		0.916
DRB1	30	Y	P	A	0.815	0.103	0.023	5.36E-06		0.898
DRB1	30	YR	P	A	0.821	0.103	0.023	6.34E-06		0.896
DRB1	33	NA	H	N	0.189	-0.146	0.022	1.70E-11		0.999
DRB1	37	SL	P	A	0.184	0.287	0.022	1.55E-39		0.911
DRB1	37	YF	P	A	0.455	-0.227	0.019	2.45E-31		0.703
DRB1	37	S	P	A	0.169	0.243	0.022	2.21E-27		0.934
DRB1	37	NS	P	A	0.530	0.182	0.019	1.01E-20		0.695
DRB1	37	L	P	A	0.015	0.685	0.077	4.69E-19	**1.11E-55**	0.722
DRB1	37	F	P	A	0.155	-0.189	0.024	7.10E-15		0.898
DRB1	37	NF	P	A	0.516	-0.123	0.020	2.77E-10		0.703
DRB1	37	Y	P	A	0.301	-0.133	0.022	1.58E-09		0.681
DRB1	37	NY	P	A	0.662	-0.093	0.018	3.69E-07		0.889
DRB1	37	FL	P	A	0.170	-0.112	0.023	1.73E-06		0.885
DRB1	38	L	P	A	0.015	0.685	0.077	4.69E-19		0.722
DRB1	38	V	P	A	0.979	-0.456	0.064	9.79E-13		0.792
DRB1	47		F	Y	0.545	0.188	0.019	1.94E-22		0.750
DRB1	57	DA	P	A	0.781	0.126	0.022	1.35E-08	9.94E-05	0.821
DRB1	57	V	P	A	0.166	-0.117	0.023	2.94E-07		0.969

DRB1	57	D	P	A	0.766	0.093	0.022	2.80E-05		0.774
DRB1	60	S	P	A	0.166	-0.117	0.023	2.94E-07		0.969
DRB1	70	R	P	A	0.033	0.352	0.057	6.75E-10		0.606
DRB1	70	D	P	A	0.523	-0.129	0.022	4.78E-09		0.488
DRB1	71	A	P	A	0.127	0.300	0.027	1.32E-27		0.785
DRB1	71	KA	P	A	0.367	0.210	0.027	3.16E-15		0.328
DRB1	71	KR	P	A	0.632	-0.141	0.023	6.10E-10	7.89E-27	0.478
DRB1	71	KE	P	A	0.481	-0.106	0.022	1.58E-06		0.480
DRB1	71	R	P	A	0.392	-0.117	0.026	6.20E-06		0.351
DRB1	73		G	A	0.259	-0.185	0.024	7.46E-15		0.542
DRB1	74	Q	P	A	0.138	-0.229	0.024	4.29E-21		1.000
DRB1	74	QL	P	A	0.164	-0.211	0.023	2.32E-20		0.988
DRB1	74	AE	P	A	0.715	0.180	0.023	3.00E-15		0.558
DRB1	74	RQ	P	A	0.259	-0.185	0.024	7.46E-15		0.542
DRB1	74	RA	P	A	0.794	0.158	0.022	1.62E-12	5.23E-27	0.835
DRB1	74	QE	P	A	0.180	-0.167	0.024	1.85E-12		0.825
DRB1	74	E	P	A	0.042	0.381	0.065	5.26E-09		0.350
DRB1	74	A	P	A	0.674	0.126	0.022	1.40E-08		0.554
DRB1	74	AL	P	A	0.700	0.125	0.023	5.31E-08		0.540
DRB1	74	RE	P	A	0.162	0.174	0.034	3.69E-07		0.321
DRB1	78		V	Y	0.151	-0.186	0.023	1.50E-15		1.000
DRB1	85		A	V	0.024	0.666	0.076	2.38E-18		0.456
DRB1	86		V	G	0.477	0.208	0.025	2.01E-16		0.364
DRB1	96	Qx	P	A	0.175	0.241	0.022	6.17E-27		0.910
DRB1	96	Q	P	A	0.162	0.235	0.022	1.68E-26		0.999
DRB1	96	QE	P	A	0.186	0.236	0.022	3.92E-26		0.864
DRB1	96	HY	P	A	0.801	-0.236	0.022	5.20E-26	2.45E-29	0.796
DRB1	96	HQ	P	A	0.774	0.149	0.022	5.26E-12		0.822
DRB1	96	Yx	P	A	0.201	-0.151	0.022	7.43E-12		0.923
DRB1	96	YE	P	A	0.213	-0.147	0.022	8.04E-12		0.880
DRB1	96	Y	P	A	0.188	-0.146	0.022	1.86E-11		0.997
DRB1	98	K	P	A	0.648	0.210	0.018	6.91E-32		0.946
DRB1	98	E	P	A	0.339	-0.203	0.018	1.10E-30		0.998
DRB1	104	S	P	A	0.638	0.216	0.018	1.03E-32		0.906
DRB1	104	A	P	A	0.339	-0.203	0.018	1.10E-30		0.998
DRB1	120	S	P	A	0.781	0.153	0.022	3.47E-12		0.858
DRB1	120	N	P	A	0.194	-0.145	0.021	1.64E-11		0.997
DRB1	133	R	P	A	0.818	-0.251	0.023	3.80E-28		0.834
DRB1	133	L	P	A	0.156	0.243	0.022	1.37E-27		0.999
DRB1	142	V	P	A	0.818	-0.251	0.023	3.80E-28		0.834

DRB1	142	M	P	A	0.156	0.243	0.022	1.37E-27	0.999
DRB1	180	V	P	A	0.752	0.152	0.022	2.83E-12	0.780
DRB1	180	L	P	A	0.196	-0.150	0.022	9.00E-12	0.951
DRB1	181	T	P	A	0.789	0.182	0.022	6.07E-16	0.766
DRB1	181	M	P	A	0.163	-0.186	0.023	1.12E-15	0.949

Table S22: Associations for HLA amino acid (AA) sequence substitutions that were significantly associated ($P<4.6\times10^{-5}$) with seroreactivity to EBV EA-D. At each position that contained a significantly associated allele and >2 possible substitutions, we tested all common (frequency ≥0.01) haplotypes at that position using the omnibus test (P_{omni}) in the conditional haplotype module of PLINK 1.07. Variants and haplotypes with the lowest p-value are indicated in bold and shaded.

Gene	AA position	AA	A1	A2	FRQ	BETA	SE	P	P_{omni}	INFO
A	70		Q	H	0.301	0.100	0.019	1.66E-07		0.905
A	152	EW	P	A	0.197	0.103	0.022	3.21E-06		0.892
A	152	ER	P	A	0.200	0.102	0.022	3.63E-06	3.67E-05	0.892
A	152	E	P	A	0.188	0.102	0.022	3.66E-06		0.949
A	152	AV	P	A	0.791	-0.096	0.021	6.52E-06		0.946
A	161		D	E	0.146	0.116	0.024	1.34E-06		0.999
**B**	**9**	**D**	**P**	**A**	**0.146**	**-0.146**	**0.024**	**1.77E-09**		**0.999**
B	67	F	P	A	0.246	-0.100	0.021	1.22E-06		0.881
B	67	FM	P	A	0.301	-0.088	0.019	2.61E-06		0.950
B	156	Dx	P	A	0.287	-0.108	0.024	4.91E-06		0.529
B	156	DW	P	A	0.311	-0.104	0.023	6.30E-06	4.46E-03	0.529
B	156	D	P	A	0.268	-0.103	0.023	8.21E-06		0.587
B	156	RL	P	A	0.670	0.094	0.022	1.72E-05		0.579
DPB1	11		L	G	0.216	0.099	0.020	8.78E-07		0.994
DQA1	26		S	T	0.204	0.111	0.021	7.51E-08		0.980
DQA1	34		E	Q	0.484	0.083	0.017	6.65E-07		0.989
DQA1	40		G	E	0.264	-0.083	0.019	1.59E-05		0.995
DQA1	47	Q	P	A	0.204	0.111	0.021	7.51E-08		0.980
DQA1	47	RC	P	A	0.660	-0.094	0.017	8.11E-08	2.87E-08	0.996
DQA1	47	C	P	A	0.264	-0.083	0.019	1.59E-05		0.996
DQA1	50	L	P	A	0.340	0.094	0.017	8.15E-08		0.996
DQA1	50	V	P	A	0.264	-0.083	0.019	1.59E-05		0.995
DQA1	51		L	F	0.264	-0.083	0.019	1.59E-05		0.996
DQA1	53	R	P	A	0.340	0.094	0.017	8.15E-08		0.996
DQA1	53	Q	P	A	0.264	-0.083	0.019	1.59E-05		0.995
DQA1	56	R	P	A	0.204	0.111	0.021	7.51E-08		0.980
DQA1	75		S	I	0.240	-0.090	0.020	6.28E-06		0.995
DQA1	76	V	P	A	0.204	0.111	0.021	7.51E-08		0.980
DQA1	107	I	P	A	0.240	-0.090	0.020	6.26E-06		0.995
DQA1	107	T	P	A	0.758	0.089	0.020	7.09E-06		0.986
DQA1	156	F	P	A	0.760	0.090	0.020	6.18E-06		0.994
DQA1	156	L	P	A	0.240	-0.090	0.020	6.19E-06		0.994
DQA1	161	E	P	A	0.240	-0.090	0.020	6.21E-06		0.994

DQA1	161	D	P	A	0.760	0.090	0.020	6.22E-06		0.995
DQA1	163	S	P	A	0.240	-0.090	0.020	6.22E-06		0.995
DQA1	163	I	P	A	0.760	0.090	0.020	6.23E-06		0.994
DQA1	175	QK	P	A	0.636	-0.093	0.017	8.16E-08	3.63E-08	0.996
DQA1	175	E	P	A	0.364	0.093	0.017	8.17E-08		0.996
DQA1	175	QE	P	A	0.760	0.090	0.020	6.01E-06		0.995
DQA1	175	K	P	A	0.240	-0.090	0.020	6.20E-06		0.995
DQA1	187	T	P	A	0.206	0.114	0.021	3.26E-08		0.971
DQA1	187	A	P	A	0.795	-0.112	0.021	5.58E-08		0.971
DQA1	215	L	P	A	0.339	0.093	0.018	1.24E-07		0.996
DQA1	215	F	P	A	0.661	-0.093	0.018	1.24E-07		0.996
DQB1	-10	A	P	A	0.714	0.099	0.019	1.74E-07		0.974
DQB1	-10	S	P	A	0.254	-0.098	0.019	5.23E-07		0.999
DQB1	13	A	P	A	0.180	0.096	0.022	1.37E-05		0.984
DQB1	13	G	P	A	0.820	-0.096	0.022	1.37E-05		0.985
DQB1	26	Y	P	A	0.180	0.096	0.022	1.38E-05		0.985
DQB1	28		S	T	0.254	-0.098	0.019	5.25E-07		1.000
DQB1	30	S	P	A	0.254	-0.098	0.019	5.25E-07		1.000
DQB1	30	Y	P	A	0.500	0.078	0.017	6.24E-06		0.939
DQB1	37	Y	P	A	0.743	0.100	0.019	2.92E-07		0.999
DQB1	37	I	P	A	0.254	-0.098	0.019	5.25E-07		1.000
DQB1	38		V	A	0.409	-0.080	0.017	2.63E-06		0.996
DQB1	45		E	G	0.177	0.100	0.022	6.01E-06		0.985
DQB1	46		E	V	0.254	-0.098	0.019	5.25E-07		0.999
DQB1	47		F	Y	0.254	-0.098	0.019	5.25E-07		0.999
DQB1	52		L	P	0.254	-0.098	0.019	5.26E-07		0.999
DQB1	55	P	P	A	0.329	0.105	0.018	3.61E-09		0.997
DQB1	55	L	P	A	0.254	-0.098	0.019	5.26E-07		0.999
DQB1	66		D	E	0.281	-0.093	0.019	7.71E-07		0.998
DQB1	67		I	V	0.281	-0.093	0.019	7.69E-07		0.998
DQB1	71	K	P	A	0.254	-0.098	0.019	5.26E-07	4.61E-06	0.999
DQB1	71	KD	P	A	0.278	-0.092	0.019	1.36E-06		0.998
DQB1	71	KA	P	A	0.404	-0.077	0.017	6.43E-06		0.996
DQB1	71	T	P	A	0.572	0.075	0.017	9.15E-06		0.995
DQB1	74	A	P	A	0.254	-0.098	0.019	5.26E-07		0.999
DQB1	74	E	P	A	0.572	0.075	0.017	9.17E-06		0.995
DQB1	75		V	L	0.428	-0.075	0.017	9.20E-06		0.995
DQB1	77		R	T	0.406	-0.078	0.017	4.46E-06		0.995
DQB1	140	T	P	A	0.350	0.104	0.018	2.85E-09		0.997
DQB1	140	A	P	A	0.648	-0.103	0.018	4.56E-09		0.996

DQB1	167	H	P	A	0.181	0.091	0.022	3.43E-05		0.988
DQB1	182	N	P	A	0.350	0.104	0.018	2.85E-09		0.997
DQB1	182	S	P	A	0.648	-0.103	0.018	4.57E-09		0.997
DRB1	9	K	P	A	0.012	0.404	0.073	3.25E-08		1.000
DRB1	11	D	P	A	0.012	0.404	0.073	3.25E-08	**1.71E-10**	1.000
DRB1	11	SP	P	A	0.650	-0.095	0.017	4.84E-08		0.986
DRB1	11	SPL	P	A	0.655	-0.095	0.017	5.54E-08		0.999
DRB1	11	VD	P	A	0.206	0.108	0.020	1.12E-07		0.999
DRB1	11	SPG	P	A	0.788	-0.108	0.020	1.14E-07		0.981
DRB1	11	LD	P	A	0.018	0.321	0.065	7.10E-07		0.825
DRB1	11	SPD	P	A	0.662	-0.073	0.018	3.05E-05		0.986
DRB1	11	VG	P	A	0.332	0.073	0.018	3.34E-05		0.999
DRB1	13	SRG	P	A	0.650	-0.095	0.017	4.84E-08	2.44E-07	0.986
DRB1	13	HF	P	A	0.212	0.108	0.020	1.14E-07		0.981
DRB1	13	SR	P	A	0.602	-0.090	0.017	2.56E-07		0.940
DRB1	13	F	P	A	0.023	0.271	0.057	1.69E-06		0.863
DRB1	13	SRY	P	A	0.740	-0.093	0.019	1.93E-06		0.926
DRB1	28	H	P	A	0.012	0.404	0.073	3.25E-08		1.000
DRB1	30	G	P	A	0.012	0.404	0.073	3.25E-08	6.80E-07	1.000
DRB1	30	GR	P	A	0.018	0.319	0.062	2.40E-07		0.999
DRB1	30	CG	P	A	0.018	0.321	0.065	7.10E-07		0.825
DRB1	30	YLH	P	A	0.977	-0.271	0.057	1.69E-06		0.863
DRB1	31	I	P	A	0.018	0.321	0.065	7.10E-07		0.825
DRB1	31	F	P	A	0.977	-0.271	0.057	1.69E-06		0.863
DRB1	37	YF	P	A	0.455	0.081	0.019	2.95E-05		0.703
DRB1	47		F	Y	0.545	-0.105	0.019	3.78E-08		0.750
DRB1	70	R	P	A	0.033	0.245	0.057	1.88E-05		0.606
DRB1	71	KR	P	A	0.632	0.096	0.023	2.92E-05		0.478
DRB1	74	E	P	A	0.042	0.342	0.065	1.78E-07		0.350
DRB1	86		V	G	0.477	-0.138	0.025	4.72E-08		0.364
DRB1	98	K	P	A	0.648	-0.097	0.018	5.41E-08		0.946
DRB1	98	E	P	A	0.339	0.092	0.017	1.40E-07		0.998
DRB1	104	S	P	A	0.638	-0.100	0.018	2.70E-08		0.906
DRB1	104	A	P	A	0.339	0.092	0.017	1.40E-07		0.998
DRB1	149	H	P	A	0.449	-0.076	0.018	2.17E-05		0.837
DRB1	180	V	P	A	0.752	-0.087	0.021	3.91E-05		0.780
DRB1	233	R	P	A	0.335	-0.092	0.018	4.85E-07		0.954
DRB1	233	T	P	A	0.626	0.083	0.018	2.06E-06		0.985

Table S23: Associations for HLA amino acid (AA) sequence substitutions that were significantly associated ($P<4.6\times10^{-5}$) with seroreactivity to VZV. At each position that contained a significant allele and >2 possible substitutions, we tested all possible common (frequency ≥0.01) haplotypes constructed from all well-imputed (INFO>0.30) amino acid residues at that position using the haplotype omnibus test (P_{omni}) in PLINK 1.07. Variants and haplotypes with the lowest p-value are indicated in bold and shaded.

Gene	AA position	AA	A1	A2	FRQ	BETA	SE	P	P_{omni}	INFO
A	-15	L	P	A	0.536	0.100	0.016	1.46E-09		0.998
A	-15	V	P	A	0.462	-0.099	0.017	1.93E-09		0.993
A	44		K	R	0.197	0.182	0.021	1.05E-18		0.999
A	62	Q	P	A	0.479	0.119	0.017	1.17E-12	2.04E-10	0.957
A	62	QE	P	A	0.575	0.112	0.017	4.12E-11		0.956
A	62	QR	P	A	0.576	0.105	0.017	2.38E-10		0.999
A	62	QL	P	A	0.519	0.105	0.017	3.52E-10		0.957
A	62	GL	P	A	0.328	-0.106	0.018	1.76E-09		0.999
A	62	GR	P	A	0.386	-0.101	0.017	5.44E-09		0.954
A	62	GE	P	A	0.384	-0.094	0.017	2.50E-08		0.999
A	62	G	P	A	0.289	-0.099	0.018	7.48E-08		0.999
A	66		K	N	0.388	-0.097	0.017	1.31E-08		0.982
A	67		M	V	0.197	0.182	0.021	1.18E-18		0.999
A	74	D	P	A	0.714	0.100	0.019	6.10E-08		0.983
A	74	H	P	A	0.286	-0.100	0.019	6.98E-08		0.983
A	76	A	P	A	0.258	0.135	0.019	2.72E-12		0.892
A	76	V	P	A	0.589	-0.101	0.017	4.56E-09		0.918
A	77	N	P	A	0.366	0.105	0.018	2.62E-09		0.872
A	77	D	P	A	0.589	-0.101	0.017	4.56E-09		0.918
A	90		D	A	0.301	0.127	0.018	1.57E-12		0.997
A	95	I	P	A	0.616	0.095	0.017	2.14E-08		0.996
A	95	V	P	A	0.272	-0.106	0.019	5.85E-08		0.916
A	97	IK	P	A	0.425	0.112	0.017	2.09E-11	1.55E-11	0.995
A	97	I	P	A	0.425	0.112	0.017	2.09E-11		0.995
A	97	IM	P	A	0.660	0.106	0.018	3.04E-09		0.958
A	97	R	P	A	0.340	-0.106	0.018	3.13E-09		0.958
A	105		P	S	0.343	0.127	0.017	3.42E-13		0.963
A	107		W	G	0.289	-0.099	0.018	5.92E-08		0.996
A	114	RE	P	A	0.493	0.100	0.017	2.55E-09	5.38E-08	0.965
A	114	H	P	A	0.392	-0.097	0.017	1.48E-08		0.964
A	114	R	P	A	0.470	0.095	0.017	1.62E-08		0.965
A	114	RQ	P	A	0.586	0.089	0.017	1.25E-07		0.964
A	116	Y	P	A	0.392	-0.097	0.017	1.49E-08		0.964
A	116	D	P	A	0.585	0.089	0.017	1.26E-07		0.964

A	127		K	N	0.420	-0.092	0.017	3.88E-08		0.999
A	142		T	I	0.325	-0.092	0.018	2.07E-07		0.999
A	145		H	R	0.324	-0.092	0.018	2.05E-07		0.999
A	150		V	A	0.197	0.182	0.021	1.19E-18		0.998
A	152	AR	P	A	0.269	0.156	0.019	1.11E-16	5.33E-15	0.948
A	152	AW	P	A	0.267	0.155	0.019	1.35E-16		0.950
A	152	EV	P	A	0.721	-0.151	0.018	1.56E-16		0.994
A	152	A	P	A	0.257	0.152	0.019	5.86E-16		0.997
A	152	V	P	A	0.533	-0.102	0.017	1.21E-09		0.966
A	152	VW	P	A	0.543	-0.102	0.017	1.89E-09		0.930
A	152	VR	P	A	0.545	-0.101	0.017	2.39E-09		0.933
A	152	AE	P	A	0.445	0.097	0.017	7.71E-09		0.969
A	156	R	P	A	0.197	0.182	0.021	1.19E-18	2.70E-17	0.998
A	156	RQ	P	A	0.345	0.128	0.018	9.15E-13		0.923
A	156	RW	P	A	0.296	0.133	0.019	1.80E-12		0.901
A	156	L	P	A	0.556	-0.109	0.018	8.53E-10		0.851
A	158		V	A	0.197	0.182	0.021	1.19E-18		0.998
A	163	T	P	A	0.701	-0.127	0.018	2.01E-12		0.988
A	163	R	P	A	0.299	0.127	0.018	2.14E-12		0.989
A	166		D	E	0.289	0.129	0.018	1.53E-12		0.982
A	167		G	W	0.289	0.129	0.018	1.52E-12		0.982
A	184	P	P	A	0.558	0.100	0.017	2.13E-09		0.998
A	184	A	P	A	0.442	-0.100	0.017	2.20E-09		0.998
A	193	P	P	A	0.521	0.100	0.017	1.38E-09		0.999
A	193	A	P	A	0.479	-0.100	0.017	1.41E-09		0.999
A	194	I	P	A	0.521	0.100	0.017	1.38E-09		0.999
A	194	V	P	A	0.479	-0.100	0.017	1.41E-09		0.999
A	207	G	P	A	0.521	0.100	0.017	1.45E-09		0.999
A	207	S	P	A	0.479	-0.100	0.017	1.46E-09		0.999
A	253	EK	P	A	0.521	0.101	0.017	1.26E-09	1.34E-09	0.999
A	253	Q	P	A	0.479	-0.101	0.017	1.28E-09		0.999
A	253	Ex	P	A	0.521	0.100	0.017	1.34E-09		0.999
A	253	E	P	A	0.521	0.100	0.017	1.34E-09		0.999
A	276	P	P	A	0.562	-0.115	0.017	9.31E-12		0.947
A	276	L	P	A	0.425	0.112	0.017	1.74E-11		0.999
A	294	F	P	A	0.466	-0.105	0.017	5.82E-10		0.948
A	294	L	P	A	0.521	0.101	0.017	1.19E-09		0.999
A	321	S	P	A	0.562	-0.116	0.017	8.74E-12		0.947
A	321	T	P	A	0.425	0.113	0.017	1.55E-11		0.999
B	-8	L	P	A	0.539	0.075	0.017	7.07E-06		0.984

B	-8	V	P	A	0.459	-0.075	0.017	7.12E-06		0.977
B	-10	G	P	A	0.554	-0.088	0.017	1.86E-07		0.975
B	-10	A	P	A	0.444	0.087	0.017	1.87E-07		0.984
B	-11	S	P	A	0.516	0.076	0.017	4.31E-06		0.984
B	-11	W	P	A	0.482	-0.076	0.017	4.32E-06		0.976
B	-21	M	P	A	0.362	0.097	0.017	1.55E-08		0.992
B	-21	T	P	A	0.635	-0.097	0.017	1.56E-08		0.983
B	-23	L	P	A	0.361	0.097	0.017	1.57E-08		0.998
B	-23	R	P	A	0.637	-0.097	0.017	1.58E-08		0.989
**B**	**9**	**D**	**P**	**A**	**0.146**	**0.237**	**0.023**	**9.71E-25**		**0.999**
B	9	Y	P	A	0.663	-0.124	0.017	1.24E-12		0.986
B	24	S	P	A	0.416	0.100	0.017	2.19E-09		0.982
B	24	T	P	A	0.333	-0.077	0.017	1.06E-05		0.988
B	32		L	Q	0.313	-0.077	0.018	1.35E-05		0.988
B	45	E	P	A	0.443	0.080	0.017	1.70E-06	1.67E-05	0.966
B	45	EG	P	A	0.445	0.080	0.017	1.92E-06		0.972
B	45	ET	P	A	0.615	0.071	0.017	2.97E-05		0.957
B	45	KM	P	A	0.383	-0.071	0.017	3.26E-05		0.963
B	45	EM	P	A	0.569	0.069	0.017	3.85E-05		0.983
B	45	TK	P	A	0.429	-0.068	0.017	4.24E-05		0.989
B	63		E	N	0.480	-0.084	0.017	1.56E-06		0.869
B	67	F	P	A	0.246	0.154	0.020	9.88E-15	4.48E-10	0.881
B	67	FM	P	A	0.301	0.125	0.018	5.18E-12		0.950
B	67	FC	P	A	0.371	0.112	0.018	4.75E-10		0.875
B	67	YS	P	A	0.573	-0.099	0.017	6.25E-09		0.932
B	67	YF	P	A	0.416	0.083	0.018	2.33E-06		0.873
B	67	SC	P	A	0.528	-0.076	0.017	9.01E-06		0.924
B	74		D	Y	0.409	0.089	0.018	5.28E-07		0.852
B	77	SG	P	A	0.613	0.089	0.020	1.17E-05	2.44E-04	0.613
B	77	S	P	A	0.612	0.088	0.020	1.29E-05		0.613
B	77	N	P	A	0.321	-0.094	0.022	1.36E-05		0.574
B	77	SD	P	A	0.678	0.094	0.022	1.51E-05		0.574
B	80	N	P	A	0.611	0.088	0.020	1.19E-05		0.619
B	81		A	L	0.321	-0.094	0.022	1.36E-05		0.574
B	82		L	R	0.389	-0.088	0.020	1.21E-05		0.619
B	83		R	G	0.389	-0.088	0.020	1.21E-05		0.619
B	95	L	P	A	0.571	0.081	0.018	1.05E-05		0.751
B	97	SW	P	A	0.318	0.111	0.018	1.95E-09	9.87E-07	0.908
B	97	S	P	A	0.313	0.110	0.018	2.37E-09		0.926
B	97	SV	P	A	0.350	0.106	0.018	3.60E-09		0.924

B	97	SWV	P	A	0.355	0.106	0.018	3.61E-09		0.919
B	97	STV	P	A	0.461	0.094	0.018	1.74E-07		0.782
B	97	RN	P	A	0.534	-0.094	0.018	1.83E-07		0.780
B	97	STW	P	A	0.429	0.094	0.018	2.28E-07		0.771
B	97	ST	P	A	0.424	0.093	0.018	2.64E-07		0.786
B	97	SWN	P	A	0.374	0.089	0.018	4.74E-07		0.907
B	97	RT	P	A	0.589	-0.088	0.017	4.86E-07		0.916
B	97	SNV	P	A	0.407	0.087	0.017	5.13E-07		0.921
B	97	SN	P	A	0.369	0.088	0.018	5.89E-07		0.927
B	97	RW	P	A	0.483	-0.081	0.018	7.41E-06		0.779
B	97	R	P	A	0.478	-0.080	0.018	7.52E-06		0.776
B	97	RV	P	A	0.515	-0.079	0.018	1.17E-05		0.771
B	97	STN	P	A	0.480	0.078	0.018	1.34E-05		0.787
B	113	H	P	A	0.712	0.083	0.020	2.75E-05		0.798
B	114	Nx	P	A	0.472	0.174	0.023	4.00E-14	3.84E-10	0.454
B	114	DH	P	A	0.528	-0.174	0.023	4.07E-14		0.454
B	114	N	P	A	0.453	0.162	0.022	1.50E-13		0.504
B	114	NK	P	A	0.453	0.162	0.022	1.50E-13		0.504
B	114	D	P	A	0.467	-0.151	0.023	4.53E-11		0.458
B	114	DK	P	A	0.467	-0.151	0.023	4.53E-11		0.458
B	114	Dx	P	A	0.486	-0.141	0.022	1.27E-10		0.509
B	114	NH	P	A	0.514	0.141	0.022	1.28E-10		0.509
B	116	YFx	P	A	0.550	0.109	0.020	4.50E-08	8.13E-05	0.617
B	116	YF	P	A	0.532	0.103	0.019	6.86E-08		0.677
B	116	DS	P	A	0.376	-0.110	0.022	8.01E-07		0.512
B	116	Y	P	A	0.431	0.088	0.018	1.45E-06		0.808
B	116	YFL	P	A	0.606	0.101	0.021	1.49E-06		0.572
B	116	Yx	P	A	0.450	0.090	0.019	1.54E-06		0.743
B	116	YFS	P	A	0.707	0.094	0.020	2.15E-06		0.769
B	116	DL	P	A	0.275	-0.089	0.020	5.74E-06		0.843
B	116	Dx	P	A	0.219	-0.109	0.024	6.85E-06		0.596
B	116	YS	P	A	0.606	0.089	0.020	8.71E-06		0.648
B	116	D	P	A	0.201	-0.103	0.024	1.82E-05		0.675
B	116	YSx	P	A	0.625	0.082	0.020	2.73E-05		0.707
B	131	R	P	A	0.364	0.099	0.018	4.31E-08		0.876
B	131	S	P	A	0.617	-0.101	0.019	4.78E-08		0.804
B	152	E	P	A	0.292	-0.115	0.025	4.19E-06		0.484
B	152	V	P	A	0.689	0.100	0.023	1.11E-05		0.559
B	156	D	P	A	0.268	0.177	0.022	1.94E-15	3.98E-05	0.587
B	156	Dx	P	A	0.287	0.180	0.023	2.45E-15		0.529

B	156	DW	P	A	0.311	0.168	0.022	3.54E-14		0.529
B	156	RL	P	A	0.670	-0.148	0.021	2.19E-12		0.579
B	156	LW	P	A	0.572	-0.118	0.022	4.31E-08		0.501
B	156	RD	P	A	0.410	0.114	0.021	4.67E-08		0.554
B	156	Lx	P	A	0.547	-0.115	0.022	1.05E-07		0.485
B	156	L	P	A	0.529	-0.104	0.021	6.35E-07		0.526
B	163	T	P	A	0.280	0.172	0.019	5.20E-19		0.881
B	163	EL	P	A	0.701	-0.174	0.019	6.40E-19	1.62E-16	0.804
B	163	L	P	A	0.415	-0.090	0.018	6.28E-07		0.801
B	163	ET	P	A	0.567	0.086	0.017	8.10E-07		0.871
B	167	W	P	A	0.830	0.114	0.027	2.82E-05		0.588
B	177	D	P	A	0.363	0.099	0.018	4.70E-08		0.877
B	177	E	P	A	0.619	-0.101	0.018	5.33E-08		0.804
B	180	E	P	A	0.363	0.099	0.018	4.70E-08		0.877
B	180	Q	P	A	0.619	-0.101	0.018	5.33E-08		0.804
B	199	A	P	A	0.837	0.121	0.029	2.48E-05		0.536
C	-9	G	P	A	0.357	0.085	0.017	6.90E-07		1.000
C	-9	A	P	A	0.643	-0.085	0.017	7.07E-07		0.998
C	-15	L	P	A	0.419	0.076	0.017	6.86E-06		1.000
C	-15	I	P	A	0.580	-0.076	0.017	6.99E-06		0.997
C	-17	A	P	A	0.363	0.090	0.017	1.34E-07		1.000
C	-17	T	P	A	0.637	-0.090	0.017	1.37E-07		0.998
C	9	D	P	A	0.446	0.081	0.017	1.01E-06		0.999
C	9	DF	P	A	0.480	0.079	0.016	1.50E-06		0.999
C	24		S	A	0.480	0.079	0.016	1.51E-06		0.999
C	66		N	K	0.201	0.176	0.020	6.87E-18		0.998
C	116	S	P	A	0.571	0.071	0.017	3.50E-05		0.939
C	147		L	W	0.363	0.090	0.017	1.44E-07		0.999
C	152	A	P	A	0.403	0.075	0.017	7.25E-06		0.999
C	152	E	P	A	0.586	-0.077	0.017	7.72E-06		0.954
C	184	PR	P	A	0.363	0.090	0.017	1.48E-07		0.999
C	184	H	P	A	0.637	-0.090	0.017	1.49E-07	1.37E-07	0.999
C	184	P	P	A	0.357	0.085	0.017	7.56E-07		0.999
C	184	Px	P	A	0.357	0.085	0.017	7.64E-07		0.999
C	194	V	P	A	0.643	-0.085	0.017	7.64E-07		0.999
C	194	L	P	A	0.357	0.085	0.017	7.66E-07		1.000
C	253	Q	P	A	0.363	0.090	0.017	1.61E-07		0.999
C	253	E	P	A	0.637	-0.090	0.017	1.63E-07		0.997
C	261	M	P	A	0.357	0.085	0.017	8.22E-07		0.999
C	261	V	P	A	0.643	-0.085	0.017	8.31E-07		0.997

C	267	Q	P	A	0.363	0.090	0.017	1.61E-07		0.999
C	267	P	P	A	0.637	-0.090	0.017	1.85E-07		0.996
C	273	S	P	A	0.357	0.085	0.017	8.21E-07		0.999
C	273	R	P	A	0.643	-0.084	0.017	9.36E-07		0.996
C	285	ML	P	A	0.362	0.090	0.017	1.61E-07	1.52E-07	0.998
C	285	V	P	A	0.637	-0.089	0.017	1.87E-07		0.996
C	285	M	P	A	0.357	0.085	0.017	8.17E-07		0.999
C	285	Mx	P	A	0.357	0.084	0.017	9.45E-07		0.996
C	295	V	P	A	0.357	0.085	0.017	8.47E-07		0.994
C	295	A	P	A	0.642	-0.084	0.017	9.80E-07		0.992
C	305	T	P	A	0.356	0.084	0.017	9.90E-07		0.998
C	305	A	P	A	0.643	-0.084	0.017	1.16E-06		0.996
C	306	A	P	A	0.362	0.089	0.017	1.96E-07		0.998
C	306	V	P	A	0.637	-0.089	0.017	2.31E-07		0.996
C	307	M	P	A	0.357	0.084	0.017	9.85E-07		0.998
C	307	V	P	A	0.643	-0.084	0.017	1.16E-06		0.996
C	326	C	P	A	0.357	0.084	0.017	9.86E-07		0.999
C	326	S	P	A	0.642	-0.084	0.017	1.07E-06		0.995
C	339	T	P	A	0.357	0.084	0.017	1.00E-06		0.999
C	339	A	P	A	0.642	-0.084	0.017	1.07E-06		0.995
DQA1	-16	L	P	A	0.420	-0.080	0.017	2.01E-06		0.999
DQA1	-16	M	P	A	0.580	0.079	0.017	2.21E-06		0.998
DQA1	11	Y	P	A	0.604	0.096	0.017	1.65E-08		0.999
DQA1	11	C	P	A	0.396	-0.096	0.017	1.65E-08		0.999
DQA1	18		F	S	0.396	-0.096	0.017	1.65E-08		0.999
DQA1	40		G	E	0.264	0.148	0.019	1.79E-15		0.995
DQA1	41		K	R	0.171	-0.126	0.024	1.28E-07		0.816
DQA1	45		A	V	0.396	-0.096	0.017	1.65E-08		0.999
DQA1	47	C	P	A	0.264	0.148	0.019	1.79E-15	7.09E-15	0.996
DQA1	47	RQ	P	A	0.600	-0.126	0.017	1.06E-13		0.986
DQA1	47	R	P	A	0.395	-0.096	0.017	1.68E-08		0.997
DQA1	47	RK	P	A	0.531	-0.089	0.017	8.77E-08		0.988
DQA1	48		W	L	0.396	-0.096	0.017	1.63E-08		0.999
DQA1	50	V	P	A	0.264	0.148	0.019	1.78E-15		0.995
DQA1	50	E	P	A	0.396	-0.096	0.017	1.66E-08		0.998
DQA1	51		L	F	0.264	0.148	0.019	1.79E-15		0.996
DQA1	52	S	P	A	0.396	-0.096	0.017	1.65E-08		0.999
DQA1	52	R	P	A	0.468	0.089	0.017	8.57E-08		0.989
DQA1	53	Q	P	A	0.264	0.148	0.019	1.78E-15		0.995
DQA1	53	K	P	A	0.396	-0.096	0.017	1.63E-08		0.999

DQA1	55		G	R	0.396	-0.096	0.017	1.66E-08		0.998
DQA1	56	x	P	A	0.400	0.126	0.017	1.08E-13		0.985
DQA1	56	G	P	A	0.396	-0.096	0.017	1.66E-08		0.998
DQA1	61		G	F	0.396	-0.096	0.017	1.63E-08		0.999
DQA1	64		R	T	0.396	-0.096	0.017	1.66E-08		0.998
DQA1	66		M	I	0.396	-0.096	0.017	1.63E-08		0.999
DQA1	69	A	P	A	0.396	-0.096	0.017	1.63E-08		0.999
DQA1	69	L	P	A	0.580	0.080	0.017	1.86E-06		0.998
DQA1	75		S	I	0.240	0.140	0.019	3.07E-13		0.995
DQA1	76	L	P	A	0.400	0.126	0.017	1.08E-13		0.985
DQA1	76	M	P	A	0.396	-0.096	0.017	1.65E-08		0.998
DQA1	80		Y	S	0.396	-0.096	0.017	1.63E-08		0.999
DQA1	107	T	P	A	0.758	-0.141	0.019	2.35E-13		0.986
DQA1	107	I	P	A	0.240	0.140	0.019	3.05E-13		0.995
DQA1	130	A	P	A	0.172	-0.126	0.024	1.25E-07		0.818
DQA1	130	S	P	A	0.829	0.126	0.024	1.26E-07		0.817
DQA1	156	L	P	A	0.240	0.140	0.019	3.00E-13		0.994
DQA1	156	F	P	A	0.760	-0.140	0.019	3.04E-13		0.994
DQA1	161	D	P	A	0.760	-0.140	0.019	2.99E-13		0.995
DQA1	161	E	P	A	0.240	0.140	0.019	3.01E-13		0.994
DQA1	163	I	P	A	0.760	-0.140	0.019	3.02E-13		0.994
DQA1	163	S	P	A	0.240	0.140	0.019	3.02E-13		0.995
DQA1	175	QE	P	A	0.760	-0.140	0.019	2.91E-13	1.19E-13	0.995
DQA1	175	K	P	A	0.240	0.140	0.019	3.01E-13		0.995
DQA1	175	Qx	P	A	0.396	-0.096	0.017	1.56E-08		0.999
DQA1	175	Q	P	A	0.396	-0.096	0.017	1.58E-08		0.999
DQA1	218	R	P	A	0.603	0.097	0.017	1.28E-08		0.999
DQA1	218	Q	P	A	0.397	-0.096	0.017	1.32E-08		0.999
DQB1	-4	V	P	A	0.607	0.095	0.017	2.05E-08		0.997
DQB1	-4	L	P	A	0.361	-0.098	0.017	2.07E-08		0.976
DQB1	-5	P	P	A	0.607	0.095	0.017	2.05E-08	3.51E-08	0.996
DQB1	-5	Px	P	A	0.639	0.098	0.017	2.08E-08		0.976
DQB1	-5	L	P	A	0.211	-0.105	0.020	2.17E-07		0.970
DQB1	-5	PS	P	A	0.757	0.096	0.019	4.85E-07		0.997
DQB1	-6	TA	P	A	0.607	0.095	0.017	2.06E-08	4.15E-08	0.996
DQB1	-6	S	P	A	0.361	-0.098	0.017	2.07E-08		0.976
DQB1	-6	T	P	A	0.605	0.095	0.017	2.88E-08		0.988
DQB1	-6	Tx	P	A	0.637	0.097	0.018	2.97E-08		0.967
DQB1	-10	S	P	A	0.254	0.161	0.019	1.30E-17		0.999
DQB1	-10	A	P	A	0.714	-0.149	0.018	4.41E-16		0.974

DQB1	-18	A	P	A	0.434	0.072	0.017	1.57E-05		0.990
DQB1	-18	V	P	A	0.534	-0.069	0.017	3.17E-05		0.971
DQB1	-21	G	P	A	0.607	0.095	0.017	2.05E-08		0.996
DQB1	-21	D	P	A	0.361	-0.098	0.017	2.07E-08		0.976
DQB1	13	G	P	A	0.820	0.091	0.022	2.71E-05		0.985
DQB1	13	A	P	A	0.180	-0.091	0.022	2.72E-05		0.984
DQB1	26	Y	P	A	0.180	-0.091	0.022	2.72E-05		0.985
DQB1	28		S	T	0.254	0.161	0.019	1.36E-17		1.000
DQB1	30	S	P	A	0.254	0.161	0.019	1.36E-17		1.000
DQB1	30	Y	P	A	0.500	-0.097	0.017	7.39E-09		0.939
DQB1	37	I	P	A	0.254	0.161	0.019	1.36E-17		1.000
DQB1	37	Y	P	A	0.743	-0.157	0.019	4.54E-17		0.999
DQB1	38		V	A	0.409	0.106	0.017	2.28E-10		0.996
DQB1	45		E	G	0.177	-0.090	0.022	4.02E-05		0.985
DQB1	46		E	V	0.254	0.161	0.019	1.35E-17		0.999
DQB1	47		F	Y	0.254	0.161	0.019	1.35E-17		0.999
DQB1	52		L	P	0.254	0.161	0.019	1.35E-17		0.999
DQB1	53		Q	L	0.395	-0.096	0.017	1.31E-08		0.997
DQB1	55	L	P	A	0.254	0.161	0.019	1.35E-17		0.999
DQB1	55	R	P	A	0.417	-0.081	0.017	1.57E-06		0.997
DQB1	57	A	P	A	0.401	0.139	0.017	3.18E-16		0.982
DQB1	57	AS	P	A	0.411	0.134	0.017	1.74E-15	7.98E-15	0.981
DQB1	57	AV	P	A	0.552	0.117	0.017	5.16E-12		0.952
DQB1	57	D	P	A	0.438	-0.114	0.017	1.45E-11		0.950
DQB1	66		D	E	0.281	0.165	0.018	1.43E-19		0.998
DQB1	67		I	V	0.281	0.165	0.018	1.43E-19		0.998
DQB1	70	G	P	A	0.358	-0.099	0.018	1.80E-08		0.965
DQB1	70	R	P	A	0.618	0.082	0.017	2.28E-06		0.966
DQB1	71	KD	P	A	0.278	0.168	0.018	4.08E-20		0.998
DQB1	71	K	P	A	0.254	0.161	0.019	1.35E-17	**9.77E-19**	0.999
DQB1	71	T	P	A	0.572	-0.120	0.017	5.26E-13		0.995
DQB1	71	KA	P	A	0.404	0.108	0.017	9.14E-11		0.996
DQB1	74	A	P	A	0.254	0.161	0.019	1.35E-17		0.999
DQB1	74	E	P	A	0.572	-0.120	0.017	5.25E-13		0.995
DQB1	75		V	L	0.428	0.120	0.017	5.30E-13		0.995
DQB1	77		R	T	0.406	0.108	0.017	1.18E-10		0.995
DQB1	84	Q	P	A	0.605	0.097	0.017	1.32E-08		0.996
DQB1	84	E	P	A	0.393	-0.096	0.017	1.55E-08		0.996
DQB1	85	L	P	A	0.605	0.096	0.017	1.32E-08		0.997
DQB1	85	V	P	A	0.393	-0.096	0.017	1.55E-08		0.996

DQB1	86	EG	P	A	0.637	0.101	0.018	9.60E-09	2.46E-08	0.965
DQB1	86	A	P	A	0.361	-0.100	0.018	1.13E-08		0.965
DQB1	86	E	P	A	0.605	0.096	0.017	1.33E-08		0.996
DQB1	86	Ex	P	A	0.607	0.096	0.017	1.55E-08		0.996
DQB1	87	L	P	A	0.605	0.096	0.017	1.32E-08	5.27E-09	0.997
DQB1	87	Lx	P	A	0.606	0.096	0.017	1.55E-08		0.997
DQB1	87	LY	P	A	0.786	0.108	0.020	1.02E-07		0.956
DQB1	87	F	P	A	0.212	-0.108	0.020	1.13E-07		0.955
DQB1	89	T	P	A	0.605	0.097	0.017	1.32E-08		0.997
DQB1	89	G	P	A	0.393	-0.096	0.017	1.55E-08		0.996
DQB1	90	T	P	A	0.605	0.096	0.017	1.31E-08		0.997
DQB1	90	I	P	A	0.394	-0.096	0.017	1.54E-08		0.997
DQB1	125	A	P	A	0.605	0.096	0.017	1.31E-08	1.86E-08	0.998
DQB1	125	Ax	P	A	0.606	0.096	0.017	1.54E-08		0.997
DQB1	125	AS	P	A	0.752	0.096	0.019	3.50E-07		0.989
DQB1	125	G	P	A	0.244	-0.097	0.019	3.66E-07		0.998
DQB1	167	R	P	A	0.817	0.092	0.022	1.82E-05		0.987
DQB1	167	H	P	A	0.181	-0.092	0.022	2.13E-05		0.988
DQB1	203	I	P	A	0.606	0.096	0.017	2.05E-08		0.991
DQB1	203	V	P	A	0.391	-0.095	0.017	2.46E-08		0.997
DQB1	220	H	P	A	0.603	0.097	0.017	1.44E-08		0.985
DQB1	220	R	P	A	0.394	-0.096	0.017	1.62E-08		0.996
DQB1	221	H	P	A	0.604	0.097	0.017	1.50E-08		0.986
DQB1	221	Q	P	A	0.394	-0.096	0.017	1.59E-08		0.995
DRB1	-1	S	P	A	0.149	-0.135	0.023	4.34E-09		0.994
DRB1	-1	A	P	A	0.814	0.102	0.022	2.90E-06		0.921
DRB1	-16	A	P	A	0.501	-0.100	0.016	1.04E-09		0.986
DRB1	-16	V	P	A	0.462	0.094	0.017	2.38E-08		0.944
DRB1	-17	T	P	A	0.162	-0.138	0.022	7.76E-10		0.977
DRB1	-17	A	P	A	0.801	0.107	0.021	5.93E-07		0.911
DRB1	-25	K	P	A	0.501	-0.100	0.016	1.04E-09		0.986
DRB1	-25	R	P	A	0.462	0.094	0.017	2.38E-08		0.944
DRB1	9	W	P	A	0.300	-0.084	0.018	2.87E-06		0.985
DRB1	9	E	P	A	0.687	0.083	0.018	2.92E-06		0.985
DRB1	10	Q	P	A	0.501	-0.100	0.016	1.03E-09		0.987
DRB1	10	Y	P	A	0.493	0.100	0.016	1.40E-09		0.987
DRB1	11	SG	P	A	0.632	0.112	0.017	6.43E-11	1.68E-10	0.986
DRB1	11	SGD	P	A	0.644	0.112	0.017	6.57E-11		0.986
DRB1	11	SGL	P	A	0.638	0.108	0.017	3.04E-10		0.999
DRB1	11	PV	P	A	0.350	-0.108	0.017	3.08E-10		0.999

DRB1	11	PL	P	A	0.162	-0.138	0.022	7.76E-10		0.977
DRB1	11	S	P	A	0.493	0.100	0.016	1.40E-09		0.987
DRB1	11	SVG	P	A	0.826	0.132	0.022	1.62E-09		0.978
DRB1	11	SD	P	A	0.506	0.099	0.016	1.90E-09		0.987
DRB1	11	P	P	A	0.156	-0.133	0.023	4.13E-09		1.000
DRB1	11	SL	P	A	0.499	0.095	0.016	6.21E-09		0.999
DRB1	11	SLD	P	A	0.511	0.094	0.016	8.19E-09		0.999
DRB1	11	PD	P	A	0.168	-0.126	0.022	8.23E-09		1.000
DRB1	11	SVD	P	A	0.700	0.084	0.018	2.87E-06		0.985
DRB1	11	SV	P	A	0.687	0.083	0.018	2.92E-06		0.985
DRB1	11	PG	P	A	0.295	-0.080	0.018	9.14E-06		1.000
DRB1	11	SVL	P	A	0.693	0.079	0.018	9.24E-06		1.000
DRB1	12		T	K	0.493	0.100	0.016	1.40E-09		0.987
DRB1	13	SYG	P	A	0.632	0.112	0.017	6.43E-11		0.986
DRB1	13	RH	P	A	0.345	-0.110	0.017	1.94E-10		1.000
DRB1	13	SY	P	A	0.584	0.105	0.017	5.56E-10		0.941
DRB1	13	SG	P	A	0.493	0.100	0.016	1.40E-09		0.987
DRB1	13	SYF	P	A	0.607	0.103	0.017	1.91E-09		0.953
DRB1	13	S	P	A	0.445	0.100	0.017	3.29E-09		0.942
DRB1	13	RF	P	A	0.180	-0.127	0.022	3.68E-09		0.978
DRB1	13	R	P	A	0.156	-0.133	0.023	4.13E-09	2.63E-10	1.000
DRB1	13	SFG	P	A	0.517	0.095	0.016	6.11E-09		1.000
DRB1	13	SF	P	A	0.469	0.094	0.017	1.66E-08		0.955
DRB1	13	SHY	P	A	0.772	0.104	0.020	2.49E-07		0.919
DRB1	13	RG	P	A	0.204	-0.105	0.021	4.24E-07		0.932
DRB1	13	SHG	P	A	0.682	0.082	0.018	4.16E-06		0.985
DRB1	13	RY	P	A	0.295	-0.080	0.018	9.14E-06		1.000
DRB1	13	SH	P	A	0.634	0.074	0.017	2.29E-05		0.938
DRB1	32		H	Y	0.392	0.112	0.019	9.77E-09		0.734
DRB1	37	SL	P	A	0.184	-0.149	0.022	1.41E-11		0.911
DRB1	37	N	P	A	0.361	0.135	0.020	1.99E-11		0.664
DRB1	37	NF	P	A	0.516	0.122	0.019	1.48E-10		0.703
DRB1	37	S	P	A	0.169	-0.143	0.023	3.03E-10	8.72E-11	0.934
DRB1	37	NL	P	A	0.376	0.121	0.020	1.28E-09		0.675
DRB1	37	SY	P	A	0.469	-0.112	0.019	4.53E-09		0.714
DRB1	37	NY	P	A	0.662	0.100	0.018	3.47E-08		0.889
DRB1	37	SF	P	A	0.323	-0.093	0.018	4.46E-07		0.898
DRB1	70	D	P	A	0.523	0.128	0.021	2.36E-09		0.488
DRB1	70	Q	P	A	0.444	-0.127	0.022	5.36E-09		0.471
DRB1	71	E	P	A	0.241	0.174	0.025	8.01E-12	2.24E-11	0.445

DRB1	71	A	P	A	0.127	-0.164	0.028	4.00E-09	0.785
DRB1	71	KA	P	A	0.367	-0.152	0.026	7.71E-09	0.328
DRB1	71	KE	P	A	0.481	0.126	0.022	9.04E-09	0.480
DRB1	96	H	P	A	0.612	0.124	0.018	2.22E-12	0.867
DRB1	96	Hx	P	A	0.626	0.120	0.017	6.27E-12	0.912
DRB1	96	HE	P	A	0.637	0.114	0.018	7.07E-11	0.946
DRB1	96	HY	P	A	0.801	0.145	0.023	1.21E-10	0.796
DRB1	96	QE	P	A	0.186	-0.143	0.022	2.25E-10	0.864
DRB1	96	QY	P	A	0.350	-0.109	0.017	2.33E-10	0.997
DRB1	96	Qx	P	A	0.175	-0.133	0.023	3.59E-09	0.910
DRB1	96	Q	P	A	0.162	-0.128	0.022	9.23E-09	0.999
DRB1	133	R	P	A	0.818	0.141	0.023	7.57E-10	0.834
DRB1	133	L	P	A	0.156	-0.133	0.023	3.98E-09	0.999
DRB1	140	T	P	A	0.643	0.096	0.018	2.15E-07	0.815
DRB1	140	A	P	A	0.331	-0.091	0.018	6.32E-07	0.907
DRB1	142	V	P	A	0.818	0.141	0.023	7.57E-10	0.834
DRB1	142	M	P	A	0.156	-0.133	0.023	3.98E-09	0.999
DRB1	149	H	P	A	0.449	0.119	0.017	1.05E-11	0.837
DRB1	149	Q	P	A	0.525	-0.109	0.017	1.16E-10	0.919
DRB1	233	T	P	A	0.626	-0.124	0.017	4.27E-13	0.985
DRB1	233	R	P	A	0.335	0.117	0.018	5.48E-11	0.954

Table S24: TWAS results for antigen response phenotypes that were analyzed using gene expression models based on whole blood and brain tissues. Associations with $P_{TWAS}<4.2\times10^{-6}$ are considered statistically significant and genes with $P_{TWAS}<4.5\times10^{-5}$ are considered suggestive.

Antigen	Region	Gene ID	Gene	Whole Blood		Brain (Frontal Cortex)	
				Z	P_{TWAS}	Z	P_{TWAS}
BKV	19q13.33	ENSG00000176909	*MAMSTR*	-4.22	2.4×10^{-5}	-	-
	7q21.2	ENSG00000004766	*VPS50*	4.09	4.4×10^{-5}	-0.33	0.74
	19q13.33	ENSG00000142233	*NTN5*	-2.33	0.020	6.09	1.1×10^{-9}
	19q13.33	ENSG00000176920	*FUT2*	-	-	7.16	8.1×10^{-13}
	20q13.2	ENSG00000054803	*CBLN4*	-	-	4.09	4.4×10^{-5}
CMV pp150	16p13.3	ENSG00000172366	*MCRIP2*	-0.44	0.66	-4.10	4.1×10^{-5}
CMV pp52	3q13.12	ENSG00000196776	*CD47*	-4.49	7.3×10^{-6}	-	-
HHV6 IE1A	6p21.32	ENSG00000196735	*HLA-DQA1*	4.29	1.8×10^{-5}	4.34	1.4×10^{-5}
	6p21.33	ENSG00000204525	*HLA-C*	-2.97	3.0×10^{-3}	-4.25	2.1×10^{-5}
HHV6 p101	4q13.3	ENSG00000163734	*CXCL3*	4.12	3.8×10^{-5}	4.12	3.8×10^{-5}
HHV7	6p21.32	ENSG00000232629	*HLA-DQB2*	5.38	7.3×10^{-8}	5.29	1.2×10^{-7}
	6p21.32	ENSG00000179344	*HLA-DQB1*	-5.07	3.9×10^{-7}	0.64	0.52
	22q13.2	ENSG00000273424	*CTA-223H9.9*	-4.71	2.5×10^{-6}	-4.71	2.5×10^{-6}
	22q13.2	ENSG00000167074	*TEF*	4.66	3.1×10^{-6}	-	-
	22q13.33	ENSG00000025708	*TYMP*	4.54	5.6×10^{-6}	3.98	6.8×10^{-5}
	6p21.32	ENSG00000204267	*TAP2*	-4.48	7.4×10^{-6}	1.90	0.058
	17q21.32	ENSG00000198933	*TBKBP1*	-4.41	1.0×10^{-5}	-4.21	2.6×10^{-5}
	22q13.33	ENSG00000177989	*ODF3B*	4.30	1.7×10^{-5}	3.01	2.6×10^{-3}
	22q13.2	ENSG00000100417	*PMM1*	-4.26	2.0×10^{-5}	-1.90	0.057
	6p21.33	ENSG00000204428	*LY6G5C*	4.24	2.3×10^{-5}	4.24	2.3×10^{-5}
	22q13.2	ENSG00000172346	*CSDC2*	4.14	3.4×10^{-5}	4.67	3.0×10^{-6}
	1q31.2	ENSG00000090104	*RGS1*	2.70	6.9×10^{-3}	-4.65	3.3×10^{-6}
	2q11.2	ENSG00000198885	*ITPRIPL1*	-2.66	7.9×10^{-3}	-4.09	4.4×10^{-5}
	6p21.32	ENSG00000204261	*PSMB8-AS1*	2.38	0.017	-5.06	4.1×10^{-7}
	6p21.32	ENSG00000241404	*EGFL8*	-0.83	0.41	-5.36	8.4×10^{-8}
HSV1	6p21.32	ENSG00000179344	*HLA-DQB1*	-5.46	4.8×10^{-8}	-4.62	3.9×10^{-6}
	6p21.32	ENSG00000232629	*HLA-DQB2*	5.11	3.3×10^{-7}	4.99	5.9×10^{-7}
	6p21.32	ENSG00000204257	*HLA-DMA*	4.93	8.2×10^{-7}	4.88	1.0×10^{-6}
	6p21.33	ENSG00000204444	*APOM*	-4.71	2.5×10^{-6}	-4.60	4.3×10^{-6}
	6p21.33	ENSG00000204386	*NEU1*	-4.44	9.1×10^{-6}	-	-
	6p21.33	ENSG00000231852	*CYP21A2*	4.41	1.0×10^{-5}	-	-
	6p21.33	ENSG00000224389	*C4B*	4.40	1.1×10^{-5}	-	-
	6p21.33	ENSG00000166278	*C2*	4.38	1.2×10^{-5}	-	-
	6p21.33	ENSG00000213719	*CLIC1*	-4.34	1.5×10^{-5}	-4.34	1.5×10^{-5}
	6p21.32	ENSG00000204305	*AGER*	4.13	3.6×10^{-5}	2.96	3.0×10^{-3}

	6p21.33	ENSG00000244731	*C4A*	-4.09	4.3×10^{-5}	-4.29	1.8×10^{-5}
	6p21.32	ENSG00000204308	*RNF5*	-1.92	0.055	-4.82	1.5×10^{-6}
	6p21.32	ENSG00000196735	*HLA-DQA1*	-1.80	0.072	-4.25	2.1×10^{-5}
	6p21.32	ENSG00000242574	*HLA-DMB*	-0.81	0.42	5.00	5.6×10^{-7}
	6p21.32	ENSG00000196126	*HLA-DRB1*	-	-	-4.50	6.8×10^{-6}
JCV	6p21.32	ENSG00000237541	*HLA-DQA2*	7.09	1.3×10^{-12}	-	-
	6p21.33	ENSG00000204371	*EHMT2*	-6.95	3.7×10^{-12}	-	-
	6p21.32	ENSG00000204305	*AGER*	6.14	8.0×10^{-10}	7.45	9.5×10^{-14}
	6p21.32	ENSG00000232629	*HLA-DQB2*	6.11	9.9×10^{-10}	5.97	2.4×10^{-9}
	6p21.32	ENSG00000179344	*HLA-DQB1*	-5.88	4.2×10^{-9}	-0.31	0.76
	6p21.33	ENSG00000213722	*DDAH2*	-5.44	5.5×10^{-8}	-1.34	0.18
	6p21.32	ENSG00000204308	*RNF5*	-5.02	5.2×10^{-7}	-1.74	0.082
	6p21.32	ENSG00000204310	*AGPAT1*	-4.85	1.2×10^{-6}	5.07	4.1×10^{-7}
	6p21.33	ENSG00000198563	*DDX39B*	-4.84	1.3×10^{-6}	-0.43	0.67
	6p21.32	ENSG00000240065	*PSMB9*	4.47	7.7×10^{-6}	2.01	0.045
	6p21.33	ENSG00000213760	*ATP6V1G2*	2.80	5.1×10^{-3}	-4.46	8.3×10^{-6}
	11q13.4	ENSG00000214517	*PPME1*	2.22	0.026	4.31	1.6×10^{-5}
	6p21.32	ENSG00000204264	*PSMB8*	1.99	0.047	4.17	3.0×10^{-5}
	6p21.32	ENSG00000204252	*HLA-DOA*	-0.68	0.50	-4.56	5.2×10^{-6}
	6p21.32	ENSG00000204315	*FKBPL*	0.01	0.99	-4.53	6.0×10^{-6}
	6p21.32	ENSG00000196126	*HLA-DRB1*	-	-	-7.50	6.5×10^{-14}
	6p21.33	ENSG00000204540	*PSORS1C1*	-	-	-4.69	2.8×10^{-6}
VZV	6p21.33	ENSG00000204444	*APOM*	-10.73	7.5×10^{-27}	-10.47	1.1×10^{-25}
	6p21.33	ENSG00000231852	*CYP21A2*	10.53	6.5×10^{-26}	-	-
	6p21.33	ENSG00000224389	*C4B*	10.47	1.2×10^{-25}	-	-
	6p21.33	ENSG00000166278	*C2*	10.29	8.1×10^{-25}	-	-
	6p21.33	ENSG00000204386	*NEU1*	-10.28	8.4×10^{-25}	-	-
	6p21.33	ENSG00000213719	*CLIC1*	-10.22	1.6×10^{-24}	-10.22	1.6×10^{-24}
	6p21.33	ENSG00000244731	*C4A*	-10.19	2.3×10^{-24}	-10.16	2.9×10^{-24}
	6p22.1	ENSG00000204613	*TRIM10*	-8.82	1.1×10^{-18}	-	-
	6p21.32	ENSG00000204305	*AGER*	8.69	3.6×10^{-18}	4.44	9.1×10^{-6}
	6p21.33	ENSG00000214894	*LINC00243*	8.01	1.1×10^{-15}	-	-
	6p22.1	ENSG00000281831	*HCP5B*	7.90	2.8×10^{-15}	1.53	0.13
	6p22.1	ENSG00000187626	*ZKSCAN4*	7.84	4.5×10^{-15}	7.24	4.4×10^{-13}
	6p22.1	ENSG00000204681	*GABBR1*	7.29	3.1×10^{-13}	1.02	0.31
	6p22.1	ENSG00000204657	*OR2H2*	7.09	1.3×10^{-12}	0.27	0.79
	6p22.1	ENSG00000204644	*ZFP57*	7.09	1.4×10^{-12}	-	-
	6p21.33	ENSG00000204435	*CSNK2B*	7.06	1.7×10^{-12}	-4.09	4.2×10^{-5}
	6p22.2	ENSG00000186470	*BTN3A2*	-6.91	4.8×10^{-12}	-6.72	1.8×10^{-11}
	6p21.32	ENSG00000204257	*HLA-DMA*	6.75	1.5×10^{-11}	6.76	1.3×10^{-11}

	6p22.1	ENSG00000158691	*ZSCAN12*	-6.73	$1.7×10^{-11}$	5.45	$5.0×10^{-8}$
	6p21.32	ENSG00000204304	*PBX2*	-6.28	$3.5×10^{-10}$	-1.48	0.14
	6p22.1	ENSG00000189298	*ZKSCAN3*	6.20	$5.7×10^{-10}$	-2.35	0.019
	6p22.1	ENSG00000206503	*HLA-A*	-6.14	$8.2×10^{-10}$	0.13	0.89
	6p22.1	ENSG00000261353	*CTA-14H9.5*	-5.96	$2.5×10^{-9}$	-	-
	6p21.32	ENSG00000179344	*HLA-DQB1*	-5.96	$2.6×10^{-9}$	-6.33	$2.5×10^{-10}$
	6p22.1	ENSG00000137185	*ZSCAN9*	-5.86	$4.7×10^{-9}$	7.84	$4.6×10^{-15}$
	6p21.32	ENSG00000232629	*HLA-DQB2*	5.79	$7.0×10^{-9}$	5.61	$2.1×10^{-8}$
	6p21.33	ENSG00000204536	*CCHCR1*	5.72	$1.1×10^{-8}$	7.34	$2.2×10^{-13}$
	6p22.1	ENSG00000197062	*ZSCAN26*	-5.57	$2.5×10^{-8}$	-5.71	$1.1×10^{-8}$
	6p21.33	ENSG00000204463	*BAG6*	5.52	$3.3×10^{-8}$	-4.29	$1.8×10^{-5}$
	6p22.2	ENSG00000180596	*HIST1H2BC*	-5.46	$4.8×10^{-8}$	-2.38	0.017
	6p22.1	ENSG00000204655	*MOG*	5.42	$5.9×10^{-8}$	-	-
	6p22.2	ENSG00000272462	*U91328.19*	5.02	$5.1×10^{-7}$	2.19	0.028
	6p21.33	ENSG00000204396	*VWA7*	-4.90	$9.5×10^{-7}$	5.72	$1.0×10^{-8}$
	6p21.33	ENSG00000204388	*HSPA1B*	4.89	$1.0×10^{-6}$	-	-
	6p21.32	ENSG00000221988	*PPT2*	4.79	$1.6×10^{-6}$	-1.48	0.14
	6p22.1	ENSG00000272468	*RP1-86C11.7*	-4.61	$3.9×10^{-6}$	1.12	0.26
	6p22.1	ENSG00000234127	*TRIM26*	4.56	$5.0×10^{-6}$	1.58	0.11
	6p22.1	ENSG00000137338	*PGBD1*	4.56	$5.2×10^{-6}$	1.34	0.18
	6p22.2	ENSG00000010704	*HFE*	-4.42	$9.8×10^{-6}$	-1.15	0.25
	6p22.1	ENSG00000241370	*RPP21*	4.41	$1.0×10^{-5}$	-3.91	$9.3×10^{-5}$
	6p21.33	ENSG00000204371	*EHMT2*	-4.36	$1.3×10^{-5}$	-	-
	6p22.1	ENSG00000204632	*HLA-G*	4.24	$2.2×10^{-5}$	-0.64	0.52
	6p22.2	ENSG00000124508	*BTN2A2*	4.22	$2.5×10^{-5}$	-1.95	0.051
	6p21.32	ENSG00000204310	*AGPAT1*	-3.70	$2.2×10^{-4}$	4.26	$2.0×10^{-5}$
	6p21.33	ENSG00000204387	*C6orf48*	-2.96	$3.1×10^{-3}$	-4.18	$3.0×10^{-5}$
	6p21.32	ENSG00000204264	*PSMB8*	2.89	$3.8×10^{-3}$	4.83	$1.3×10^{-6}$
	6p21.33	ENSG00000213722	*DDAH2*	-2.46	0.014	-8.27	$1.3×10^{-16}$
	6p21.32	ENSG00000204301	*NOTCH4*	-2.37	0.018	-6.98	$3.0×10^{-12}$
	6p21.33	ENSG00000204420	*MPIG6B*	2.10	0.036	-5.68	$1.4×10^{-8}$
	6p22.1	ENSG00000112812	*PRSS16*	2.03	0.043	4.94	$7.7×10^{-7}$
	6p22.1	ENSG00000198315	*ZKSCAN8*	1.66	0.097	-4.90	$9.5×10^{-7}$
	6p21.33	ENSG00000137411	*VARS2*	-1.64	0.10	-5.17	$2.3×10^{-7}$
	6p21.33	ENSG00000204410	*MSH5*	-1.45	0.15	-7.81	$5.7×10^{-15}$
	6p21.32	ENSG00000213676	*ATF6B*	-1.36	0.18	-4.38	$1.2×10^{-5}$
	6p21.33	ENSG00000137312	*FLOT1*	1.22	0.22	6.32	$2.6×10^{-10}$
	6p21.33	ENSG00000226979	*LTA*	-0.97	0.33	4.09	$4.3×10^{-5}$
	6p21.33	ENSG00000213760	*ATP6V1G2*	0.83	0.40	-5.43	$5.8×10^{-8}$
	6p21.32	ENSG00000204308	*RNF5*	-0.69	0.49	-9.65	$5.2×10^{-22}$

	6p21.32	ENSG00000242574	*HLA-DMB*	0.55	0.58	6.63	3.3×10^{-11}
	6p21.32	ENSG00000196735	*HLA-DQA1*	-0.37	0.71	-5.22	1.8×10^{-7}
	6p21.33	ENSG00000228022	*HCG20*	-	-	-5.62	1.9×10^{-8}
	6p21.33	ENSG00000204540	*PSORS1C1*	-	-	-5.09	3.6×10^{-7}
	6p21.33	ENSG00000204531	*POU5F1*	-	-	4.23	2.4×10^{-5}
	10p14	ENSG00000151657	*KIN*	-	-	4.14	3.5×10^{-5}

Table S25: TWAS results for EBV antigen response phenotypes that were analyzed using gene expression models based on whole blood, brain tissues, and EBV-transformed lymphocytes. Associations with $P_{TWAS}<4.2×10^{-6}$ are considered statistically significant and genes with $P_{TWAS}<4.5×10^{-5}$ are considered suggestive.

Antigen	Region	Gene ID	Gene	Whole Blood		Brain (Frontal Cortex)		EBV Lymphocytes	
				Z	P_{WAS}	Z	P_{TWAS}	Z	P_{TWAS}
EBV EA-D	6p21.32	ENSG00000204257	*HLA-DMA*	-5.91	$3.5×10^{-9}$	-5.93	$3.0×10^{-9}$	5.88	$4.0×10^{-9}$
	6p21.33	ENSG00000204444	*APOM*	5.86	$4.6×10^{-9}$	5.73	$1.0×10^{-8}$	5.45	$5.1×10^{-8}$
	6p21.33	ENSG00000166278	*C2*	-5.70	$1.2×10^{-8}$	-	-	-	-
	6p21.33	ENSG00000244731	*C4A*	5.66	$1.5×10^{-8}$	5.55	$2.9×10^{-8}$	5.59	$2.3×10^{-8}$
	6p21.33	ENSG00000204386	*NEU1*	5.59	$2.3×10^{-8}$	-	-	-1.65	0.099
	6p21.33	ENSG00000213719	*CLIC1*	5.55	$2.9×10^{-8}$	5.55	$2.9×10^{-8}$	1.14	0.25
	6p21.33	ENSG00000224389	*C4B*	-5.43	$5.6×10^{-8}$	-	-	-	-
	6p21.33	ENSG00000231852	*CYP21A2*	-5.41	$6.4×10^{-8}$	-	-	-	-
	6p22.1	ENSG00000281831	*HCP5B*	-4.66	$3.2×10^{-6}$	-5.30	$1.1×10^{-7}$	-6.01	$1.9×10^{-9}$
	6p22.1	ENSG00000204625	*HCG9*	-4.52	$6.2×10^{-6}$	-	-	-	-
	6p22.1	ENSG00000204613	*TRIM10*	4.48	$7.6×10^{-6}$	-	-	-	-
	6p21.33	ENSG00000204388	*HSPA1B*	-4.43	$9.3×10^{-6}$	-	-	1.61	0.11
	6p21.33	ENSG00000214894	*LINC00243*	-4.38	$1.2×10^{-5}$	-	-	1.19	0.23
	6q22.1	ENSG00000188820	*FAM26F*	4.26	$2.0×10^{-5}$	4.68	$2.8×10^{-6}$	3.41	$6.4×10^{-4}$
	6p21.33	ENSG00000204536	*CCHCR1*	-3.77	$1.7×10^{-4}$	-4.75	$2.1×10^{-6}$	0.40	0.69
	20q11.23	ENSG00000080839	*RBL1*	-2.68	$7.3×10^{-3}$	-2.28	0.022	-4.10	$4.2×10^{-5}$
	12q23.1	ENSG00000139343	*SNRPF*	-2.46	0.014	-4.12	$3.9×10^{-5}$	-4.13	$3.6×10^{-5}$
	6p21.33	ENSG00000204410	*MSH5*	-2.40	0.016	5.71	$1.2×10^{-8}$	-1.32	0.19
	6p21.32	ENSG00000242574	*HLA-DMB*	2.00	0.046	-5.80	$6.8×10^{-9}$	-2.59	$9.7×10^{-3}$
	6p21.32	ENSG00000232629	*HLA-DQB2*	1.48	0.14	1.31	0.19	4.15	$3.3×10^{-5}$
	6p21.33	ENSG00000204482	*LST1*	0.87	0.39	-	-	-4.34	$1.4×10^{-5}$
	6p21.33	ENSG00000204560	*DHX16*	0.72	0.47	0.07	0.94	-4.17	$3.1×10^{-5}$
	6p21.32	ENSG00000198502	*HLA-DRB5*	-	-	-	-	-5.85	$4.8×10^{-9}$
	6p21.33	ENSG00000204531	*POU5F1*	-	-	-1.64	0.10	-5.40	$6.7×10^{-8}$
	6p21.33	ENSG00000204540	*PSORS1C1*	-	-	4.43	$9.6×10^{-6}$	4.67	$3.0×10^{-6}$
	6p21.33	ENSG00000228022	*HCG20*	-	-	4.10	$4.2×10^{-5}$	3.58	$3.5×10^{-4}$
	6p21.32	ENSG00000196126	*HLA-DRB1*	-	-	-4.38	$1.2×10^{-5}$	-	-
EBV EBNA	6p21.32	ENSG00000179344	*HLA-DQB1*	13.30	$2.2×10^{-40}$	11.46	$2.2×10^{-30}$	10.67	$1.4×10^{-26}$
	6p21.32	ENSG00000232629	*HLA-DQB2*	-12.19	$3.6×10^{-34}$	-12.16	$5.0×10^{-34}$	-3.78	$1.6×10^{-4}$
	6p21.32	ENSG00000204305	*AGER*	-10.70	$9.9×10^{-27}$	-8.91	$5.1×10^{-19}$	-7.28	3.4^{-13}
	6p21.33	ENSG00000204371	*EHMT2*	9.43	$4.0×10^{-21}$	-	-	-	-
	6p21.33	ENSG00000213722	*DDAH2*	9.00	$2.2×10^{-19}$	7.76	$8.6×10^{-15}$	0.01	1.00
	6p21.33	ENSG00000204435	*CSNK2B*	-7.62	$2.5×10^{-14}$	3.36	$7.8×10^{-4}$	-1.61	0.11
	6p21.32	ENSG00000204308	*RNF5*	7.53	$5.0×10^{-14}$	9.45	$3.6×10^{-21}$	10.17	$2.8×10^{-24}$

	6p21.32	ENSG00000204304	*PBX2*	7.31	$2.7{\times}10^{-13}$	4.05	$5.1{\times}10^{-5}$	4.05	$5.1{\times}10^{-5}$
	6p21.33	ENSG00000204444	*APOM*	7.30	$2.8{\times}10^{-13}$	7.16	$7.8{\times}10^{-13}$	6.14	$8.2{\times}10^{-10}$
	6p21.32	ENSG00000204310	*AGPAT1*	7.30	$3.0{\times}10^{-13}$	-7.57	$3.7{\times}10^{-14}$	-	-
	6p21.33	ENSG00000204463	*BAG6*	-7.07	$1.5{\times}10^{-12}$	3.26	$1.1{\times}10^{-3}$	2.51	0.012
	6p21.32	ENSG00000204315	*FKBPL*	-6.74	$1.6{\times}10^{-11}$	4.81	$1.5{\times}10^{-6}$	-	-
	6p21.33	ENSG00000224389	*C4B*	-6.43	$1.3{\times}10^{-10}$	-	-	-	-
	6p21.33	ENSG00000231852	*CYP21A2*	-6.41	$1.4{\times}10^{-10}$	-	-	-	-
	6p21.33	ENSG00000213719	*CLIC1*	6.21	$5.3{\times}10^{-10}$	6.21	$5.3{\times}10^{-10}$	6.98	$3.0{\times}10^{-12}$
	6p21.33	ENSG00000166278	*C2*	-6.21	$5.3{\times}10^{-10}$	-	-	-	-
	6p21.33	ENSG00000204386	*NEU1*	6.14	$8.2{\times}10^{-10}$	-	-	-2.05	0.040
	6p21.33	ENSG00000204536	*CCHCR1*	-5.94	$2.9{\times}10^{-9}$	-5.82	$6.0{\times}10^{-9}$	3.30	$9.7{\times}10^{-4}$
	6p21.33	ENSG00000227507	*LTB*	5.77	$7.8{\times}10^{-9}$	-	-	-	-
	3q25.1	ENSG00000169313	*P2RY12*	5.53	$3.3{\times}10^{-8}$	5.20	$2.0{\times}10^{-7}$	5.20	$2.0{\times}10^{-7}$
	6p21.33	ENSG00000244731	*C4A*	5.30	$1.2{\times}10^{-7}$	5.83	$5.6{\times}10^{-9}$	7.18	$6.8{\times}10^{-13}$
	6p21.32	ENSG00000236104	*ZBTB22*	-5.13	$2.9{\times}10^{-7}$	-3.73	$1.9{\times}10^{-4}$	-	-
	6p21.32	ENSG00000204314	*PRRT1*	-5.02	$5.1{\times}10^{-7}$	-	-	-9.99	$1.6{\times}10^{-23}$
	6p21.33	ENSG00000204396	*VWA7*	4.96	$7.0{\times}10^{-7}$	-6.57	$4.9{\times}10^{-11}$	0.42	0.68
	6p21.32	ENSG00000204209	*DAXX*	4.94	$7.8{\times}10^{-7}$	0.19	0.85	4.55	$5.3{\times}10^{-6}$
	6p21.32	ENSG00000237541	*HLA-DQA2*	-4.84	$1.3{\times}10^{-6}$	-	-	-	-
EBV EBNA	6p21.32	ENSG00000204257	*HLA-DMA*	-4.69	$2.7{\times}10^{-6}$	-4.54	$5.5{\times}10^{-6}$	4.64	$3.5{\times}10^{-6}$
	6p21.32	ENSG00000227057	*WDR46*	-4.54	$5.6{\times}10^{-6}$	-1.86	0.063	-1.86	0.063
	6p21.33	ENSG00000204366	*ZBTB12*	-4.44	$8.9{\times}10^{-6}$	3.02	$2.5{\times}10^{-3}$	0.26	0.80
	6p21.33	ENSG00000204420	*MPIG6B*	-4.44	$9.0{\times}10^{-6}$	2.89	$3.8{\times}10^{-3}$	4.44	$9.0{\times}10^{-6}$
	6p21.33	ENSG00000204344	*STK19*	4.44	$9.1{\times}10^{-6}$	1.21	0.23	-	-
	6p21.32	ENSG00000231925	*TAPBP*	-4.37	$1.2{\times}10^{-5}$	-5.18	$2.3{\times}10^{-7}$	5.42	$5.9{\times}10^{-8}$
	6p22.1	ENSG00000204625	*HCG9*	-4.25	$2.1{\times}10^{-5}$	-	-	-	-
	6p21.32	ENSG00000196735	*HLA-DQA1*	4.18	$2.9{\times}10^{-5}$	11.93	$8.8{\times}10^{-33}$	0.87	0.38
	6p21.32	ENSG00000221988	*PPT2*	-3.72	$2.0{\times}10^{-4}$	-0.59	0.55	-6.36	$2.0{\times}10^{-10}$
	6p22.1	ENSG00000281831	*HCP5B*	-3.49	$4.9{\times}10^{-4}$	-4.24	$2.3{\times}10^{-5}$	-5.08	$3.7{\times}10^{-7}$
	6p21.33	ENSG00000206344	*HCG27*	-3.22	$1.3{\times}10^{-3}$	0.74	0.46	-5.86	$4.6{\times}10^{-9}$
	6p21.33	ENSG00000232810	*TNF*	3.06	$2.2{\times}10^{-3}$	3.06	$2.2{\times}10^{-3}$	-7.43	$1.1{\times}10^{-13}$
	6p21.33	ENSG00000213760	*ATP6V1G2*	-3.04	$2.4{\times}10^{-3}$	3.58	$3.5{\times}10^{-4}$	-4.89	$9.9{\times}10^{-7}$
	6p21.32	ENSG00000204252	*HLA-DOA*	2.45	0.014	7.80	$6.3{\times}10^{-15}$	1.84	0.066
	6p21.33	ENSG00000204410	*MSH5*	-2.04	0.041	5.27	$1.4{\times}10^{-7}$	1.68	0.093
	6p21.32	ENSG00000231389	*HLA-DPA1*	-1.97	0.049	-5.49	$4.1{\times}10^{-8}$	0.33	0.74
	6p21.32	ENSG00000223865	*HLA-DPB1*	1.96	0.050	-6.35	$2.1{\times}10^{-10}$	-	-
	6p22.1	ENSG00000234127	*TRIM26*	1.34	0.18	-4.69	$2.7{\times}10^{-6}$	1.34	0.18
	6p21.32	ENSG00000242574	*HLA-DMB*	1.22	0.22	-5.05	$4.4{\times}10^{-7}$	-3.61	$3.1{\times}10^{-4}$
	6p21.33	ENSG00000204469	*PRRC2A*	-1.20	0.23	0.62	0.53	-7.58	$3.5{\times}10^{-14}$
	6p21.32	ENSG00000204228	*HSD17B8*	1.16	0.25	4.63	$3.7{\times}10^{-6}$	1.14	0.26

	6p21.33	ENSG00000204388	*HSPA1B*	1.05	0.30	-	-	4.46	8.4×10^{-6}
	6p21.33	ENSG00000226979	*LTA*	-0.75	0.45	-5.76	8.6×10^{-9}	-5.71	1.1×10^{-8}
	6p21.32	ENSG00000204231	*RXRB*	-0.67	0.50	4.54	5.7×10^{-6}	0.67	0.50
	3q25.1	ENSG00000181631	*P2RY13*	0.48	0.63	5.72	1.1×10^{-8}	-0.13	0.90
	6p21.32	ENSG00000204301	*NOTCH4*	0.36	0.72	4.41	1.0×10^{-5}	2.12	0.034
	6p21.33	ENSG00000204389	*HSPA1A*	-	-	-	-	8.81	1.3×10^{-18}
	6p21.33	ENSG00000204531	*POU5F1*			-4.32	1.5×10^{-5}	-6.23	4.7×10^{-10}
EBV EBNA	6p21.32	ENSG00000198502	*HLA-DRB5*	-	-	-	-	5.24	1.6×10^{-7}
	6p21.33	ENSG00000168631	*DPCR1*	-	-	-	-	4.92	8.5×10^{-7}
	6p21.33	ENSG00000204540	*PSORS1C1*	-	-	4.89	1.0×10-6	4.91	9.3×10^{-7}
	6p21.33	ENSG00000137337	*MDC1*	-	-	-	-	4.73	2.2×10^{-6}
	6p21.32	ENSG00000204287	*HLA-DRA*	-	-	-	-	4.67	3.0×10^{-6}
	6p21.32	ENSG00000168477	*TNXB*	-	-	-	-	4.25	2.2×10^{-5}
	6p21.33	ENSG00000228789	*HCG22*	-	-	-4.47	7.7×10^{-6}	3.27	1.1×10^{-3}
	6p21.32	ENSG00000196126	*HLA-DRB1*	-	-	13.05	6.7×10^{-39}	-	-
	6p22.1	ENSG00000270604	*HCG17*	-	-	4.16	3.3×10^{-5}	-	-
	6p21.33	ENSG00000213722	*DDAH2*	9.74	2.0×10^{-22}	6.03	1.6×10^{-9}	1.85	0.064
	6p21.33	ENSG00000204371	*EHMT2*	9.56	1.2×10^{-21}	-	-	-	-
	6p21.32	ENSG00000204305	*AGER*	-8.39	4.8×10^{-17}	-8.90	5.4×10^{-19}	-5.02	5.1×10^{-7}
	6p21.33	ENSG00000204463	*BAG6*	-8.20	2.5×10^{-16}	-2.14	0.032	-1.18	0.24
	6p21.33	ENSG00000204536	*CCHCR1*	-6.66	2.7×10^{-11}	-5.05	4.4×10^{-7}	0.09	0.93
	6p21.33	ENSG00000227507	*LTB*	6.65	3.0×10^{-11}	-	-	-	-
	6p21.33	ENSG00000213760	*ATP6V1G2*	-6.36	2.0×10^{-10}	2.65	8.1×10^{-3}	-7.02	2.2×10^{-12}
	6p21.32	ENSG00000179344	*HLA-DQB1*	5.79	7.1×10^{-9}	5.74	9.8×10^{-9}	4.65	3.3×10^{-6}
	6p21.33	ENSG00000137310	*TCF19*	-5.76	8.6×10^{-9}	-3.44	5.9×10^{-4}	3.65	2.6×10^{-4}
	6p21.32	ENSG00000204310	*AGPAT1*	5.72	1.1×10^{-8}	-6.10	1.1×10^{-9}	-	-
	6p21.32	ENSG00000232629	*HLA-DQB2*	-5.61	2.1×10^{-8}	-5.64	1.7×10^{-8}	-1.74	0.082
EBV p18	6p21.33	ENSG00000214894	*LINC00243*	-5.60	2.2×10^{-8}	-	-	-0.16	0.87
	6p21.32	ENSG00000237541	*HLA-DQA2*	-5.41	6.5×10^{-8}	-	-	-	-
	6p21.33	ENSG00000166278	*C2*	-5.35	8.9×10^{-8}	-	-	-	-
	6p22.2	ENSG00000186470	*BTN3A2*	5.33	9.9×10^{-8}	5.05	4.4×10^{-7}	5.25	1.5×10^{-7}
	6p21.33	ENSG00000213719	*CLIC1*	5.31	1.1×10^{-7}	5.31	1.1×10^{-7}	6.84	8.2×10^{-12}
	6p21.33	ENSG00000204386	*NEU1*	5.26	1.5×10^{-7}	-	-	-1.05	0.29
	6p21.33	ENSG00000204396	*VWA7*	5.21	1.9×10^{-7}	-6.23	4.8×10^{-10}	1.10	0.27
	6p21.33	ENSG00000204469	*PRRC2A*	-5.16	2.4×10^{-7}	0.77	0.44	-8.33	8.1×10^{-17}
	6p21.33	ENSG00000206344	*HCG27*	-5.11	3.3×10^{-7}	-1.61	0.11	-8.68	4.1×10^{-18}
	6p21.33	ENSG00000224389	*C4B*	-5.03	4.9×10^{-7}	-	-	-	-
	6p21.33	ENSG00000204444	*APOM*	4.97	6.6×10^{-7}	4.83	1.4-6	5.38	7.5×10^{-8}
	6p21.33	ENSG00000231852	*CYP21A2*	-4.95	7.3×10^{-7}	-	-	-	-
	6p21.32	ENSG00000204304	*PBX2*	4.83	1.4×10^{-6}	0.20	0.84	0.20	0.84

	6p22.2	ENSG00000124508	*BTN2A2*	-4.70	$2.6×10^{-6}$	-0.72	0.47	5.06	$4.2×10^{-7}$
	6p22.1	ENSG00000261353	*CTA-14H9.5*	4.67	$3.0×10^{-6}$	-	-	-3.65	$2.6×10^{-4}$
	6p22.1	ENSG00000137185	*ZSCAN9*	4.53	$6.0×10^{-6}$	-4.40	$1.1×10^{-5}$	4.40	$1.1×10^{-5}$
	6p22.1	ENSG00000197279	*ZNF165*	-4.50	$6.9×10^{-6}$	-0.76	0.45	-2.03	0.042
	6p22.1	ENSG00000158691	*ZSCAN12*	4.45	$8.6×10^{-6}$	-3.27	$1.1×10^{-3}$	4.46	$8.1×10^{-6}$
	6p22.1	ENSG00000187626	*ZKSCAN4*	-4.42	$1.0×10^{-5}$	-4.79	$1.7×10^{-6}$	-4.79	$1.7×10^{-6}$
	19q13.12	ENSG00000126243	*LRFN3*	4.34	$1.4×10^{-5}$	4.14	$3.5×10^{-5}$	4.47	$7.8×10^{-6}$
	9q21.11	ENSG00000226337	*RP11-274B18.4*	-4.22	$2.5×10^{-5}$	-	-	-	-
	19q13.12	ENSG00000011600	*TYROBP*	-4.22	$2.5×10^{-5}$	4.22	$2.5×10^{-5}$	-	-
	6p22.1	ENSG00000197062	*ZSCAN26*	4.18	$2.9×10^{-5}$	4.33	$1.5×10^{-5}$	3.86	$1.1×10^{-4}$
	6p22.1	ENSG00000272468	*RP1-86C11.7*	4.17	$3.1×10^{-5}$	-0.93	0.35	-	-
	6p21.32	ENSG00000204257	*HLA-DMA*	-4.15	$3.4×10^{-5}$	-4.11	3.9x10-5	4.16	$3.2×10^{-5}$
	6p22.1	ENSG00000204613	*TRIM10*	4.10	$4.2×10^{-5}$	-	-	-	-
	6p21.33	ENSG00000244731	*C4A*	4.01	$6.1×10^{-5}$	4.24	$2.2×10^{-5}$	5.60	$2.2×10^{-8}$
	6p21.32	ENSG00000241106	*HLA-DOB*	3.97	$7.1×10^{-5}$	4.54	$5.6×10^{-6}$	-1.10	0.27
	6p21.33	ENSG00000204435	*CSNK2B*	-3.97	$7.1×10^{-5}$	6.41	$1.4×10^{-10}$	-3.45	$5.5×10^{-4}$
	6p21.32	ENSG00000204301	*NOTCH4*	-3.93	$8.4×10^{-5}$	4.70	$2.6×10^{-6}$	0.84	0.40
	6p21.32	ENSG00000204308	*RNF5*	3.66	$2.5×10^{-4}$	5.59	$2.3×10^{-8}$	5.96	$2.5×10^{-9}$
	6p21.33	ENSG00000204516	*MICB*	-3.62	$3.0×10^{-4}$	2.97	$3.0×10^{-3}$	-8.06	$7.4×10^{-16}$
EBV p18	6p22.1	ENSG00000233822	*HIST1H2BN*	3.36	$7.8×10^{-4}$	0.83	0.41	4.69	$2.8×10^{-6}$
	6p21.32	ENSG00000204267	*TAP2*	-3.09	$2.0×10^{-3}$	-4.22	$2.4×10^{-5}$	-4.09	$4.2×10^{-5}$
	6p21.33	ENSG00000204387	*C6orf48*	3.04	$2.3×10^{-3}$	3.78	$1.5×10^{-4}$	4.85	$1.2×10^{-6}$
	6p21.33	ENSG00000204482	*LST1*	3.00	$2.7×10^{-3}$	-	-	-4.52	$6.1×10^{-6}$
	6p21.33	ENSG00000204520	*MICA*	2.52	0.012	1.90	0.057	5.60	$2.1×10^{-8}$
	6p21.33	ENSG00000272221	*XXbac-BPG181B23.7*	2.09	0.037	4.52	$6.1×10^{-6}$	-0.37	0.71
	6p21.32	ENSG00000204228	*HSD17B8*	1.87	0.062	4.81	$1.5×10^{-6}$	2.47	0.014
	5q31.1	ENSG00000164402	*SEPTIN8*	1.65	0.098	4.42	$9.9×10^{-6}$	-1.95	0.051
	6p21.32	ENSG00000204252	*HLA-DOA*	-1.34	0.18	6.56	$5.5×10^{-11}$	-1.13	0.26
	6p22.2	ENSG00000111801	*BTN3A3*	-0.93	0.35	-4.68	$2.8×10^{-6}$	0.25	0.80
	6p21.32	ENSG00000242574	*HLA-DMB*	-0.87	0.38	-4.40	$1.1×10^{-5}$	-3.24	$1.2×10^{-3}$
	6p21.33	ENSG00000204472	*AIF1*	0.86	0.39	-3.01	$2.6×10^{-3}$	-4.21	$2.6×10^{-5}$
	6p21.32	ENSG00000204314	*PRRT1*	0.68	0.50	-	-	-5.91	$3.5×10^{-9}$
	6p21.33	ENSG00000226979	*LTA*	-0.65	0.51	-6.72	$1.8×10^{-11}$	-6.64	$3.1×10^{-11}$
	6p22.1	ENSG00000198315	*ZKSCAN8*	0.62	0.53	5.15	$2.7×10^{-7}$	-0.01	1.00
	6p21.32	ENSG00000204248	*COL11A2*	-0.60	0.55	4.15	$3.3×10^{-5}$	1.64	0.10
	6p21.32	ENSG00000196735	*HLA-DQA1*	0.50	0.62	6.81	$1.0×10^{-11}$	1.01	0.31
	6p21.33	ENSG00000232810	*TNF*	0.48	0.63	0.48	0.63	-10.43	$1.8×10^{-25}$
	22q13.1	ENSG00000278195	*SSTR3*	0.23	0.82	-4.70	$2.6×10^{-6}$	1.23	0.22
	6p21.33	ENSG00000204531	*POU5F1*	-	-	-5.64	$1.7×10^{-8}$	-5.84	$5.2×10^{-9}$

EBV p18	6p21.33	ENSG00000228022	*HCG20*	-	-	5.10	3.4×10^{-7}	5.70	1.2×10^{-8}
	6p21.33	ENSG00000204389	*HSPA1A*	-	-	-	-	5.65	1.6×10^{-8}
	6p22.1	ENSG00000184357	*HIST1H1B*	-	-	-	-	-4.85	1.2×10^{-6}
	6p21.33	ENSG00000137337	*MDC1*	-	-	-	-	4.76	1.9×10^{-6}
	6p21.33	ENSG00000204540	*PSORS1C1*	-	-	4.99	6.0×10^{-7}	4.15	3.3×10^{-5}
	6p21.33	ENSG00000168631	*DPCR1*	-	-	-	-	4.10	4.1×10^{-5}
	6p21.33	ENSG00000228789	*HCG22*	-	-	-4.33	1.5×10^{-5}	3.03	2.4×10^{-3}
	6p21.32	ENSG00000196126	*HLA-DRB1*	-	-	7.02	2.2×10^{-12}	-	-
EBV ZEBRA	6p21.32	ENSG00000232629	*HLA-DQB2*	8.87	7.6×10^{-19}	8.54	1.4×10^{-17}	5.29	1.3×10^{-7}
	6p21.32	ENSG00000179344	*HLA-DQB1*	-8.56	1.1×10^{-17}	1.04	0.30	-11.40	4.1×10^{-30}
	6p21.32	ENSG00000204257	*HLA-DMA*	-7.28	3.4×10^{-13}	-7.45	9.5×10^{-14}	7.32	2.6×10^{-13}
	6p21.33	ENSG00000214894	*LINC00243*	-6.89	5.6×10^{-12}	-	-	-0.22	0.82
	6p21.33	ENSG00000244731	*C4A*	6.80	1.1×10^{-11}	6.46	1.0×10^{-10}	6.54	6.0×10^{-11}
	6p21.33	ENSG00000166278	*C2*	-6.42	1.4×10-^{10}	-	-	-	-
	6p21.33	ENSG00000204386	*NEU1*	6.39	1.6×10^{-10}	-	-	-2.35	0.019
	6p21.33	ENSG00000213719	*CLIC1*	6.20	5.6×10^{-10}	6.20	5.6×10^{-10}	-0.45	0.65
	6p21.33	ENSG00000224389	*C4B*	-6.17	7.0×10^{-10}	-	-	-	-
	6p21.33	ENSG00000231852	*CYP21A2*	-6.16	7.5×10^{-10}	-	-	-	-
	6p21.33	ENSG00000204444	*APOM*	6.13	8.6×10^{-10}	5.97	2.3×10^{-9}	6.18	6.6×10^{-10}
	6p21.33	ENSG00000228727	*SAPCD1*	-6.06	1.4×10^{-9}	-1.18	0.24	-6.06	1.4×10^{-9}
	6p21.33	ENSG00000146112	*PPP1R18*	-5.71	1.1×10^{-8}	0.84	0.40	-5.77	8.1×10^{-9}
	6p21.33	ENSG00000204351	*SKIV2L*	5.70	1.2×10^{-8}	5.76	8.2×10^{-9}	6.13	8.7×10^{-10}
	6p21.33	ENSG00000204396	*VWA7*	5.49	4.0×10^{-8}	-4.62	3.9×10^{-6}	4.31	1.6×10^{-5}
	6p21.33	ENSG00000204392	*LSM2*	-5.43	5.7×10^{-8}	-5.01	5.4×10^{-7}	-5.01	5.4×10^{-7}
	6p21.33	ENSG00000227507	*LTB*	5.28	1.3×10^{-7}	-	-	-	-
	6p21.33	ENSG00000204388	*HSPA1B*	-5.27	1.4×10^{-7}	-	-	-2.17	0.030
	6p21.33	ENSG00000204564	*C6orf136*	5.06	4.1×10^{-7}	0.38	0.70	5.05	4.4×10^{-7}
	6p21.32	ENSG00000204315	*FKBPL*	5.03	4.9×10^{-7}	-0.60	0.55	-	-
	6p21.32	ENSG00000204252	*HLA-DOA*	-4.98	6.5×10^{-7}	1.48	0.14	-4.95	7.5×10^{-7}
	6p21.33	ENSG00000204536	*CCHCR1*	-4.96	7.0×10^{-7}	-5.72	1.1×10^{-8}	-1.37	0.17
	6p21.33	ENSG00000204463	*BAG6*	-4.86	1.2×10^{-6}	0.80	0.42	1.20	0.23
	6p22.1	ENSG00000204592	*HLA-E*	-4.66	3.2×10^{-6}	-4.66	3.2×10^{-6}	-4.66	3.2×10^{-6}
	6p22.1	ENSG00000204613	*TRIM10*	4.60	4.2×10^{-6}	-	-	-	-
	6p21.33	ENSG00000204525	*HLA-C*	-4.60	4.3×10^{-6}	1.19	0.23	-5.39	7.1×10^{-8}
	6p21.32	ENSG00000196735	*HLA-DQA1*	-4.57	5.0×10^{-6}	-6.72	1.8×10^{-11}	-4.09	4.4×10^{-5}
	22q11.21	ENSG00000185608	*MRPL40*	4.49	7.1×10^{-6}	3.96	7.5×10^{-5}	4.48	7.5×10^{-6}
	4q24	ENSG00000109320	*NFKB1*	-4.37	1.2×10^{-5}	3.88	1.1×10^{-4}	0.94	0.35
	6p21.33	ENSG00000204394	*VARS*	4.32	1.5×10^{-5}	0.44	0.66	0.44	0.66
	6p22.1	ENSG00000281831	*HCP5B*	-4.29	1.8×10^{-5}	-5.24	1.6×10^{-7}	-4.40	1.1×10^{-5}
	6p21.32	ENSG00000241404	*EGFL8*	-4.26	2.0×10^{-5}	-2.58	9.8×10^{-3}	-4.90	9.6×10^{-7}

	6p22.1	ENSG00000204625	*HCG9*	-4.16	3.1×10^{-5}	-	-	-	-
	6p21.33	ENSG00000204356	*NELFE*	-4.14	3.4×10^{-5}	0.77	0.44	-	-
	6p22.1	ENSG00000158691	*ZSCAN12*	4.10	4.1×10^{-5}	-0.49	0.63	3.19	1.4×10^{-3}
	6p21.33	ENSG00000204428	*LY6G5C*	-3.48	5.1×10^{-4}	-3.48	5.1×10^{-4}	-6.49	8.8×10^{-11}
	6p22.1	ENSG00000204619	*PPP1R11*	3.39	7.1×10^{-4}	0.08	0.94	5.08	3.8×10^{-7}
	6p21.33	ENSG00000204438	*GPANK1*	2.81	4.9×10^{-3}	2.21	0.027	4.36	1.3×10^{-5}
	6p21.32	ENSG00000242574	*HLA-DMB*	2.70	6.9×10^{-3}	-7.33	2.3×10^{-13}	0.72	0.47
	6p21.32	ENSG00000204261	*PSMB8-AS1*	-2.67	7.6×10^{-3}	2.36	0.018	4.26	2.0×10^{-5}
	6p21.32	ENSG00000204267	*TAP2*	-2.21	0.027	-4.76	2.0×10^{-6}	-3.92	9.0×10^{-5}
	6p21.33	ENSG00000226979	*LTA*	2.05	0.040	-6.15	7.6×10^{-10}	-5.29	1.3×10^{-7}
	6p22.1	ENSG00000198315	*ZKSCAN8*	2.00	0.046	4.54	5.7×10^{-6}	-0.78	0.44
	6p21.32	ENSG00000231389	*HLA-DPA1*	1.75	0.079	5.52	3.5×10^{-8}	2.44	0.015
	6p21.33	ENSG00000204482	*LST1*	1.53	0.13	-	-	-6.81	1.0×10^{-11}
EBV ZEBRA	6p21.33	ENSG00000213760	*ATP6V1G2*	1.44	0.15	4.23	2.4×10^{-5}	2.55	0.011
	6p21.33	ENSG00000204435	*CSNK2B*	-1.25	0.21	4.91	9.3×10^{-7}	-3.55	3.9×10^{-4}
	6p21.33	ENSG00000204410	*MSH5*	-0.82	0.41	5.67	1.4×10^{-8}	-0.02	0.98
	6p21.32	ENSG00000221988	*PPT2*	-0.59	0.55	4.90	9.6×10^{-7}	-3.68	2.3×10^{-4}
	6p21.33	ENSG00000137312	*FLOT1*	0.54	0.59	-5.77	8.1×10^{-9}	-1.41	0.16
	6p21.32	ENSG00000198502	*HLA-DRB5*	-	-	-	-	-14.09	4.2×10^{-45}
	6p21.32	ENSG00000204287	*HLA-DRA*	-	-	-	-	8.48	2.2×10^{-17}
	6p21.33	ENSG00000204531	*POU5F1*	-	-	0.64	0.52	-6.11	9.9×10^{-10}
	6p21.33	ENSG00000204574	*ABCF1*	-	-	-	-	-5.74	9.3×10^{-9}
	6p21.33	ENSG00000204540	*PSORS1C1*	-	-	6.24	4.4×10^{-10}	5.25	1.5×10^{-7}
	6p21.32	ENSG00000168394	*TAP1*	-	-	-2.30	0.022	4.76	2.0×10^{-6}
	6p21.33	ENSG00000228022	*HCG20*	-	-	5.05	4.4×10^{-7}	3.92	8.9×10^{-5}
	6p21.32	ENSG00000196126	*HLA-DRB1*	-	-	-12.01	3.3×10^{-33}	-	-
	6p22.1	ENSG00000271755	*RP1-153G14.4*	-	-	4.16	3.2×10^{-5}	-	-

Table S26: TWAS results for MCV antigen response using gene expression models based on whole blood, brain, and skin tissues. Associations with $P_{TWAS}<4.2\times10^{-6}$ are considered statistically significant and genes with $P_{TWAS}<4.5\times10^{-5}$ are considered suggestive.

Region	Gene ID	Gene	Whole Blood		Brain (Frontal Cortex)		Skin (Sun Exposed)		Skin (Sun Unexposed)	
			Z	P_{TWAS}	Z	P_{TWAS}	Z	P_{TWAS}	Z	P_{TWAS}
6p21.33	ENSG00000204396	*VWA7*	-5.89	4.0×10^{-9}	5.42	6.0×10^{-8}	-4.91	9.0×10^{-7}	-4.85	1.2×10^{-6}
6p21.33	ENSG00000204371	*EHMT2*	-5.88	4.0×10^{-9}	-	-	-2.68	7.5×10^{-3}	-2.66	7.9×10^{-3}
6p21.33	ENSG00000204564	*C6orf136*	-5.76	8.2×10^{-9}	-0.97	0.33	-2.75	6.0×10^{-3}	-2.58	9.9×10^{-3}
6p21.33	ENSG00000213722	*DDAH2*	-5.68	1.4×10^{-8}	-1.49	0.14	-3.94	8.2×10^{-5}	-	-
5q31.2	ENSG00000170464	*DNAJC18*	4.82	1.5×10^{-6}	6.07	1.3×10^{-9}	3.39	6.9×10^{-4}	3.40	6.7×10^{-4}
6p21.33	ENSG00000227507	*LTB*	-4.72	2.3×10^{-6}	-	-	-	-	-	-
5q31.2	ENSG00000228672	*PROB1*	-4.64	3.6×10^{-6}	-3.29	1.0×10^{-3}	-	-	-6.75	1.5×10^{-11}
6p21.33	ENSG00000204388	*HSPA1B*	-4.48	7.4×10^{-6}	-	-	-	-	-	-
6p22.1	ENSG00000204599	*TRIM39*	-4.36	1.3×10^{-5}	-0.43	0.67	0.20	0.84	-0.11	0.91
5q31.2	ENSG00000170482	*SLC23A1*	-4.34	1.4×10^{-5}	-4.07	4.7×10^{-5}	4.39	1.1×10^{-5}	4.07	4.7×10^{-5}
5q31.2	ENSG00000170469	*SPATA24*	4.20	2.7×10^{-5}	-	-	4.30	1.7×10^{-5}	4.29	1.8×10^{-5}
6p22.1	ENSG00000204632	*HLA-G*	4.11	3.9×10^{-5}	-1.45	0.15	0.19	0.85	0.11	0.92
5q31.2	ENSG00000184584	*TMEM173*	-4.08	4.5×10^{-5}	-4.28	1.9×10^{-5}	-4.17	3.0×10^{-5}	-4.20	2.6×10^{-5}
6p21.32	ENSG00000241106	*HLA-DOB*	-3.84	1.2×10^{-4}	-5.30	1.2×10^{-7}	-3.59	3.3×10^{-4}	-4.73	2.3×10^{-6}
6p22.1	ENSG00000204619	*PPP1R11*	-3.46	5.4×10^{-4}	0.64	0.52	4.21	2.5×10^{-5}	4.20	2.7×10^{-5}
6p21.32	ENSG00000204267	*TAP2*	3.32	9.1×10^{-4}	3.11	1.9×10^{-3}	5.52	3.4×10^{-8}	3.18	1.5×10^{-3}
6p21.33	ENSG00000226979	*LTA*	2.94	3.3×10^{-3}	5.07	4.0×10^{-7}	0.88	0.38	1.33	0.18
6p21.32	ENSG00000204314	*PRRT1*	-2.80	5.1×10^{-3}	-	-	5.75	9.2×10^{-9}	5.72	1.1×10^{-8}
5q31.2	ENSG00000249751	*ECSCR*	2.38	0.018	7.83	5.0×10^{-15}	7.25	4.2×10^{-13}	7.83	$5.0\times10-^{15}$
6p22.1	ENSG00000234127	*TRIM26*	-2.24	0.025	-1.64	0.10	-6.21	5.3×10^{-10}	-5.47	4.6×10^{-8}
6p21.33	ENSG00000204498	*NFKBIL1*	-2.19	0.029	-1.94	0.052	-4.16	3.2×10^{-5}	-3.32	9.0×10^{-4}
6p21.32	ENSG00000204305	*AGER*	0.91	0.36	5.83	5.6×10^{-9}	1.87	0.062	0.28	0.78
5q31.2	ENSG00000131508	*UBE2D2*	-	-	4.33	1.5×10^{-5}	-	-	-	-

Table S27: TWAS results for seropositivity using gene expression models based on whole blood, brain, and skin tissues. Only statistically significant associations with P_{TWAS}<4.2×10^{-6} are presented.

Antigen	Region	Gene ID	Gene	Whole Blood		Brain (Frontal Cortex)	
				Z	P_{TWAS}	Z	P_{TWAS}
EBV EA-D[1]	6p21.33	ENSG00000204388	*HSPA1B*	-6.33	2.5×10^{-10}	-	-
	6p21.33	ENSG00000204371	*EHMT2*	-5.77	8.0×10^{-9}	-	-
	6p21.33	ENSG00000244731	*C4A*	5.58	2.4×10^{-8}	5.27	1.4×10^{-7}
	6p21.33	ENSG00000166278	*C2*	-4.88	1.0×10^{-6}	-	-
	6p21.33	ENSG00000204469	*PRRC2A*	4.84	1.3×10^{-6}	-	-
	6p21.33	ENSG00000231852	*CYP21A2*	-4.78	1.7×10^{-6}	-	-
	6p21.33	ENSG00000213719	*CLIC1*	4.76	2.0×10^{-6}	4.76	2.0×10^{-6}
	6p21.33	ENSG00000224389	*C4B*	-4.76	2.0×10^{-6}	-	-
	6p21.33	ENSG00000204386	*NEU1*	4.75	2.1×10^{-6}	-	-
	6p22.3	ENSG00000137177	*KIF13A*	-4.65	3.3×10^{-6}	-	-
	6p21.32	ENSG00000196126	*HLA-DRB1*	-	-	-6.87	6.4×10^{-12}
	6p21.32	ENSG00000204305	*AGER*	-	-	5.77	8.1×10^{-9}
	6p21.33	ENSG00000204420	*MPIG6B*	-	-	4.71	2.5×10^{-6}
	6p21.32	ENSG00000213676	*ATF6B*	-	-	4.80	1.6×10^{-6}
EBV EBNA[1]	6p21.32	ENSG00000179344	*HLA-DQB1*	8.37	5.6×10^{-17}	-	-
	6p21.32	ENSG00000232629	*HLA-DQB2*	-8.10	5.7×10^{-16}	-7.95	1.9×10^{-15}
	6p21.32	ENSG00000237541	*HLA-DQA2*	-5.46	4.8×10^{-8}	-	-
	6p21.32	ENSG00000196126	*HLA-DRB1*	-	-	7.78	7.0×10^{-15}
	6p21.32	ENSG00000196735	*HLA-DQA1*	-	-	6.91	4.7×10^{-12}
	6p21.32	ENSG00000204252	*HLA-DOA*	-	-	4.79	1.7×10^{-6}
	6p21.32	ENSG00000231389	*HLA-DPA1*	-	-	-5.20	2.0×10^{-7}
EBV ZEBRA[1]	6p21.33	ENSG00000244731	*C4A*	4.64	3.5×10^{-6}	-	-
	6p21.32	ENSG00000196126	*HLA-DRB1*	-	-	-5.95	2.7×10^{-9}
VZV[1]	4p15.2	ENSG00000109680	*TBC1D19*	-4.96	7.2×10^{-7}	-	-
JCV	6p21.32	ENSG00000237541	*HLA-DQA2*	9.68	3.5×10^{-22}	-	-
	6p21.33	ENSG00000204371	*EHMT2*	-9.30	1.4×10^{-20}	-	-
	6p21.32	ENSG00000179344	*HLA-DQB1*	-8.05	8.4×10^{-16}	-	-
	6p21.32	ENSG00000232629	*HLA-DQB2*	7.98	1.5×10^{-15}	7.85	4.3×10^{-15}
	6p21.32	ENSG00000204305	*AGER*	7.82	5.3×10^{-15}	10.39	2.8×10^{-25}
	6p21.33	ENSG00000213722	*DDAH2*	-7.46	8.5×10^{-14}	-	-
	6p21.32	ENSG00000204310	*AGPAT1*	-6.80	1.0×10^{-11}	7.80	6.0×10^{-15}
	6p21.32	ENSG00000204308	*RNF5*	-6.13	9.0×10^{-10}	-	-
	6p21.33	ENSG00000204396	*VWA7*	-5.64	1.7×10^{-8}	4.98	6.4×10^{-7}
	6p21.33	ENSG00000227507	*LTB*	-5.56	2.8×10^{-8}	-	-
	6p21.33	ENSG00000204463	*BAG6*	5.30	1.2×10^{-7}	-	-
	6p21.32	ENSG00000196126	*HLA-DRB1*	-	-	-9.995	1.6×10^{-23}

	6p21.32	ENSG00000196735	*HLA-DQA1*	-	-	-5.73	9.8×10^{-9}
	6p21.32	ENSG00000204252	*HLA-DOA*	-	-	-5.90	3.6×10^{-9}
	6p21.32	ENSG00000204261	*PSMB8-AS1*	-	-	-5.36	8.1×10^{-8}
	6p21.32	ENSG00000204264	*PSMB8*	-	-	5.71	1.2×10^{-8}
	6p21.32	ENSG00000204301	*NOTCH4*	-	-	-5.85	5.0×10^{-9}
	6p21.33	ENSG00000213760	*ATP6V1G2*	-	-	-4.98	6.2×10^{-7}
	6p21.33	ENSG00000226979	*LTA*	-	-	5.81	6.2×10^{-9}
MCV	6p21.33	ENSG00000204371	*EHMT2*	-8.64	5.8×10^{-18}	-	-
	6p21.33	ENSG00000213722	*DDAH2*	-8.49	2.2×10^{-17}	-4.69	2.7×10^{-6}
	6p21.33	ENSG00000227507	*LTB*	-8.24	1.7×10^{-16}	-	-
	6p21.33	ENSG00000204396	*VWA7*	-6.20	5.7×10^{-10}	5.40	6.7×10^{-8}
	6p21.32	ENSG00000204305	*AGER*	5.27	1.4×10^{-7}	9.35	9.0×10^{-21}
	6p21.32	ENSG00000240065	*PSMB9*	5.18	2.2×10^{-7}	-	-
	6p21.33	ENSG00000146112	*PPP1R18*	5.11	3.2×10^{-7}	-	-
	6p21.32	ENSG00000241106	*HLA-DOB*	-4.75	2.0×10^{-6}	-5.79	7.1×10^{-9}
	6p21.32	ENSG00000237541	*HLA-DQA2*	4.72	2.3×10^{-6}	-	-
	6p21.32	ENSG00000179344	*HLA-DQB1*	-	-	-6.79	1.1×10^{-11}
	6p21.32	ENSG00000196126	*HLA-DRB1*	-	-	-5.41	6.4×10^{-8}
	6p21.32	ENSG00000196735	*HLA-DQA1*	-	-	-6.48	9.4×10^{-11}
	6p21.32	ENSG00000204310	*AGPAT1*	-	-	4.92	8.5×10^{-7}
	6p21.33	ENSG00000226979	*LTA*	-	-	8.84	9.9×10^{-19}
	6p21.33	ENSG00000228789	*HCG22*	-	-	5.40	6.5×10^{-8}
	5q31.2	ENSG00000249751	*ECSCR*	-	-	5.63	1.8×10^{-8}

Table S28: Replication results for previously reported genome-wide significant associations for variants and classical HLA alleles for selected antigens. Replication was restricted to findings from genome-wide association studies of serological phenotypes (IgG levels or seropositivity) conducted in primarily non-diseased populations or controls. Variants reported in multiple studies are listed under the earlier publication.

Study	Population	Antigen	Phenotype	Variant/Allele	Beta/OR	P
Hammer et. al (2015) PMID: 26456283	European ancestry (N=2,363)	EBV EBNA	IgG	DRβ1: Ser-11 Ser-13 His-96 Arg-133	0.017	0.30
				DRβ1: Gly-11 Tyr-13 His-96 Arg-133	-0.231	1.4E-21
				DRβ1: Pro-11 Arg-13 Gln-96 Leu-133	0.243	1.5E-27
				DRβ1: Leu-11 Phe-13 Glu-96* Arg-133	-	-
				rs6927022-A	0.204	6.0E-33
				HLA-DRb1 (11)	-	2.6E-49
				DRB1*07:01	-0.230	3.8E-21
				DRB1*15:01	0.253	7.8E-28
				DQB1*06:02	0.244	8.5E-26
				DRB1*03:01	-0.209	6.5E-19
				DRB1*04:01	-0.118	8.2E-03
				DRB1*15:01-DQB1*06:02	0.292	6.3E-27
				DRB1*15:01-DQB1*06:02-DQA1*01:02	0.305	2.5E-28
		JCV	Serostatus	DRβ1: Ser-11 Ser-13 His-96 Arg-133	1.24	2.9E-11
				DRβ1: Gly-11 Tyr-13 His-96 Arg-133	1.09	0.058
				DRβ1: Pro-11 Arg-13 Gln-96 Leu-133	0.54	1.4E-42
				DRβ1: Leu-11 Phe-13 Glu-96* Arg-133	-	-
				rs9269910-A	1.83	1.6E-41
				HLA-DRb1 (133)	-	1.4E-42
				HLA-DQA1*01:02	0.61	2.4E-34
				HLA-DRB1*15:01	0.54	6.7E-41
				DRB1*15:01-DQB1*06:02	0.49	7.4E-41
				DRB1*15:01-DQB1*06:02-DQA1*01:02	0.48	7.8E-42
			IgG	rs1049130-G	0.117	1.3E-09
		MCV	Serostatus	DRβ1: Ser-11 Ser-13 His-96 Arg-133	1.33	2.5E-16
				DRβ1: Gly-11 Tyr-13 His-96 Arg-133	1.08	0.13
				DRβ1: Pro-11 Arg-13 Gln-96 Leu-133	0.627	3.9E-25
				DRβ1: Leu-11 Phe-13 Glu-96* Arg-133	-	-
				rs9269268-C	1.60	1.4E-25
				DRb1 (13)	-	8.5E-28
				DQB1*06:02	0.62	4.0E-25
				DRB1*15:01	0.62	2.6E-24
				DRB1*15:01-DQB1*06:02	0.56	6.4E-26
				DRB1*15:01-DQB1*06:02-DQA1*01:02	0.56	9.3E-26
Rubicz et al. (2013) PMID: 23326239	Mexican Americans (N=1367)	EBV EBNA	IgG	rs477515-G	0.212	4.3E-33
				rs2854275-C	0.214	1.1E-19
				rs3130048-T	0.142	1.9E-14
				rs204999-A	0.187	2.7E-25
				rs4248166-T	-0.045	0.043
			Serostatus	rs652888-A	1.22	3.9E-04

				rs204999-A		1.26	9.2E-06
				rs4248166-T		0.85	0.018
				rs2294884-T		0.86	0.033
				rs2294882-T		0.86	0.028
				rs2294881-T		0.86	0.034
				rs28362680-G		0.89	0.28
				rs28362683-G		0.90	0.34
				rs10947261-G		0.90	0.30
				rs10947262-C		0.91	0.34
				rs7192-T		1.19	7.6E-04
				rs2239803-C		1.22	9.4E-05
				rs7194-G		1.19	8.4E-04
				rs7195-A		1.19	7.8E-04
				rs2213586-A		1.19	9.3E-04
				rs2213585-G		1.19	7.4E-04
				rs2227139-G		1.19	7.4E-04
				rs7754768-C		1.22	1.3E-04
				rs9268832-T		1.22	1.1E-04
Scepanovic et al. (2018)<br>PMID: 30053915	French, predominantly European ancestry (N=1000)	EBV EBNA	IgG	DQB1*03:01		0.055	0.014
				HLA-DRβ1 (58) *		-	-
				HLA-DRβ1 (56) **		-	-
				rs74951723-A ***		-	-
				rs9469200-C ****		0.311	1.3E-26
Sundqvist et al. (2014)<br>PMID: 24763718	Swedish (N=2865)	JCV	Serostatus	DQB1*03:01		0.189	1.3E-05
Chen et al. (2011)<br>PMID: 21896673	European ancestry (N=4811)	HPV8	Serostatus	rs9357152-G	HPV16 L1	1.27	7.8E-03
					HPV16 E6/E7	1.04	0.65

* Unable to assess replication: amino acid sequences at this position were not well imputed in UK Biobank data (INFO<0.30)
** Unable to assess replication: amino acid sequences at this position were not available in reference panel
*** Unable to assess replication: no available LD proxy in our GWAS data
**** Replication results for variant with the second-lowest p-value (rs9469200: P=9.2×10^{-14} ; LD with rs74951723: r^2=0.30)
